# Synthesis and Biotransformation of Bicyclic Unsaturated Lactones with Three or Four Methyl Groups

**DOI:** 10.3390/molecules22010147

**Published:** 2017-01-17

**Authors:** Katarzyna Wińska, Małgorzata Grabarczyk, Wanda Mączka, Adrianna Kondas, Gabriela Maciejewska, Radosław Bonikowski, Mirosław Anioł

**Affiliations:** 1 Department of Chemistry, Wrocław University of Environmental and Life Sciences, Norwida 25, 50-375 Wrocław, Poland; magrab@onet.pl (M.G.); wanda_m19@o2.pl (W.M.); adakondas@gmail.com (A.K.); miroslaw.aniol@up.wroc.pl (M.A.); 2 Faculty of Chemistry, Wrocław University of Technology, Wybrzeże Wyspiańskiego 27, 50-370 Wrocław, Poland; gabriela.maciejewska@pwr.edu.pl; 3 Institute of General Food Chemistry, Lodz University of Technology, Stefanowskiego 4/10, 90-924 Łódź, Poland; radoslaw.bonikowski@p.lodz.pl

**Keywords:** unsaturated lactones, hydroxylation, epoxidation, biotransformation

## Abstract

The aim of this study was to obtain new unsaturated lactones by chemical synthesis and their microbial transformations using fungal strains. Some of these strains were able to transform unsaturated lactones into different hydroxy or epoxy derivatives. Strains of *Syncephalastrum racemosum* and *Absidia cylindrospora* gave products with a hydroxy group introduced into a tertiary carbon, while the *Penicillium vermiculatum* strain hydroxylated primary carbons. The *Syncephalastrum racemosum* strain hydroxylated both substrates in an allylic position. Using the *Absidia cylindrospora* and *Penicillium vermiculatum* strains led to the obtained epoxylactones. The structures of all lactones were established on the basis of spectroscopic data.

## 1. Introduction

Hydroxylactones are a very important group of natural compounds. Such compounds are often encountered in Nature, mainly in plants and marine organisms. Natural hydroxylactones are widely known for their biological properties, which include cytotoxicity [1,2,3,4], anti-cancer effects [5,6], inhibition of plant growth [7,8], anti-inflammatory action [9,10] and anti-depressant properties [10]. Because lactones occur in natural sources in small amounts, their isolation is hard and expensive. From this it follows that in order to study these biologically active compounds it is necessary to obtain them by means other than isolation from their natural sources. One such method is hydroxylation of synthetically obtained lactones by means of biotransformations. Hydroxylation can be performed on different lactone derivatives, one of which are unsaturated lactones. Usually the presence of a double bond in these molecules leads to the introduction of a hydroxy group into the allylic position [11,12,13,14,15,16,17,18]. A double bond in the molecule can also undergo epoxidation [11,15]. Another possibility is the direct hydroxylation of the double bond [11,13,14,16,18] or opening of an epoxide ring [11,15,18]. The hydroxy group can also be introduced into primary [19], secondary [16,19,20] or tertiary carbons of the molecules [12,17,20,21].

The biotransformation of lactones with double bonds in their structure may thus yield different and interesting derivatives like hydroxy- or epoxylactones. Our team has been conducting studies dealing with transformations of different kinds of lactones for a long time. Previously unsaturated lactones with one, two or three methyl groups attached to the cyclohexane ring were subjected to biotransformation [22,23,24]. During these biotransformations, hydroxylation in allylic position or epoxidation of double bond was observed. Continuing our research we decided to synthesize two new unsaturated lactones with two or three methyl groups in the cyclohexane ring and one additional methyl group in the lactone ring. We hoped that it would be possible to obtain some interesting hydroxy derivatives as a result of their biotransformation.

## 2. Results and Discussion

**Scheme 1:**
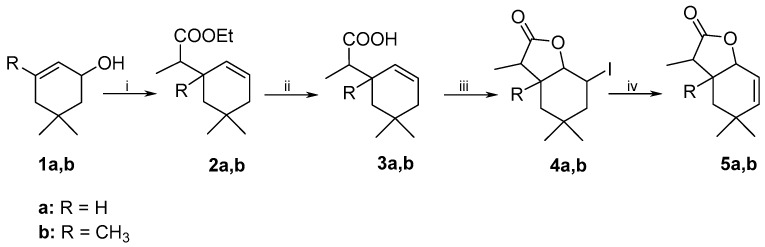
Synthesis of lactones **5a** and **5b**. *Reagents and Conditions*: (i): CH_3_CH_2_C(OC_2_H_5_)_3_, CH_3_CH_2_COOH, 137 °C (**2a**: 84%, **2b**: 82%); (ii): KOH, EtOH, 100 °C (**3a**: 96%, **3b**: 92%); (iii): I_2_, KI r.t. (**4a**: 71%, **4b**: 75%); (iv): DBU, toluene, 110 °C (**5a**: 79%,**5b**: 79%).

New unsaturated lactone biotransformation substrates **5a** and **5b** were obtained from a four-step synthesis from the corresponding known allylic alcohols. Substrates **1a** and **1b** were subjected to a Claisen rearrangement with orthopropionate modification, giving γ,δ-unsaturated esters **2a** and **2b** as two pairs of diastereoisomers (46% **A**:54% **B**) and **2b** (46% **A**:54% **B**). These esters were then hydrolyzed into the γ,δ-unsaturated acids **3a** and **3b** also as pairs of diastereoisomers (48% **A**:52% **B**) and (41% **A**:59% **B**). In the next step these acids were transformed into the corresponding iodolactones **4a** and **4b**. Both iodolactones were also obtained as mixtures of diastereoisomers **4a** (37% **A**:63% **B**); **4b** (39% **A**:61% **B**). In the last step, the diastereoisomeric mixtures of iodolactones were subjected to dehydrohalogenation, also providing diastereoisomeric mixtures of the unsaturated lactones **5a** (33% **A**:67% **B**) and **5b** (34% **A**:66% **B**). (Scheme 1).

**Figure 1:**
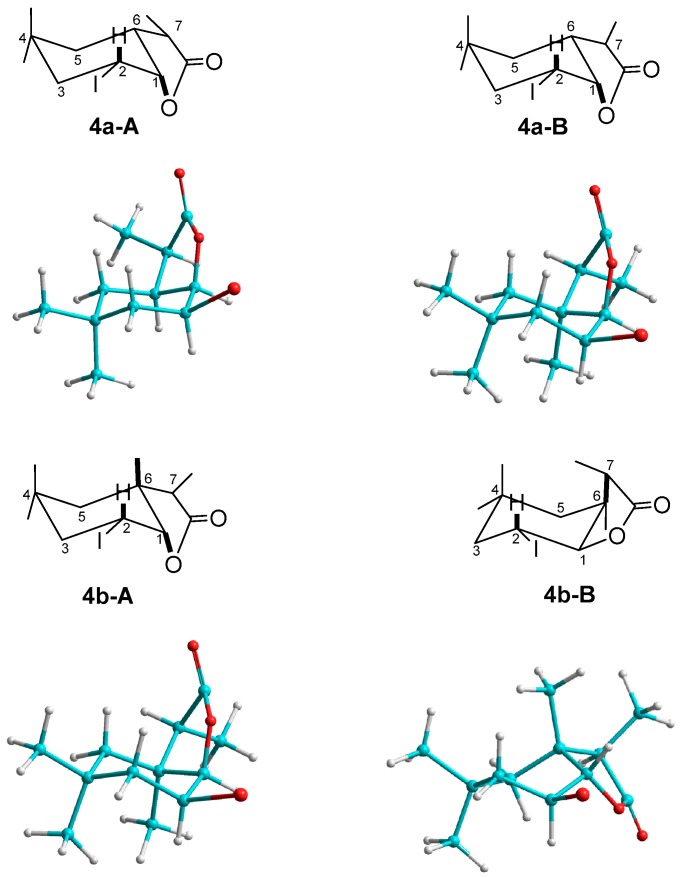
Structures of iodolactones **4a** and **4b**.

The structures of these compounds were established on the basis of their spectral data (^1^H-NMR, ^13^C-NMR, COSY, HMQC, IR) and confirmed by HRMS analysis. Because the aim of our study was to obtain new lactones, these compounds will be discussed in detail here. In the case of iodolactone **4b** and unsaturated lactone **5b**, we were able to partially separate each of the diastereoisomers in small quantities which allowed for a more accurate analysis of these compounds. Analysis of the ^1^H-NMR spectra of iodolactones **4a** and **4b** indicates the presence of two diastereoisomers named **A** and **B** Firstly the spectrum of iodolactone **4a** (as mixture of diastereoisomers **A** and **B**) will be discussed. Both signals of the H-1 protons appear as narrow singlets, suggesting an equatorial orientation of these protons. Signals corresponding to the H-2 and H-6 protons are broad multiplets, indicating their axial orientation. It follows from this that the C–O bonds in both molecules occupy an axial position. The CH_3_-11 groups are located in the same plane as the lactone ring (**4a-A**) or across this plane (**4a-B**). In the case of iodolactone **4b** it was possible to analyze each diastereoisomer separately. In the case of compound **4b-A**, the signal coming from proton H-2 is a wide multiplet, suggesting its axial orientation. The doublet with a smaller (5.0 Hz) coupling constant corresponding to the H-1 proton indicates its equatorial orientation. A different situation is observed in the case of compound **4b-B**. Both signals of the H-1 (d, *J* = 10.2 Hz) and H-2 (ddd, *J* = 13.9, 10.2 and 3.8 Hz) protons suggested their axial orientation. These observations indicated that in compound **4b-A**, the C–O bond of the lactone ring occupied an axial position, but in compound **4b-B** this bond is in an equatorial position. In the case of isomer **4b-A**, the CH_3_-11 group is located across the plane of the lactone ring and in the case of isomer **4b-B** this group is lying in the same plane as the lactone ring (Figure 1).

Analysis of the ^1^H-NMR spectra of the unsaturated lactones **5a** and **5b** proves that these compounds are also mixtures of two diastereoisomers named **A** and **B**. The structures of these lactones are very similar to those described above for the corresponding iodolactones.

**Figure 2:**
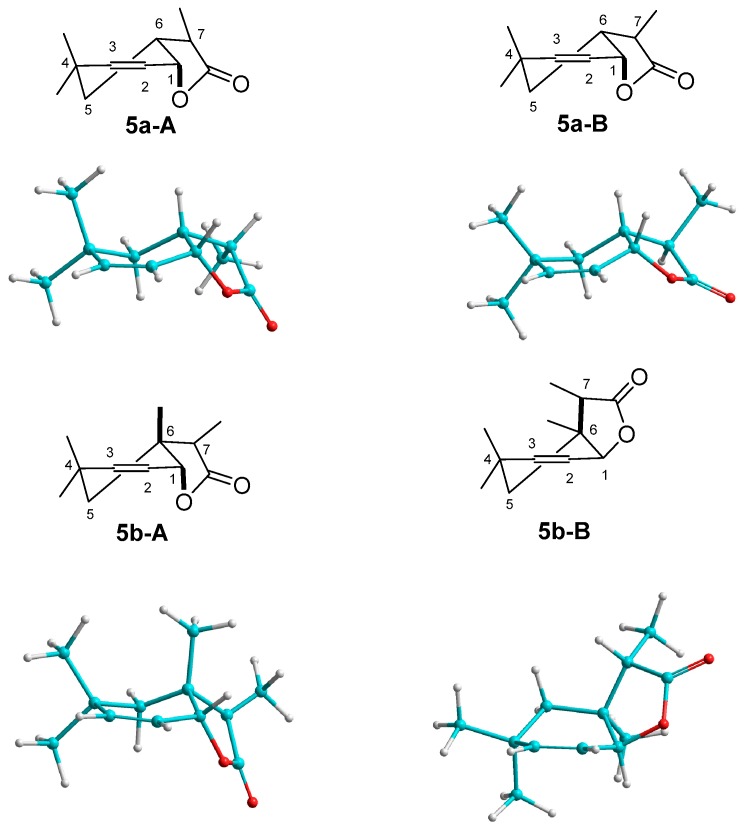
Structure of unsaturated lactones **5a** and **5b**.

The only difference is the presence of a double bond between carbons C-2 and C-3. The signals of the H-2 and H-3 protons, which look like wide multiplets for lactone **5a**, suggests their axial orientation. In the case of lactone **5b**, the coupling constant between protons H-2 and H-3 is 10.2 Hz (for **5b-A**) and 10.0 Hz (for **5b-B**), also indicating their axial position (Figure 2).

Both unsaturated lactones **5a** and **5b** (as mixtures of diastereoisomers) were used as biotransformation substrates. During the screening biotransformation, the ability of some fungal strains to convert lactones **5a** and **5b** into any products was checked. From the fourteen different strains (*Fusarium* sp., *Penicilium* sp., *Absidia* sp. and *Syncephalastrum racemosum*) examined only some of them showed any ability to transform the substrates into some derivatives. These positive results obtained during screening biotransformation using these five strains are presented in Table 1 and Table 2.

As it can be seen in the above tables, the unsaturated lactones **5a** and **5b** were converted into different products by three (**5a**) or five strains (**5b**), respectively Taking into consideration the yields of these processes, the strains *S. racemosum* AM105, *A. cylindrospora* AM336 and *P. vermiculatum* AM30 were chosen for preparative-scale biotransformations of both substrates. The results of this are presented in Table 3 and Table 4.

**Scheme 2:**
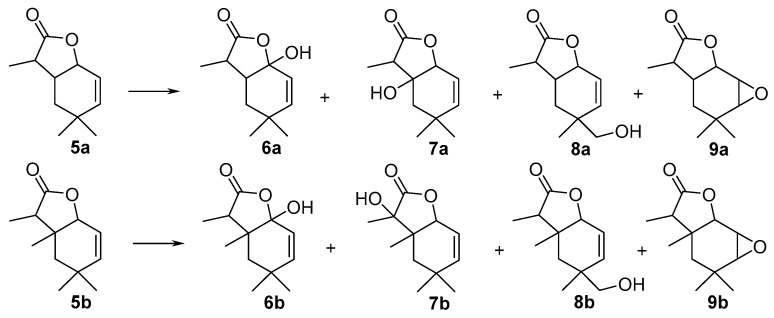
The products of the biotransformation of unsaturated lactones **5a** and **5b**.

The biotransformation results proved that each substrate was converted into four different products. One of them—lactone **7a**—was produced by all three fungal strains. Its analog—lactone **7b**—by two strains (not by *P. vermiculatum*). Lactones **8a** and **8b** were formed by the *P. vermiculatum* strain. It is worth noting that these products were formed as single isomers. The *S. racemosum* strain was able to produce lactones **6a** and **6b** as pairs of diastereoisomers. A similar situation (the formation of products observed as pairs of diastereoisomers) was observed in the case of lactones **9a** and **9b** (Scheme 2).

After the separation and purification of all compounds obtained during the preparative-scale biotransformations of lactones **5a** and **5b** it was possible to determine the quantity of each of them. These data are listed in Table 5 and Table 6.

Analysis of the ^1^H-NMR spectra of all obtained products proved that the microorganisms used here preferred hydroxylation of the tertiary carbons present in the molecules of both substrates. All three fungal strains converted substrate **5a** into hydroxylactone **7a**, in which the hydroxy group attacks the tertiary C-6 carbon. A comparison of the ^1^H-NMR spectra of the substrate with the product concluded that this product was created only from isomer **B** of the substrate. This result was very surprising because until now such a hydroxylation position was not observed in similar compounds. The *S. racemosum* strain was able to introduce the hydroxy group in an allylic position (the tertiary C-1 carbon) giving product **6a** as a pair of diastereoisomers, whereas in the case of the *P. vermiculatum* strain, the product **8a** in which the hydroxy group is introduced into the CH_3_-10 group was obtained. This compound was created only from isomer **B** of the substrate. The *A. cylindrospora* strain gave one other product—the epoxylactone **9a**—as a pair of diastereoisomers. The small coupling constants between protons H-1, H-2 and H-3 (about 3.0 Hz) in epoxylactone **9a** indicate that these protons are in equatorial positions. This observation also indicates that the epoxide ring is introduced *trans* relative to the lactone ring.

In the case of lactone **5b** some other products were also observed. Two strains (*S. racemosum* and *A. cylindrospora*) transformed this compound into lactone **7b**, in which the hydroxy group is introduced into the tertiary C-7 carbon. Taking into consideration the spatial structure of both diastereoisomers, it can be observed that for the above product, isomer **B** of the substrate is more privileged. In this case hydroxylation of carbon C-6 was not possible, because this position is occupied by the methyl group. Therefore the hydroxy group was introduced into the next tertiary carbon (C-7). Like in substrate **5a**, the *S. racemosum* strain introduced the hydroxy group in an allylic position giving product **6b**, also as a pair of diastereoisomers. The *P. vermiculatum* strain in this case produced two products—hydroxylactone **8b** with the hydroxy group introduced into the CH_3_-10 group and the epoxylactone **9b**. The first of them was created only from isomer **B** of the substrate, while the second one was a pair of diastereoisomers. In epoxylactone **9b** a small (about 3.0 Hz) coupling constant is observed between protons H-1, H-2 and H-3. This information suggests the equatorial positions of these protons and also the *trans* orientation of the epoxide ring in relation to the lactone ring.

During these transformations the formation of two kinds of products, either as a single isomer (**B**) or as a pair of diastereoisomers (**A** + **B**), was observed. This was because isomer **B** was the predominant one in the substrate mixtures.

In the next step, enantiospecificity and optical purity of hydroxylactone **7a-B**, **7b-B**, **8a-B** and **8b-B** which were obtained as single isomers during the preparative biotransformations, were determined. The results of this step are presented in Table 7.

The best enantiomeric excess (72.1%) was observed for this compound when *A. cylindrospora* was used as a biocatalyst. In the case of lactone **7a-B** the (−)-isomer was preferentially formed. The other compounds, lactones **7b-B**, **8a-B** and **8b-B**, were created with a predominance of the (+)-isomer.

## 3. Materials and Methods

### 3.1. General Methods

The progress of the reactions and biotransformation was checked by analytical TLC on silica gel-coated aluminium plates (DC-Alufolien Kieselgel 60 F254, Merck, Darmstadt, Germany) with a mixture of hexane and acetone in various ratios as eluent. Preparative column chromatography was performed on silica gel (Kieselgel 60, 230–400 mesh ASTM, Merck) with a mixture of hexane and acetone (for esters hexane–acetone 19:1, for acids hexane-acetone 3:1, for iodolactones and unsaturated lactones hexane–acetone 6:1, for biotransformations products hexane–acetone 3:1) as eluents. Compounds were detected by spraying the plates with 1% Ce(SO_4_)_2_, 2% H_3_[P(Mo_3_O_10_)_4_] in 10% H_2_SO_4_ or 20% ethanolic H_2_SO_4_, containing 0.1% anisaldehyde, followed by heating to 120 °C. GC analysis was carried out on an Agilent Technologies 6890N instrument (Agilent Technologies, Santa Clara, CA, USA) using a DB-17 column (cross-linked methyl silicone gum, 30 m × 0.32 mm × 0.25 µm). The enantiomeric compositions of the products obtained during the biotransformation were determined by Agilent Technologies 6890N GC analysis using the chiral column CP-cyclodextrin-B-110 (30 m × 0.25 mm × 0.25 µm) (Supelco, Bellefonte, PA, USA) under the following conditions: injector 200 °C, detector (FID) 200 °C, column temperature 160 °C, ramp 160–175 °C at a rate of 0.5 °C/min, ramp 175–200 °C at a rate of 10 °C/min and hold 1 min at 200 °C (for compounds **7a**, **8a**, **8b**) (Supplementary Materials, Figures S110–S107, S113). For compound **7b** enantiomeric excess was determined by a Varian CP3380 instrument (Varian, Agilent Technologies, Santa Clara, CA, USA) using a Gamma DEX 325 (30 m × 0.25 mm × 0.25 µm) chiral column (Supelco) (injector 150 °C, detector (FID) 200 °C, column temperature 130 °C, ramp 130–175 °C at a rate of 0.7 °C/min, ramp 175–200 °C at a rate of 20 °C/min) (Supplementary Materials, Figures S111 and S112). The molar masses of the obtained compounds were confirmed by high resolution mass spectrometry analysis using a Waters LCT Premier XE instrument (ESI ionization, Waters, Milford, MA, USA). NMR spectra were recorded in a CDCl_3_ solution on an Avance^TM^ 600 MHz spectrometer (600 MHz for ^1^H, 151 MHz for ^13^C, Bruker, Billerica, MA, USA). Chemical shifts are reported in reference to the residual solvent signal (δ_H_ = 7.26). IR spectra were recorded on a IR300 FT-IR spectrometer (Thermo-Nicolet, Waltham, MA, USA). Optical rotations were determined on a P-2000 polarimeter (Jasco, Easton, PA, USA) in chloroform solutions, with concentrations denoted in g/100 mL. The melting points were determined on a Boetius apparatus. The refractive index was measured on a Carl Zeiss Abbe and Pulfrich refractometer (Carl Zeiss AG, Jena, Germany).

### 3.2. Synthesis of Substrates

Two known allylic alcohols **1a** [25] and **1b** [26] were used as the starting materials for obtaining unsaturated lactones. All of the intermediates were obtained according to the procedures described below:

#### 3.2.1. Ethyl (5,5,7–Trimethylcyclohex-2-en-1-yl)acetate (**2a**) and Ethyl (1,5,5,7–tetramethylcyclohex-2-en-1-yl)acetate (**2b**)

Ester **2a** (5.0 g, 23.8 mmol, yield 84%) and **2b** (5.0 g, 22.3 mmol, yield 82%) were obtained as mixtures of two diastereoisomers from the Claisen rearrangement with orthopropionate modification [27]. They displayed the following physical and spectral properties:

**2a**: n_D_ = 1.4571; ^1^H-NMR: 0.91 (s, 3H, CH_3_-9B), 0.92 (s, 3H, CH_3_-9A), 0.96 (s, 3H, CH_3_-10A), 0.97 (s, 3H, CH_3_-10B), 1.12 (d, *J* = 7.0 Hz, 3H, CH_3_-11A), 1.16 (d, *J* = 7.0 Hz, 3H, CH_3_-11B), 1.27 (dd, *J* = 7.0 and 3.2 Hz, 3H, CH_3_-12B), 1.29 (dd, *J* = 7.2 and 3.2 Hz, 3H, CH_3_-12A), 1.33–1.34 (m, 1H, H-1B), 1.35–1.36 (m, 1H, H-1A), 1.70–1.74 (m, 2H, CH_2_-4A), 1.84–1.87 (m, 2H, CH_2_-4B), 2.33–2.36 (m, 2H, CH_2_-6B), 2.39–2.42 (m, 2H, CH_2_-6A), 2.44–2.48 (m, 1H, CH_2_-6A), 2.49–2.52 (m, 2H, CH_2_-7A, CH_2_-7B), 4.16–4.19 (m, 4H, CH_2_-12A, CH_2_-12B), 5.44–5.46 (m, 1H, H-2B), 5.58–5.60 (m, 1H, H-2A), 5.66–5.70 (m, 2H, H-3A, H-3B); ^13^C-NMR: 13.4 (C-12B), 13.6 (C-12A), 14.3 (C-13A), 14.3 (C-13B), 25.1 (C-9A), 25.1 (C-9B), 29.5 (C-5B), 29.5 (C-5A), 32.1 (C-10A), 32.2 (C-10B), 36.6 (C-7B), 36.7 (C-7A), 38.5 (C-4B), 38.89 (C-4A), 39.1 (C-6B), 40.1 (C-6A), 43.8 (C-1A), 44.1 (C-1B), 60.1 (C-12A), 60.1 (C-12B), 126.7 (C-3A), 127.2 (C-3B), 127.5 (C-2A), 128.0 (C-2B), 176.0 (C-8B), 176.1 (C-8A); IR (KBr, cm^−1^): 2952, 1732, 1464, 1365, 1180; ESIHRMS: calcd. for C_13_H_22_O_2_, *m*/*z* 211.1698 [M + H]^+^, found 211.1691. (Supplementary Materials, Figures S1–S6).

**2b**: n_D_ = 1.4645; ^1^H-NMR: 0.98 (s, 9H, CH_3_-9A, CH_3_-10A, CH_3_-10B), 0.99 (s, 3H, CH_3_-9B), 1.08 (d, *J* = 7.2 Hz, 3H, CH_3_-12A), 1.15 (d, *J* = 7.2 Hz, 3H, CH_3_-12B), 1.11 (s, 3H, CH_3_-12A), 1.14 (s, 3H, CH_3_-11B), 1.26 (t, *J* = 7.2 Hz, 3H, CH_3_-14A), 1.28 (t, *J* = 7.2 Hz, 3H, CH_3_-14B), 1.63–1.80 (m, 8H, CH_2_-4A, CH_2_-4B, CH_2_-6A, CH_2_-6A), 2.32 (q, *J* = 7.2 Hz, 1H, H-7A), 2.33 (q, *J* = 7.2 Hz, 1H, H-7B), 4.07–4.12 (m, 2H, CH_2_-13A), 4.13–4.16 (m, 2H, CH_2_-13B), 5.30 (d, *J* = 10.2 Hz, 1H, H-2B), 5.57 (d, *J* = 10.2 Hz, 1H, H-2A), 5.64 (ddd, *J* = 10.2, 5.4 and 2.4 Hz, 1H, H-3A), 5.68 (ddd, *J* = 10.2, 6.0 and 3.0 Hz, 1H, H-3B); ^13^C-NMR: 12.1 (C-12A), 12.6 (C-12B), 14.3 (C-14A), 14.4 (C-14B), 25.5 (C-11A), 25.9 (C-11B), 28.1 (C-10B), 28.2 (C-10A), 29. 9 (C-1B), 29.0 (C-1A), 32.6 (C-9A), 32.6 (C-9B), 37.6 (C-5A), 38.0 (C-5B), 38.3 (C-4B), 38.4 (C-6B), 43.7 (C-4A), 44.4 (C-6A), 50.2 (C-7B), 50.3 (C-7A), 59.8 (C-13A), 59.9 (C-13B), 125.1 (C-3A), 125.4 (C-3B), 132.7 (C-2A), 133.5 (C-2B), 175.5 (C-8A), 175.6 (C-8B); IR (KBr, cm^−1^): 2953, 1732, 1457, 1369, 1175; ESIHRMS: calcd. for C_14_H_24_O_2_, *m*/*z* 225.1855 [M + H]^+^, found 225.1860. (Supplementary Materials, Figures S47–S52).

#### 3.2.2. (5,5,7–Trimethylcyclohex-2-en-1-yl)acetic acid (**3a**) and (1,5,5,7–Tetramethylcyclohex-2-en-1-yl)acetic acid (**3b**)

Basic hydrolysis of the mixtures of two diastereoisomers of esters **3a** and **3b** (according to a previously described procedure [28] gave 3.5 g (19.2 mmol, yield 96%) of acid **3a** (two diastereoisomers and 3.6 g (18.4 mmol, yield 92%) of acid **3b** (two diastereoisomers with the following physical and spectral data:

**3a**: n_D_ = 1.4719; ^1^H-NMR: 1.00 (s, 9H, CH_3_-9B, CH_3_-10A, CH_3_-10B), 1.01 (s, 3H, CH_3_-9A), 1.12 (d, *J* = 7.5 Hz, 3H, CH_3_-11A), 1.16 (s, 2H, CH_2_-4B), 1.16 (s, 1H, H-1A), 1.17 (s, 1H, H-1B), 1.20 (s, 3H, CH_3_-11B), 1.63 (d, *J* = 14.0 Hz, 2H, CH_2_-4B), 1.74–1.75 (m, 2H, CH_2_-6A), 1.77–1.79 (m, 2H, CH_2_-4B), 2.35–2.38 (m, 2H, CH_2_-7A, CH_2_-7B), 5.34–5.36 (m, 1H, H-2B), 5.59–5.61 (m, 1H, H-2A), 5.68–5.73 (m, 2H, H-3A, H-3B); ^13^C-NMR: 12.2 (C-11A), 12.6 (C-11B), 25.8 (C-1A), 25.9 (C-1B), 28.2 (C-9A), 28.2 (C-9B), 29.9 (C-5B), 30.0 (C-5A), 32.5 (C-10A), 32.6 (C-10B), 37.9 (C-6A), 38.3 (C-6B), 43.5 (C-4B), 44.7 (C-4A), 50.1 (C-7B), 50.2 (C-7A), 125.6 (C-3A), 125.8 (C-3B), 132.2 (C-2A), 133.2 (C-2B), 181.6 (C-8B), 181.6 (C-8A); IR (KBr, cm^−1^): 2952, 1706, 1457, 1365, 1206; ESIHRMS: calcd. for C_11_H_18_O_2_Na, *m*/*z* 205.1205 [M + H]^+^, found 205.1210. (Supplementary Materials, Figures S7–S12).

**3b**: m.p. = 43–45 °C; ^1^H-NMR: 0.99 (s, 9H, CH_3_-9A, CH_3_-10A, CH_3_-10B), 1.00 (s, 3H, CH_3_-9B), 1.12 (d, *J* = 7.2 Hz, 3H, CH_3_-12B), 1.51 (s, 3H, CH_3_-11A), 1.62 (t, *J* = 7.2 Hz, 3H, CH_3_-12A), 1.20 (s, 3H, CH_3_-11B), 1.62 (d, *J* = 13.8 Hz, 2H, CH_2_-6B), 1.67–1.75 (m, 4H, CH_2_-4A, CH_2_-4B), 1.77–1.81 (m, 2H, CH_2_-6A), 2.36 (q, *J* = 7.2 Hz, 1H, H-7B), 2.37 (q, *J* = 6.6 Hz, 1H, H-7A), 5.60 (d, *J* = 10.2 Hz, 1H, H-2A), 5.57 (d, *J* = 10.2 Hz, 1H, H-2B), 5.64 (ddd, *J* = 10.2, 5.4 and 2.4 Hz, 1H, H-3A), 5.68 (ddd, *J* = 10.2, 5.4 and 2.4 Hz, 1H, H-3B); ^13^C-NMR: 12.2 (C-12A), 12.6 (C-12B), 25.3 (C-11A), 25.9 (C-11B), 28.2 (C-10A), 28.2 (C-10B), 29.9 (C-1B), 29.0 (C-1A), 32.5 (C-9A), 32.6 (C-9B), 37.8 (C-5B), 38.0 (C-5A), 38.3 (C-4A), 38.3 (C-4B), 43.5 (C-6B), 44.8 (C-6A), 50.2 (C-7B), 50.3 (C-7A), 125.6 (C-3A), 125.8 (C-3B), 132.2 (C-2A), 133.3 (C-2B), 181.8 (C-8B), 181.9 (C-8A); IR (KBr, cm^−1^): 2955, 1703, 1460, 1248, 1074; ESIHRMS: calcd. for C_12_H_20_O_2_, *m*/*z* 197.1542 [M + H]^+^, found 197.1532. (Supplementary Materials, Figures S53–S58).

#### 3.2.3. 2-Iodo-4,4,7-trimethyl-9-oxabicyclo[4.3.0]nonan-8-one (**4a**) and 2-Iodo-4,4,6,7-tetramethyl-9-oxabicyclo-[4.3.0]nonan-8-one (**4b**)

According to the known procedure [28] we obtained 4.2 g, 13.6 mmol (yield 71%) of iodolactone **4a** as a mixture of diastereoisomers and 4.4 g, 13.7 mmol (yield 75%) and **4b** characterised by the data presented below:

**4a**: m.p. = 108–110 °C; ^1^H-NMR: 0.99 (s, 3H, CH_3_-9A), 1.01 (s, 3H, CH_3_-9B), 1.03 (s, 3H, CH_3_-10A), 1.09 (d, *J* = 7.2 Hz, 3H, CH_3_-11A), 1.10 (s, 3H, CH_3_-10B), 1.11 (d, *J* = 7.2 Hz, 3H, CH_3_-11B), 1.23 (s, 2H, CH_2_-5), 1.35 (d, *J* = 15.2 Hz, 1H, one of CH_2_-3A), 1.48 (s, 2H, CH_2_-5B), 1.75 (dd, *J* = 15.2 and 2.5 Hz, 1H, one of CH_2_-3A), 1.99 (dd, *J* = 14.8 and 4.6 Hz, 1H, H-6B), 2.02 (t, *J* = 13.7 Hz, 1H, one of CH_2_-3B), 2.10 (dd, *J* = 14.8 and 9.9 Hz, 1H, H-6A), 2.19–2.22 (m, 1H, one of CH_2_-3B), 2.42 (q, *J* = 7.2 Hz, 1H, H-7A), 2.92 (q, *J* = 7.1 Hz, 1H, H-7B), 4.23 (ddd, *J* = 17.5, 13.8 and 3.8 Hz, 1H, H-2B), 4.30–4.33 (m, 2H, H-2A, H-1B), 4.68 (d, *J* = 5.0 Hz, 1H, H-1A); ^13^C-NMR: 7.6 (C-11B), 7.6 (C-11A), 20.4 (C-2A), 25.7 (C-10A), 25.8 (C-10B), 26.0 (C-5A), 26.2 (C-2B), 30.0 (C-5B), 33.7 (C-9A), 34.0 (C-9B), 38.1 (C-6B), 40.5 (C-7B), 43.0 (C-6A), 43.1 (C-4A), 43.4 (C-4B), 44.2 (C-6A), 49.4 (C-3B), 49.6 (C-7A), 89.8 (C-1A), 91.1 (C-1B), 177.0 (C-8A), 177.5 (C-8B); IR (KBr, cm^−1^): 2955, 1779, 1464, 1164, 1024; ESIHRMS: calcd. for C_11_H_17_IO_2_, *m*/*z* 309.0346 [M + H]^+^, found 309.0352. (Supplementary Materials, Figures S13–S18).

**4b-A**: m.p. = 110–112 °C; ^1^H-NMR: 0.99 (s, 3H, CH_3_-9), 1.10 (d, *J* = 7.1 Hz, 3H, CH_3_-12), 1.11 (s, 3H, CH_3_-11), 1.12 (d, *J* = 7.1 Hz, 3H, CH_3_-12), 1.11–1.26 (m, 1H, one of CH_2_-5), 1.19–1.21 (m, 1H, one of CH_2_-5), 1.49 (s, 3H, CH_2_-12), 1.99 (dd, *J* = 14.7 and 4.5 Hz, 1H, one of CH_2_-3), 2.11 (dd, *J* = 14.7 and 9.7 Hz, 1H, one of CH_2_-3), 2.42 (q, *J* = 7.2 Hz, 1H, H-7), 4.32 (m, 1H, H-2), 4.33 (d, *J* = 5.0 Hz, 1H, H-1); ^13^C-NMR: 7.6 (C-11), 20.4 (C-2), 26.0 (C-12), 29.7 (C-4), 30.0 (C-10), 31.4 (C-6), 33.7 (C-9), 38.1 (C-5), 43.0 (C-3), 49.7 (C-7), 89.8 (C-1), 177.0 (C-8); IR (KBr, cm^−1^): 2956, 1763, 1465, 1189, 1009; ESIHRMS: calcd. for C_12_H_19_IO_2_Na, *m*/*z* 345.0327 [M + H]^+^, found 345.0322. (Supplementary Materials, Figures S59–S64).

**4b-B**: m.p. = 137–139 °C; ^1^H-NMR: 1.01 (s, 3H, CH_3_-9), 1.03 (s, 3H, CH_3_-10), 1.11 (s, 3H, CH_3_-11), 1.12 (d, *J* = 7.1 Hz, 3H, CH_3_-12), 1.36 (d, *J* = 15.2 Hz, 1H, one of CH_2_-5), 1.77 (dd, *J* = 15.2 and 2.5 Hz, 1H, one of CH_2_-5), 2.03 (t, *J* = 13.7 Hz, 1H, one of CH_2_-3), 2.21 (dd, *J* = 13.7 and 2.7 Hz, 1H, one of CH_2_-3), 2.93 (q, *J* = 7.1 Hz, 1H, H-7), 4.24 (ddd, *J* = 13.9, 10.2 and 3.8 Hz, 1H, H-2), 4.33 (d, *J* = 10.2 Hz, 1H, H-1); ^13^C-NMR: 7.6 (C-11), 25.6 (C-9), 25.78 (C-12), 26.2 (C-2), 34.0 (C-10), 34.1 (C-4), 40.6 (C-7), 43.4 (C-6), 44.3 (C-5), 49.4 (C-3), 91.1 (C-1), 177.5 (C-8); IR (KBr, cm^−1^): 2937, 1779, 1456, 1163, 1024; ESIHRMS: calcd. for C_12_H_19_IO_2_Na, *m*/*z* 345.0327 [M + H]^+^, found 345.0323. (Supplementary Materials, Figures S65–S70).

#### 3.2.4. 4,4,7-Trimethyl-9-oxabicyclo[4.3.0]non-2-en-8-one (**5a**) and 4,4,6,7-Trimethyl-9-oxabicyclo[4.3.0]non-2-en-8-one (**5b**)

Dehydrodehalogenation of this mixture, according to the known procedure [28], gave as a diastereoisomeric mixture 1.9 g, 10.6 mmol (yield 79%) of unsaturated lactone **5a** and 2.1 g, 10.8 mmol (yield 79%) of **5b** unsaturated lactone with the following physical and spectral data:

**5a**: m.p. = 74–76 °C; ^1^H-NMR: 1.02 (s, 6H, CH_3_-9A, CH_3_-9B), 1.07 (s, 6H, CH_3_-10A, CH_3_-10B), 1.08–1.14 (m, 1H, one of CH_2_-5B), 1.20 (d, *J* = 7.2 Hz, 3H, CH_3_-11B), 1.30–1.34 (m, 1H, one of CH_2_-5A), 1.37 (d, *J* = 7.8 Hz, 3H, CH_3_-11A), 1.45 (dd, *J* = 12.0 and 3.6 Hz, 1H, one of CH_2_-5B), 1.55 (dd, *J* = 13.8 and 4.2 Hz, 1H, one of CH_2_-5A), 2.28–2.32 (m, 1H, H-6A), 2.41 (q, *J* = 7.6 Hz, 1H, H-7A), 2.62–2.68 (m, 1H, H-6B), 2.96 (quintet, *J* = 7.3 Hz, 1H, H-7B), 4.62 (t, *J* = 4.7 Hz, 1H, H-1B), 4.81 (t, *J* = 4.9 Hz, 1H, H-1A), 5.78–5.81 (m, 2H, H-2A, H-2B), 5.90–5.93 (m, 2H, H-3A, H-3B); ^13^C-NMR: 9.2 (C-11B), 15.5 (C-11A), 26.8 (C-9B), 27.2 (C-9A), 30.1 (C-10A), 30.5 (C-10B), 31.7 (C-4A), 31.8 (C-4B), 33.5 (C-5B), 35.3 (C-5A), 38.4 (C-6B), 38.6 (C-6A), 40.0 (C-7B), 43.2 (C-7A), 73.1 (C-1B), 73.2 (C-1A), 119.7 (C-2B), 119.8 (C-2A), 144.8 (C-3A), 145.3 (C-3B), 178.8 (C-8A), 178.89 (C-8B); IR (KBr, cm^−1^): 2960, 1768, 1470, 1381, 1172, 967; ESIHRMS: calcd. for C_11_H_16_O_2_, *m*/*z* 181.1228 [M + H]^+^, found 181.1221. (Supplementary Materials, Figures S19–S24).

**5b-A**: n_D_ = 1.4810; ^1^H-NMR: 1.02 (s, 3H, CH_3_-9), 1.06 (s, 3H, CH_3_-10), 1.09 (s, 3H, CH_3_-12), 1.10 (d, *J* = 7.3 Hz, 3H, CH_3_-11), 1.47 (d, *J* = 14.9 Hz, 1H, one of CH_2_-5), 1.71 (dd, *J* = 14.9 and 2.5 Hz, 1H, one of CH_2_-5), 2.03 (t, *J* = 13.7 Hz, 1H, one of CH_2_-3), 2.21 (dd, *J* = 13.7 and 2.7 Hz, 1H, one of CH_2_-3), 2.78 (q, *J* = 7.3 Hz, 1H, H-7), 4.49 (s, 1H, H-1), 5.56 (dd, *J* = 10.2 and 2.5 Hz, 1H, H-2), 5.69 (d, *J* = 10.2 Hz, 1H, H-3); ^13^C-NMR: 7.6 (C-11), 22.9 (C-9), 29.4 (C-12), 31.5 (C-6), 33.0 (C-10), 40.4 (C-4), 41.1 (C-7), 42.3 (C-5), 81.0 (C-1), 120.6 (C-2), 140.6 (C-3), 178.9 (C-8); IR (KBr, cm^−1^): 2962, 1775, 1456, 1378, 1169, 977, ESIHRMS: calcd. for C_12_H_18_O_2_, *m*/*z* 195.1385 [M + H]^+^, found 195.1391. (Supplementary Materials, Figures S71–S76).

**5b-B**: n_D_ = 1.4810; ^1^H-NMR: 1.04 (s, 3H, CH_3_-9), 1.12 (s, 3H, CH_3_-10), 1.13 (d, *J* = 7.2 Hz, 3H, CH_3_-11), 1.24 (s, 3H, CH_3_-12), 1.28 (d, *J* = 14.0 Hz, 1H, one of CH_2_-5), 1.36 (dd, *J* = 14.0 and 2.5 Hz, 1H, one of CH_2_-5) 2.44 (q, *J* = 7.2 Hz, 1H, H-7), 4.28 (d, *J* = 5.2 Hz, 1H, H-1), 5.77 (dd, *J* = 10.0 and 5.2 Hz, 1H, H-2), 5.89 (d, *J* = 10.0 Hz, 1H, H-3); ^13^C-NMR: 7.2 (C-11), 23.0 (C-12), 28.4 (C-10), 31.8 (C-6), 33.1 (C-9), 38.3 (C-5), 40.2 (C-4), 49.9 (C-7), 78.3 (C-1), 117.6 (C-2), 144.7 (C-3), 178.5 (C-8); IR (KBr, cm^−1^): 2961, 1771, 1457, 1379, 1169, 976; ESIHRMS: calcd. for C_12_H_18_O_2_, *m*/*z* 195.1385 [M + H]^+^, found 195.1322. (Supplementary Materials, Figures S77–S82).

### 3.3. Biotransformations

#### 3.3.1. Microorganisms

The fungal and yeast strains which were used for biotransformation came from the collection of the Department of Chemistry, Wrocław University of Environmental and Life Sciences (Wrocław, Poland). The following strains were used: *Fusarium culmorum* AM10, *Fusarium avenaceum* AM11, *Fusarium oxysporum* AM13, *Fusarium tricinctum* AM16, *Fusarium semitectum* AM20, *Penicilium vermiculatum* AM30, *Penicillium albidum* AM79, *Penicillium camembertii* AM83, *Penicilium chermesinum* AM113, *Absidia corerulea* AM93, *Absidia glauca* AM254, *Absidia glauca* AM177, *Absidia cylindrospora* AM336, *Syncephalastrum racemosum* AM105. All of these strains were cultivated on Sabouraud’s agar consisting of aminobac (0.5%), peptone (0.5%), glucose (4%) and agar (1.5%) dissolved in distilled water at 28 °C and stored in a refrigerator at 4 °C.

#### 3.3.2. Screening Procedure

Each strain of the fungus was cultured in two 300 mL Erlenmeyer flasks containing standard medium (3 g of glucose, 1 g of peptone, dissolved in 100 mL of distilled water). After three days 10 mg of substrate dissolved in 1 mL of acetone was added to each flask with the grown culture. Incubation of the shaken cultures with substrate was continued. After 7 and 14 days of incubation, the medium were extracted with dichloromethane (15 mL) and analyzed by GC (DB-17) column.

#### 3.3.3. Preparative Biotransformation

Preparative biotransformation was carried out in ten 300 mL Erlenmeyer flasks containing cultures of 3-day fungal strains (prepared in a similar manner as described in the screening procedure). Substrate (100 mg) was dissolved in 10 mL of acetone and added to ten bottles. After 14 days the reaction mixture was extracted with dichloromethane (3 × 40 mL). The combined organic fractions were dried (MgSO_4_) and evaporated under reduced pressure. The pure product was purified by column chromatography (silica gel, hexane−acetone 3:1). As a result of these reactions two products were obtained.

*1-Hydroxy-4,4,7-trimethyl-9-oxabicyclo[4.3.0]non-2-en-8-one* (**6a**) was characterized by the following physical and spectral properties: m.p. = 77–78 °C; ^1^H-NMR: 1.14 (d, *J* = 7.2 Hz, 3H, CH_3_-11A), 1.19 (s, 3H, CH_3_-9A), 1.20 (s, 3H, CH_3_-9B), 1.23 (s, 3H, CH_3_-10B), 1.24 (s, 3H, CH_3_-10A), 1.25 (d, *J* = 7.2 Hz, 3H, CH_3_-11B), 1.78–1.79 (m, 2H, CH_2_-5A), 1.80–1.81 (m, 1H, CH_2_-5B), 1.95–2.00 (m, 1H, one of CH_2_-5B), 2.91–2.95 (m, 1H, one of H-6B), 3.02–3.04 (m, 1H, H-7B), 3.06–3.09 (m, 1H, H-6A), 3.16–3.18 (m, 1H, H-7A), 5.86 (d, *J* = 10.0 Hz, 1H, H-3A), 5.87 (d, *J* = 10.0 Hz, 1H, H-3B), 6.63–6.66 (m, 2H, H-2A, H-2B); ^13^C-NMR: 12.0 (C-11A), 12.2 (C-11B), 24.3 (C-10A), 24.8 (C-10B), 30.1 (C-9B), 30.2 (C-9A), 33.2 (C-4A), 33.2 (C-4B), 37.4 (C-7B), 37.4 (C-7A), 38.2 (C-5B), 38.3 (C-5A), 44.3 (C-6A), 44.7 (C-6B), 125.9 (C-3A), 126.1 (C-3B), 158.6 (C-2B), 158.7 (C-2A), 179.4 (C-1B), 181.3 (C-1A), 198.6 (C-8A), 198.7 (C-8B); ESIHRMS: calcd. for C_11_H_16_O_3_, *m*/*z* 197.1178 [M + H]^+^, found 197.1173. (Supplementary Materials, Figures S25–S29).

*6-Hydroxy-4,4,7-trimethyl-9-oxabicyclo[4.3.0]non-2-en-8-one* (**7a**) was characterized by the following physical and spectral properties: m.p. = 76–77 °C; ^1^H-NMR: 1.04 (s, 3H, CH_3_-9), 1.09 (s, 3H, CH_3_-10), 1.43 (d, *J* = 5.0 Hz, 3H, CH_3_-11), 1.45 (dd, *J* = 14.9 and 4.5 Hz, 2H, CH_2_-5), 2.50 (dt, *J* = 14.4 and 4.8 Hz, 1H, H-7), 5.03 (dd, *J* = 8.8 and 4.4 Hz, 1H, H-1), 5.83 (dd, *J* = 9.9 and 4.4 Hz, 1H, H-2), 5.94 (d, *J* = 9.9 Hz, 1H, H-3); ^13^C-NMR: 19.1 (C-11), 26.7 (C-9), 30.3 (C-10), 32.1 (C-4), 34.0 (C-5), 42.0 (C-7), 73.0 (C-1), 77.7 (C-6), 119.6 (C-2), 145.1 (C-3), 177.1 (C-8); ESIHRMS: calcd. for C_11_H_16_O_3_, *m*/*z* 197.1178 [M + H]^+^, found 197.1176. (Supplementary Materials, Figures S30–S34).

*10-Hydroxy-4,4,7-trimethyl-9-oxabicyclo[4.3.0]non-2-en-8-one* (**8a**) was characterized by the following physical and spectral properties: colourless oil; ^1^H-NMR: 1.06 (s, 3H, CH_3_-9), 1.20–1.22 (m, 1H, one of CH_2_-5), 1.35 (d, *J* = 7.6 Hz, 3H, CH_3_-11), 1.88 (dd, *J* = 13.6 and 4.9 Hz, 1H, one of CH_2_-5), 2.40–2.44 (m, 1H, H-7), 2.45–2.49 (m, 1H, H-6), 3.40 (d, *J* = 2.4 Hz, 2H, CH_2_-10), 4.84 (dd, *J* = 5.0 and 4.8 Hz, 1H, H-1), 5.82 (d, *J* = 10.0 Hz, 1H, H-3), 5.94 (dd, *J* = 10.0 and 3.9 Hz, 1H, H-2); ^13^C-NMR: 15.3 (C-11), 24.8 (C-9), 33.5 (C-5), 37.3 (C-4), 38.6 (C-7), 42.9 (C-6), 69.2 (C-10), 73.12 (C-1), 123.01 (C-2), 139. 9 (C-3), 179.8 (C-8); IR (KBr, cm^−1^): 3468, 2964, 1785, 1456, 1170, 1024; ESIHRMS: calcd. for C_11_H_16_O_3_, *m*/*z* 197.1178 [M + H]^+^, found 197.1181. (Supplementary Materials, Figures S35–S40).

*2,3-Epoxy-4,4,7-trimethyl-9-oxabicyclo[4.3.0]nonan-8-one* (**9a**) was characterized by the following physical and spectral properties: colourless oil; ^1^H-NMR: 0.85 (dd, *J* = 13.7 and 13.7 Hz, 1H, one of CH_2_-5B), 1.05 (s, 3H, CH_3_-9B), 1.08 (s, 3H, CH_3_-9A), 1.15 (d, *J* = 7.1 Hz, 3H, CH_3_-11B), 1.17 (s, 6H, CH_3_-10A, CH_3_-10B), 1.18–1.19 (m, 1H, one of CH_2_-5B), 1.20–1.21 (m, 2H, CH_2_-5A), 1.32 (d, *J* = 7.5 Hz, 3H, CH_3_-11A), 12.15–2.20 (m, 1H, H-6A), 2.41–2.47 (m, 2H, H-6B and H-7A), 2.84 (quintet, *J* = 7.1 Hz, 1H, H-7B), 3.02 (d, *J* = 3.2 Hz, 1H, H-3A), 3.04 (d, *J* = 3.3 Hz, 1H, H-3B), 3.46 (d, *J* = 3.2 Hz, 1H, H-2A), 3.52 (dd, *J* = 3.3 and 1.3 Hz, 1H, H-2B), 4.67 (d, *J* = 4.2 Hz, 1H, H-1B), 4.82 (d, *J* = 6.1 Hz, 1H, H-1A); ^13^C-NMR: 9.2 (C-11B), 14.4 (C-11A), 23.4 (C-9B), 25.4 (C-9A), 28.3 (C-4B), 28.8 (C-10A), 28. 8 (C-4A), 30.0 (C-10B), 33.2 (C-5B), 34.4 (C-6B), 35.7 (C-5A), 27.6 (C-6A), 39.9 (C-7B), 41.4 (C-7A), 52.3 (C-2B), 52.4 (C-2A), 61.1 (C-3A), 61.2 (C-3B), 73.5 (C-A), 74.1 (C-1B), 178.2 (C-8A), 178.2 (C-8B); IR (KBr, cm^−1^): 2952, 1785, 1476, 1457, 1160, 1024; ESIHRMS: calcd. for C_11_H_16_O_3_, *m*/*z* 197.1178 [M + H]^+^, found 197.1179. (Supplementary Materials, Figures S41–S46)

*1-Hydroxy-4,4,6,7-tetramethyl-9-oxabicyclo[4.3.0]non-2-en-8-one* (**6b**) was characterized by the following physical and spectral properties: colourless oil; ^1^H-NMR: 1.13 (d, *J* = 7.0 Hz, 3H, CH_3_-11A), 1.20 (s, 3H, CH_3_-9A), 1.21 (s, 3H, CH_3_-9B), 1.23 (d, *J* = 7.5 Hz, 3H, CH_3_-11B), 1.28 (s, 6H, CH_3_-10B, CH_3_-12B), 1.30 (s, 3H, CH_3_-10A), 1.34 (s, 3H, CH_3_-12A), 1.65 (d, *J* = 14.1 Hz, 1H, one of CH_2_-5B), 1.86–1.89 (m, 1H, CH_2_-5A), 2.02–2.05 (m, 1H, one of CH_2_-5A), 2.19–2.22 (m, 1H, one of CH_2_-5B), 3.01–3.02 (m, 1H, H-7B), 3.12–3.13 (m, 1H, H-7A), 5.86 (d, *J* = 9.9 Hz, 1H, H-3A), 5.89 (d, *J* = 10.1 Hz, 1H, H-3A), 6.60–6.62 (m, 2H, H-2A, H-2B); ^13^C-NMR: 10.9 (C-11B), 12.5 (C-11A), 24.5 (C-12A), 24.3 (C-10A), 28.8 (C-10B), 29.2 (C-12B), 32.2 (C-9B), 32.4 (C-9A), 40.8 (C-5A), 42.1 (C-5B), 44.7 (C-7B), 45.0 (C-7A), 44.7 (C-4A), 45.0 (C-4B), 44.4 (C-6A), 44.7 (C-6B), 124.4 (C-3A), 124.7 (C-3B), 157.3 (C-2B), 157.3 (C-2A), 178.1 (C-1B), 178.2 (C-1A), 178.2 (C-8A), 179.6 (C-8B); IR (KBr, cm^−1^): 3480, 2962, 1751, 1675, 1461, 1382, 1242, 1154, 1065; ESIHRMS: calcd. for C_11_H_18_O_3_, *m*/*z* 211.1334 [M + H]^+^, found 211.1330. (Supplementary Materials, Figures S83–S88).

*7-Hydroxy-4,4,6,7-tetramethyl-9-oxabicyclo[4.3.0]non-2-en-8-one* (**7b**) was characterized by the following physical and spectral properties: colourless oil; ^1^H-NMR: 1.05 (s, 3H, CH_3_-9), 1.15–1.16 (m, 1H, one of CH_2_-5), 1.17 (s, 3H, CH_3_-10), 1.19 (s, 3H, CH_3_-11), 1.32 (s, 3H, CH_3_-12), 1.33–1.36 (m, 1H, one of CH_2_-5), 4.67 (d, *J* = 5.3 Hz, 1H, H-1), 5.80 (dd, *J* = 10.0 and 5.3 Hz, 1H, H-2), 5.90 (d, *J* = 10.0 Hz, 1H, H-3); ^13^C-NMR: 14.7 (C-11), 16.9 (C-12), 17.0 (C-4), 28.8 (C-9), 32.3 (C-6), 33.2 (C-10), 40.0 (C-5), 43.0 (C-7), 77.3 (C-1), 117.4 (C-2), 144.4 (C-3), 177.7 (C-8B); IR (KBr, cm^−1^): 2464, 2927, 1757, 1464, 1382, 1141, 974; ESIHRMS: calcd. for C_11_H_18_O_3_, *m*/*z* 211.1334 [M + H]^+^, found 211.1331, calcd. for C_11_H_18_O_3_Na, *m*/*z* 233.1154 [M + H]^+^, found 233.1166. (Supplementary Materials, Figures S89–S94).

*10-Hydroxy-4,4,6,7-tetramethyl-9-oxabicyclo[4.3.0]non-2-en-8-one* (**8b**) was characterized by the following physical and spectral properties: colourless oil; ^1^H-NMR: 1.06 (s, 3H, CH_3_-9), 1.09 (s, 3H, CH_3_-12), 1.12 (d, *J* = 7.3 Hz, 3H, CH_3_-11), 1.57 (d, *J* = 15.0 Hz, 1H, one of CH_2_-5), 1.80 (d, *J* = 15.0 Hz, 1H, one of CH_2_-5), 2.80 (q, *J* = 7.3 Hz, 1H, H-7), 3.29 (d, *J* = 10.5 Hz, 1H, one of CH_2_-10), 3.38 (d, *J* = 10.5 Hz, 1H, one of CH_2_-10), 4.53 (s, 1H, H-1), 5.64 (dd, *J* = 10.4 and 5.3 Hz, 1H, H-2), 5.79 (d, *J* = 10.4 Hz, 1H, H-3); ^13^C-NMR: 7.2 (C-11), 23.2 (C-9), 24.1 (C-12), 36.03 (C-5), 37.2 (C-4), 40.4 (C-6), 40.8 (C-7), 72.0 (C-10), 80.9 (C-1), 125.2 (C-3), 135.8 (C-2), 178.7 (C-8B); IR (KBr, cm^−1^): 3409, 2933, 1757, 1451, 1207, 1175, 1041; ESIHRMS: calcd. for C_11_H_18_O_3_, *m*/*z* 211.1334 [M + H]^+^, found 211.1331, calcd. for C_11_H_18_O_3_Na, *m*/*z* 233.1154 [M + H]^+^, found 233.1019. (Supplementary Materials, Figures S95–S100).

*2,3-Epoxy-4,4,6,7-tetramethyl-9-oxabicyclo[4.3.0]nonan-8-one* (**9b**) was characterized by the following physical and spectral properties: colourless oil; ^1^H-NMR: 0.98 (s, 6H, CH_3_-9A, CH_3_-9B), 1.08 (d, *J* = 7.2 Hz, 3H, CH_3_-11A), 1.10 (d, *J* = 7.2 Hz, 3H, CH_3_-11B), 1.12 (s, 3H, CH_3_-12A), 1.16 (s, 3H, CH_3_-12B), 1.21 (s, 6H, CH_3_-10A, CH_3_-10B), 1.26–1.30 (dd, *J* = 15.1 and 9.9 Hz, 2H, CH_2_-5A), 1.37 (d, *J* = 15.1 Hz, 2H, CH_2_-5B), 2.37 (q, *J* = 7.2 Hz, 1H, H-7A), 2.80 (q, *J* = 7.2 Hz, 1H, H-7B), 2.98 (d, *J* = 3.3 Hz, 1H, H-3B), 3.02 (d, *J* = 3.5 Hz, 1H, H-3A), 3.33 (d, *J* = 3.3 Hz, 1H, H-2B), 3.60 (dd, *J* = 3.5 and 3.3 Hz, 1H, H-2A), 4.29 (s, 1H, H-1B), 4.42 (m, 1H, H-1A); ^13^C-NMR: 6.9 (C-11A), 7.4 (C-11B), 23.5 (C-9A), 23.6 (C-9B), 26.5 (C-5B), 26.6 (C-5A), 29.5 (C-6B), 30.1 (C-12B), 31.1 (C-12A), 33.1 (C-6A), 38.0 (C-10A), 38.2 (C-10B), 39.6 (C-4B), 39.9 (C-7B), 41.1 (C-4A), 49.9 (C-7A), 53.4 (C-2A), 53.8 (C-2B), 60.7 (C-3B), 60.8 (C-3A), 78.3 (C-1A), 79.3 (C-1B), 178.2 (C-8A), 178.2 (C-8B); IR (KBr, cm^−1^): 2963, 1707, 1673, 1459, 1383, 1241, 1096; ESIHRMS: calcd. for C_11_H_18_O_3_, *m*/*z* 211.1334 [M + H]^+^, found 211.1331, calcd. for C_11_H_18_O_3_Na, *m*/*z* 233.1154 [M + H]^+^, found 233.1019. (Supplementary Materials, Figures S101–S106).

## 4. Conclusions

Two new bicyclic unsaturated lactones **5a** and **5b**, obtained as a pairs of diastereoisomers **A** and **B** after a four-step synthesis, were subjected to a screening biotransformation using fourteen fungal strains. Some of them were able to introduce a hydroxy group or an oxirane ring into the molecules of lactones **5a** and **5b**. During these biotransformations eight new compounds were obtained: six hydroxylactones and two epoxylactones. In both substrates the hydroxy group was introduced onto a tertiary carbon (allylic position) or onto a primary carbon. Unexpectedly, hydroxylation of other non-allylic tertiary carbons (C-6 for **5a** and C-7 for **5b**) was also observed. The products with hydroxy groups in an allylic position and epoxylactones were created as pairs of diastereoisomers **A** and **B**. Four other products were formed as single isomers (only **B**). Among these compounds, lactones **7a** were obtained with a predominance of the (−)-isomer, while in the case of lactones **7b**, **8a** and **8b**, the (+)-isomer was formed preferentially.

## Figures and Tables

**Table 1:** Positive results of the screening biotransformation of lactone **5a** after 7 and 14 days of incubation (in % according to GC).

Strain	Days	Lactone 5a (A + B)	Lactone 6a (A + B)	Lactone 7a (B)	Lactone 8a (B)	Lactone 9a (A + B)
*S. racemosum* AM105	7	9.7 + 70.2	0 + 9.3	10.8	-	-
	14	0 + 36.5	10.2 + 25.0	24.5	-	-
*A. cylindrospora* AM336	7	11.1 + 70.3	-	15.0	-	0 + 3.6
	14	3.7 + 35.0	-	33.6	-	9.3 + 18.3
*A. glauca* AM177	7	12.2 + 70.8	0 + 12.3	4.6	-	-
	14	9.2 + 54.9	5.6 + 20.4	9.9	-	-
*A. glauca* AM254	7	11.3 + 57.9	4.8 + 13.9	12.0	-	-
	14	9.6 + 57.8	3.5 + 13.3	15.8	-	-
*P. vermiculatum* AM30	7	-	-	-	82.6	-
	14	-	-	-	87.1	-

**Table 2:** Positive results of the screening biotransformation of lactone **5b** after 7 and 14 days of incubation (in % according to GC).

Strain	Days	Lactone 5b (A + B)	Lactone 6b (A + B)	Lactone 7b (B)	Lactone 8b (B)	Lactone 9b (A + B)
*S. racemosum* AM105	7	22.3 + 48.0	0 + 7.9	21.8	-	-
	14	11.1 + 22.6	8.6 + 19.7	38.0	-	-
*A. cylindrospora* AM336	7	24.4 + 54.6	-	21.0	-	-
	14	25.9 + 33.2	-	50.9	-	-
*A. glauca* AM177	7	21.7 + 44.6	8.4 + 16.3	9.0	-	-
	14	18.0 + 35.5	8.1 + 22.6	15.8	-	-
*A. glauca* AM254	7	36.8 + 45.8	4.9 + 4.6	7.9	-	-
	14	24.7 + 31.4	9.9 + 9.4	14.5	-	-
*P. vermiculatum* AM30	7	10.9 + 40.8	-	-	16.2	25.9 + 6.2
	14	0 + 8.3	-	-	36.7	43.8 + 10.9

**Table 3:** Results of the preparative-scale biotransformation of lactone **5a** after 14 days of incubation (in % according to GC).

Strain	Lactone 5a (A + B)	Lactone 6a (A + B)	Lactone 7a (B)	Lactone 8a (B)	Lactone 9a (A + B)
*S. racemosum* AM105	0 + 31.2	13.0 + 37.5	18.4	-	-
*A. cylindrospora* AM336	14.7 + 30.1	-	27.5	-	16.2 + 11.4
*P. vermiculatum* AM30	0 + 16.2	-	40.4	43.4	-

**Table 4:** Results of the preparative-scale biotransformation of lactone **5b** after 14 days of incubation (in % according to GC).

Strain	Lactone 5b (A + B)	Lactone 6b (A + B)	Lactone 7b (B)	Lactone 8b (B)	Lactone 9b (A + B)
*S. racemosum* AM105	8.4 + 14.5	9.1 + 33.4	34.6	-	-
*A. cylindrospora* AM336	20.4 + 36.5	-	43.1	-	-
*P. vermiculatum* AM30	0 + 6.1	-	-	48.7	23.6 + 21.6

**Table 5:** Yield of products obtained from lactone **5a**.

Strain	Lactone 6a (A + B) mg/%	Lactone 7a (B) mg/%	Lactone 8a (B) mg/%	Lactone 9a (A + B) mg/%
*S. racemosum* AM105	21.7/19.9	13.7/12.6	-	-
*A. cylindrospora* AM336	-	15.8/14.5	-	8.1/7.4
*P. vermiculatum* AM30	-	29.2/26.8	35.7/32.8	-

**Table 6:** Yield of products obtained from lactone **5b**.

Strain	Lactone 6b (A + B) mg/%	Lactone 7b (B) mg/%	Lactone 8b (B) mg/%	Lactone 9b (A + B) mg/%
*S. racemosum* AM105	19.6/18.1	11.4/10.5	-	-
*A. cylindrospora* AM336	-	19.8/18.3	-	-
*P. vermiculatum* AM30	-	-	19.0/17.6	13.4/12.4

**Table 7:** The values of enantiospecificity and optical purity of hydroxylactones **7a-B**, **7b-B**, **8a-B** and **8b-B**.

Strain	Lactone	ee (%)	${\left[\alpha \right]}_{20}^{D}$
*S. racemosum* AM105	**7a-B**	47.9	−23.8 (*c* = 0.51, CH_3_Cl)
	**7b-B**	50.8	+10.1 (*c* = 0.60, CH_3_Cl)
*A. cylindrospora* AM336	**7a-B**	72.1	−27.4 (*c* = 0.70, CH_3_Cl)
	**7b-B**	57.2	+15.3 (*c* = 0.45, CH_3_Cl)
*P. vermiculatum* AM30	**7a-B**	36.8	−18.0 (*c* = 0.58, CH_3_Cl)
	**8a-B**	34.7	+17.2 (*c* = 0.67, CH_3_Cl)
	**8b-B**	38.5	+21.1 (*c* = 0.35, CH_3_Cl)

## Supplementary Materials

Supplementary materials can be accessed at: http://www.mdpi.com/1420-3049/22/1/147/s1.
