# Supplementary material for: Synthesis and Biotransformation of Bicyclic Unsaturated Lactones with Three or Four Methyl Groups
Source: Molecules. 2017 Jan 17;22(1):147. doi: 10.3390/molecules22010147 (PMC6155629; doi:10.3390/molecules22010147)
Supplement: Supplementary file 1 [file molecules-22-00147-s001.pdf]

Supplementary Materials: Synthesis and Biotransformation of Bicyclic Unsaturated Lactones with Three or Four Methyl Groups

Katarzyna Wińska, Małgorzata Grabarczyk, Wanda Mączka, Adrianna Kondas, Gabriela Maciejewska, Radosław Bonikowski and Mirosław Anioł

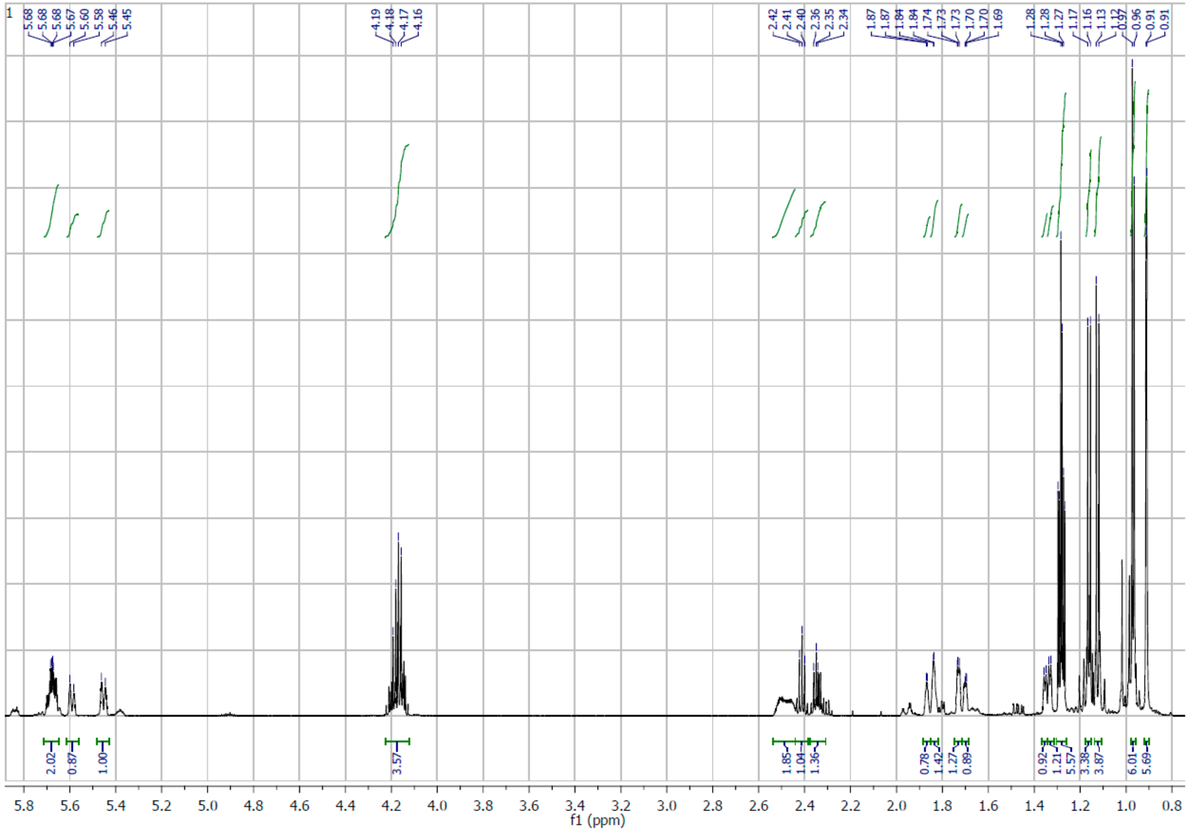

Figure S1. ^{1}H-NMR (600 MHz, $CDCl_3$) spectrum of ester **2a**.

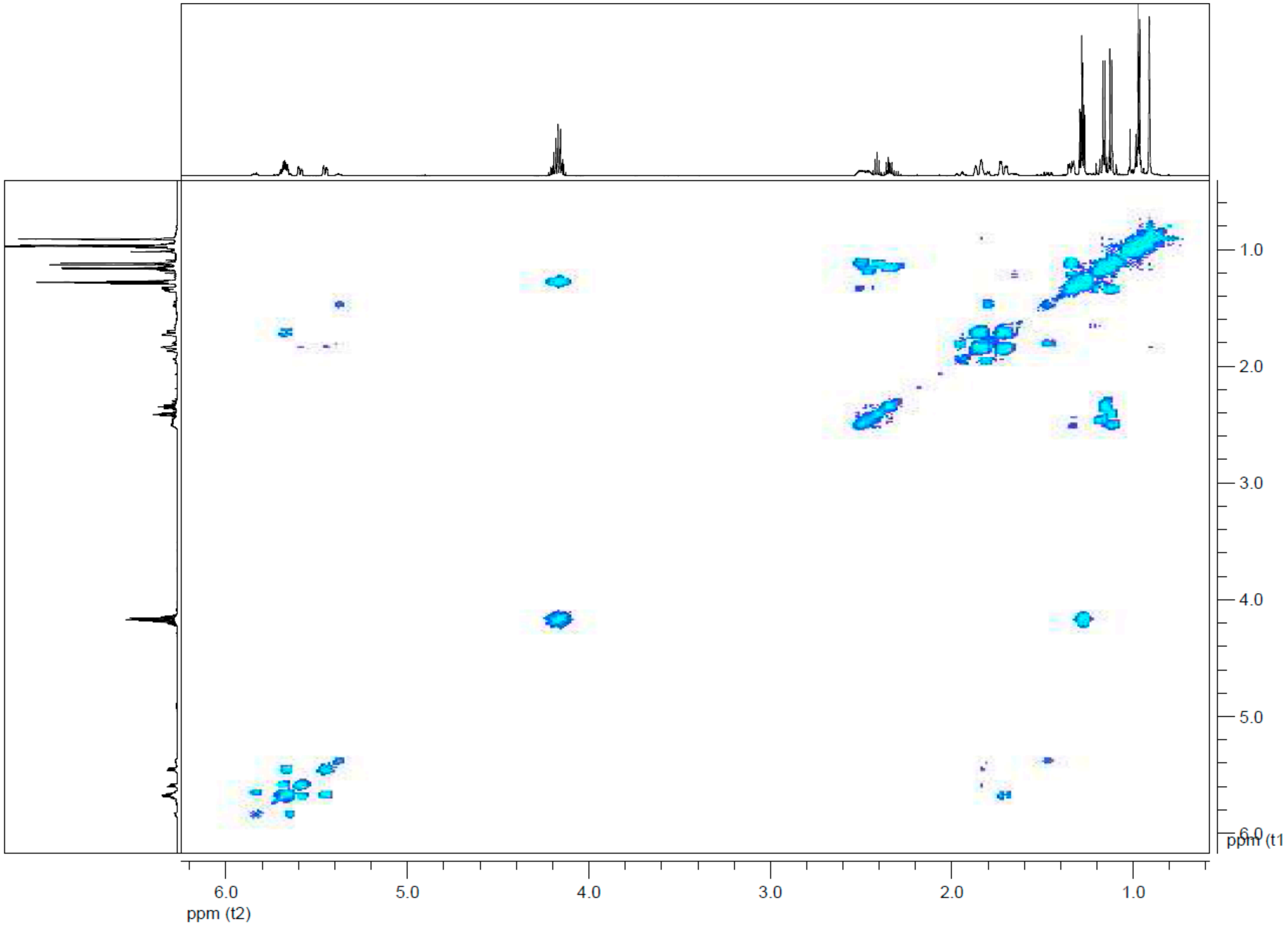

Figure S2. COSY (151 MHz, $CDCl_3$) spectrum of ester **2a**.

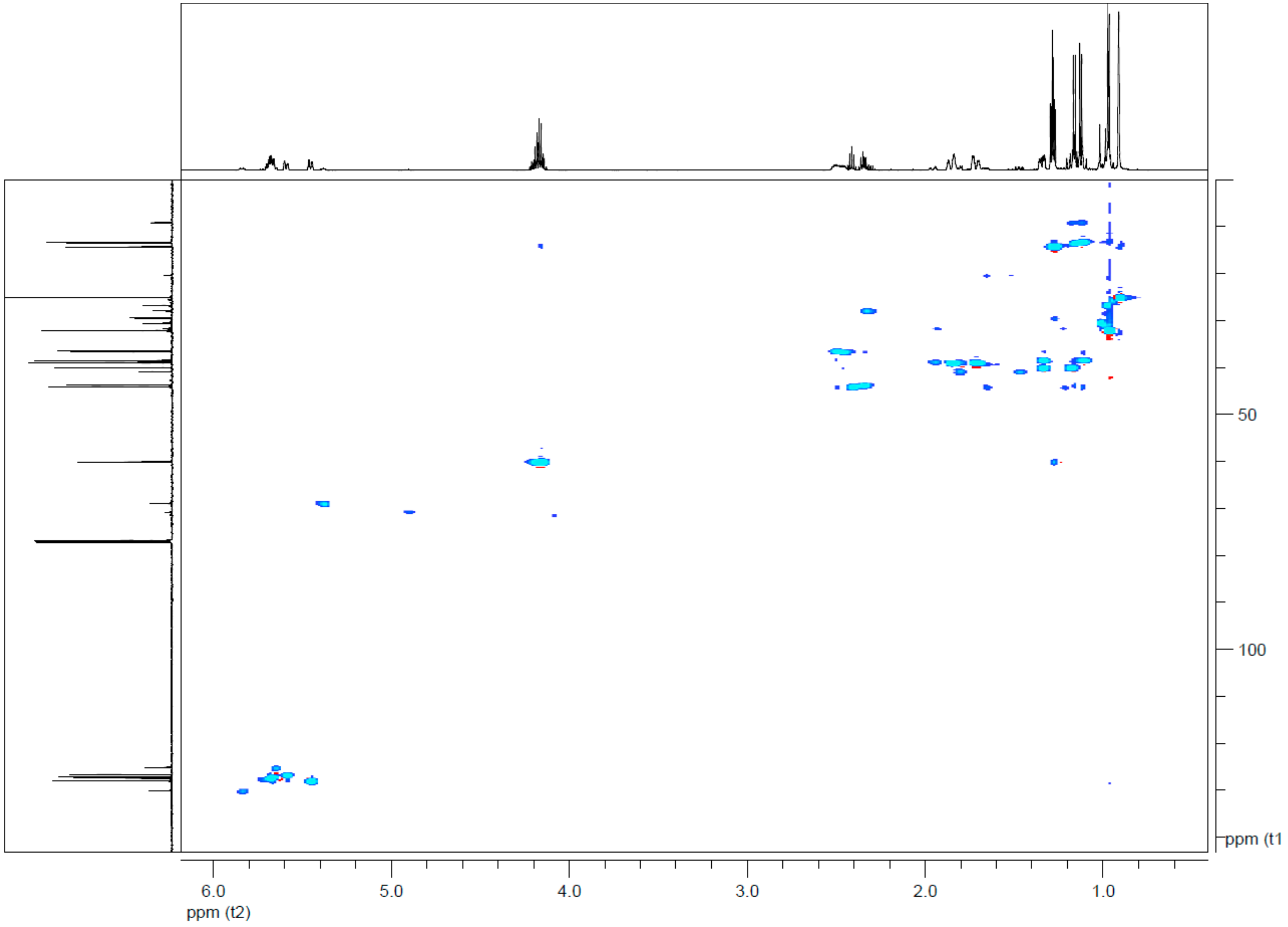

Figure S3. HMQC (151 MHz, $CDCl_3$) spectrum of ester **2a**.

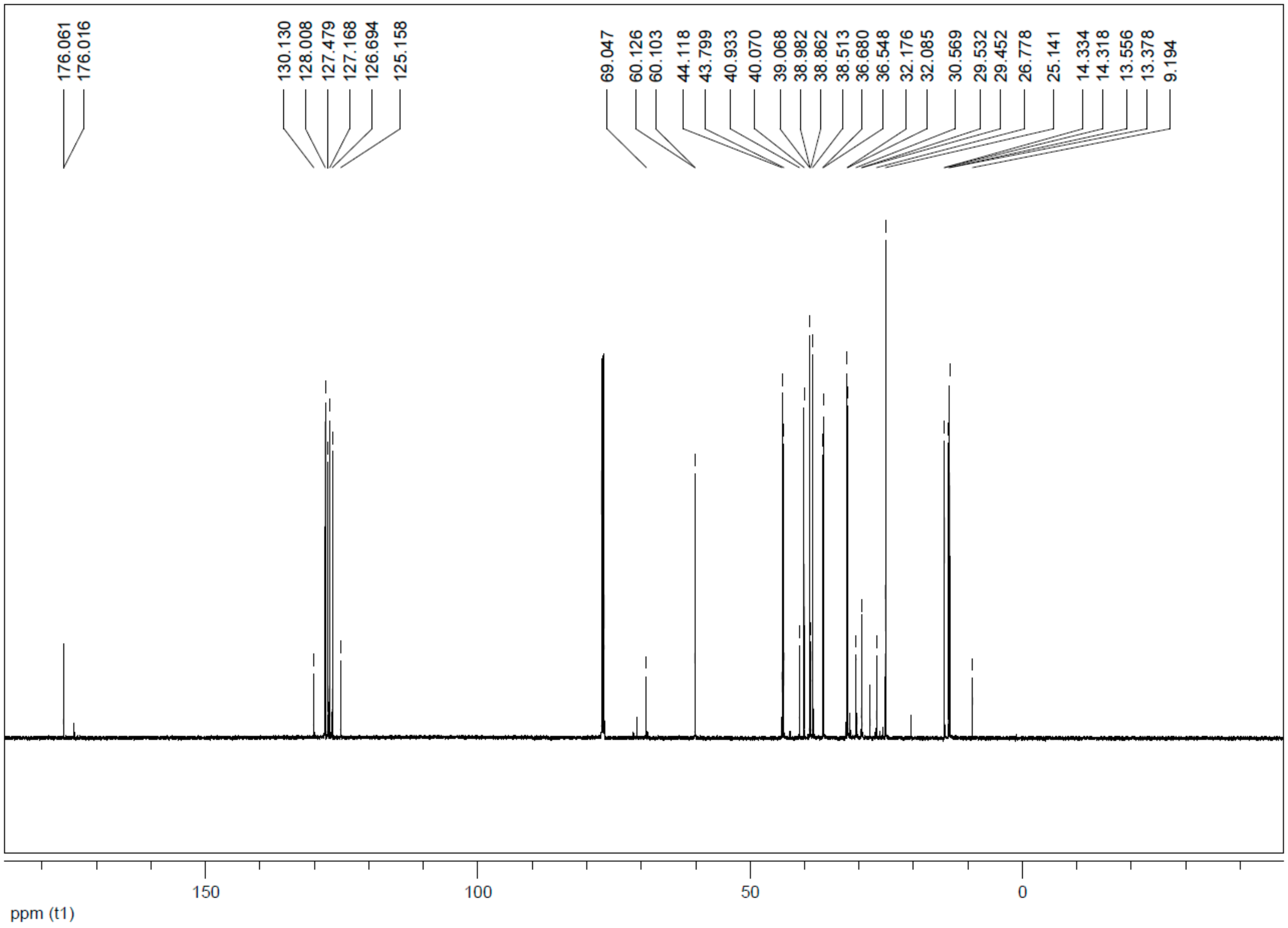

Figure S4. ^{13}C-NMR (151 MHz, $CDCl_3$) spectrum of ester **2a**.

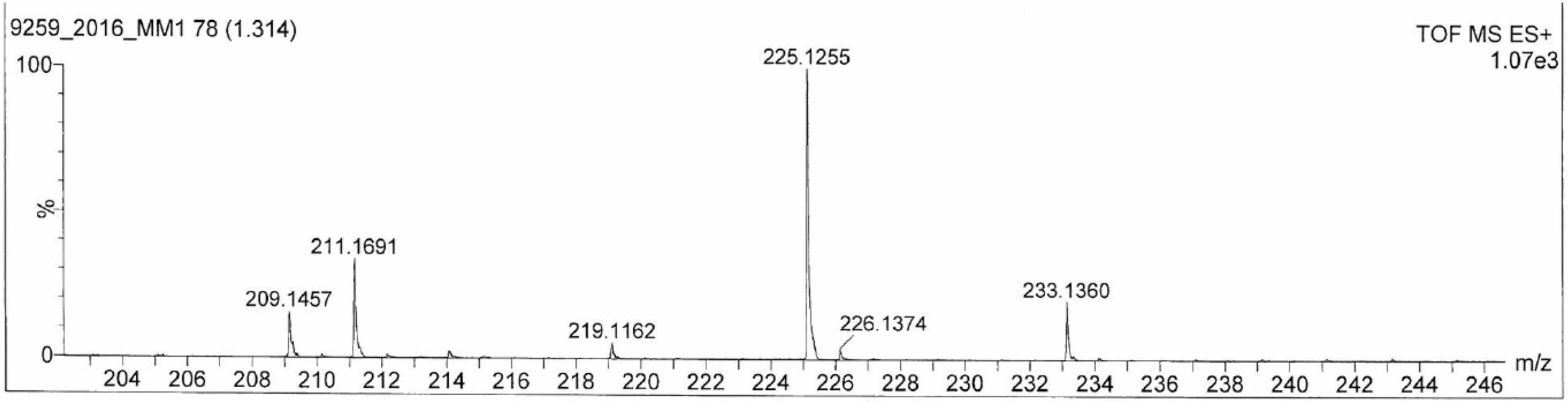

Figure S5. HRMS spectrum of ester **2a**.

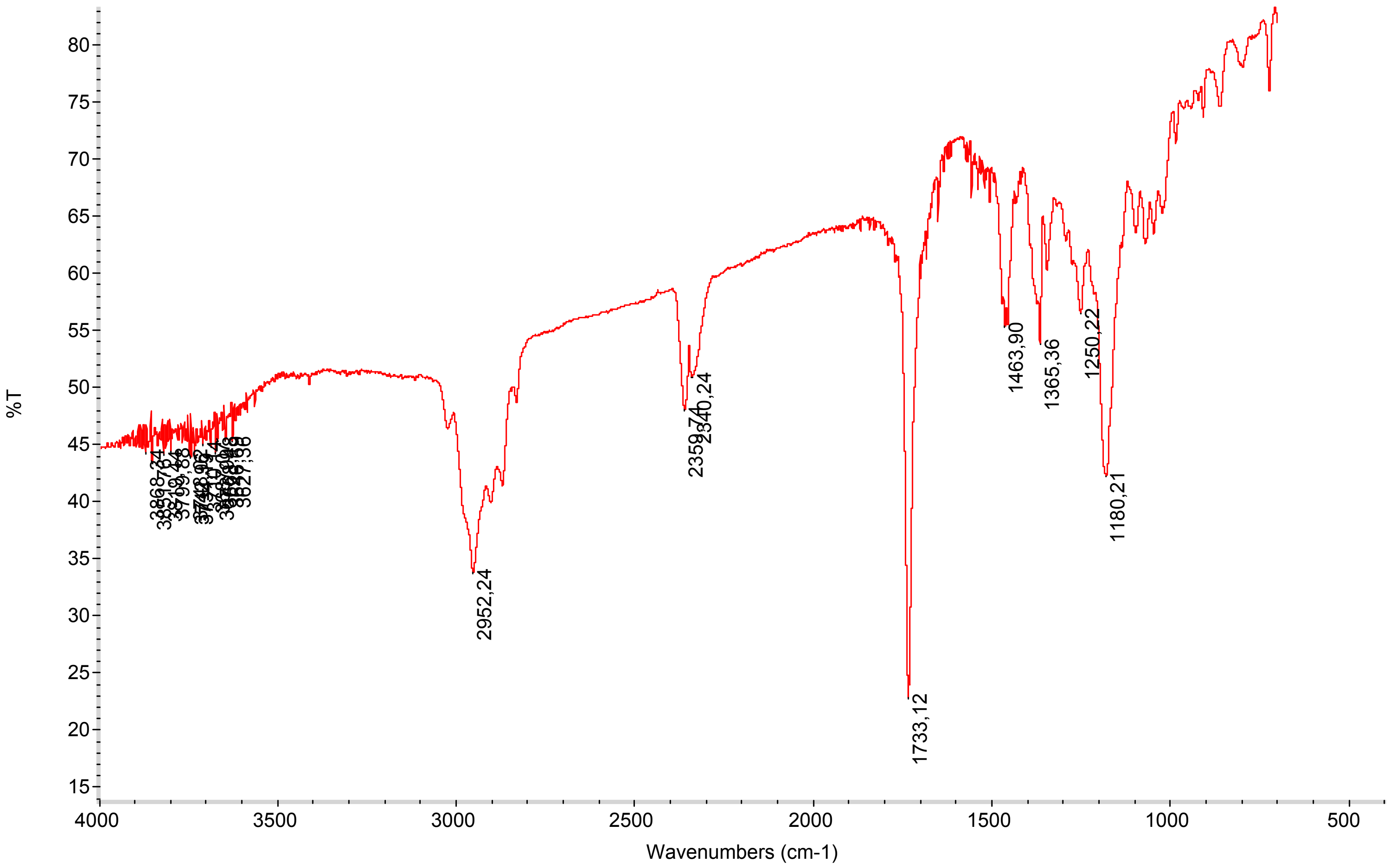

Figure S6. IR spectrum of ester **2a**.

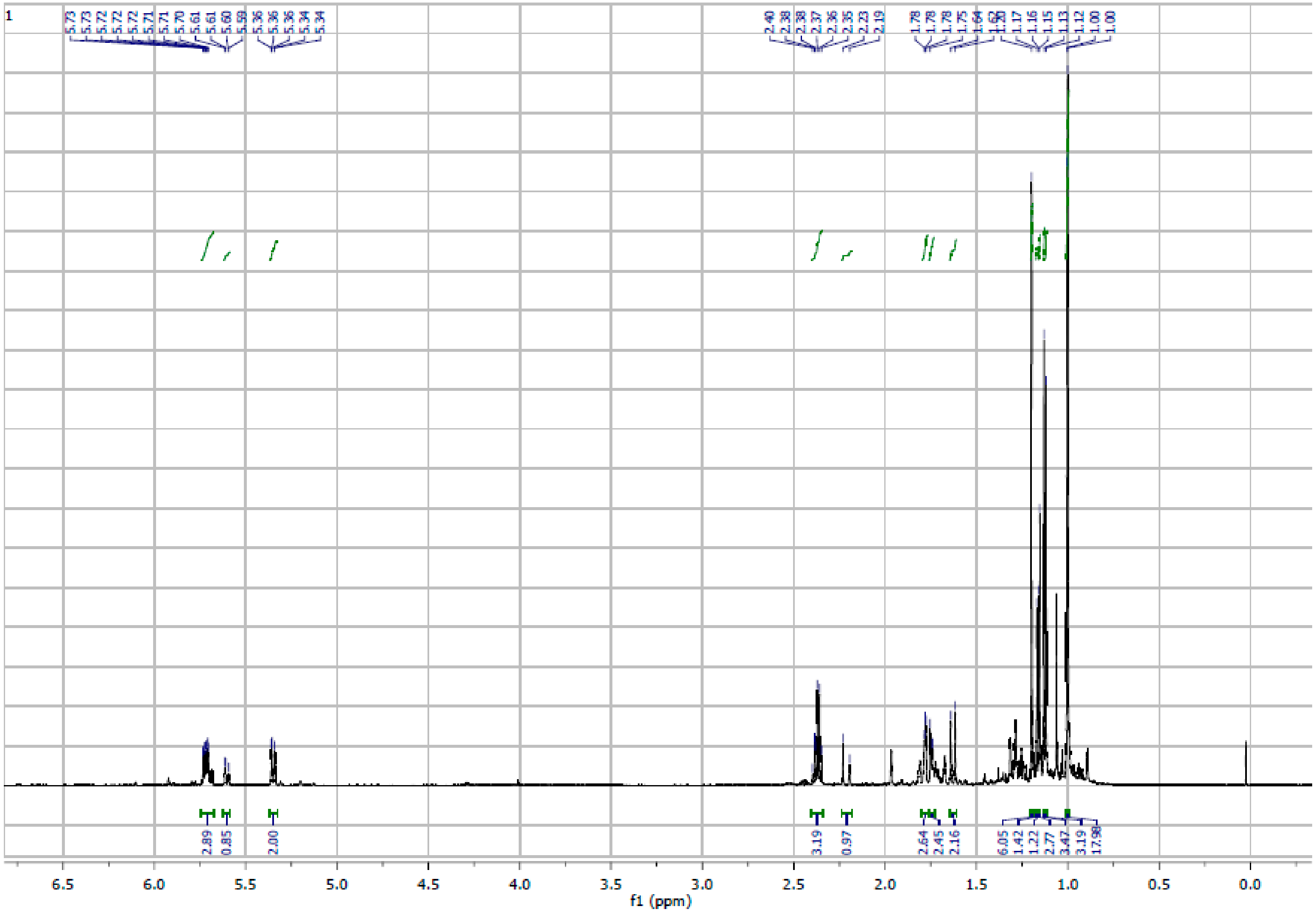

Figure S7. ^{1}H-NMR (600 MHz, $CDCl_3$) spectrum of acid **3a**.

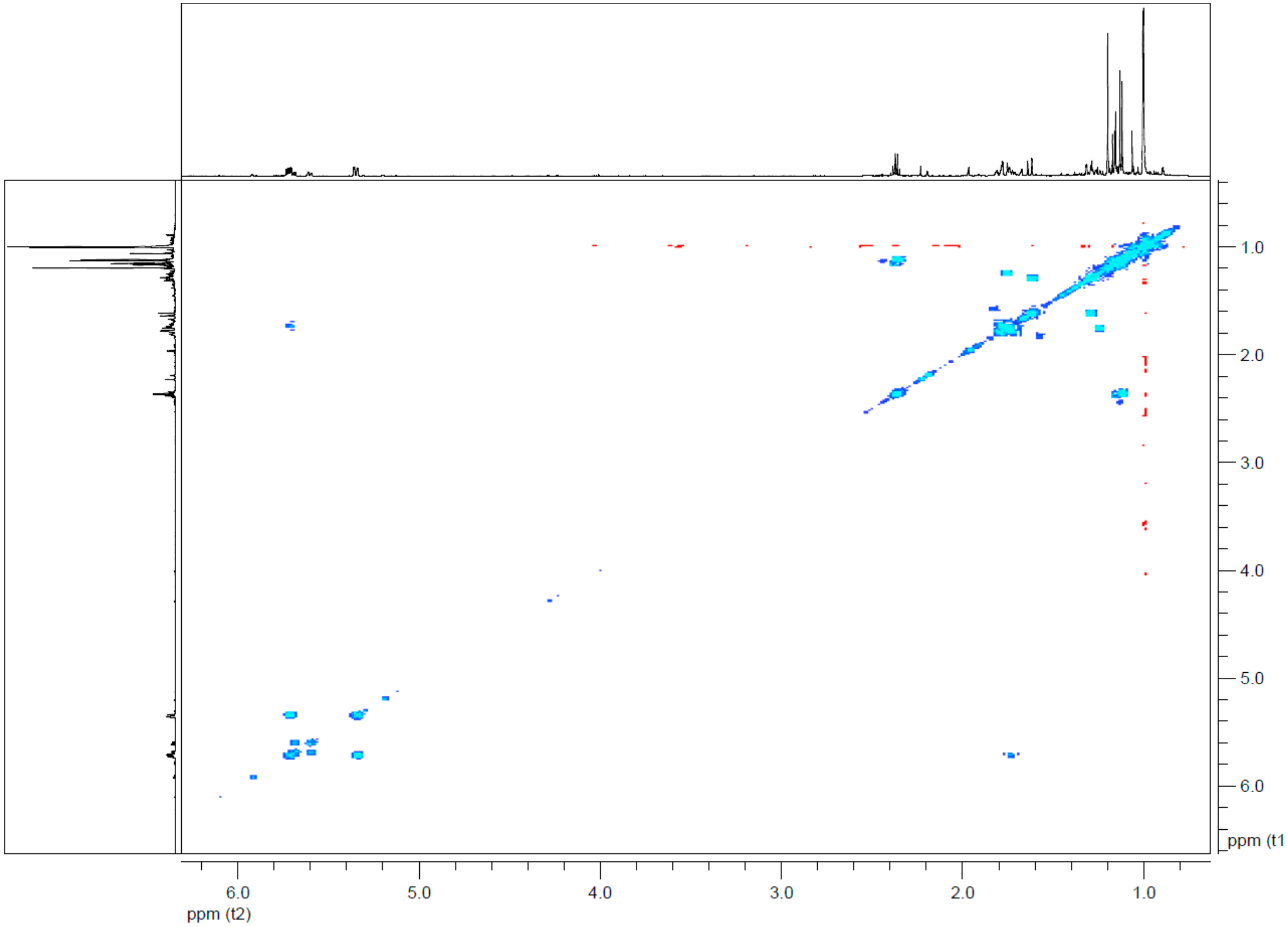

Figure S8. COSY (151 MHz, CDCl3) spectrum of acid **3a**.

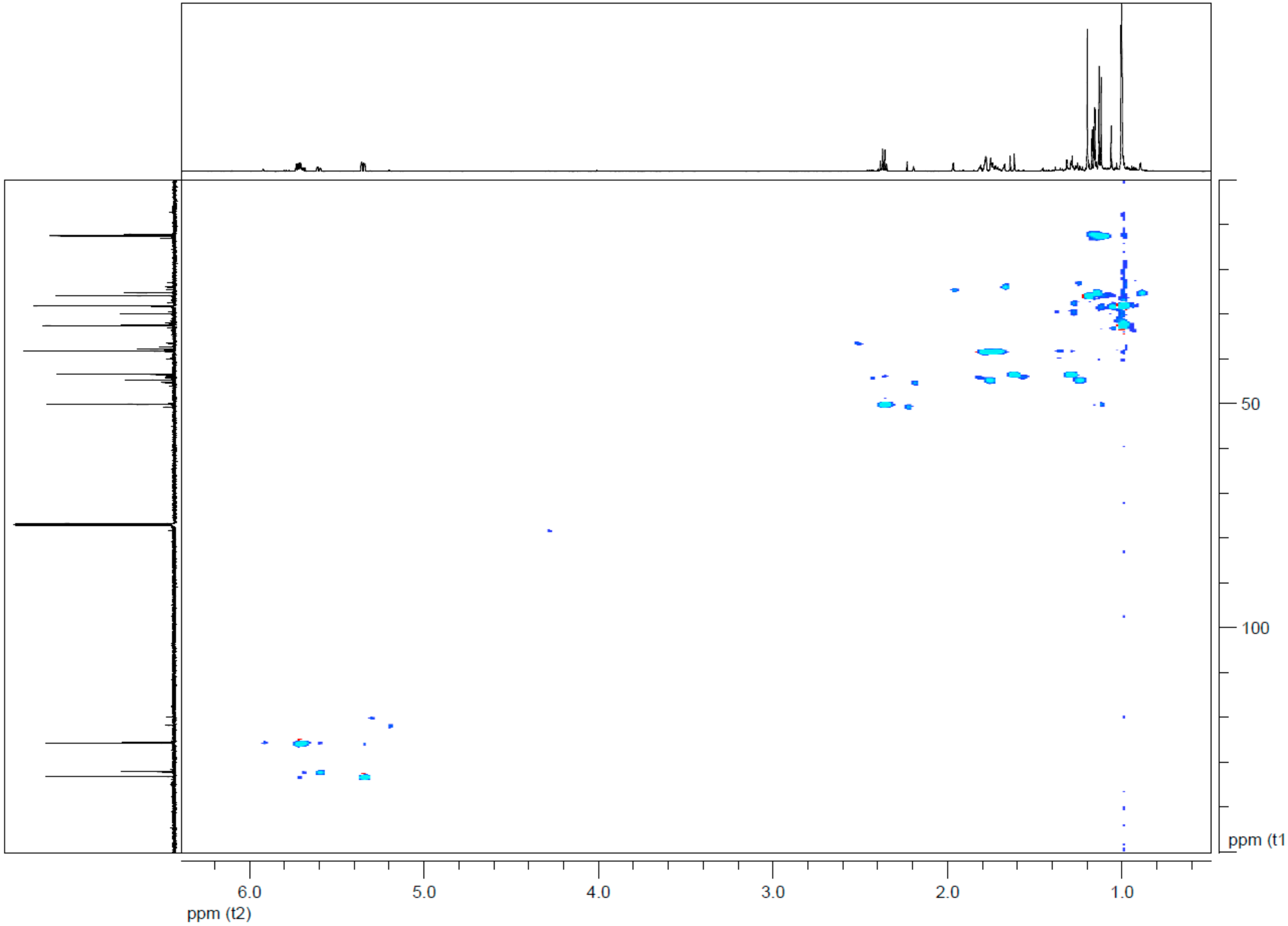

Figure S9. HMQC (151 MHz, $CDCl_3$) spectrum of acid **3a**.

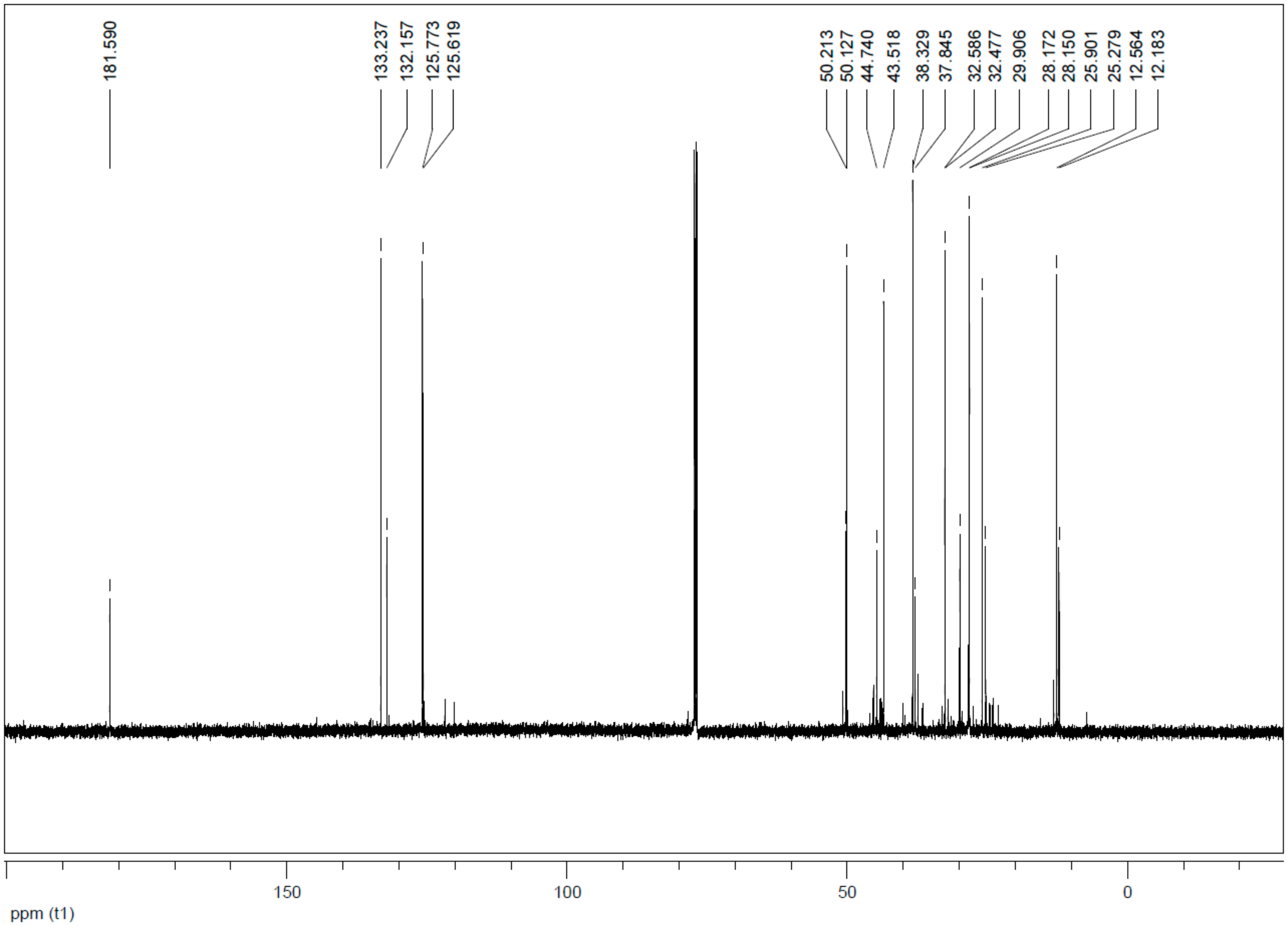

Figure S10. ^{13}C-NMR (151 MHz, $CDCl_3$) spectrum of acid **3a**.

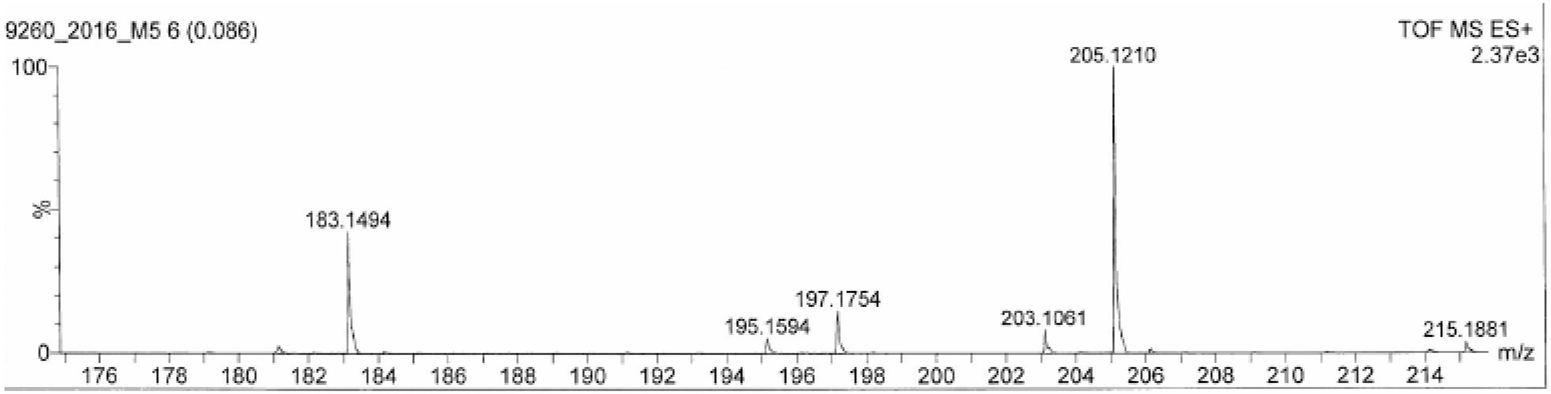

Figure S11. HRMS spectrum of acid **3a**.

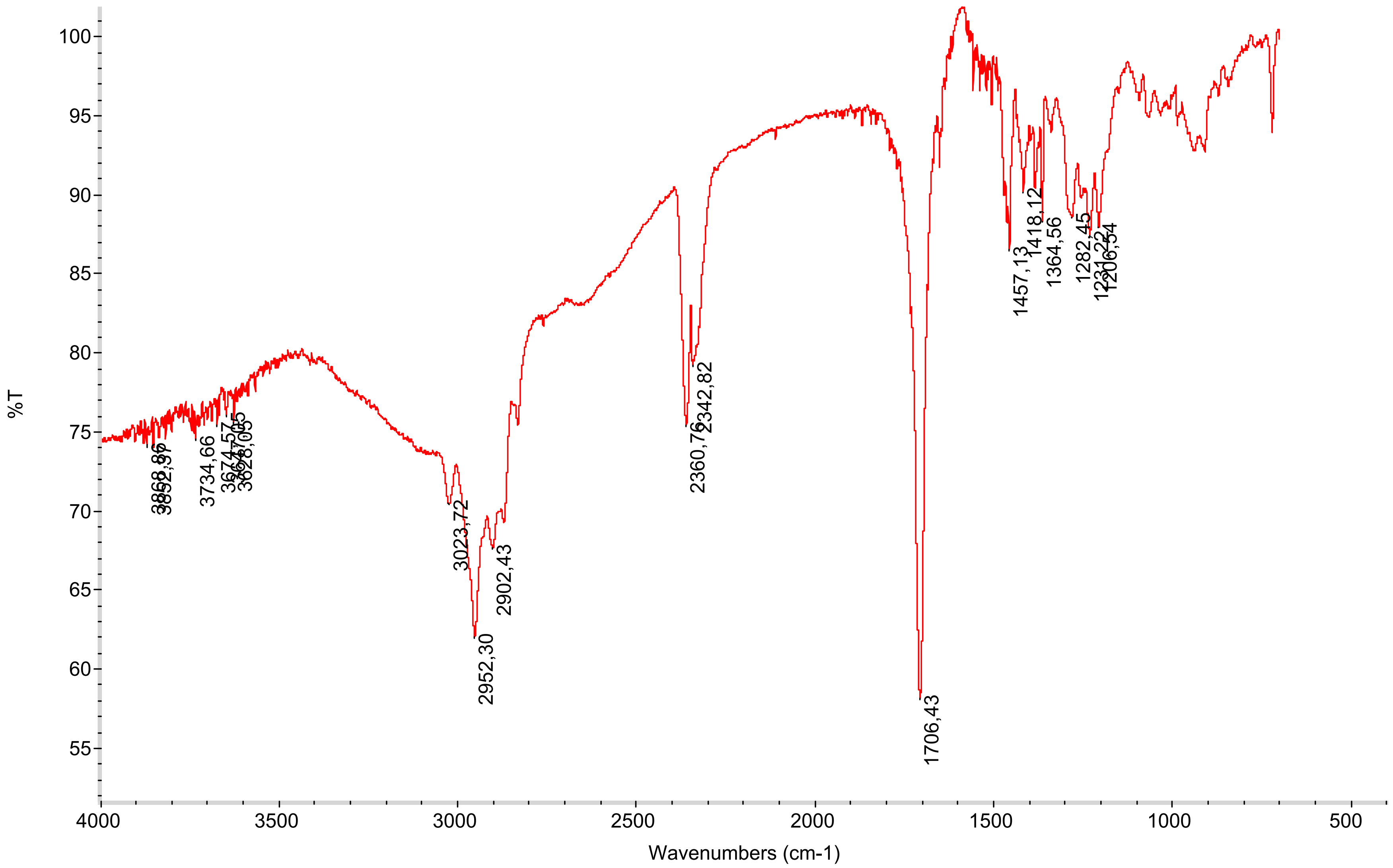

Figure S12. IR spectrum of acid **3a**.

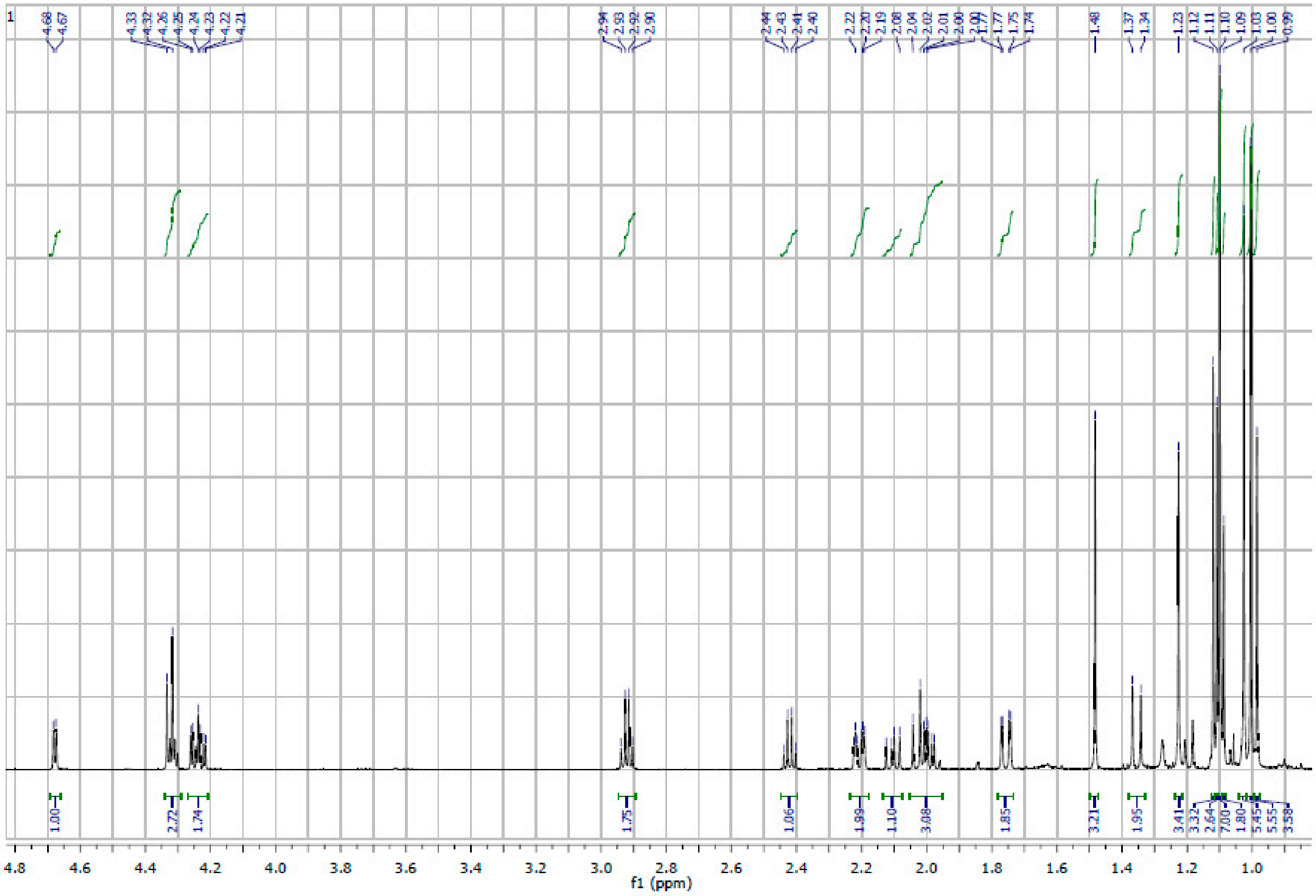

Figure S13. ^{1}H-NMR (600 MHz, $CDCl_3$) spectrum of iodolactone **4a**.

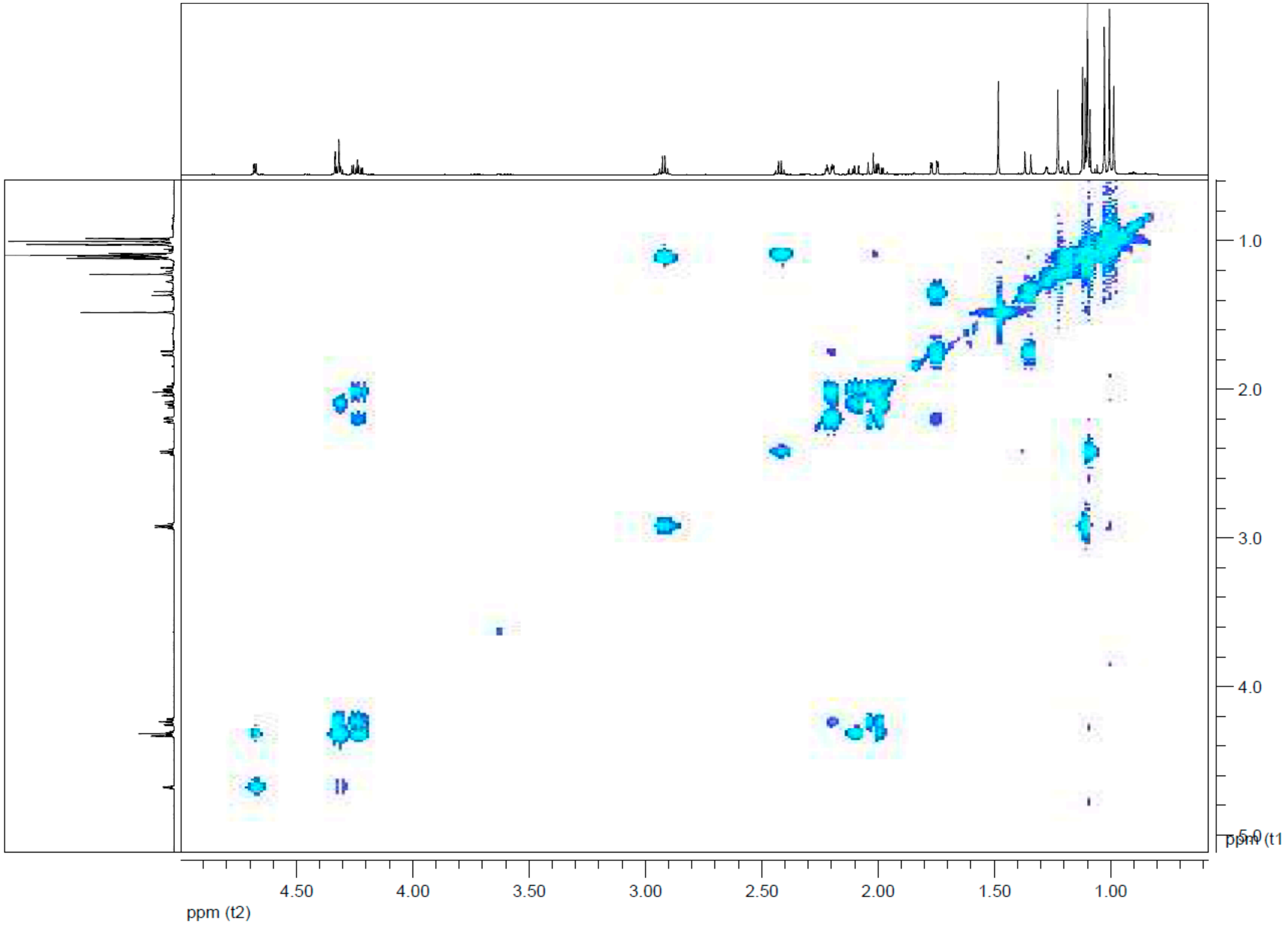

Figure S14. COSY (151 MHz, CDCl3) spectrum of iodolactone **4a**.

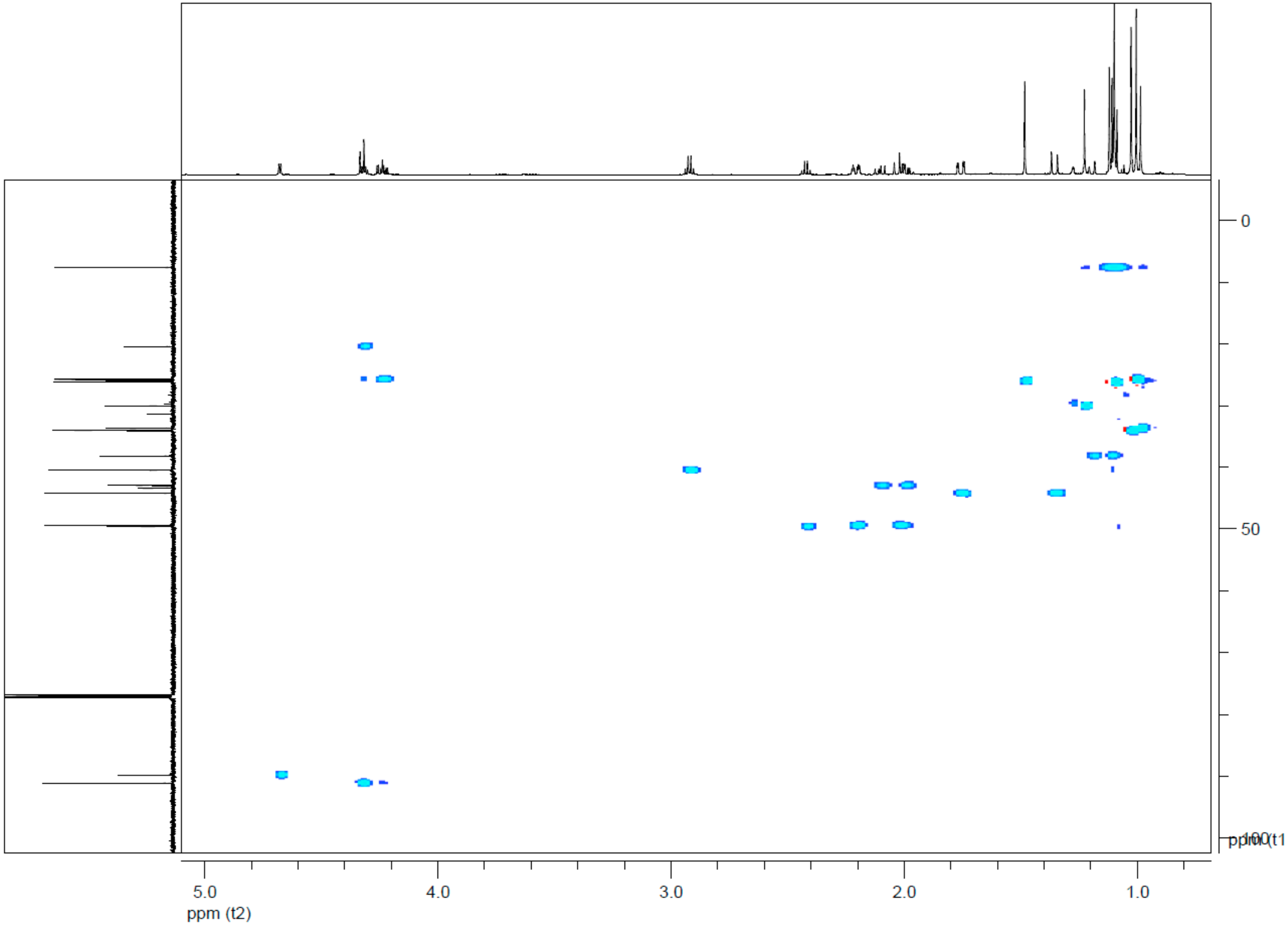

Figure S15. HMQC (151 MHz, CDCl3) spectrum of iodolactone **4a**.

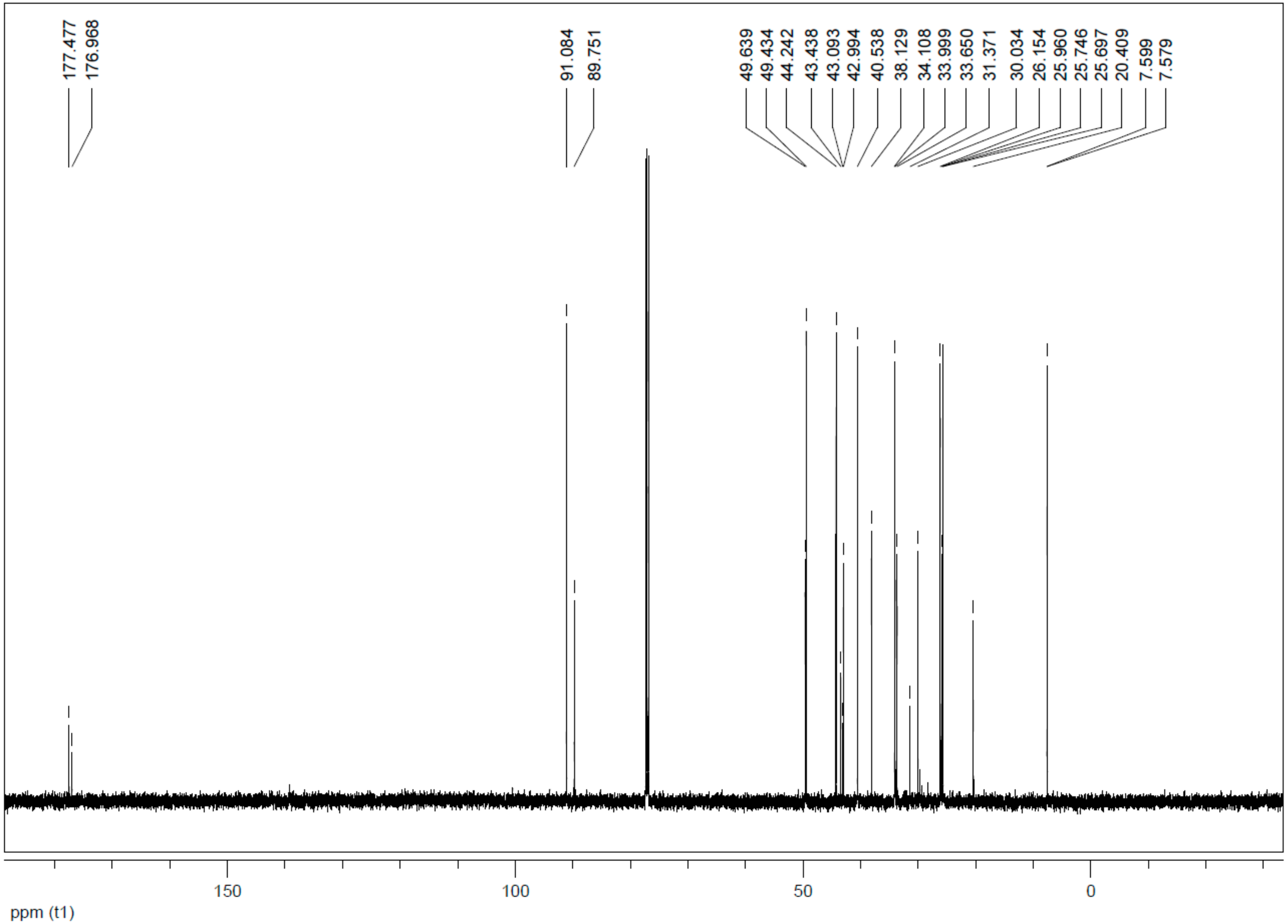

Figure S16. ^{13}C-NMR (151 MHz, $CDCl_3$) spectrum of iodolactone **4a**.

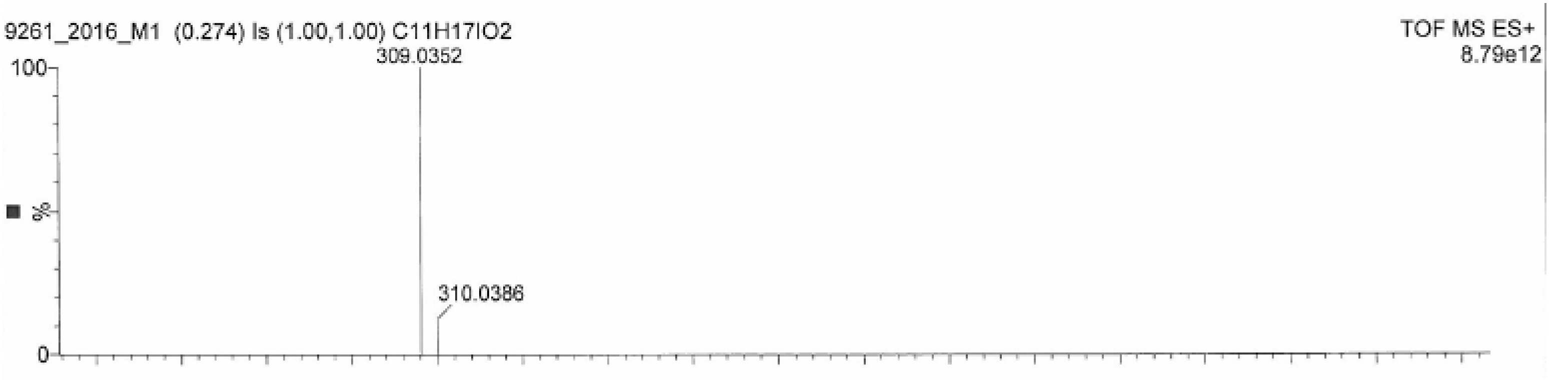

Figure S17. HRMS spectrum of iodolactone **4a**.

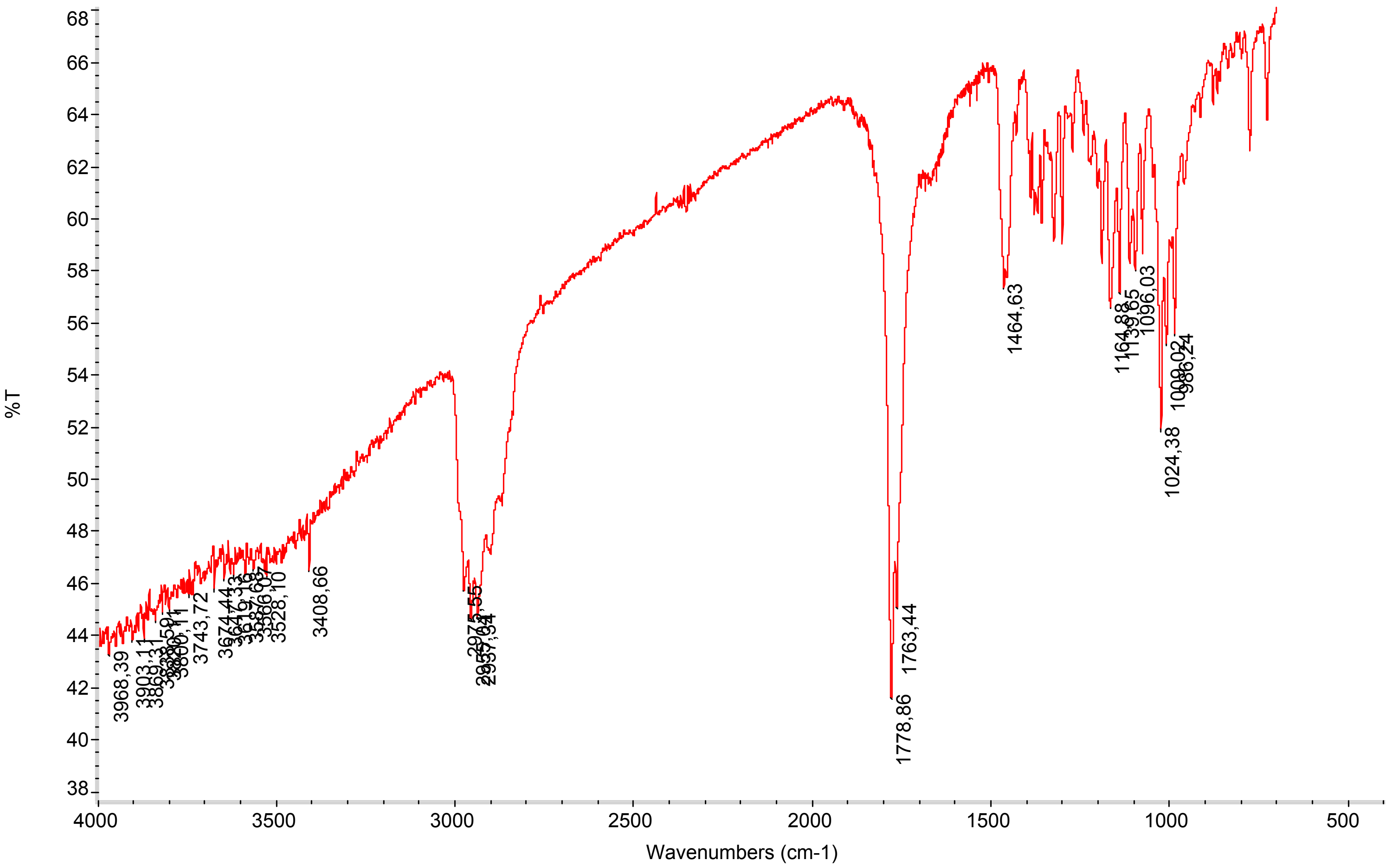

Figure S18. IR spectrum of iodolactone **4a**.

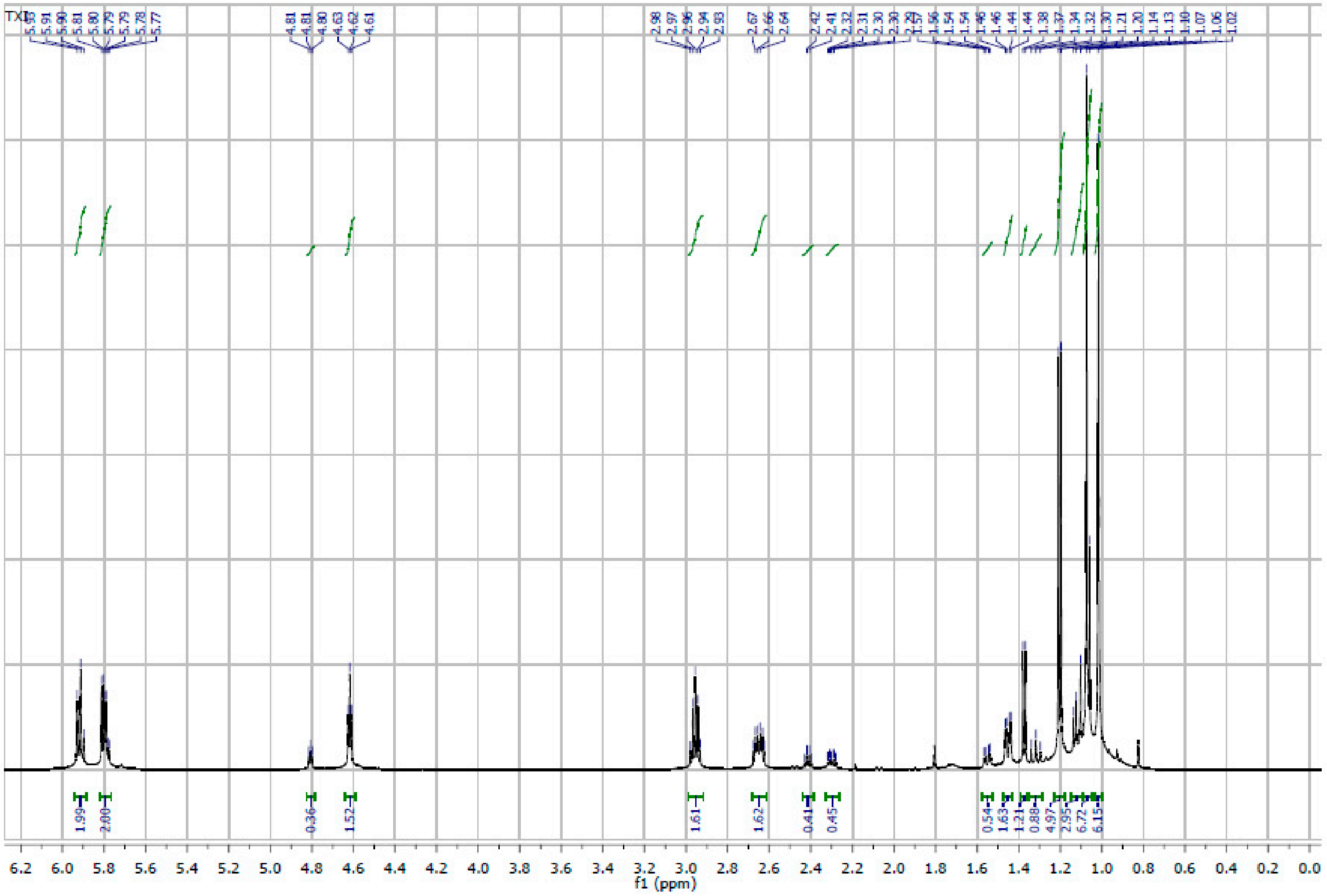

Figure S19. ^{1}H-NMR (600 MHz, $CDCl_3$) spectrum of unsaturated lactone **5a**.

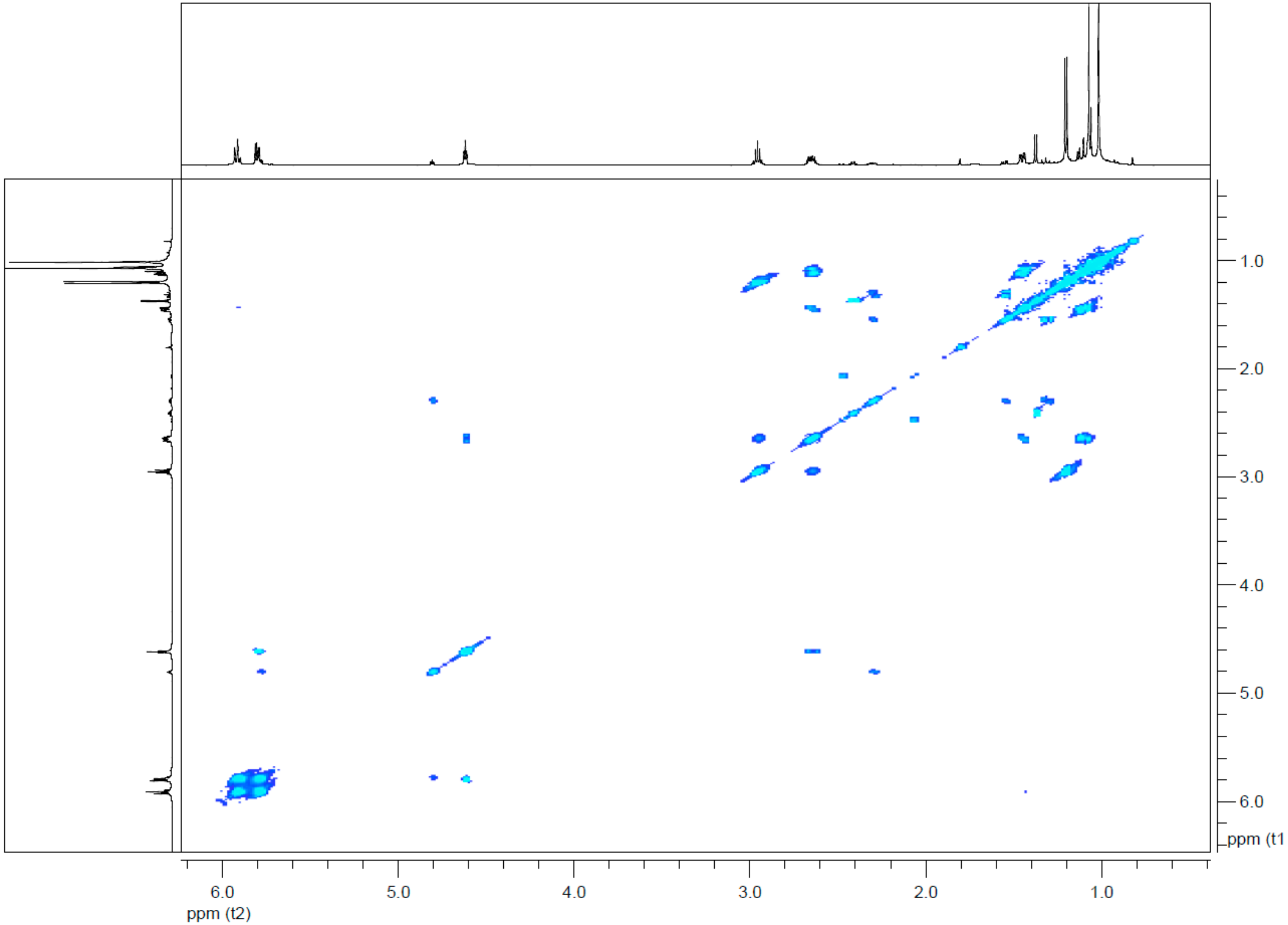

Figure S20. COSY (151 MHz, $CDCl_3$) spectrum of unsaturated lactone **5a**.

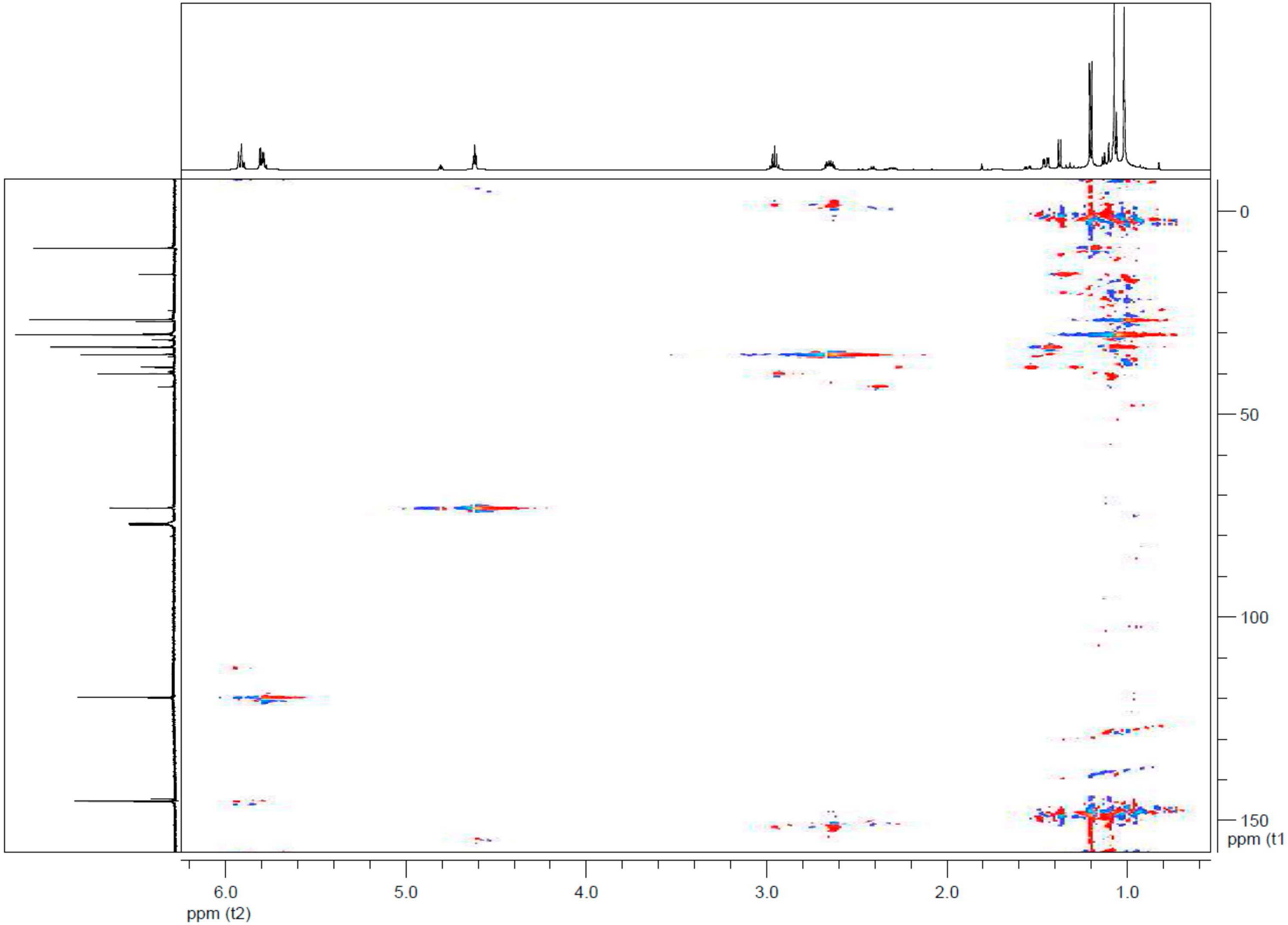

Figure S21. HMQC (151 MHz, $CDCl_3$) spectrum of unsaturated lactone **5a**.

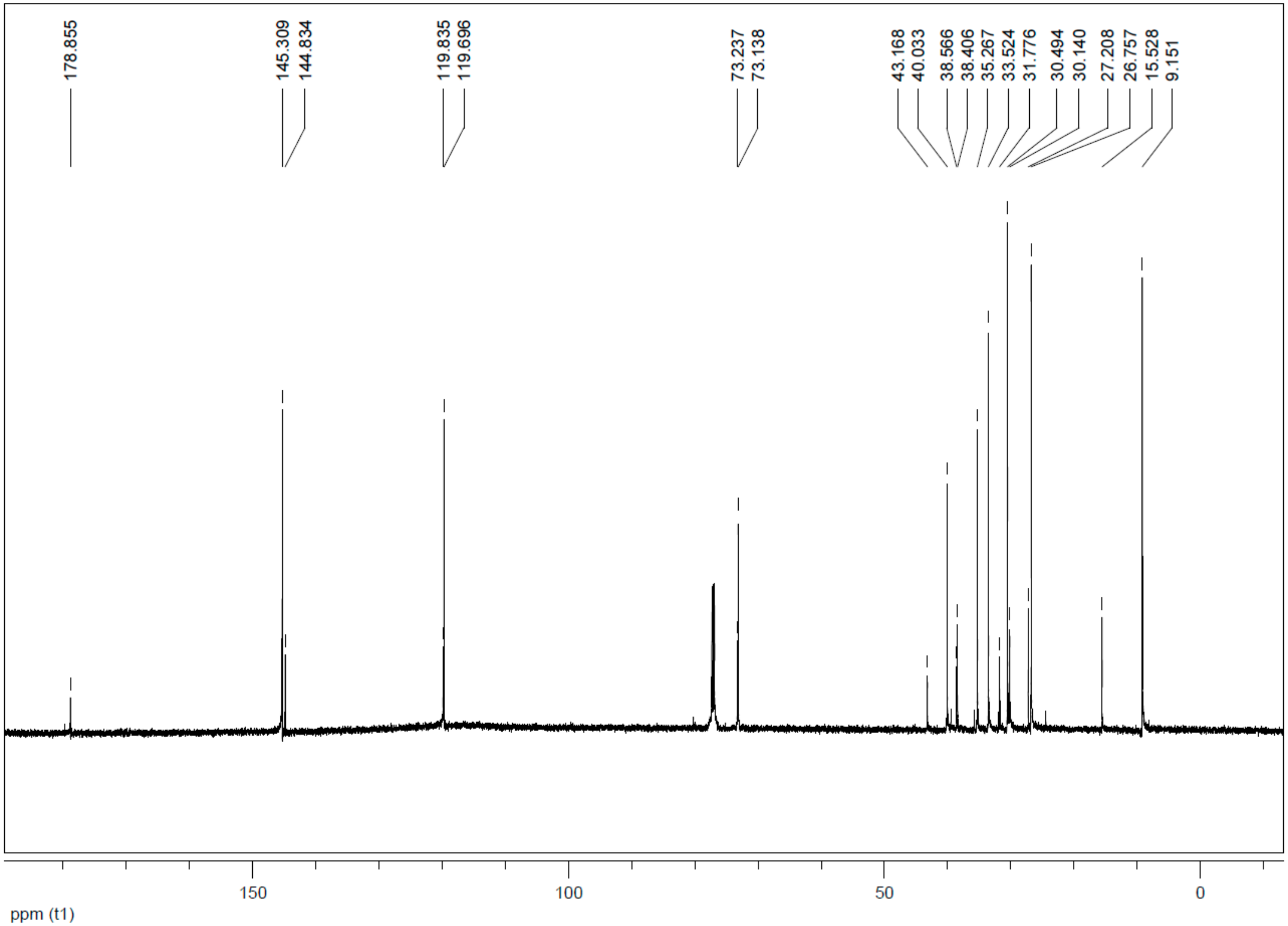

Figure S22. ^{13}C-NMR (151 MHz, $CDCl_3$) spectrum of unsaturated lactone **5a**.

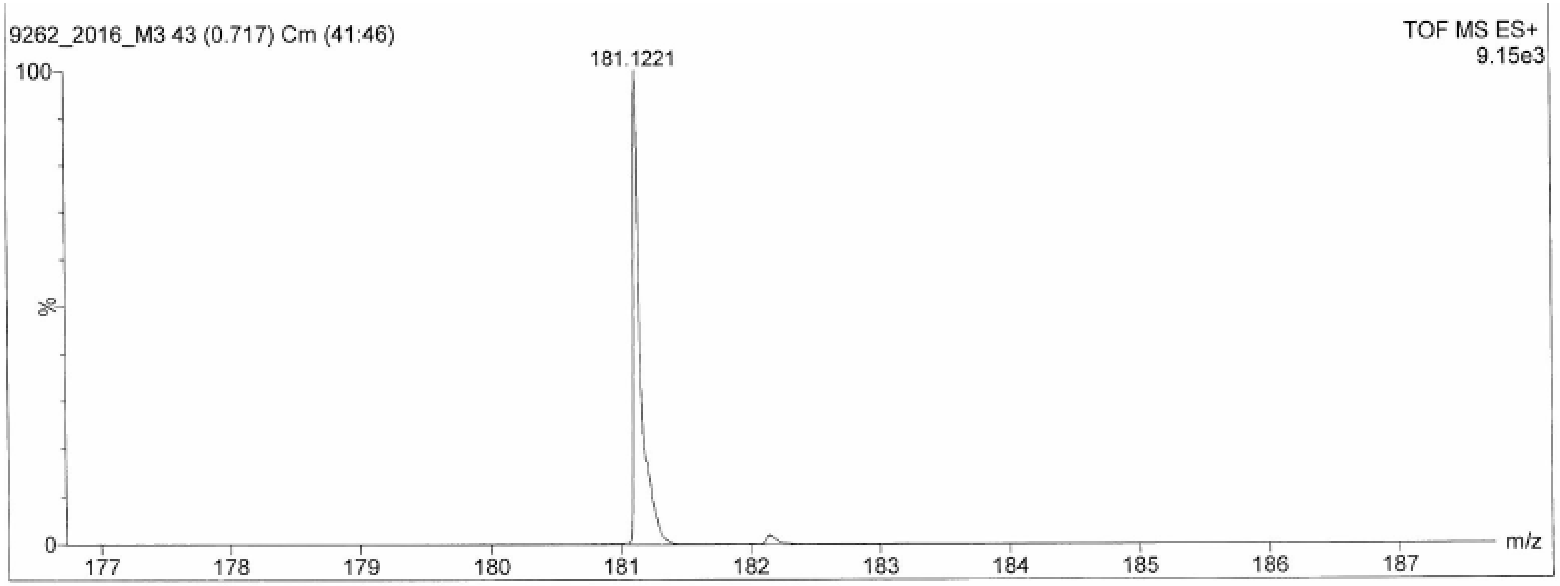

Figure S23. HRMS spectrum of unsaturated lactone **5a**.

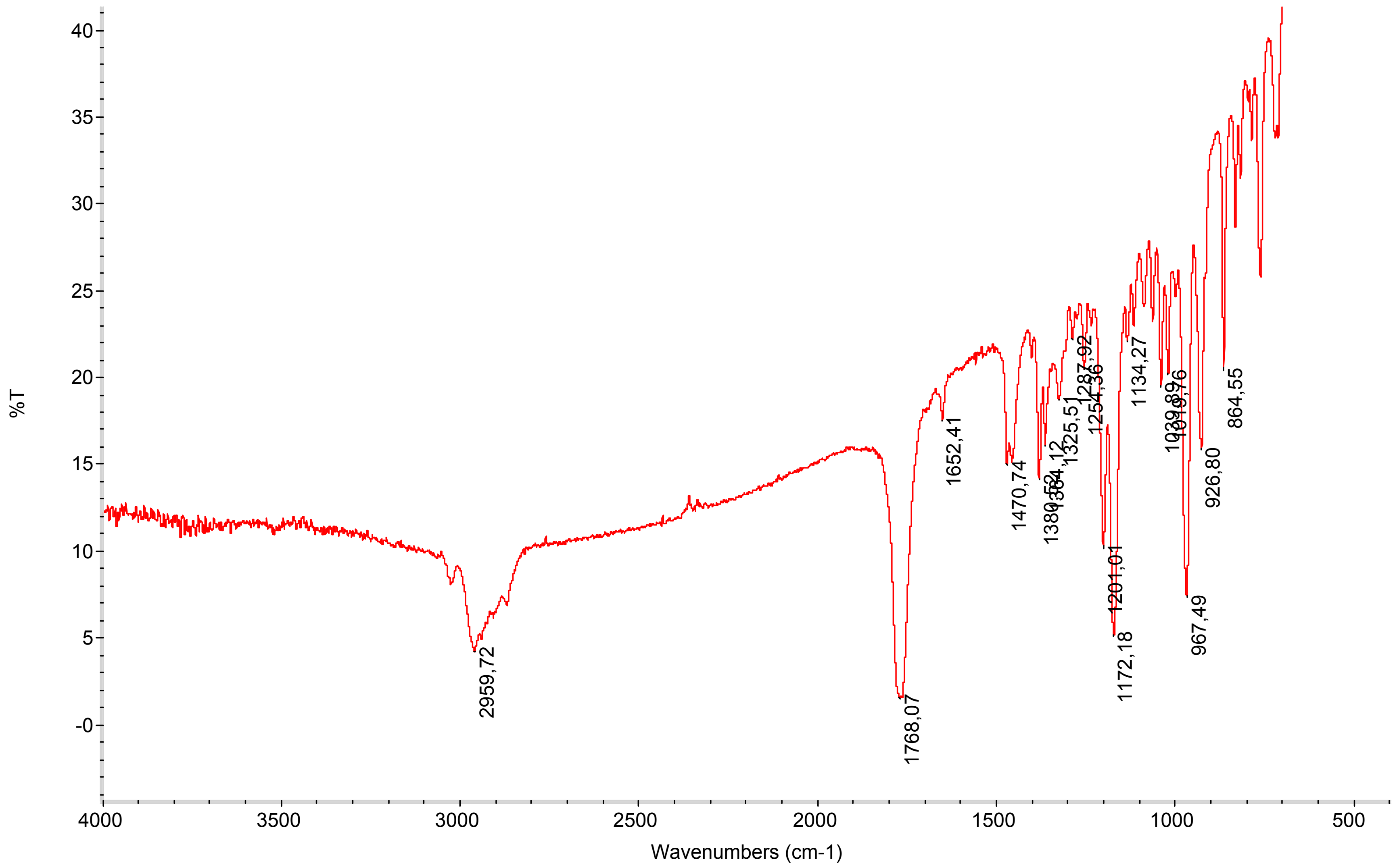

Figure S24. IR spectrum of unsaturated lactone **5a**.

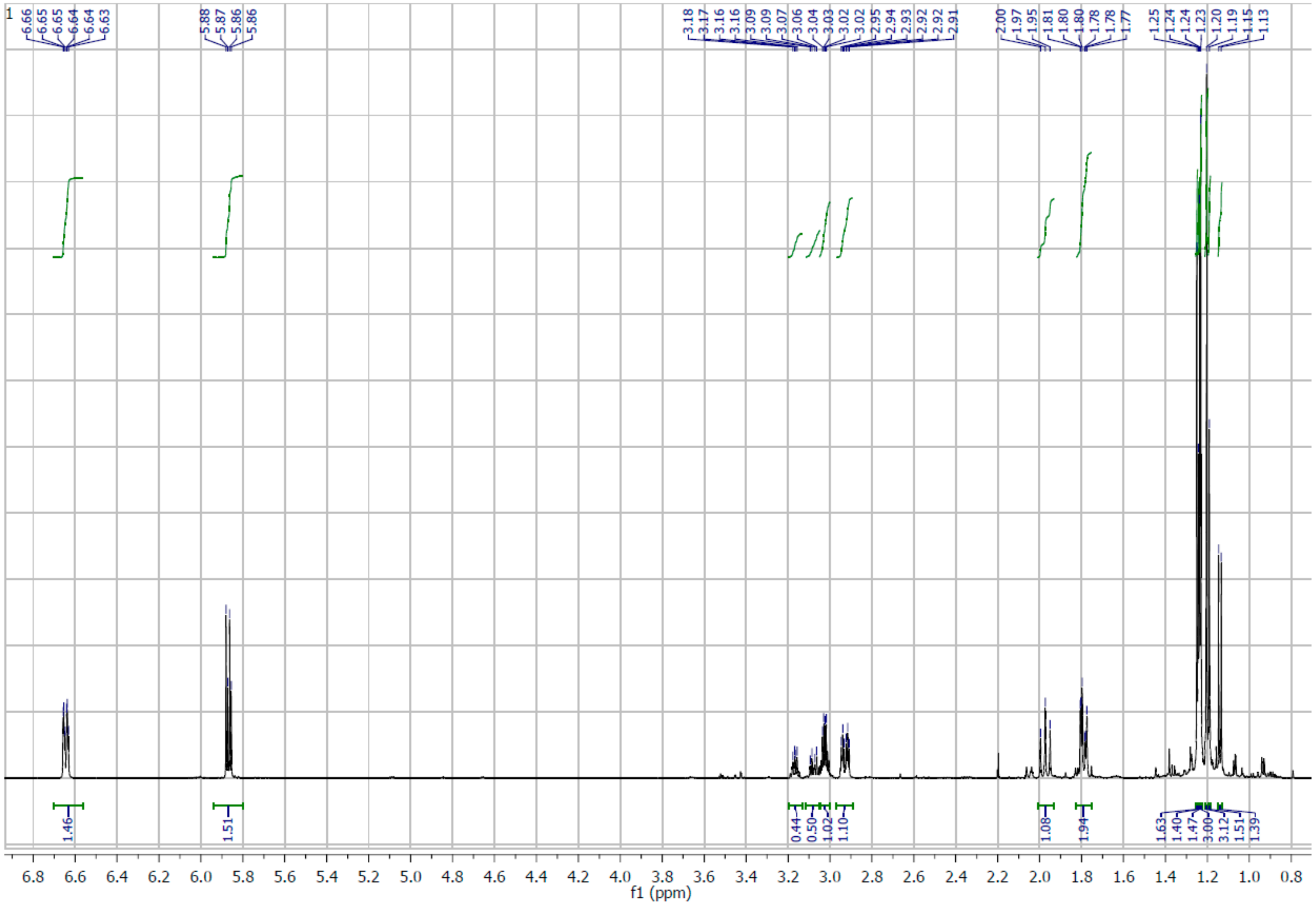

Figure S25. ^{1}H-NMR (600 MHz, $CDCl_3$) spectrum of hydroxylactone **6a**.

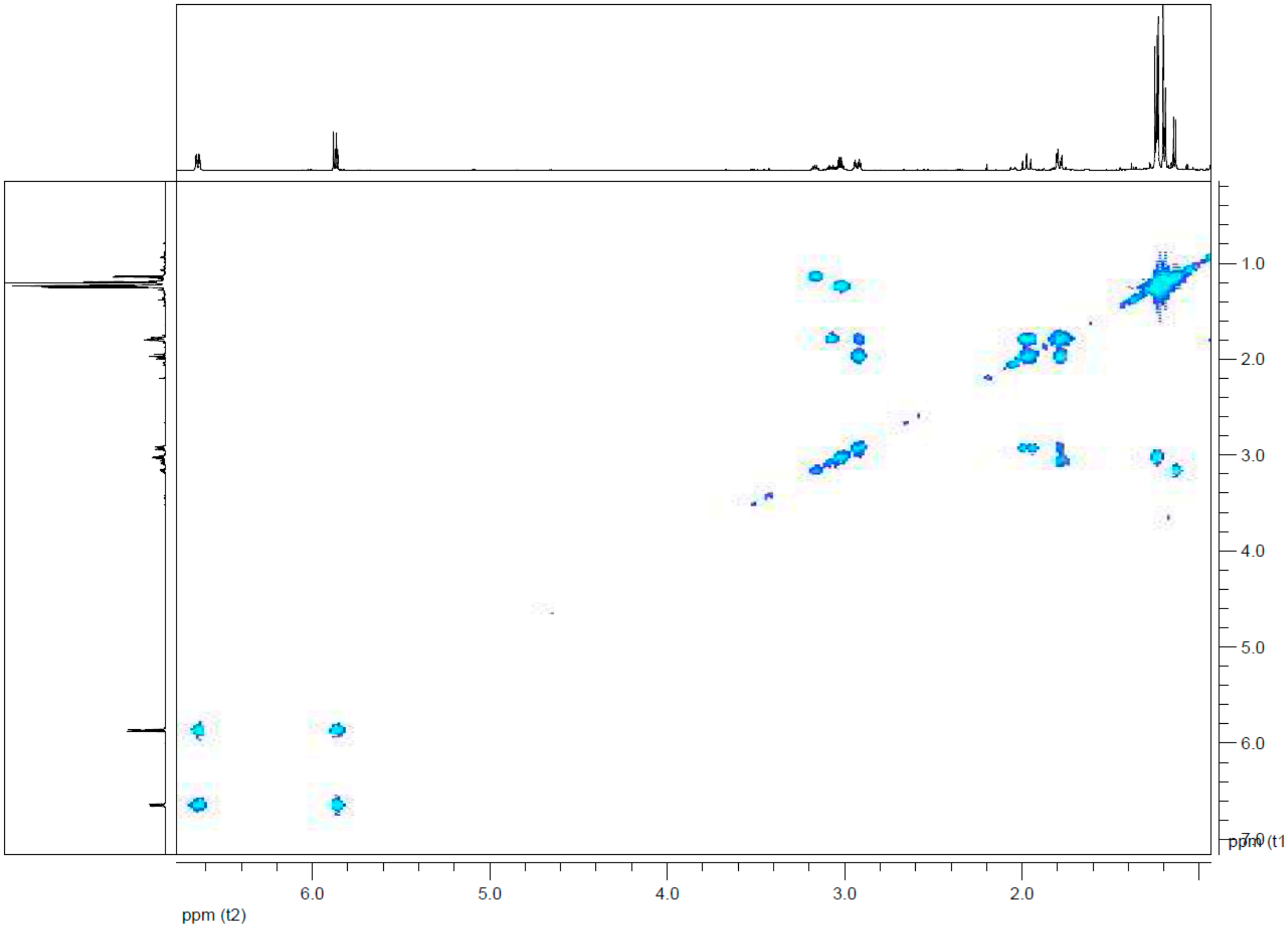

Figure S26. COSY (151 MHz, $CDCl_3$) spectrum of hydroxylactone **6a**.

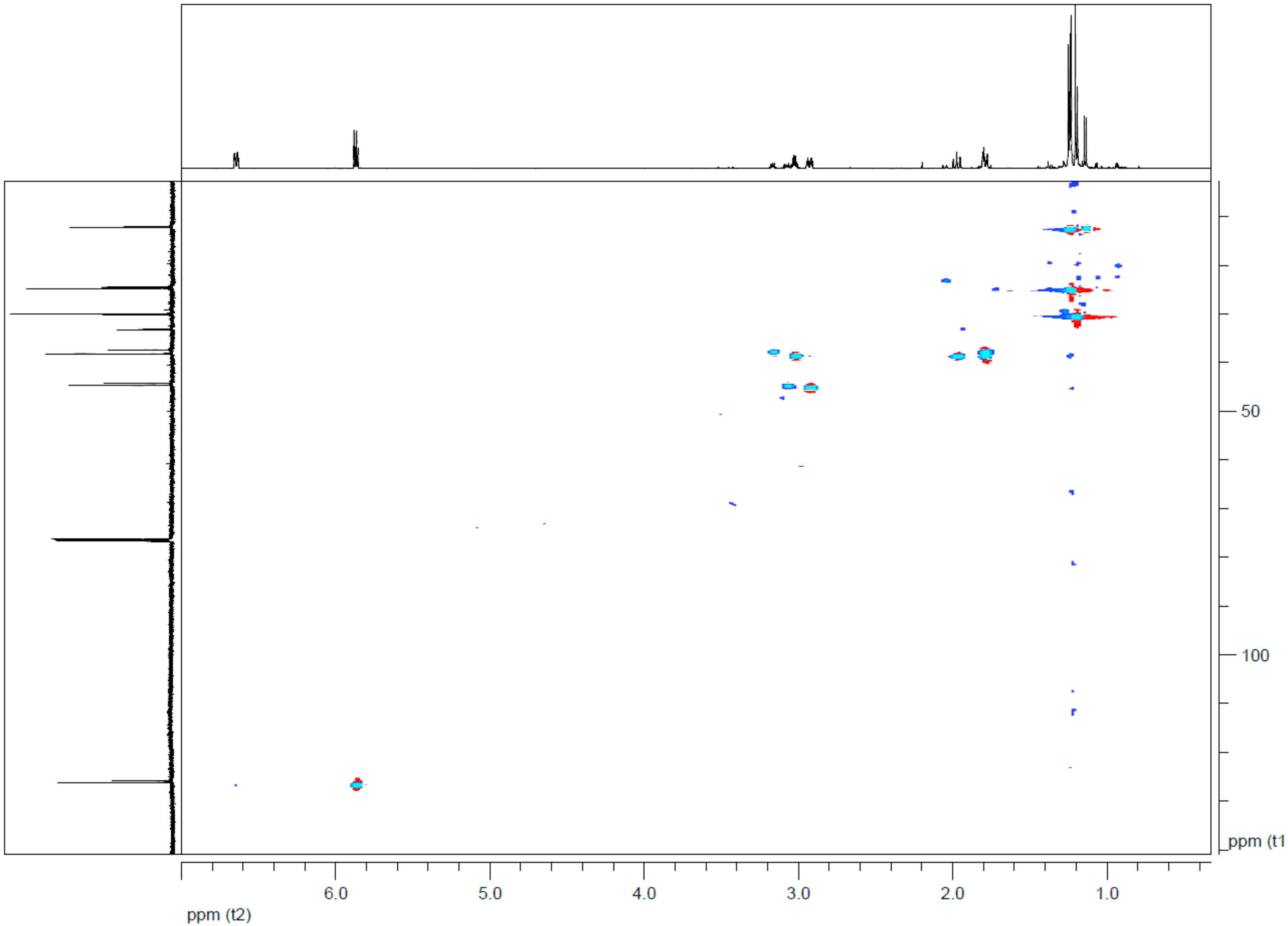

Figure S27. HMQC (151 MHz, $CDCl_3$) spectrum of hydroxylactone **6a**.

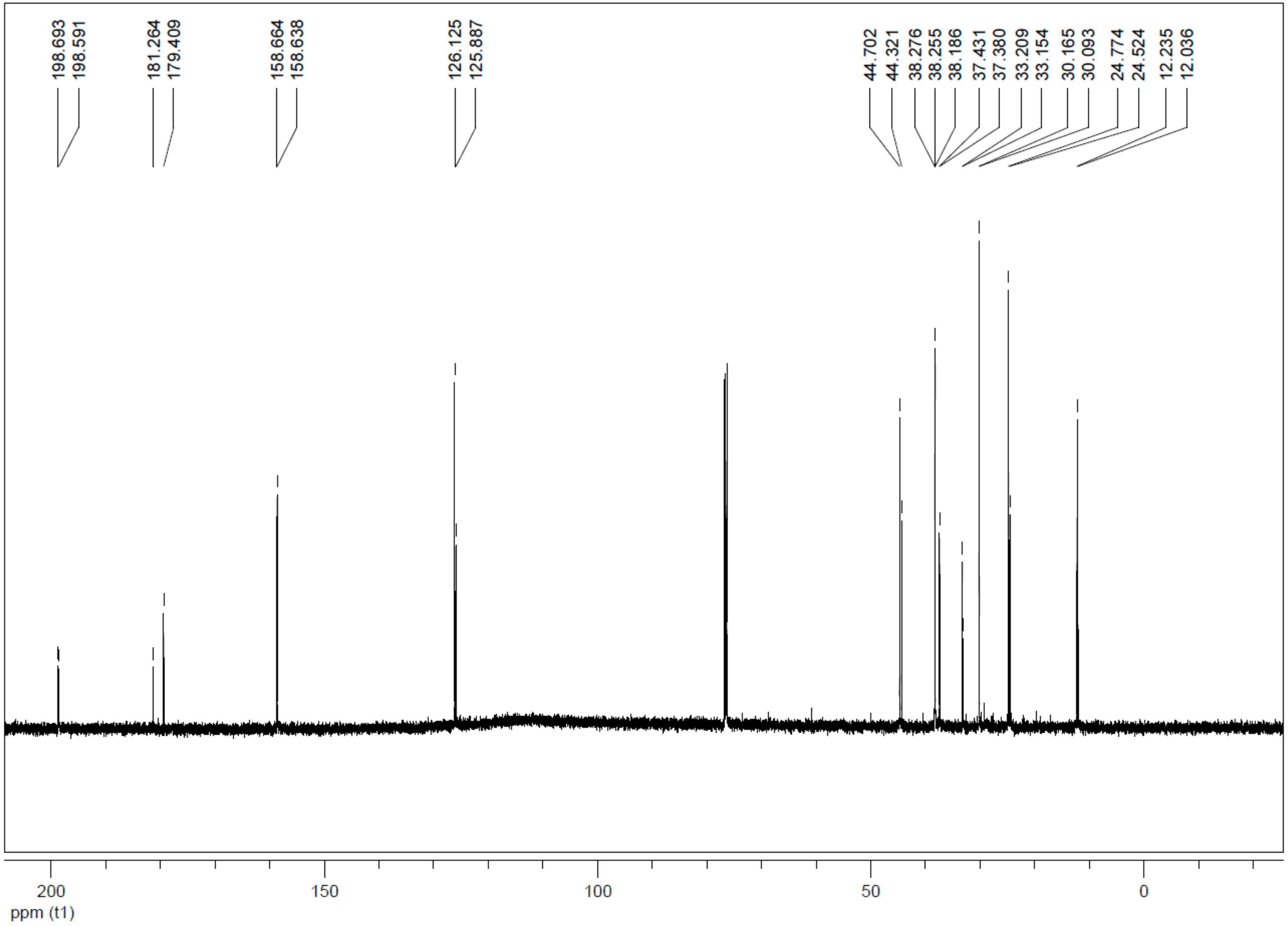

Figure S28. ^{13}C-NMR (151 MHz, $CDCl_3$) spectrum of hydroxylactone **6a**.

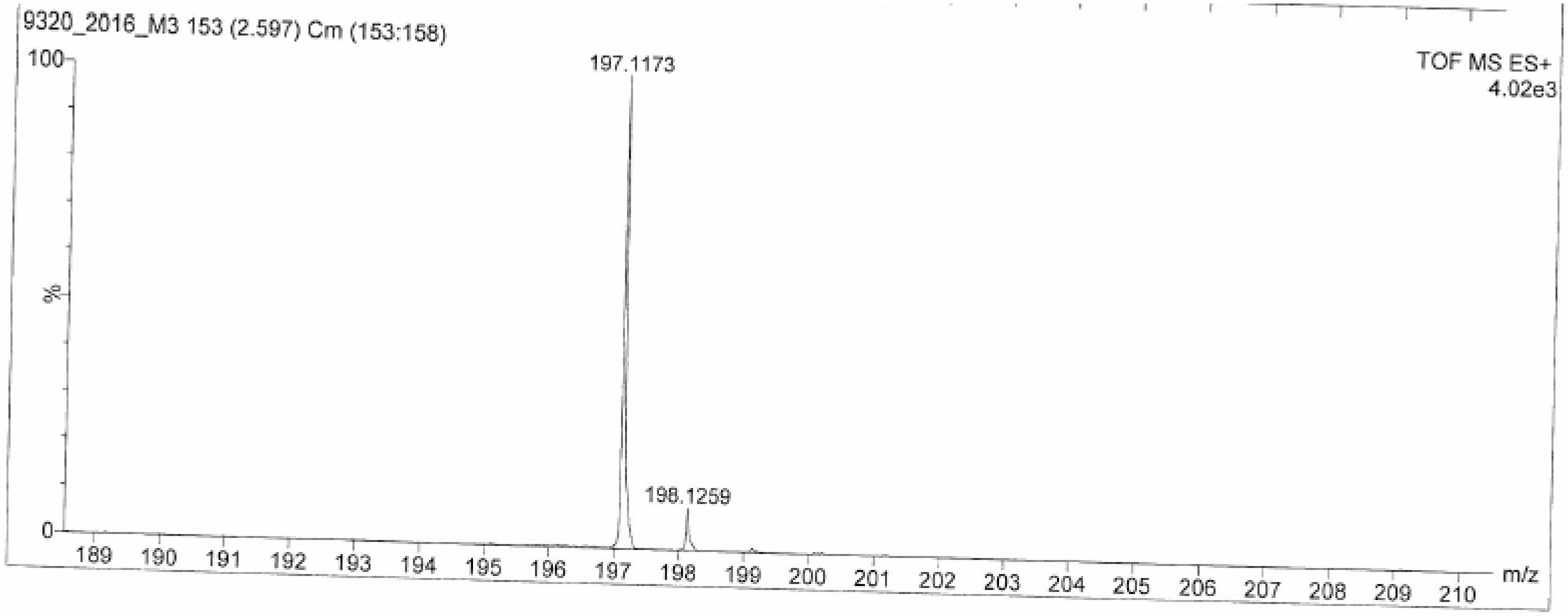

Figure S29. HRMS spectrum of hydroxylactone **6a**.

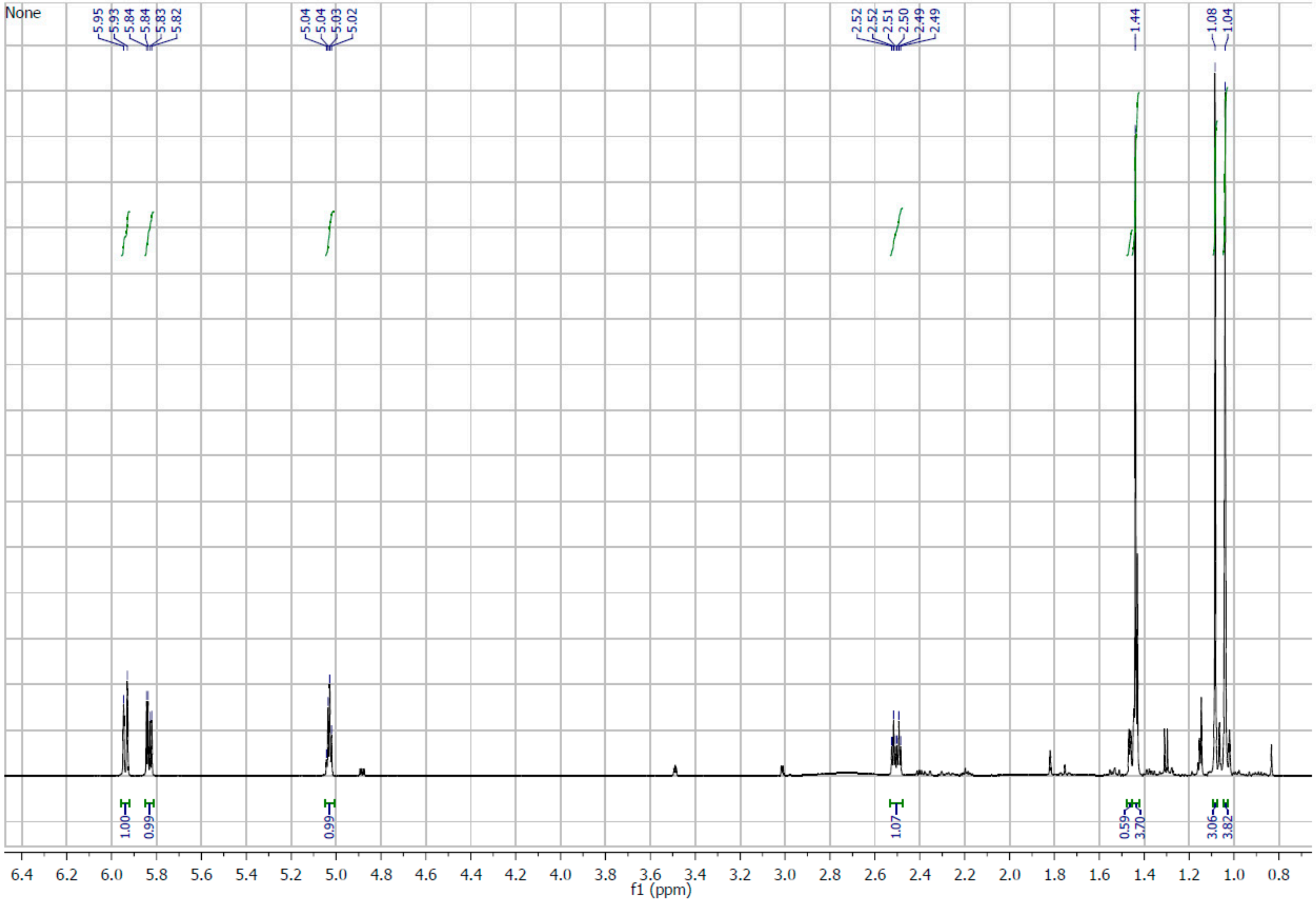

Figure S30. ^{1}H-NMR (600 MHz, $CDCl_3$) spectrum of hydroxylactone **7a**.

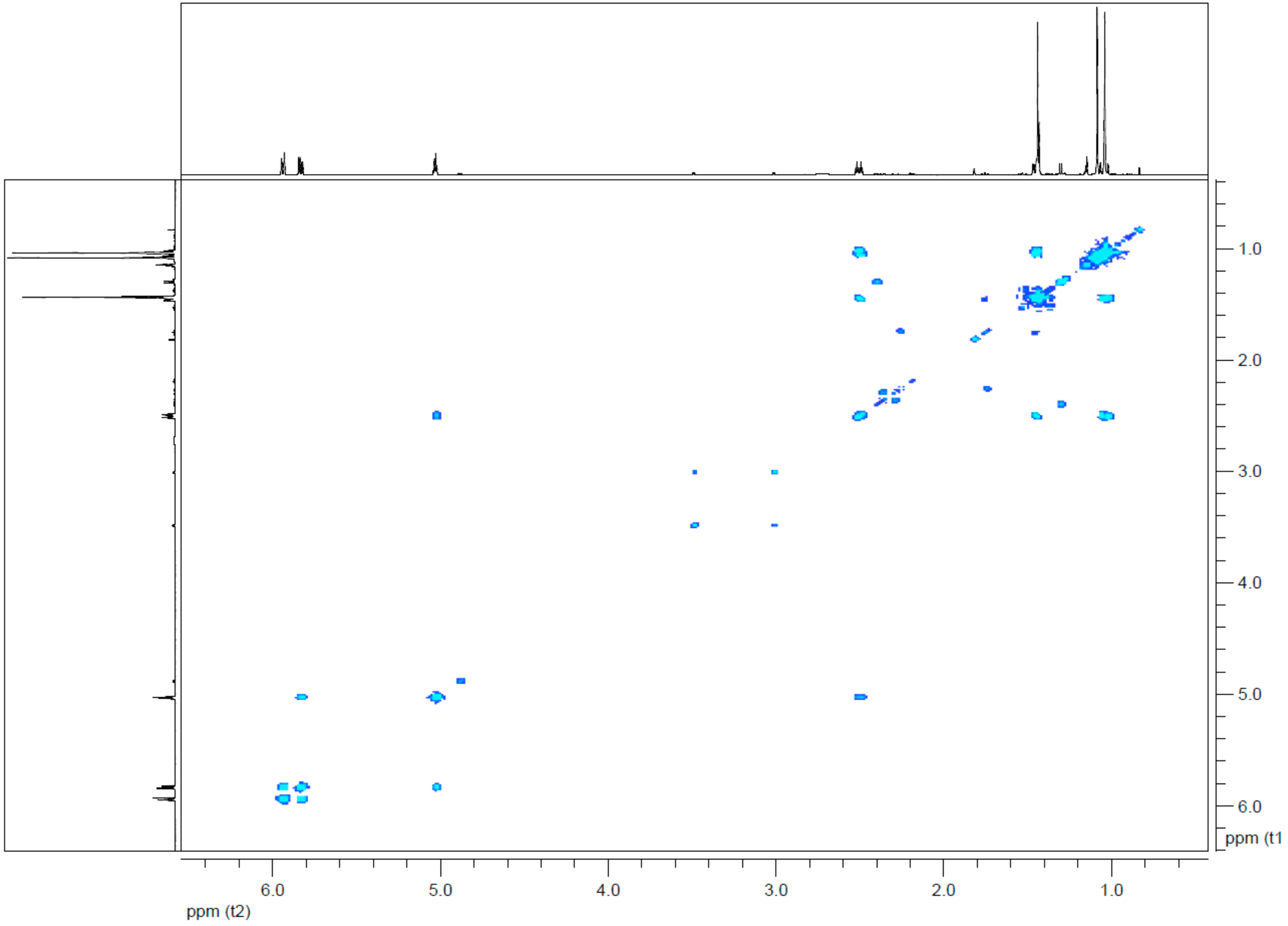

Figure S31. COSY (151 MHz, $CDCl_3$) spectrum of hydroxylactone **7a**.

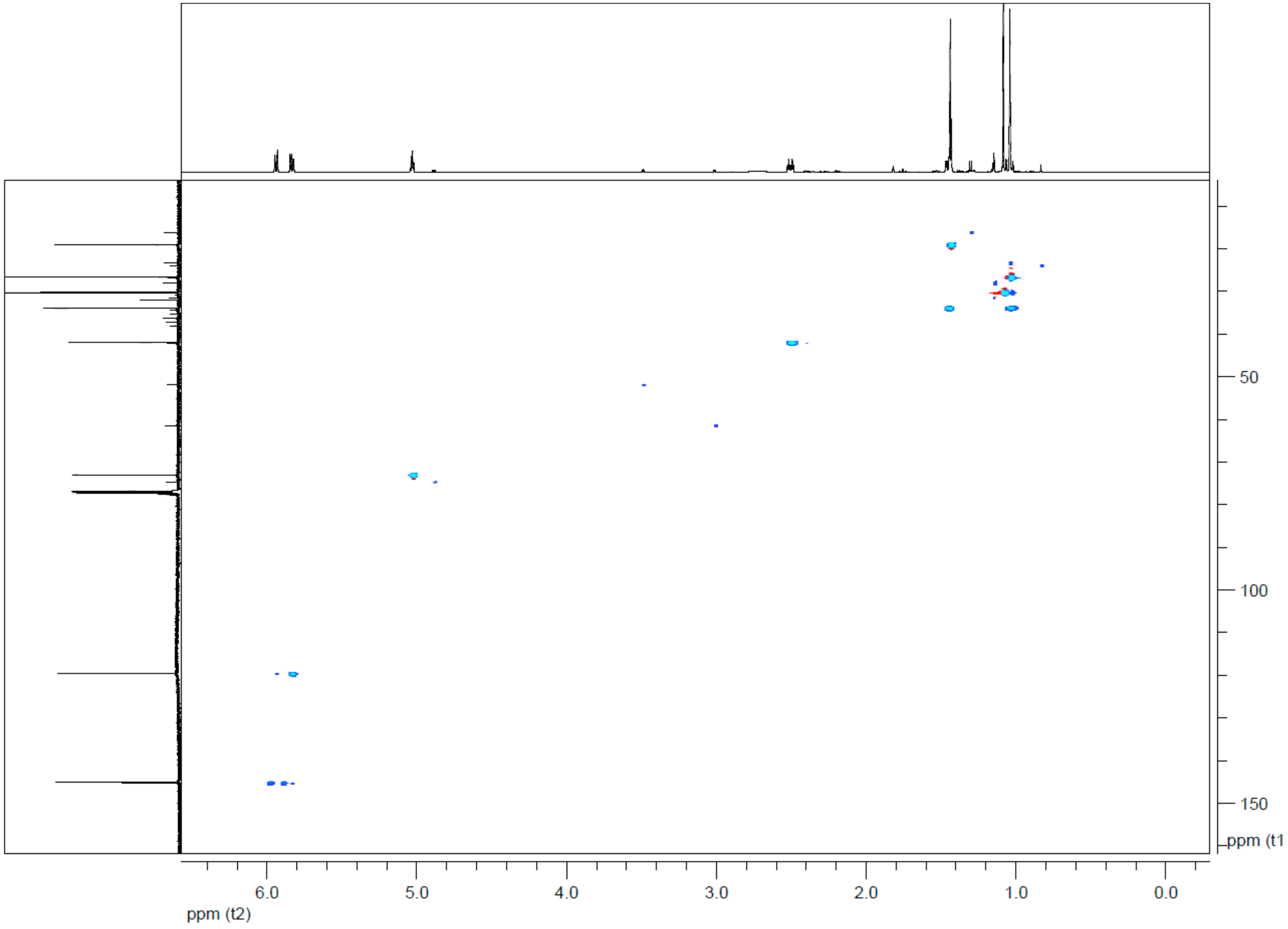

Figure S32. HMQC (151 MHz, $CDCl_3$) spectrum of hydroxylactone **7a**.

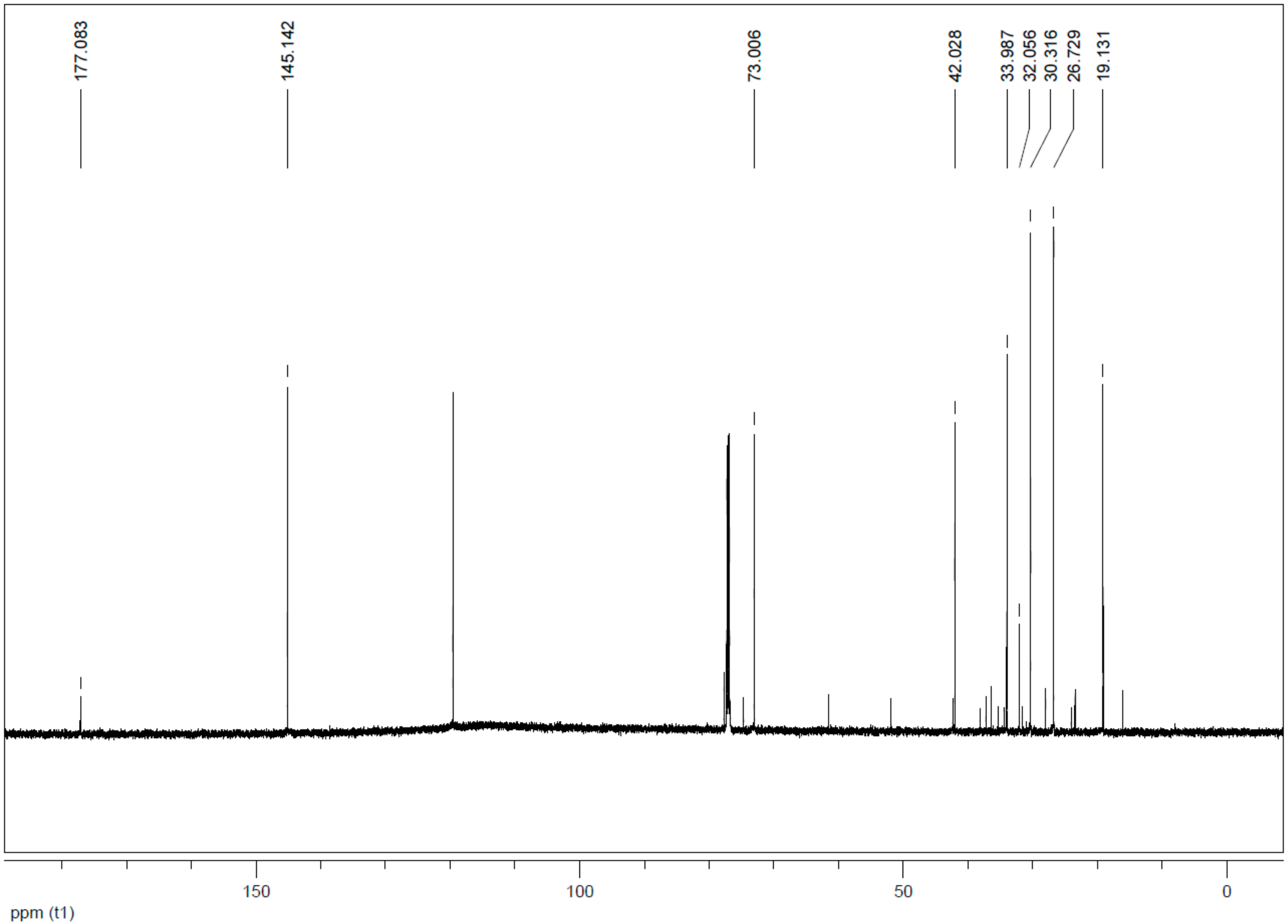

Figure S33. ^{13}C-NMR (151 MHz, $CDCl_3$) spectrum of hydroxylactone **7a**.

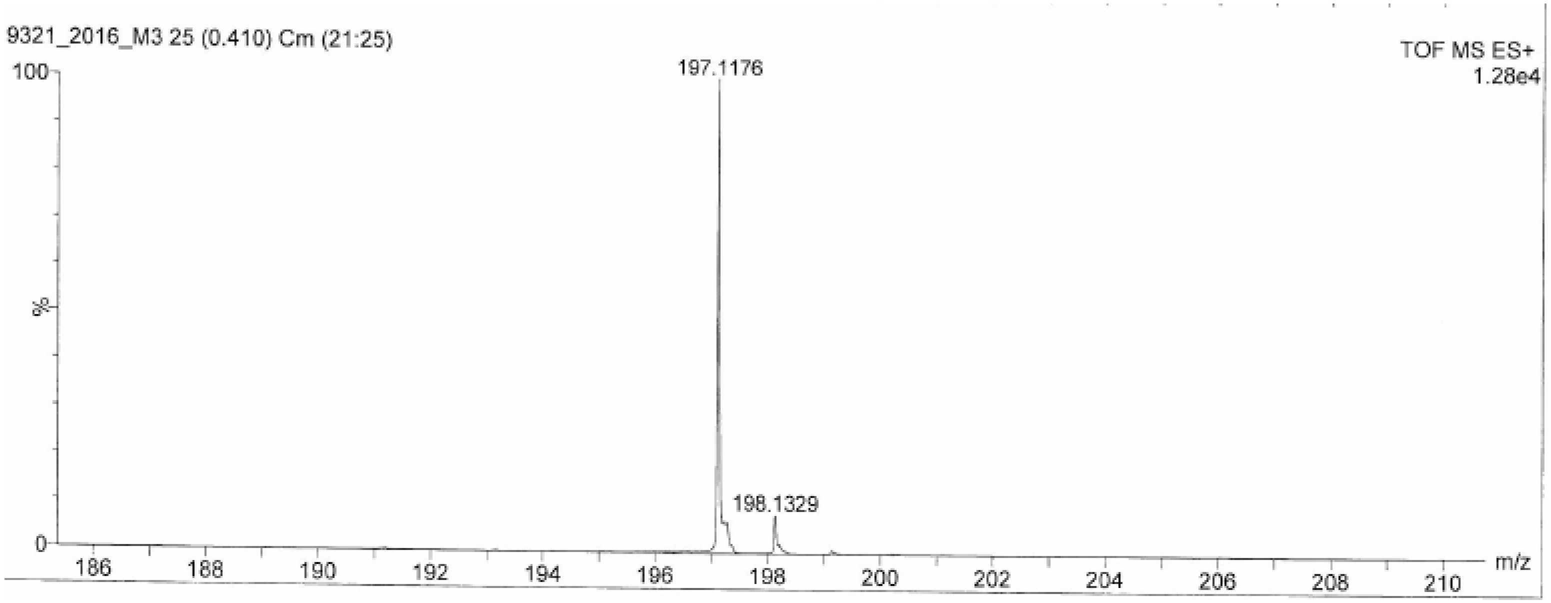

Figure S34. HRMS spectrum of hydroxylactone **7a**.

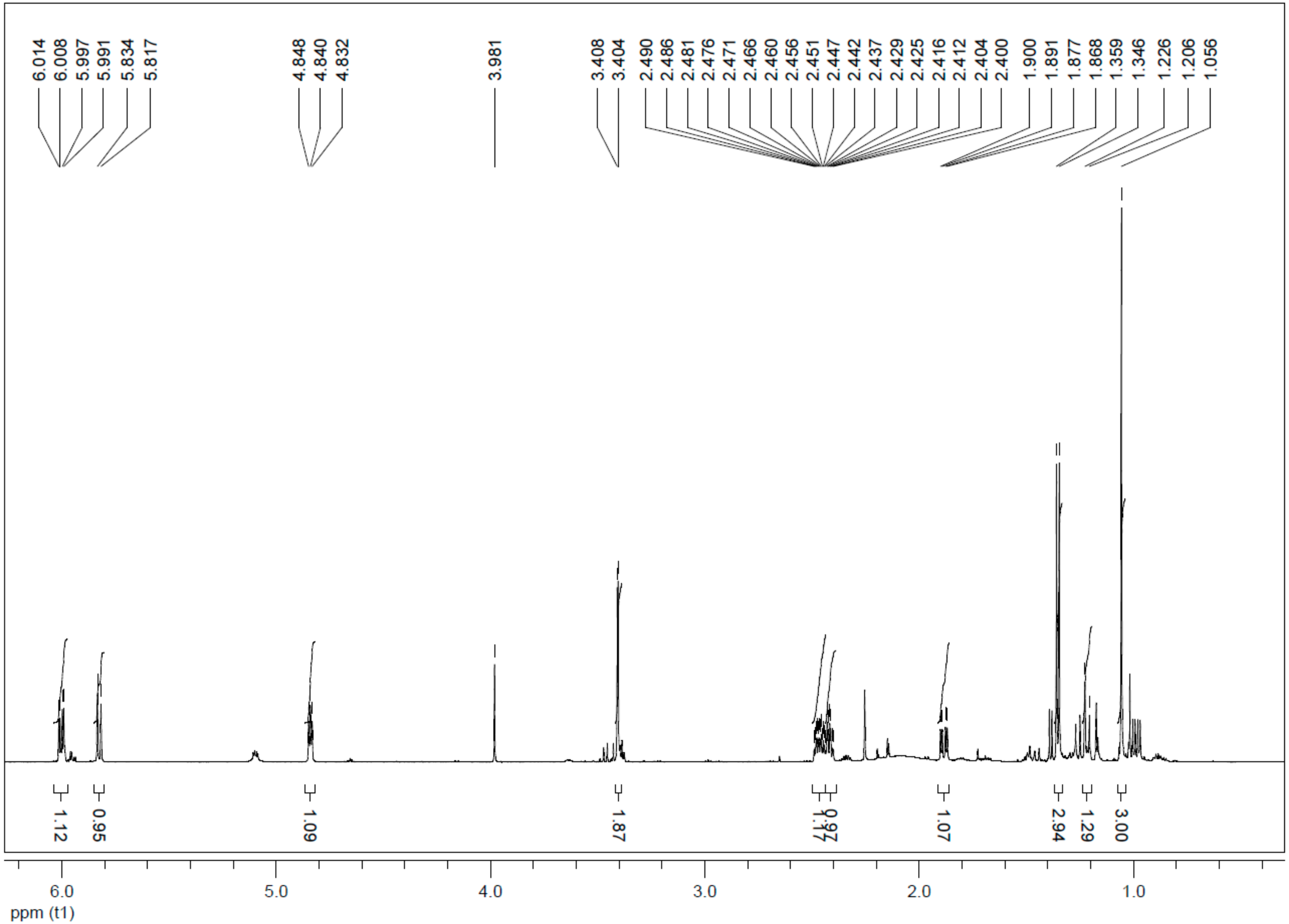

Figure S35. ^{1}H-NMR (600 MHz, $CDCl_3$) spectrum of hydroxylactone **8a**.

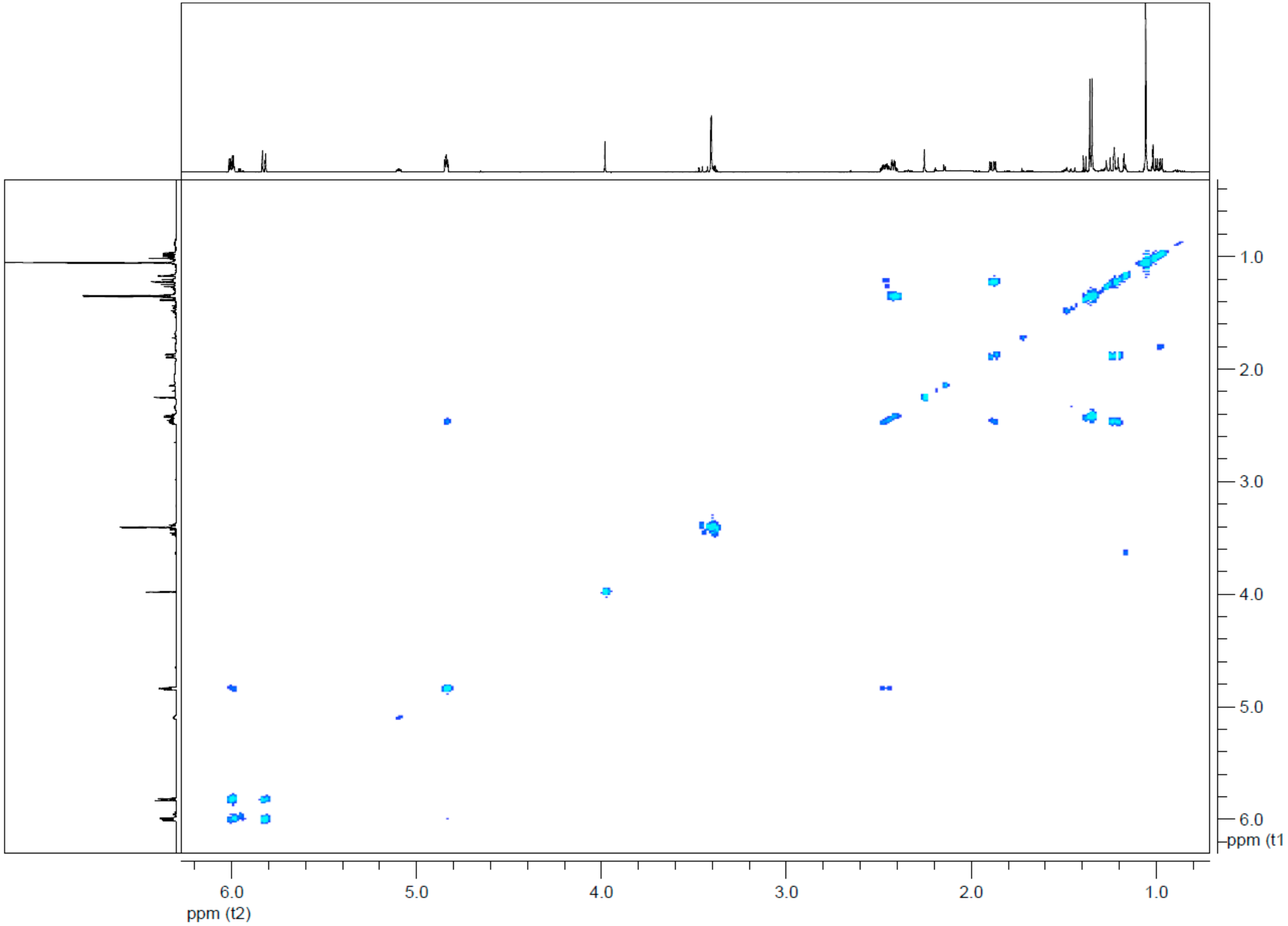

Figure S36. COSY (151 MHz, $CDCl_3$) spectrum of hydroxylactone **8a**.

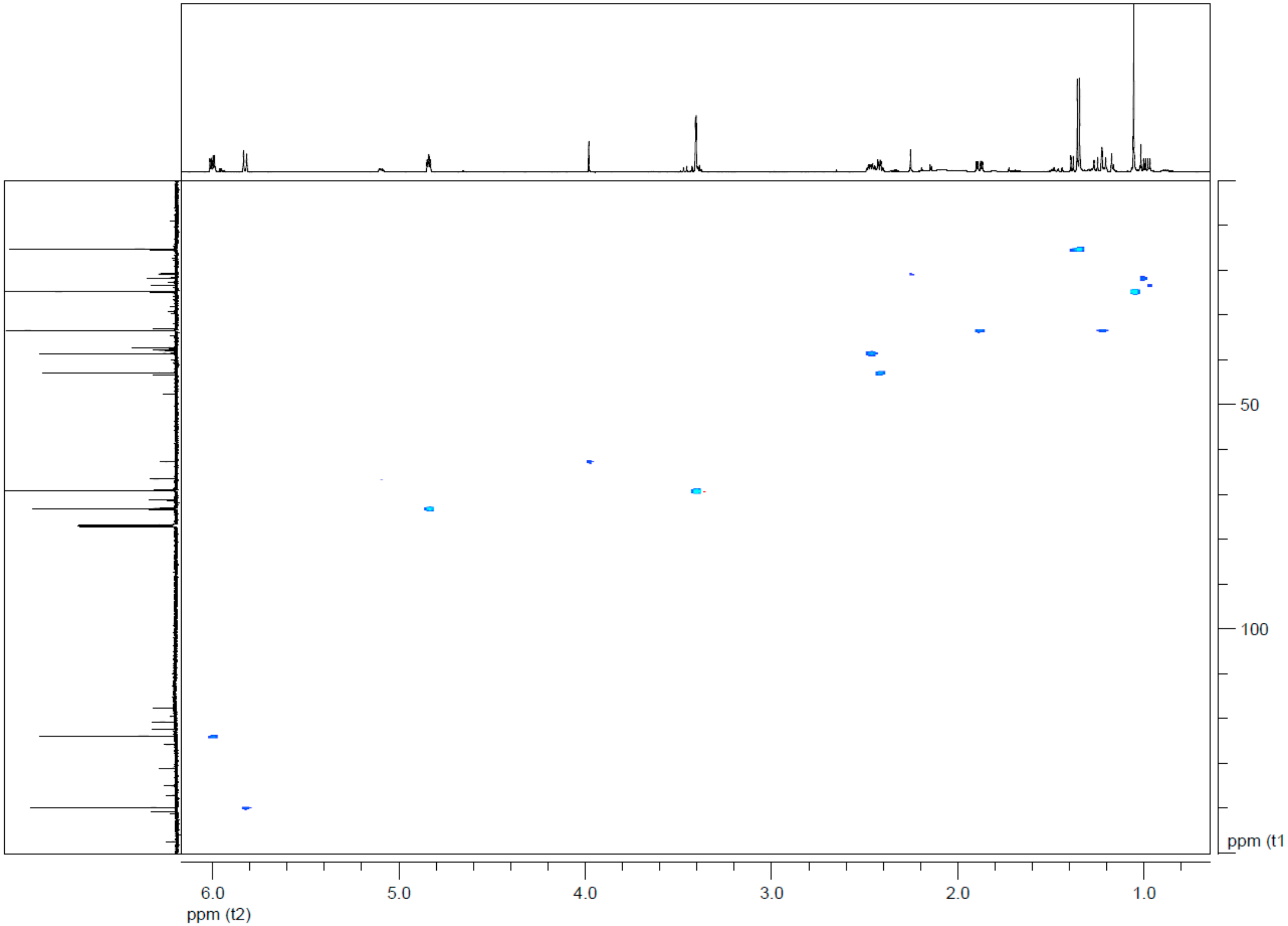

Figure S37. HMQC (151 MHz, $CDCl_3$) spectrum of hydroxylactone **8a**.

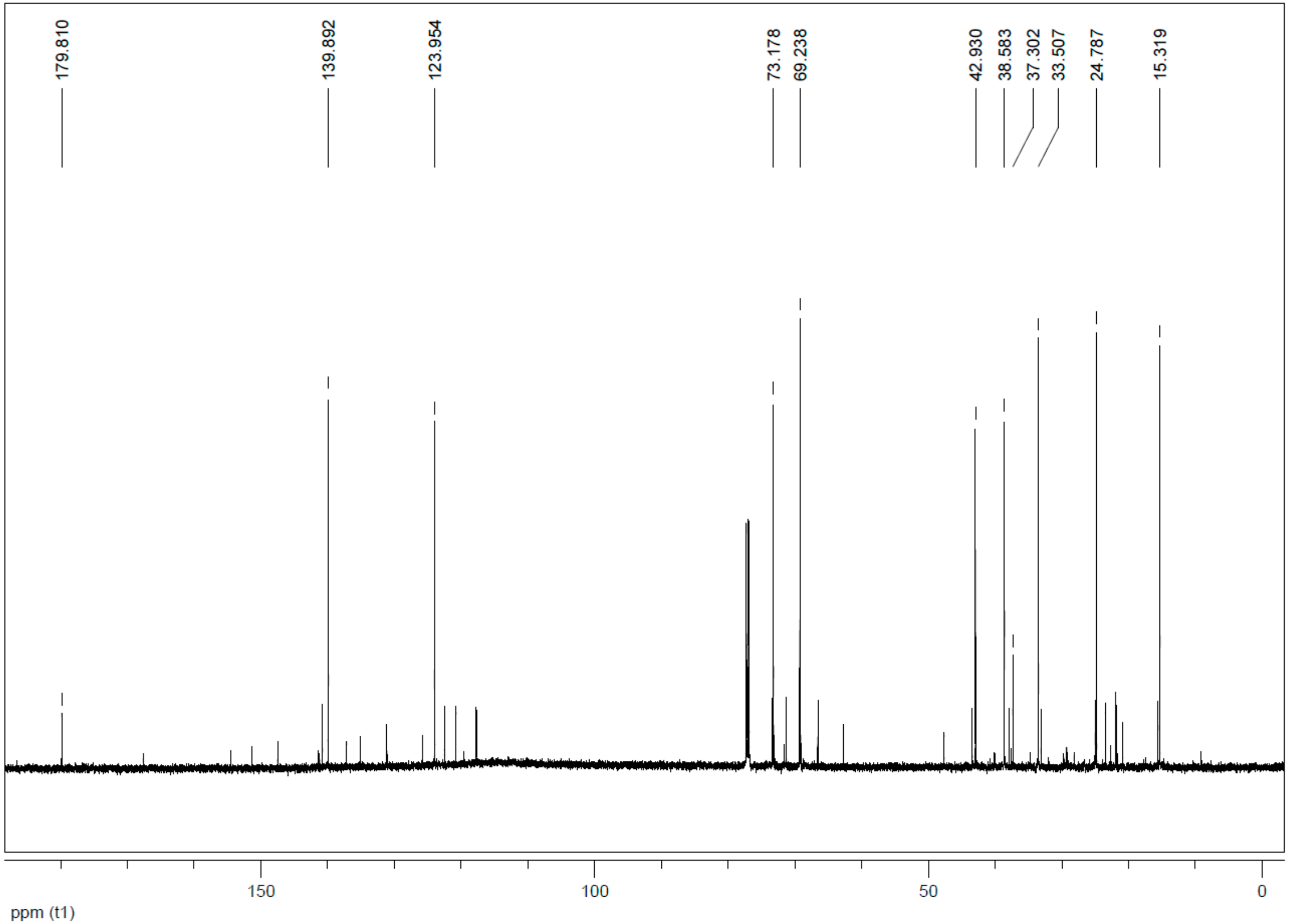

Figure S38. ^{13}C-NMR (151 MHz, $CDCl_3$) spectrum of hydroxylactone **8a**.

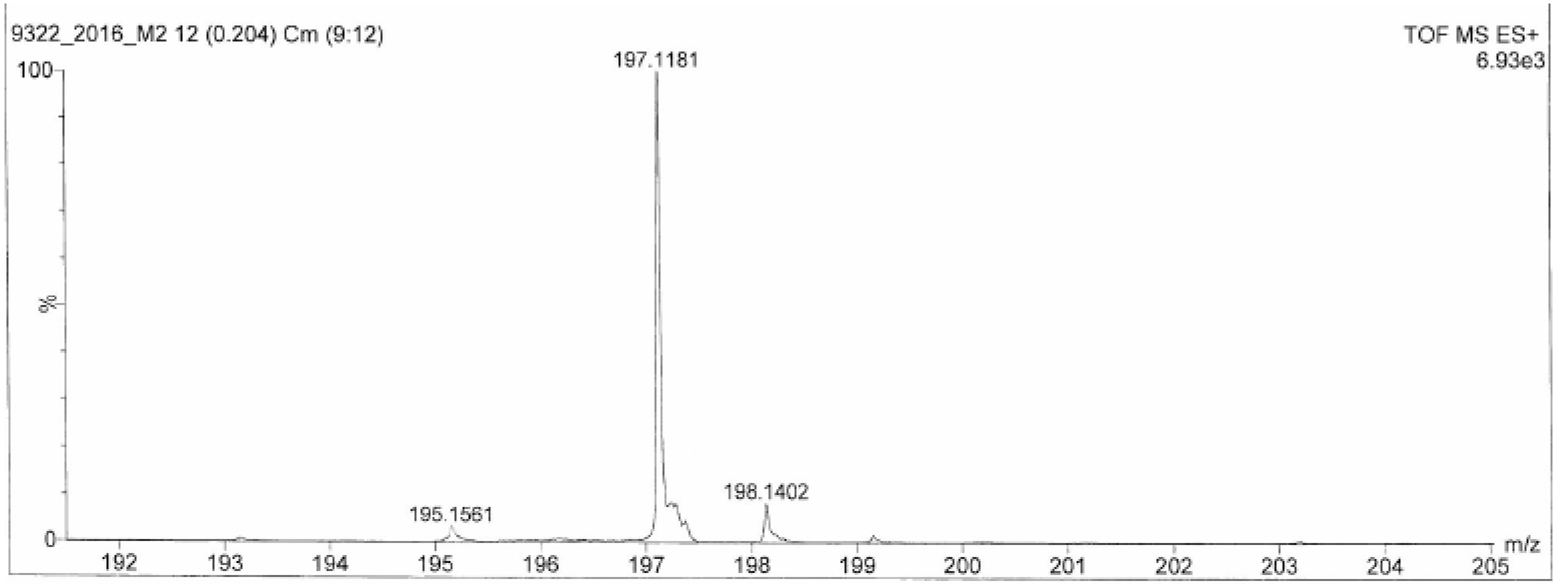

Figure S39. HRMS spectrum of hydroxylactone **8a**.

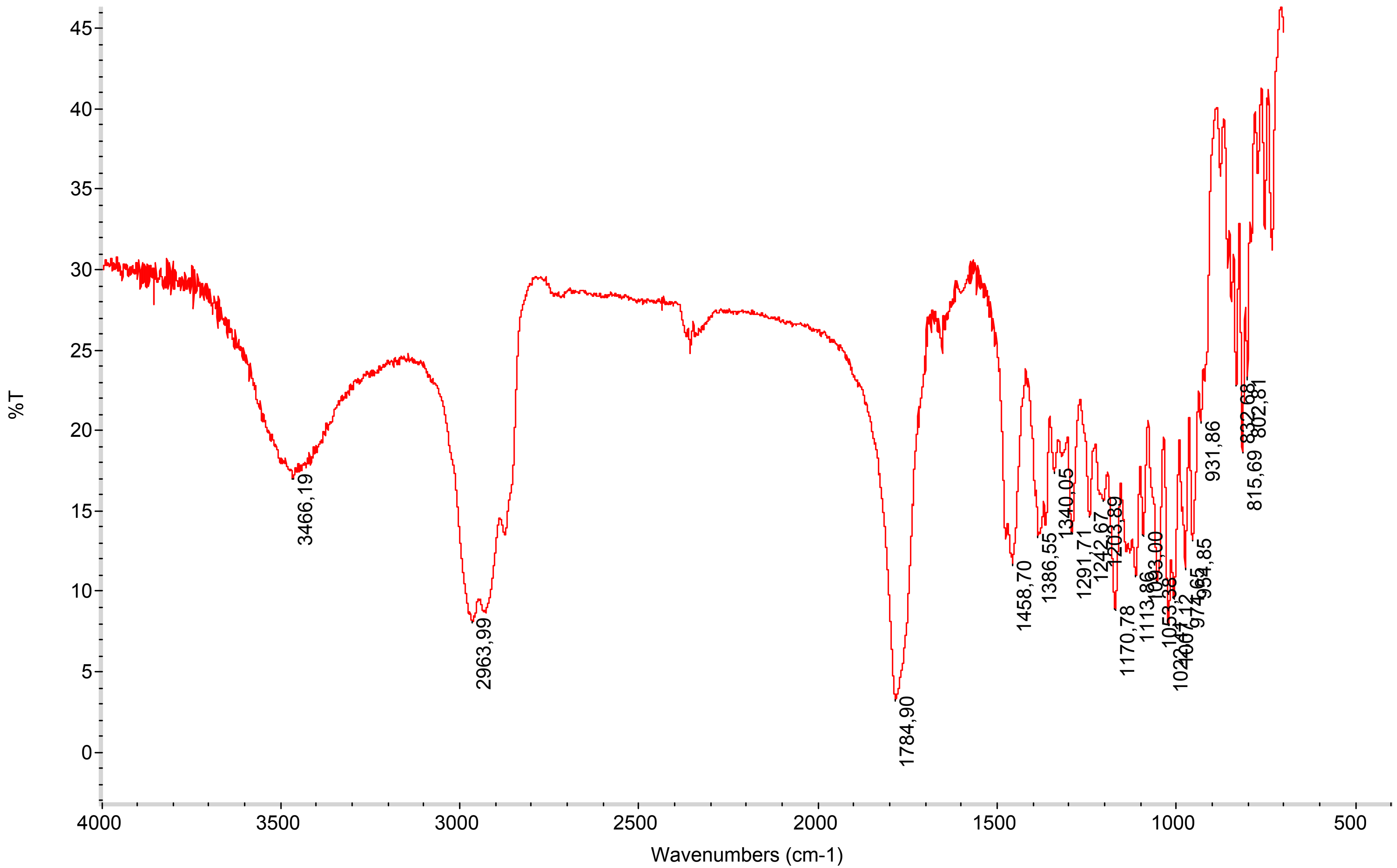

Figure S40. IR spectrum of hydroxylactone **8a**.

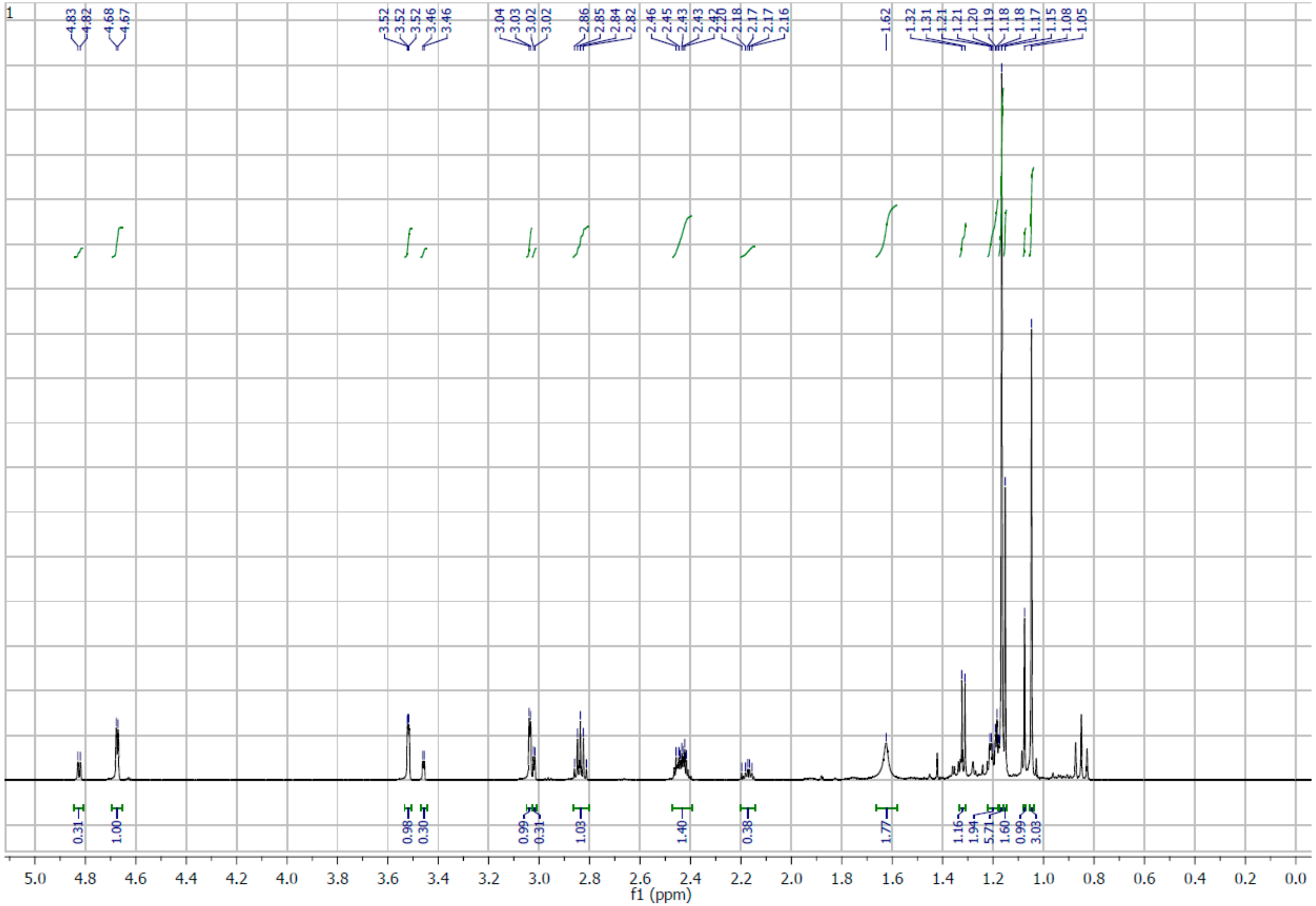

Figure S41. ^{1}H-NMR (600 MHz, $CDCl_3$) spectrum of epoxylactone **9a**.

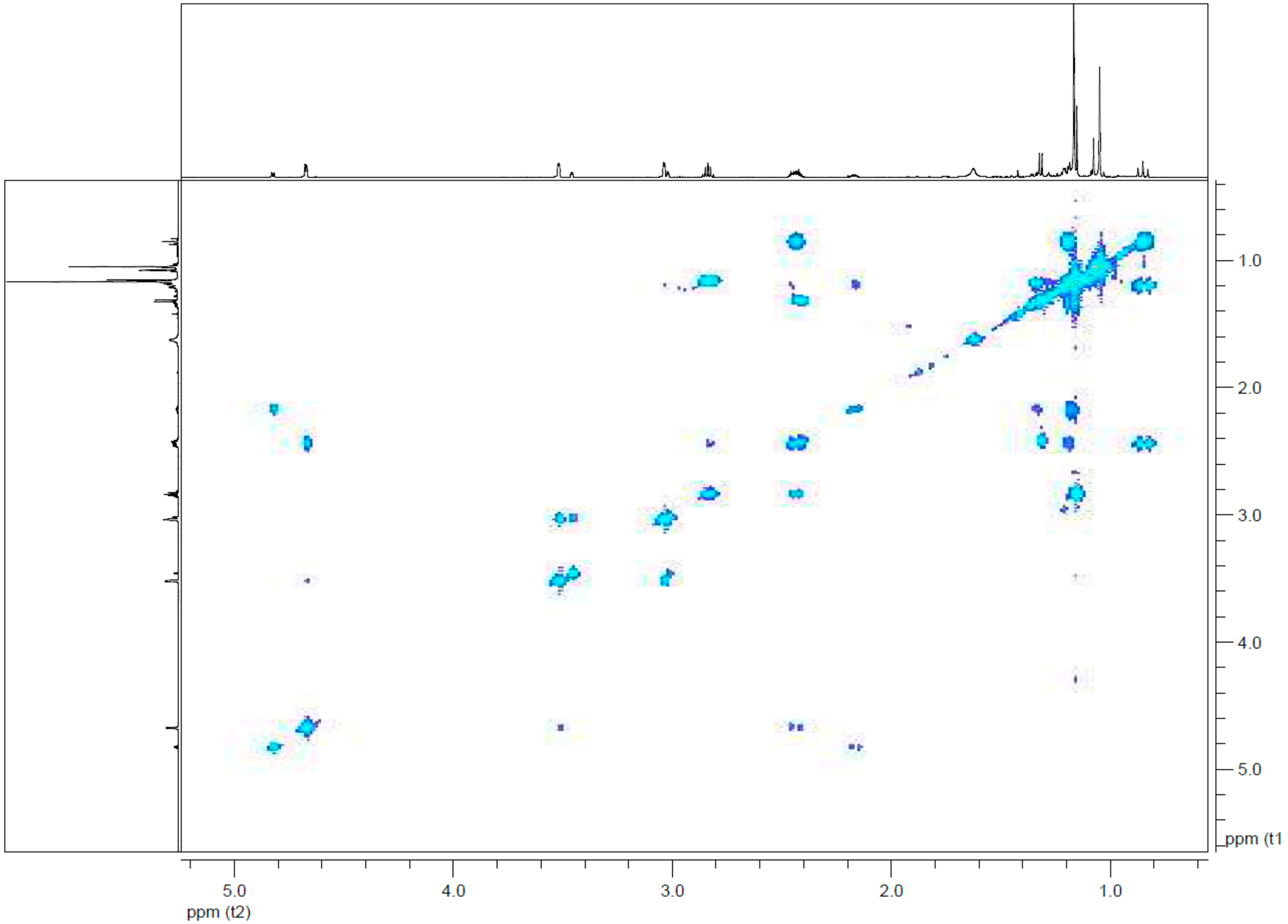

Figure S42. COSY (151 MHz, $CDCl_3$) spectrum of epoxylactone **9a**.

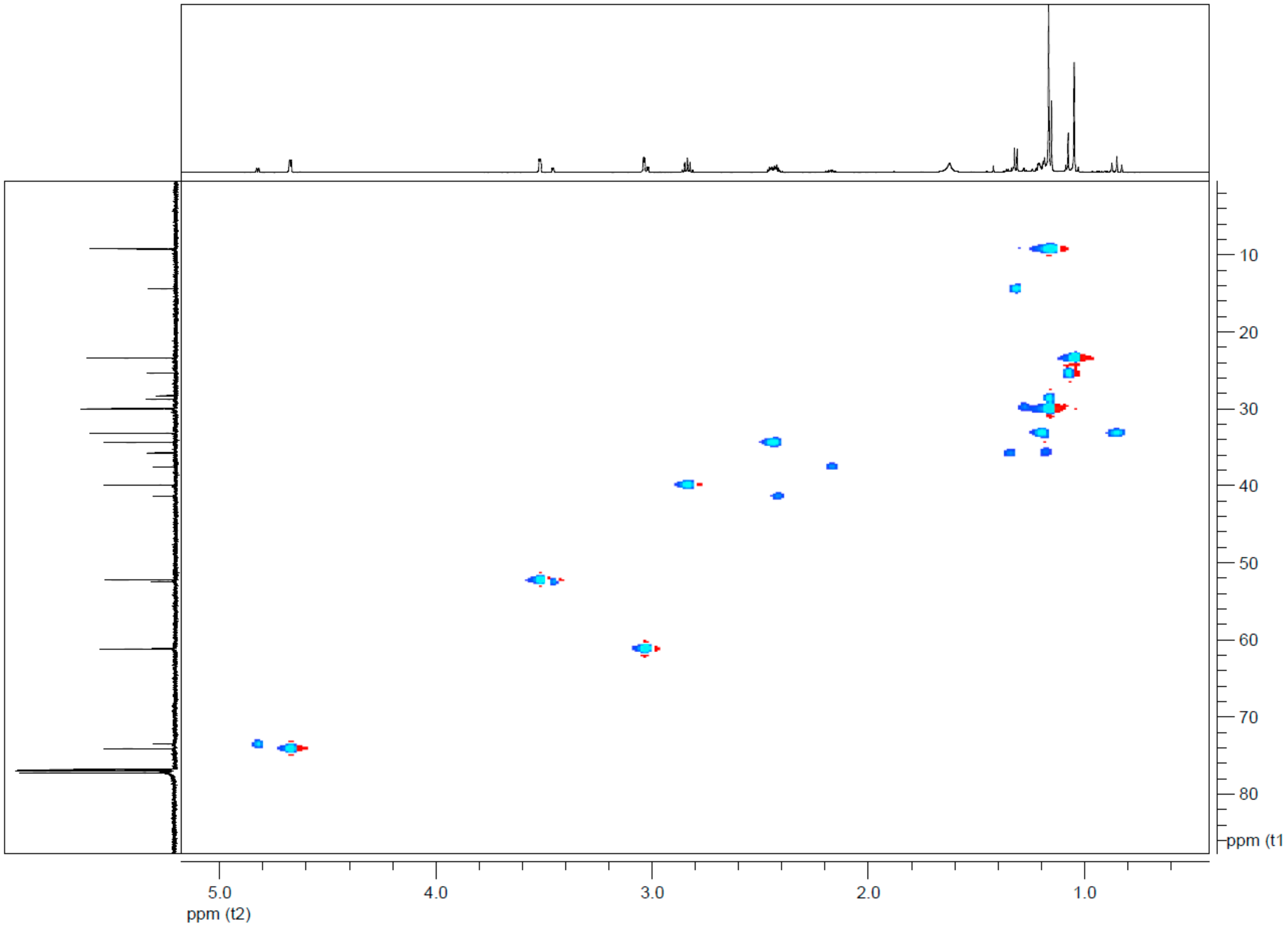

Figure S43. HMQC (151 MHz, $CDCl_3$) spectrum of epoxylactone **9a**.

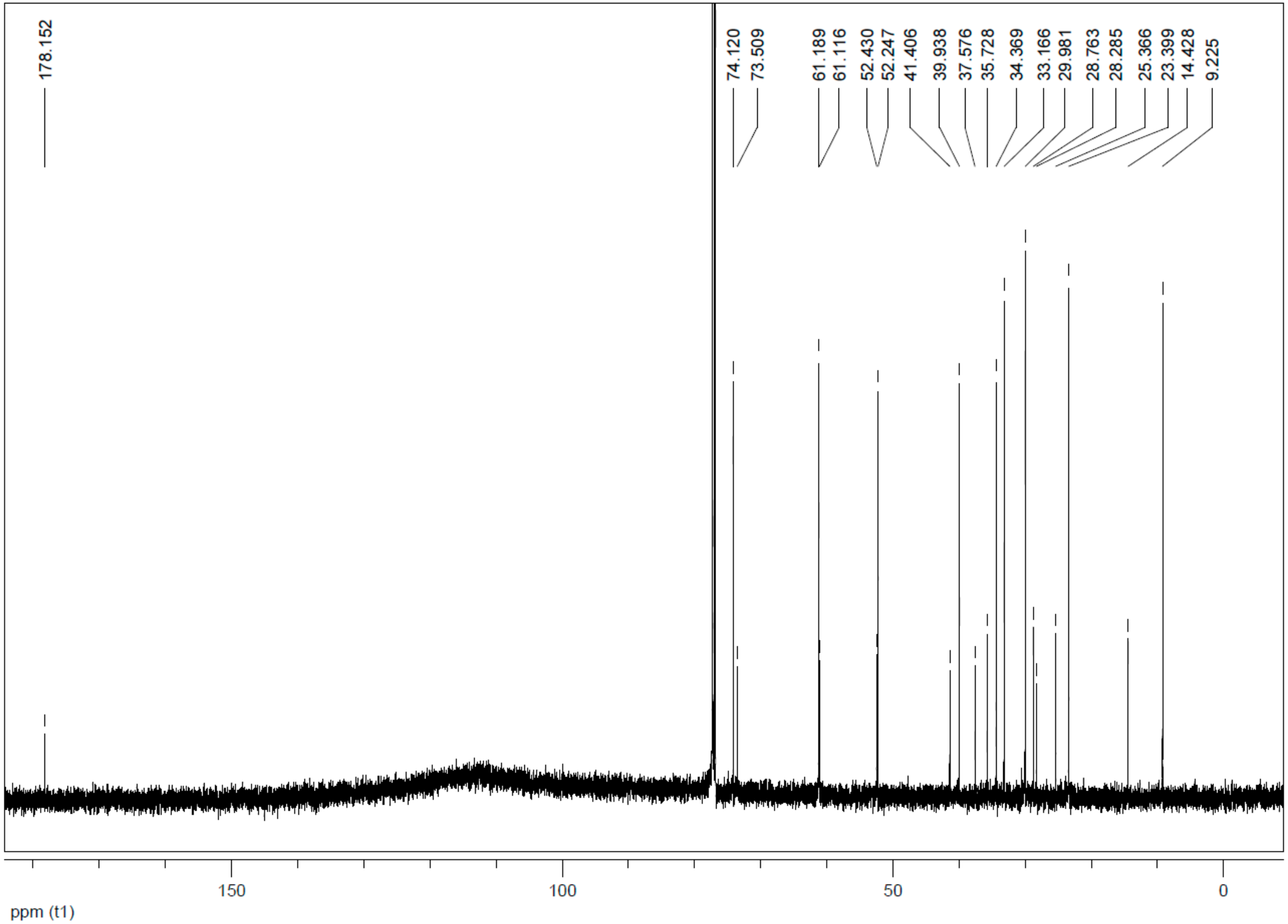

Figure S44. ^{13}C-NMR (151 MHz, $CDCl_3$) spectrum of epoxylactone **9a**.

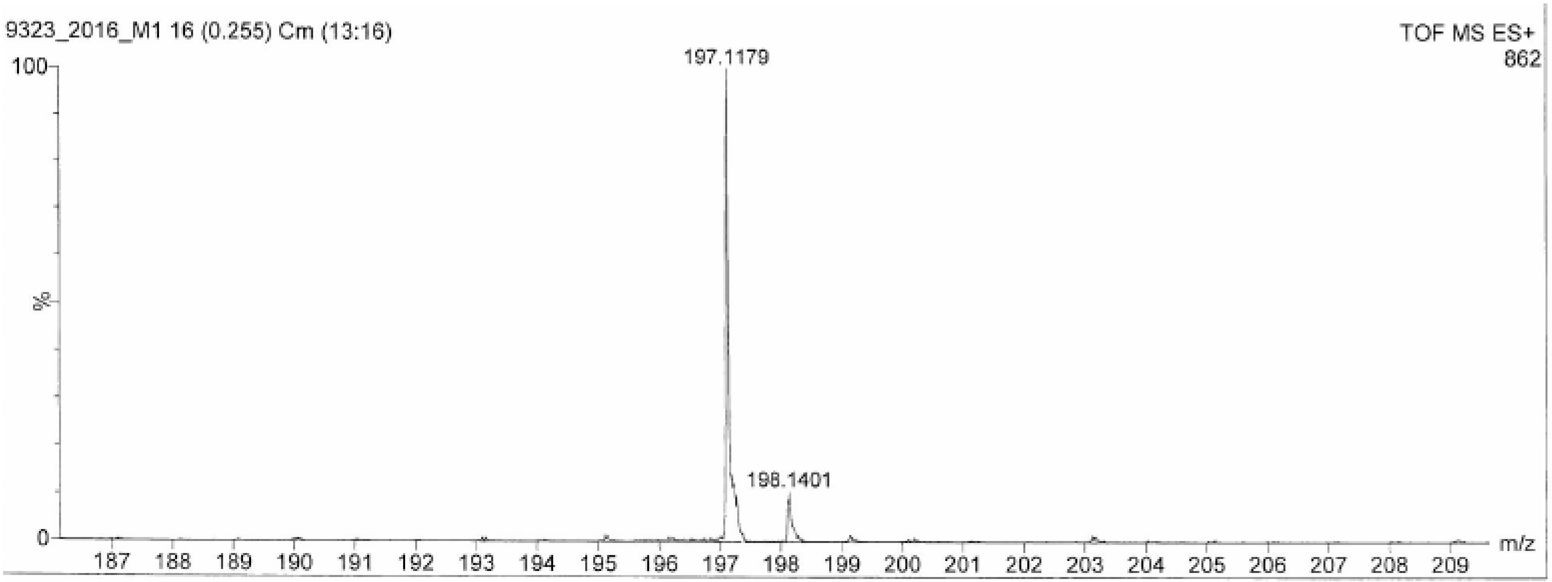

Figure S45. HRMS spectrum of epoxylactone **9a**.

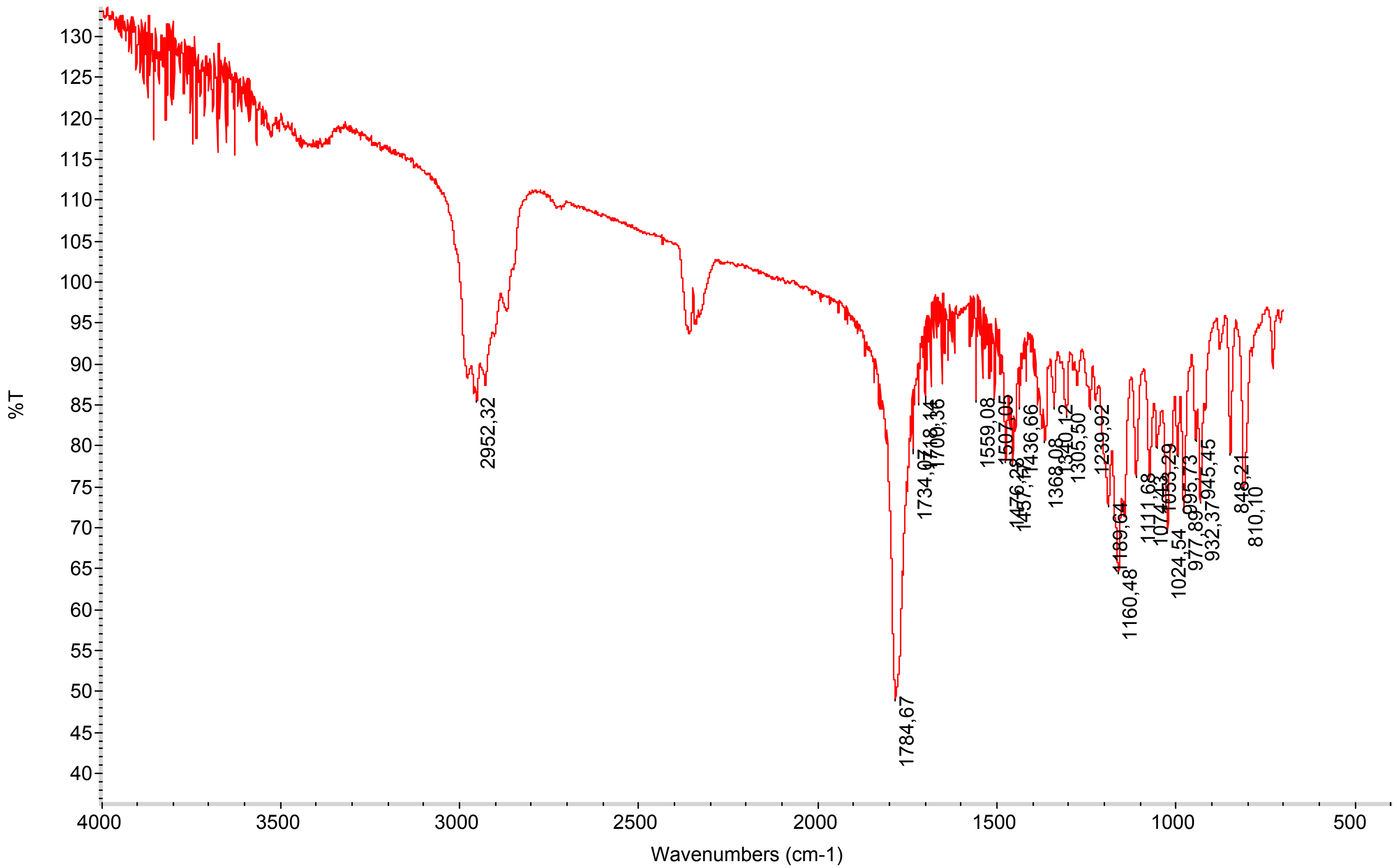

Figure S46. IR spectrum of epoxylactone **9a**.

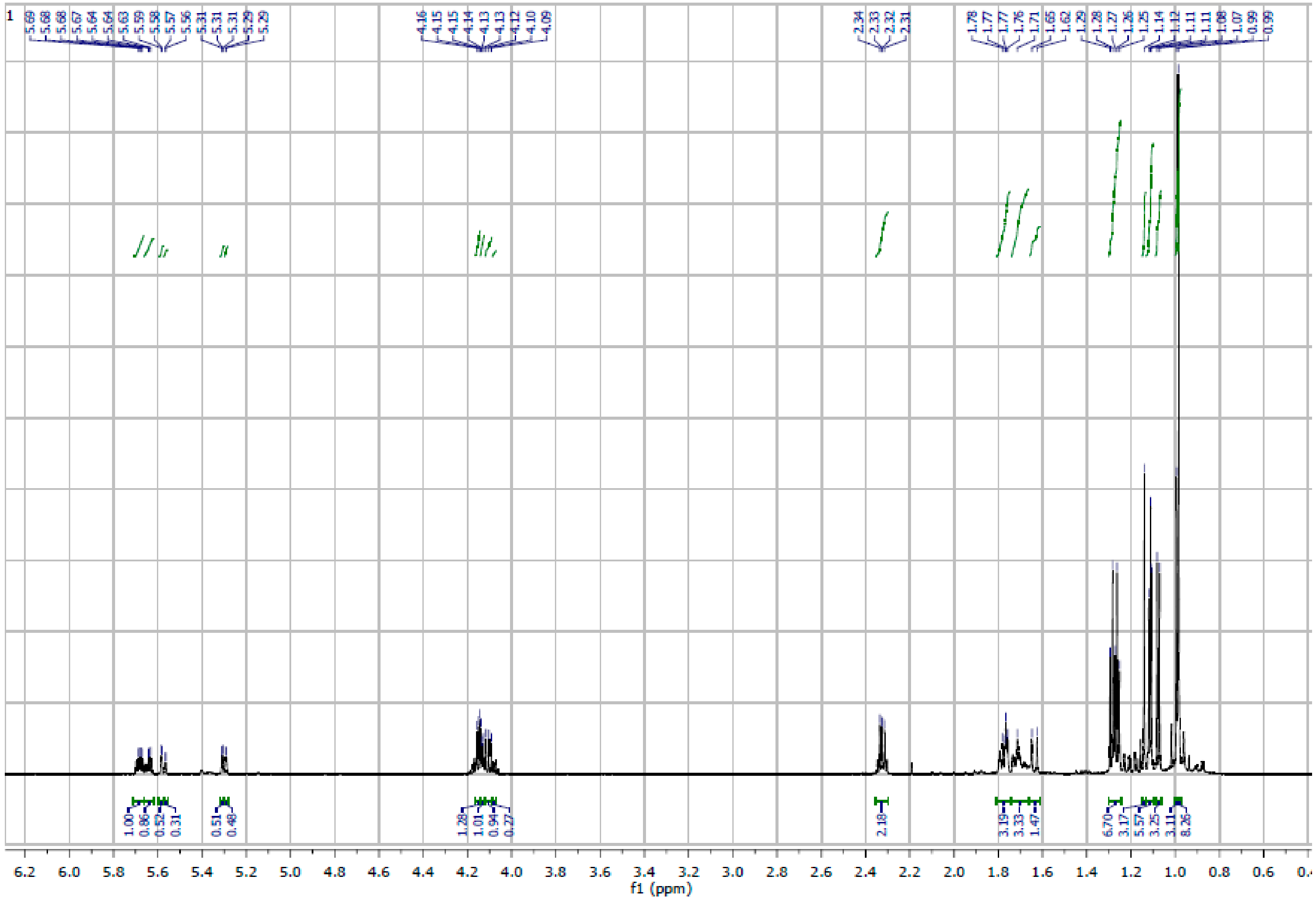

Figure S47. ^{1}H-NMR (600 MHz, $CDCl_3$) spectrum of ester **2b**.

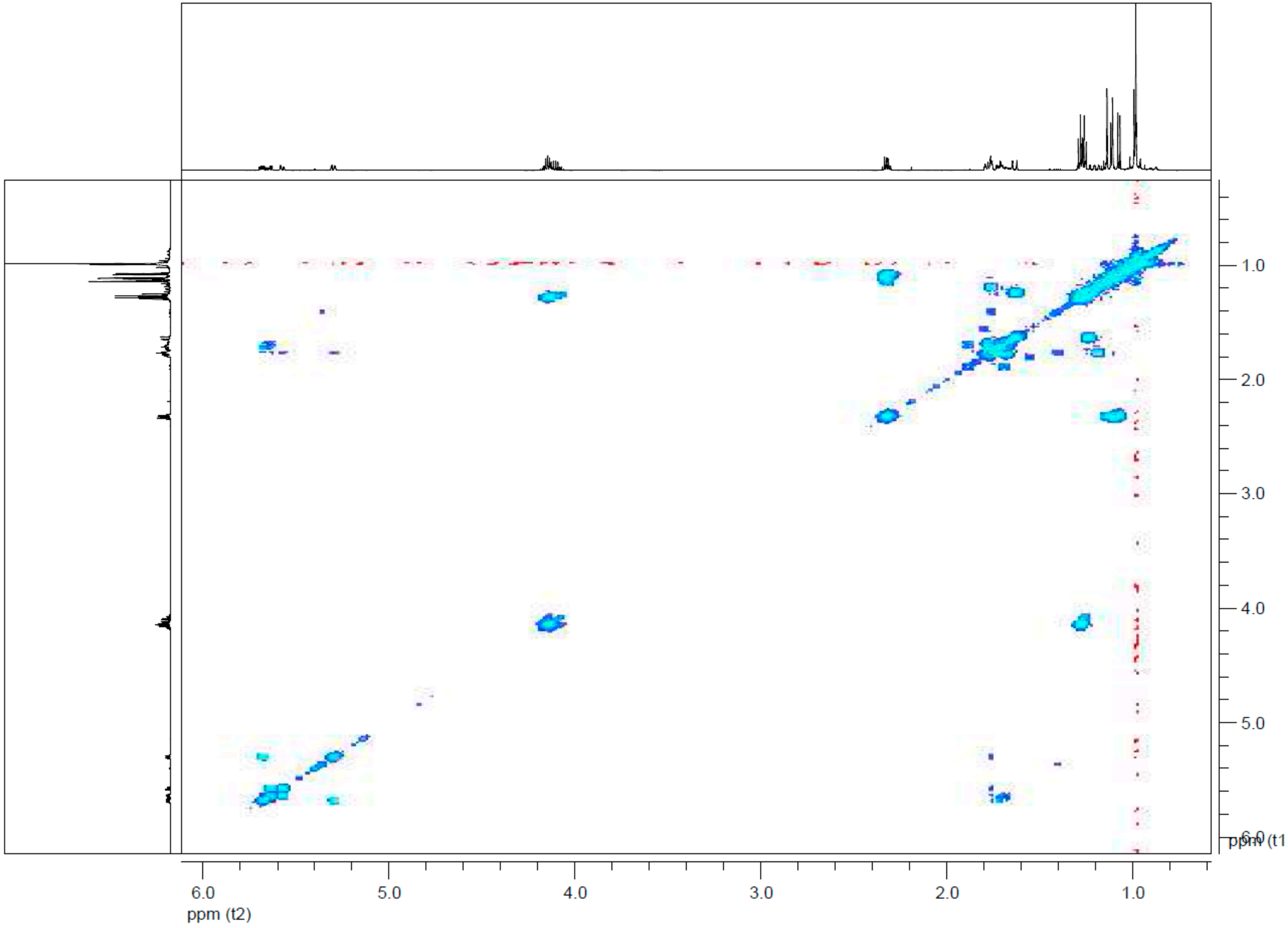

Figure S48. COSY (151 MHz, CDCl3) spectrum of ester **2b**.

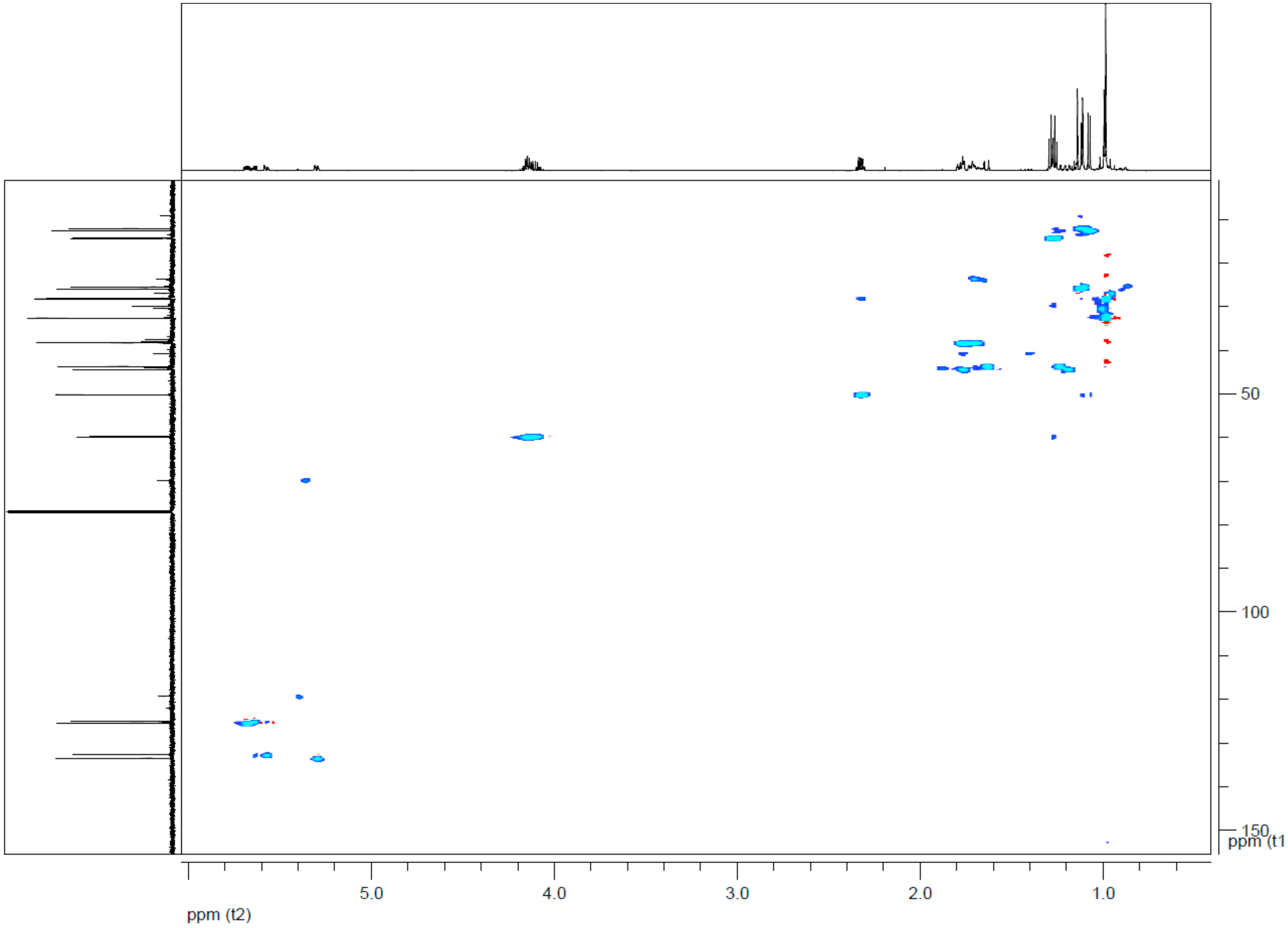

Figure S49. HMQC (151 MHz, $CDCl_3$) spectrum of ester **2b**.

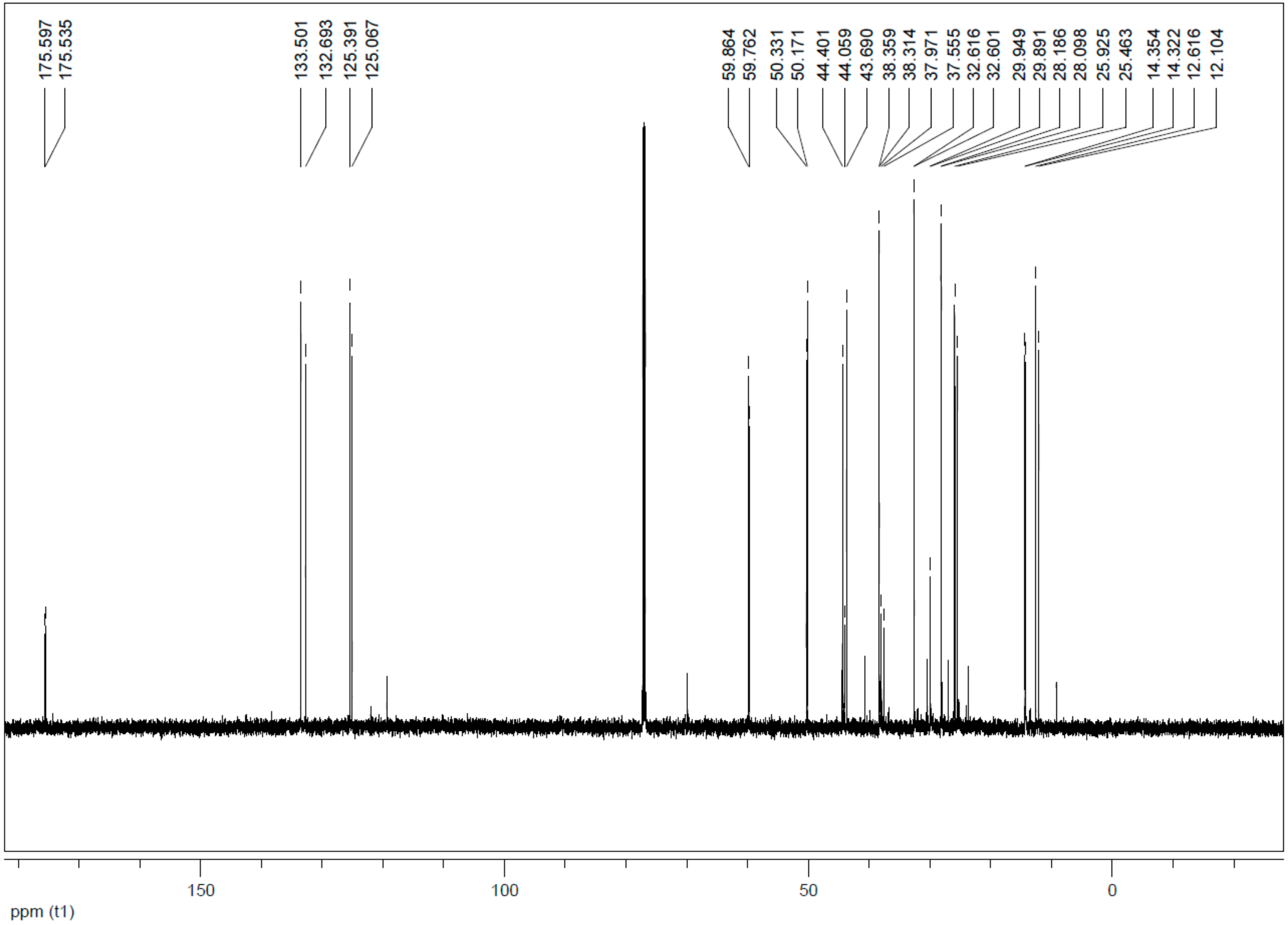

Figure S50. ^{13}C-NMR (151 MHz, $CDCl_3$) spectrum of ester **2b**.

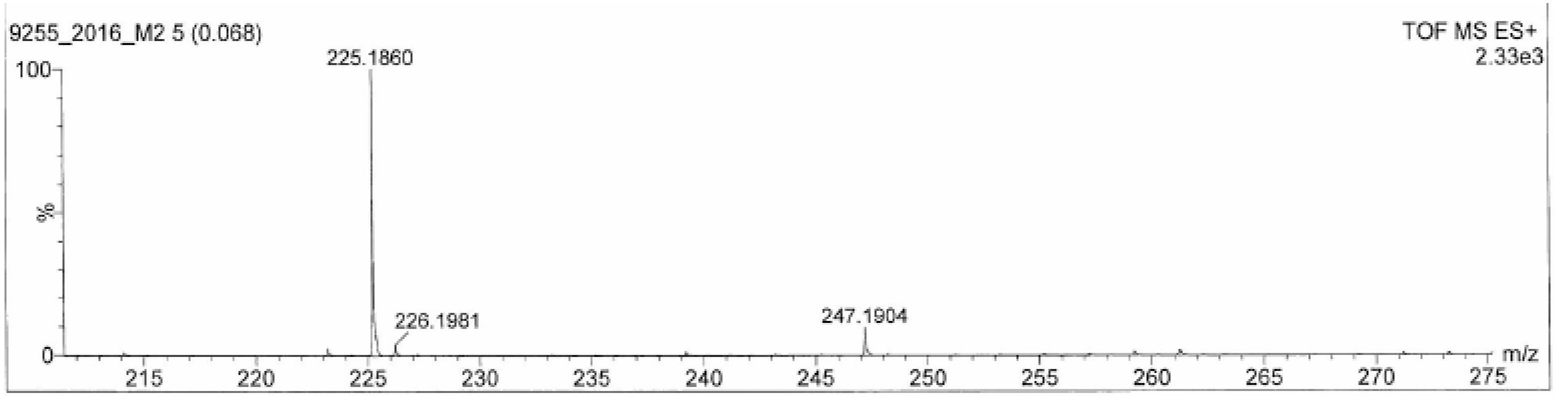

Figure S51. HRMS spectrum of ester **2b**.

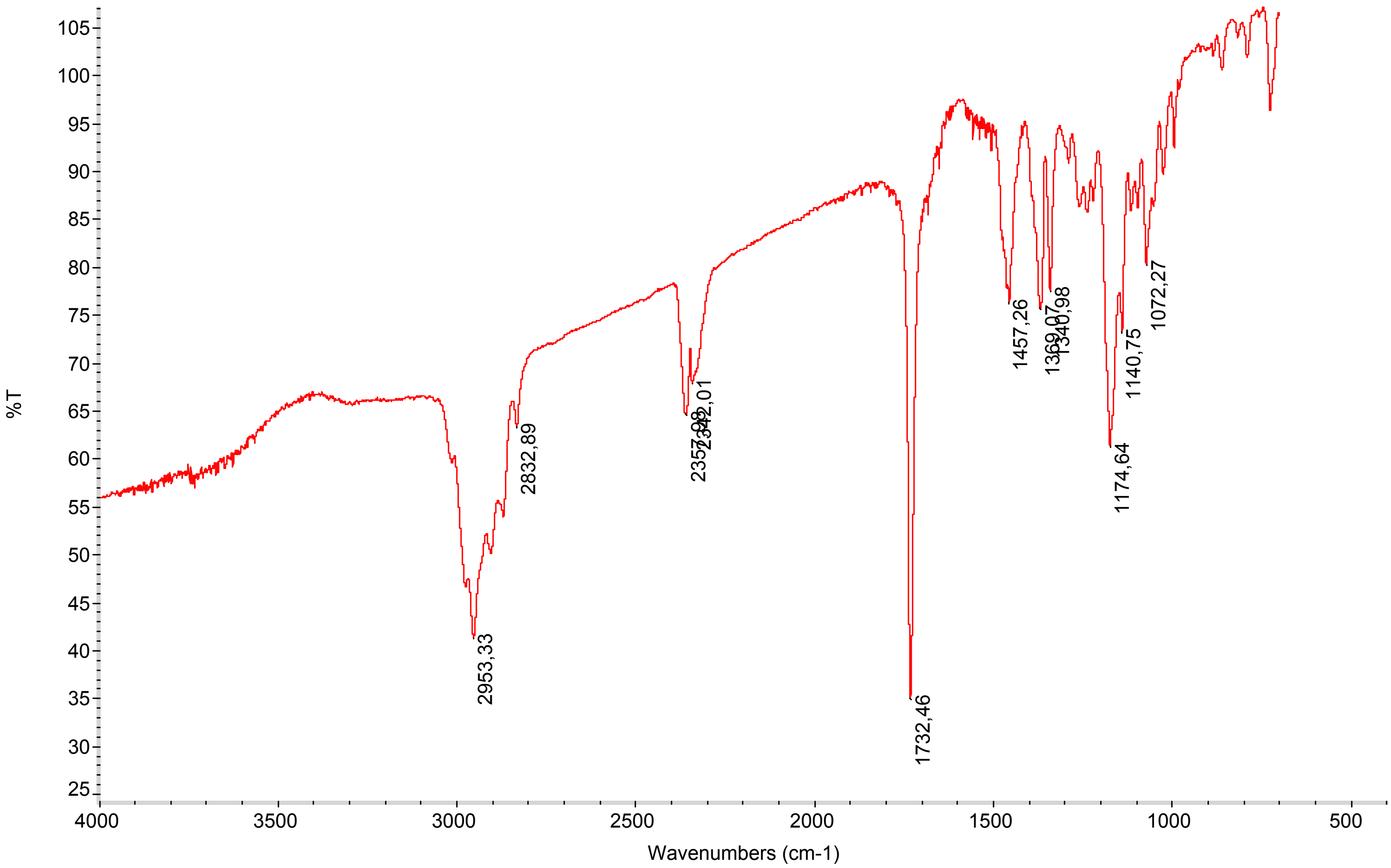

Figure S52. IR spectrum of ester **2b**.

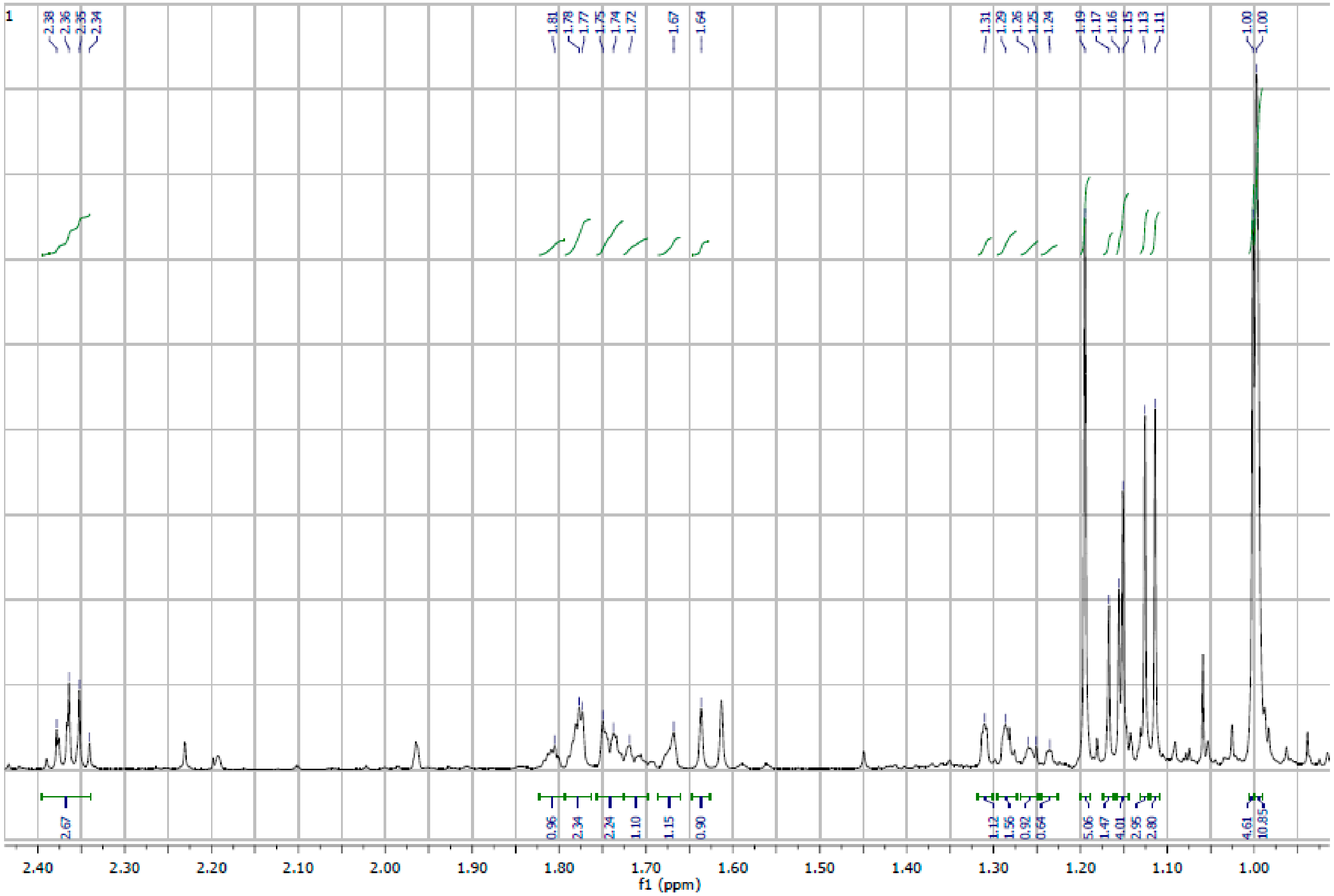

Figure S53. ^{1}H-NMR (600 MHz, $CDCl_3$) spectrum of acid **3b**.

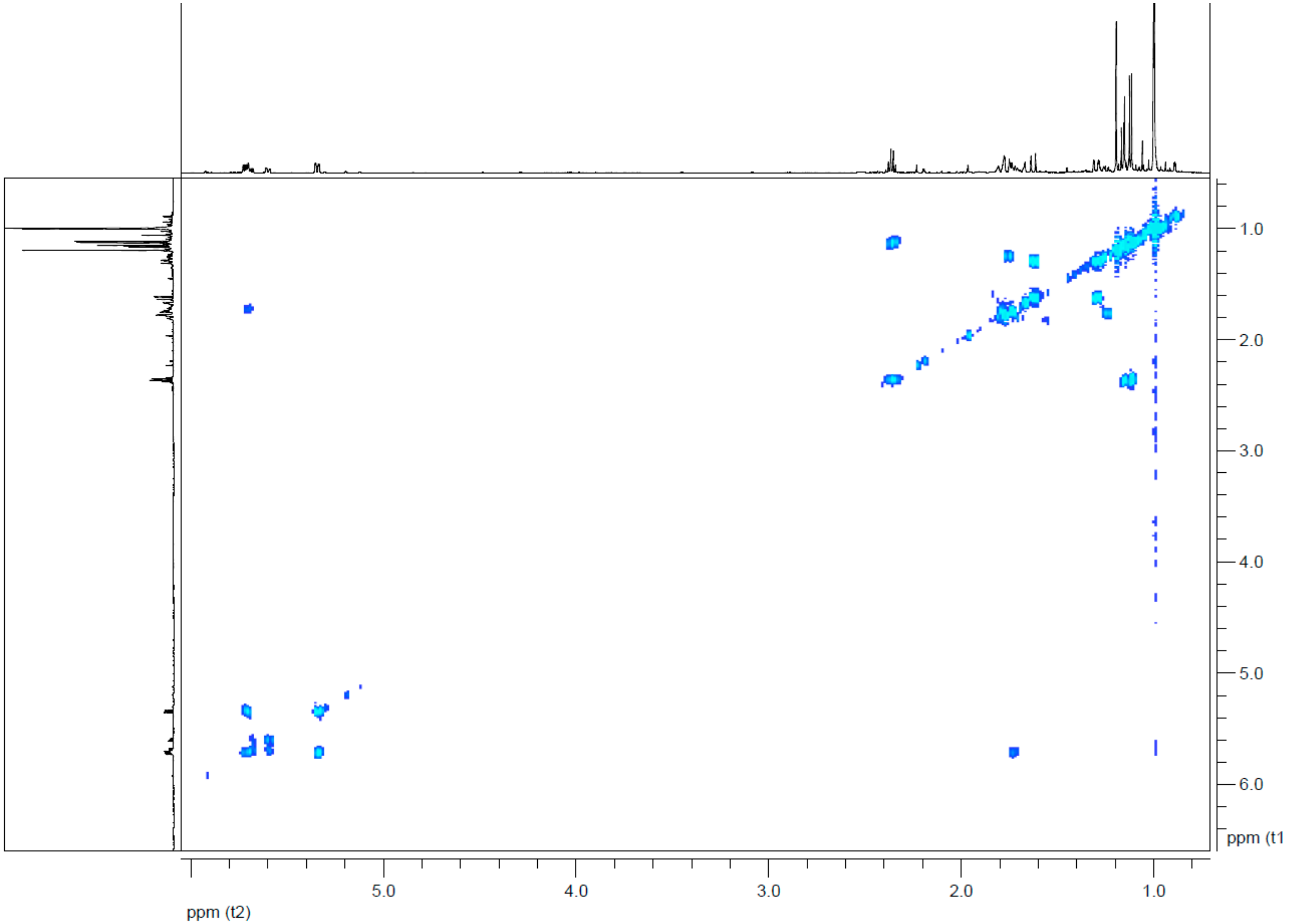

Figure S54. COSY (151 MHz, $CDCl_3$) spectrum of acid **3b**.

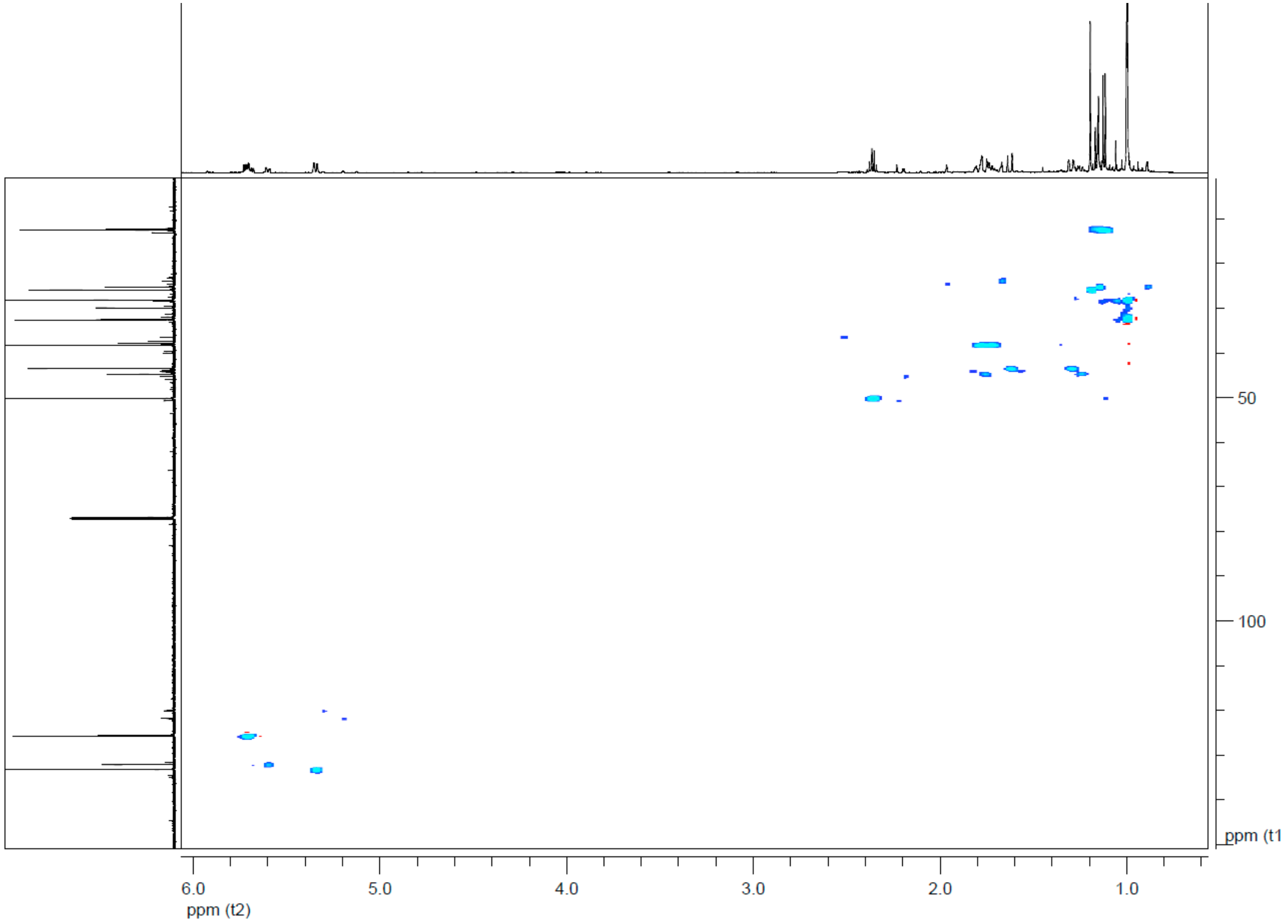

Figure S55. HMQC (151 MHz, $CDCl_3$) spectrum of acid **3b**.

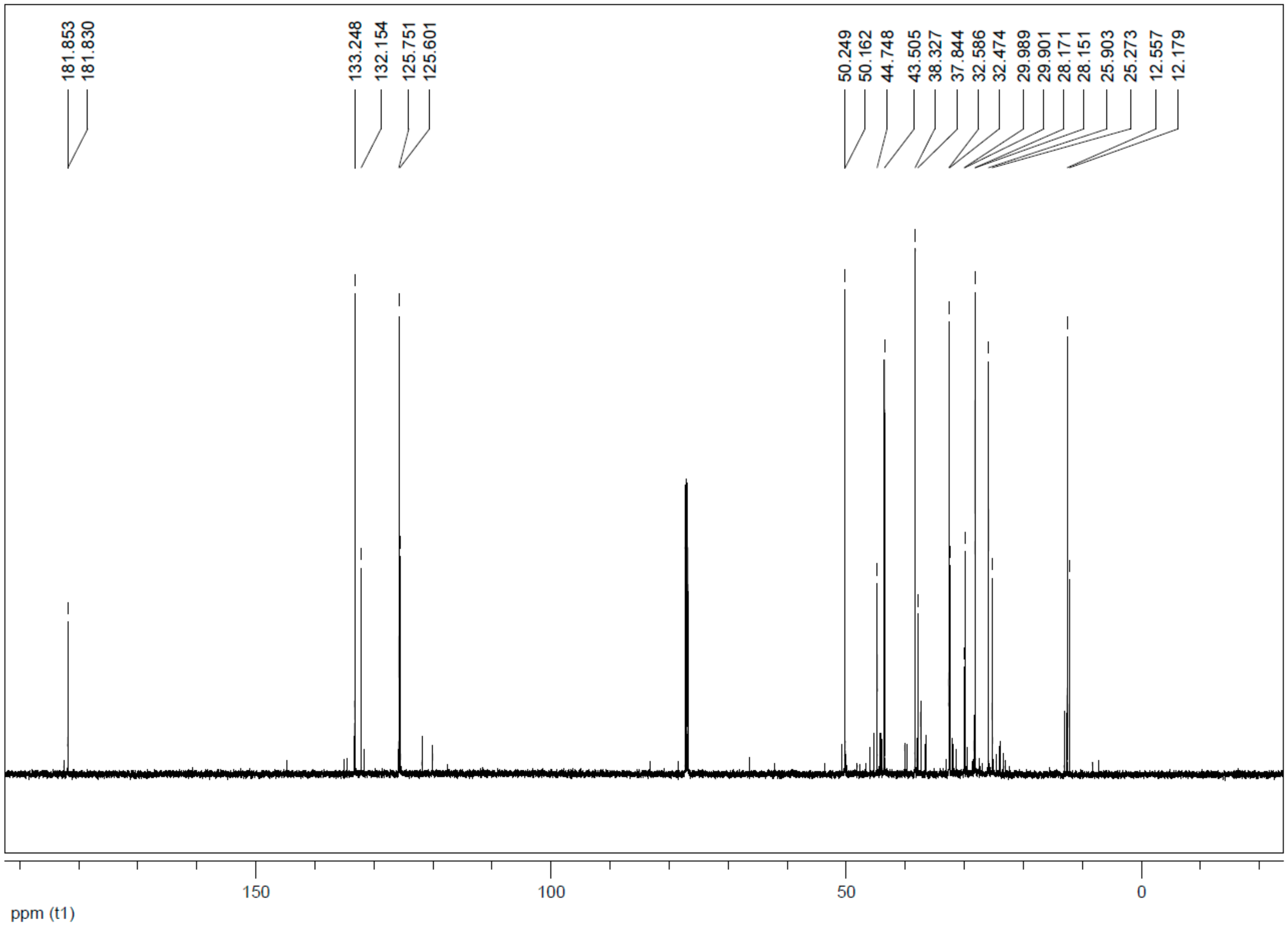

Figure S56. ^{13}C-NMR (151 MHz, $CDCl_3$) spectrum of acid **3b**.

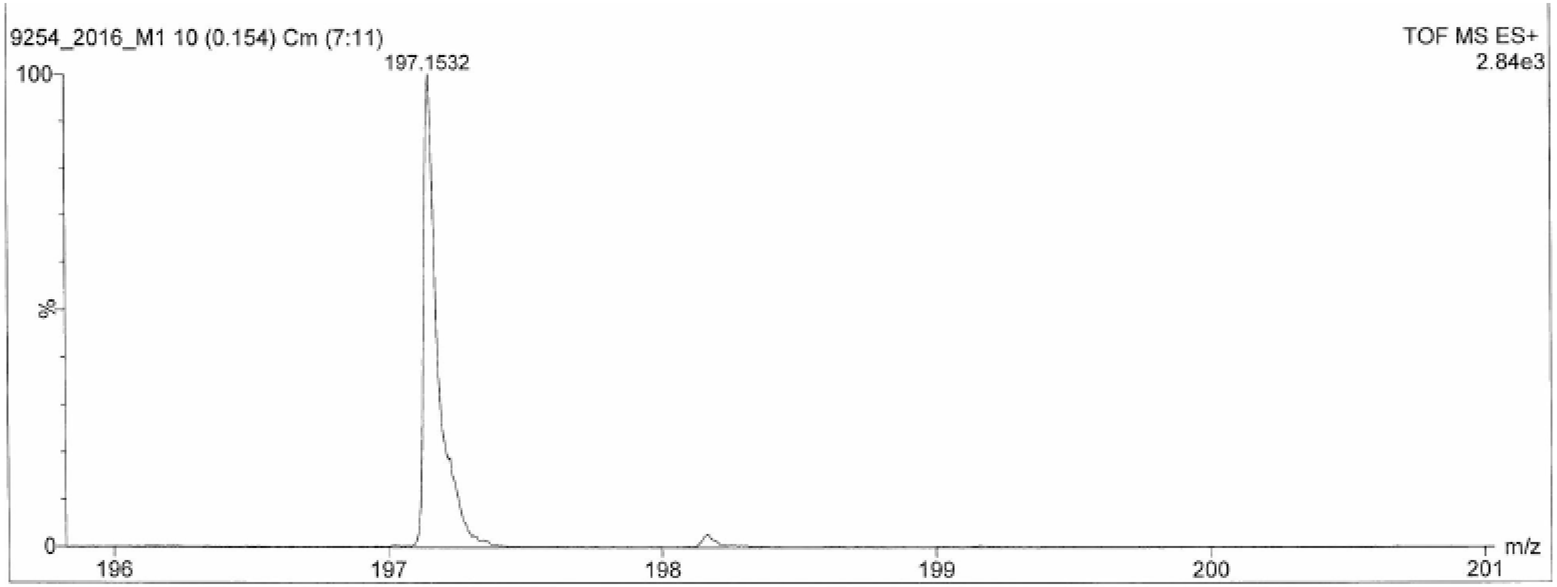

Figure S57. HRMS spectrum of acid **3b**.

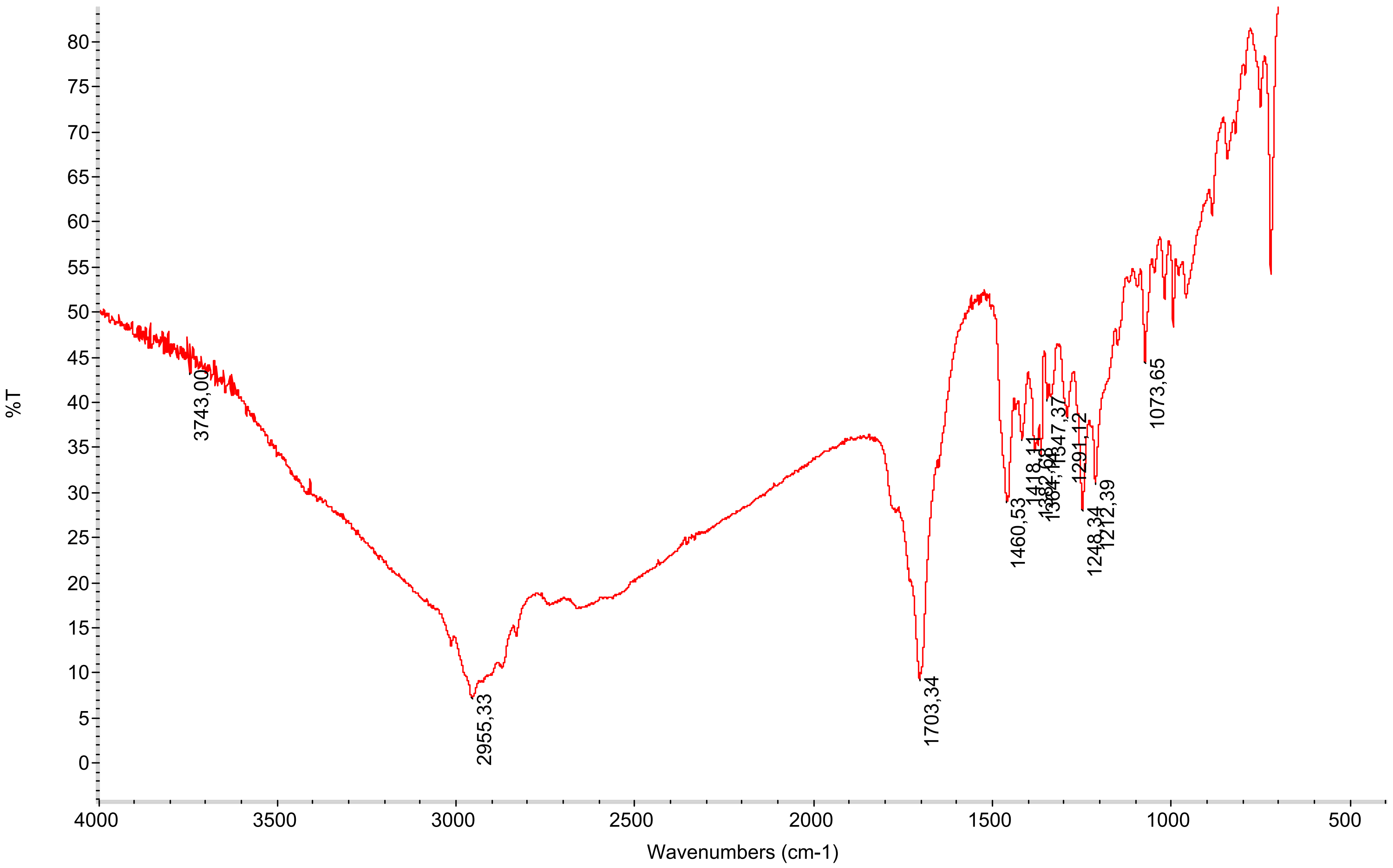

Figure S58. IR spectrum of acid **3b**.

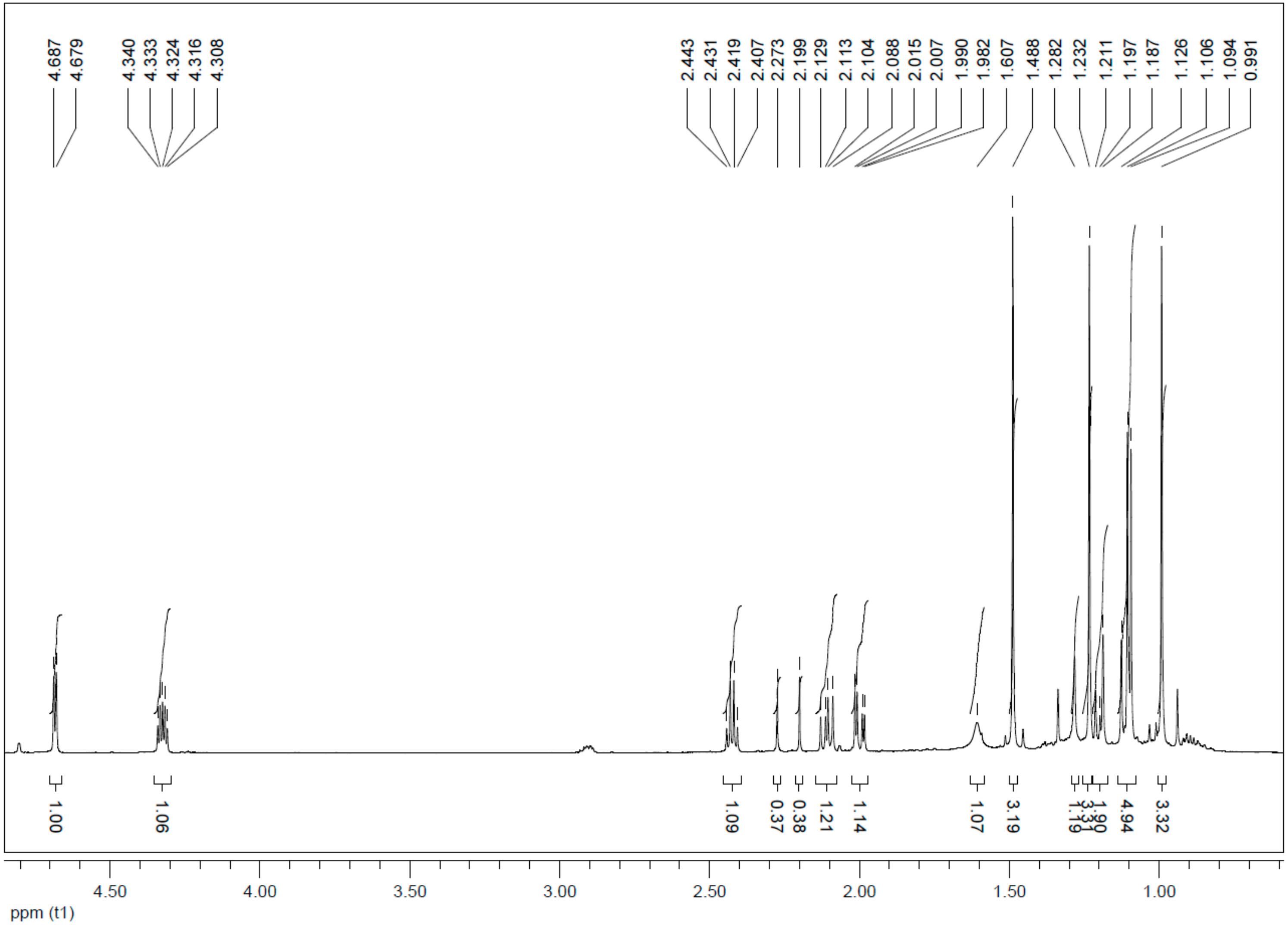

Figure S59. ^{1}H-NMR (600 MHz, $CDCl_3$) spectrum of iodolactone **4b-A**.

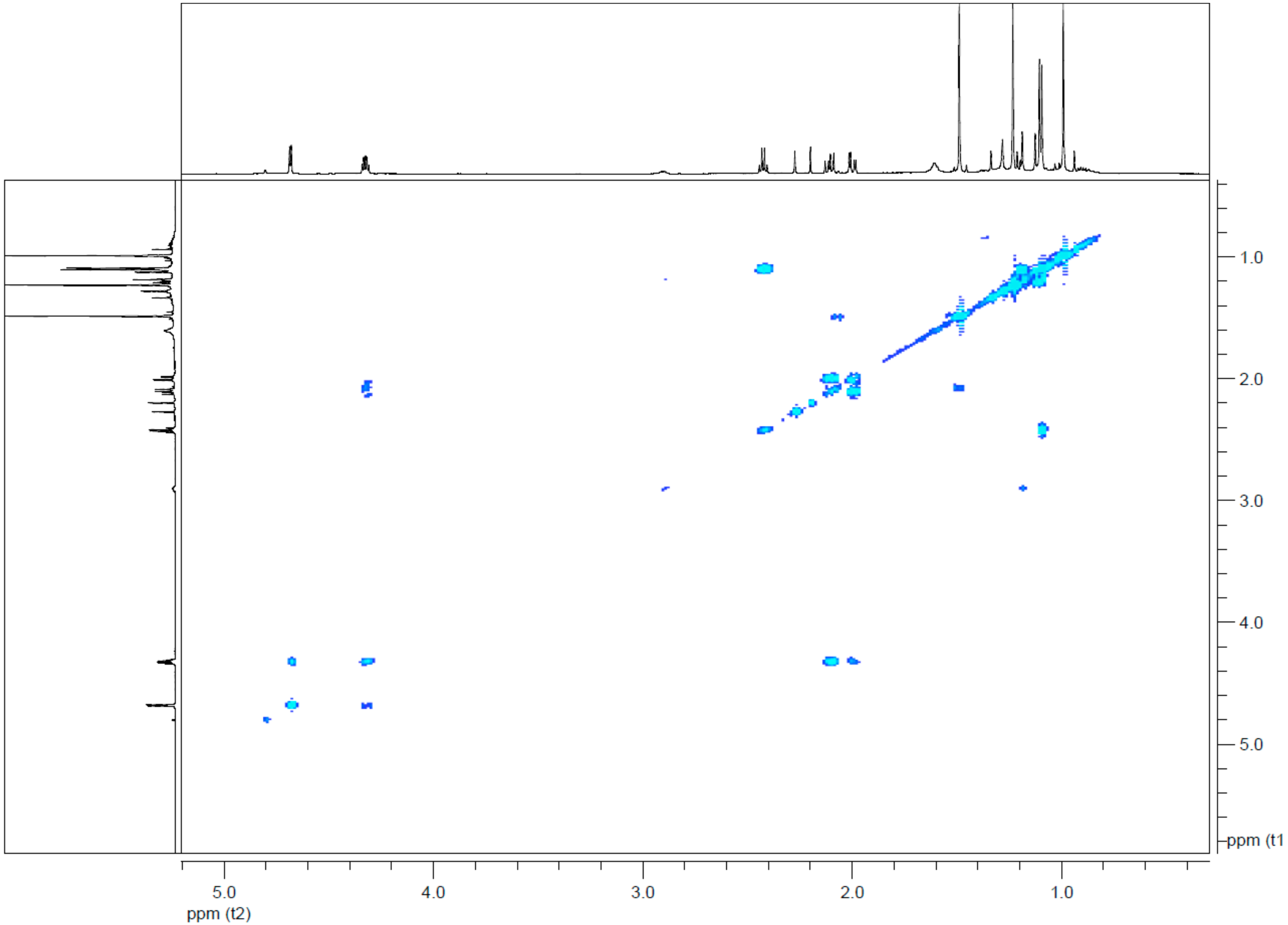

Figure S60. COSY (151 MHz, $CDCl_3$) spectrum of iodolactone **4b-A**.

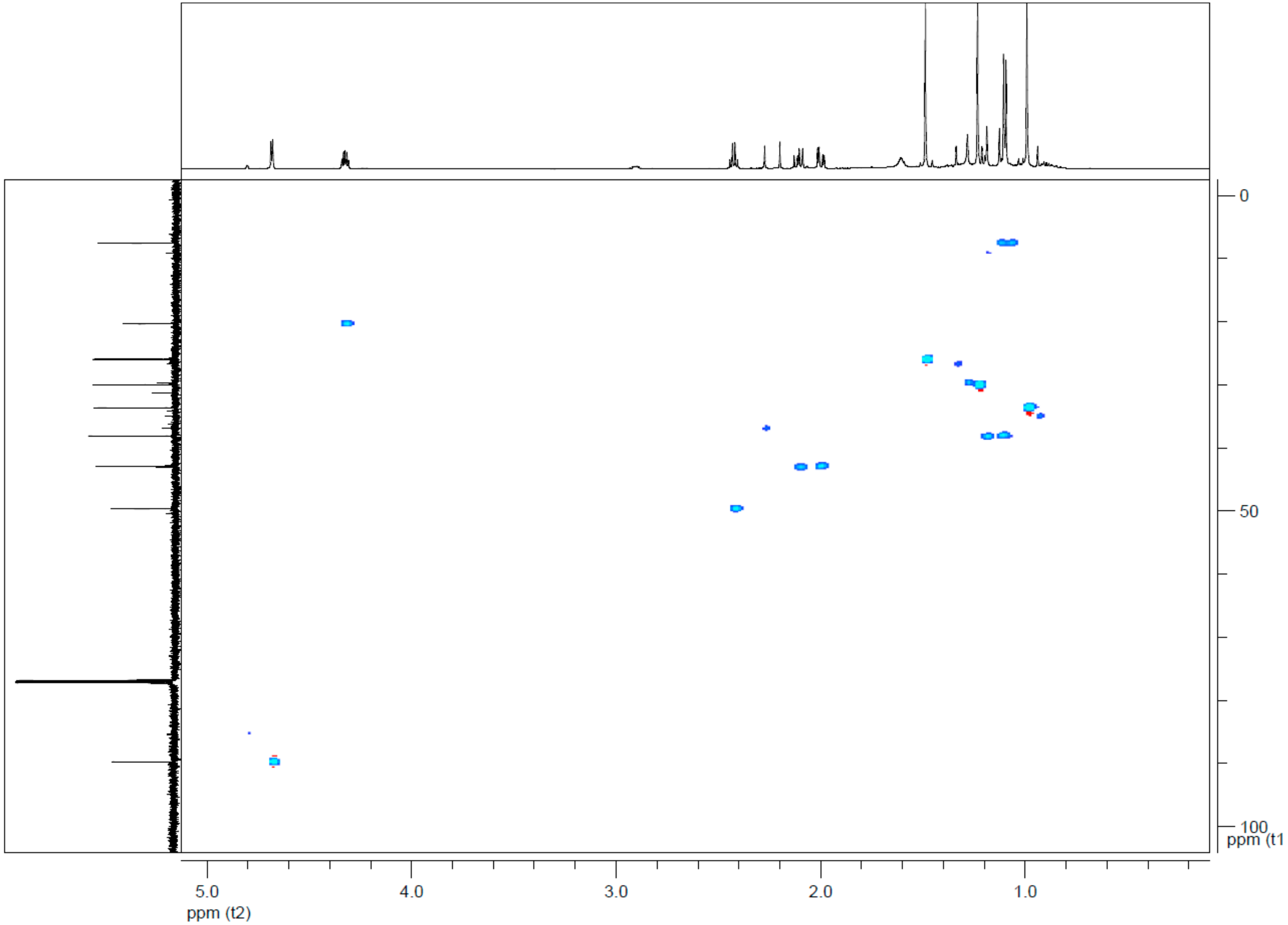

Figure S61. HMQC (151 MHz, $CDCl_3$) spectrum of iodolactone **4b-A**.

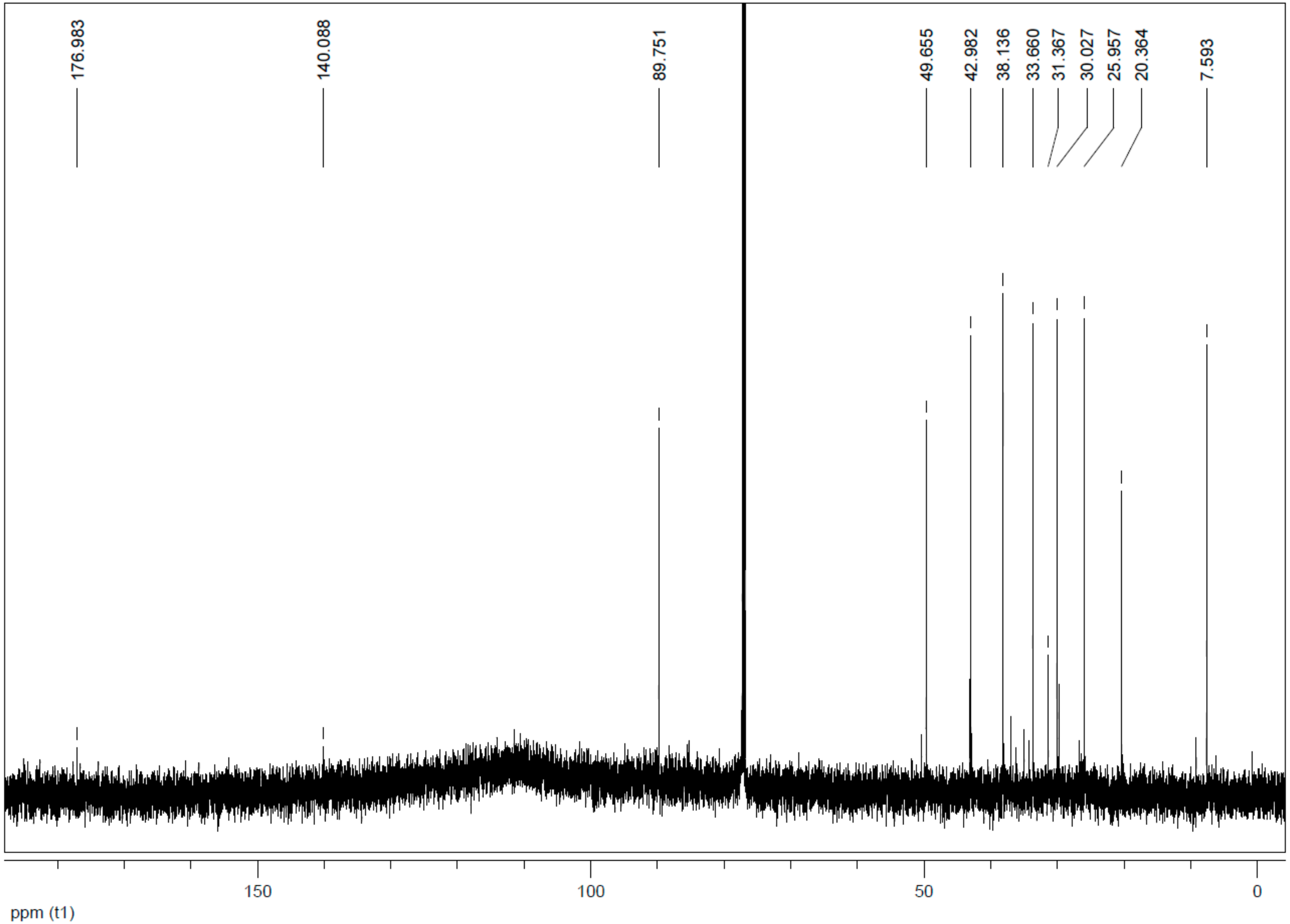

Figure S62. ^{13}C-NMR (151 MHz, $CDCl_3$) spectrum of iodolactone **4b-A**.

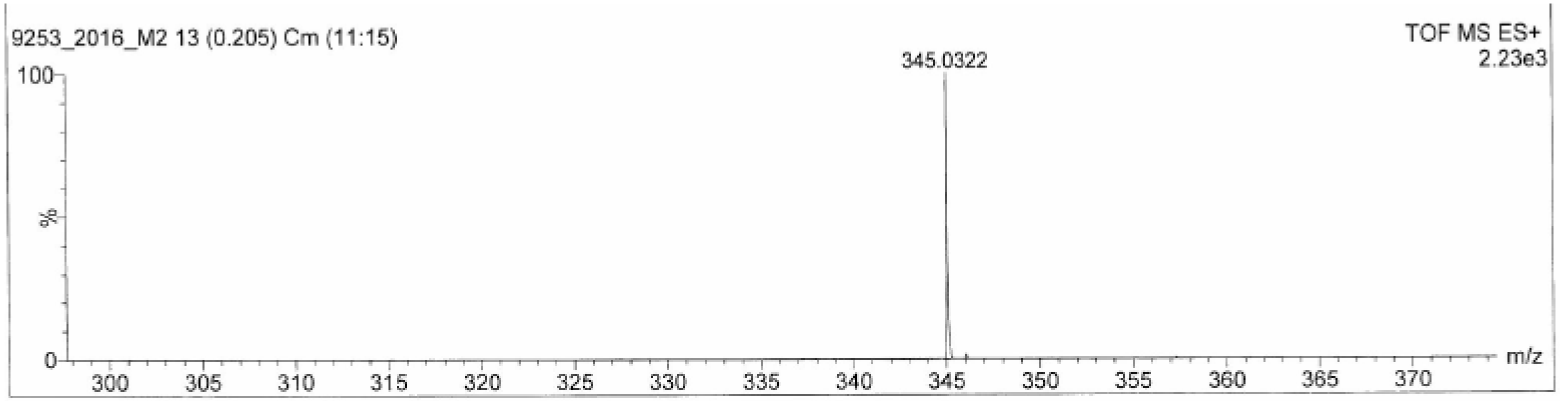

Figure S63. HRMS spectrum of iodolactone **4b-A**.

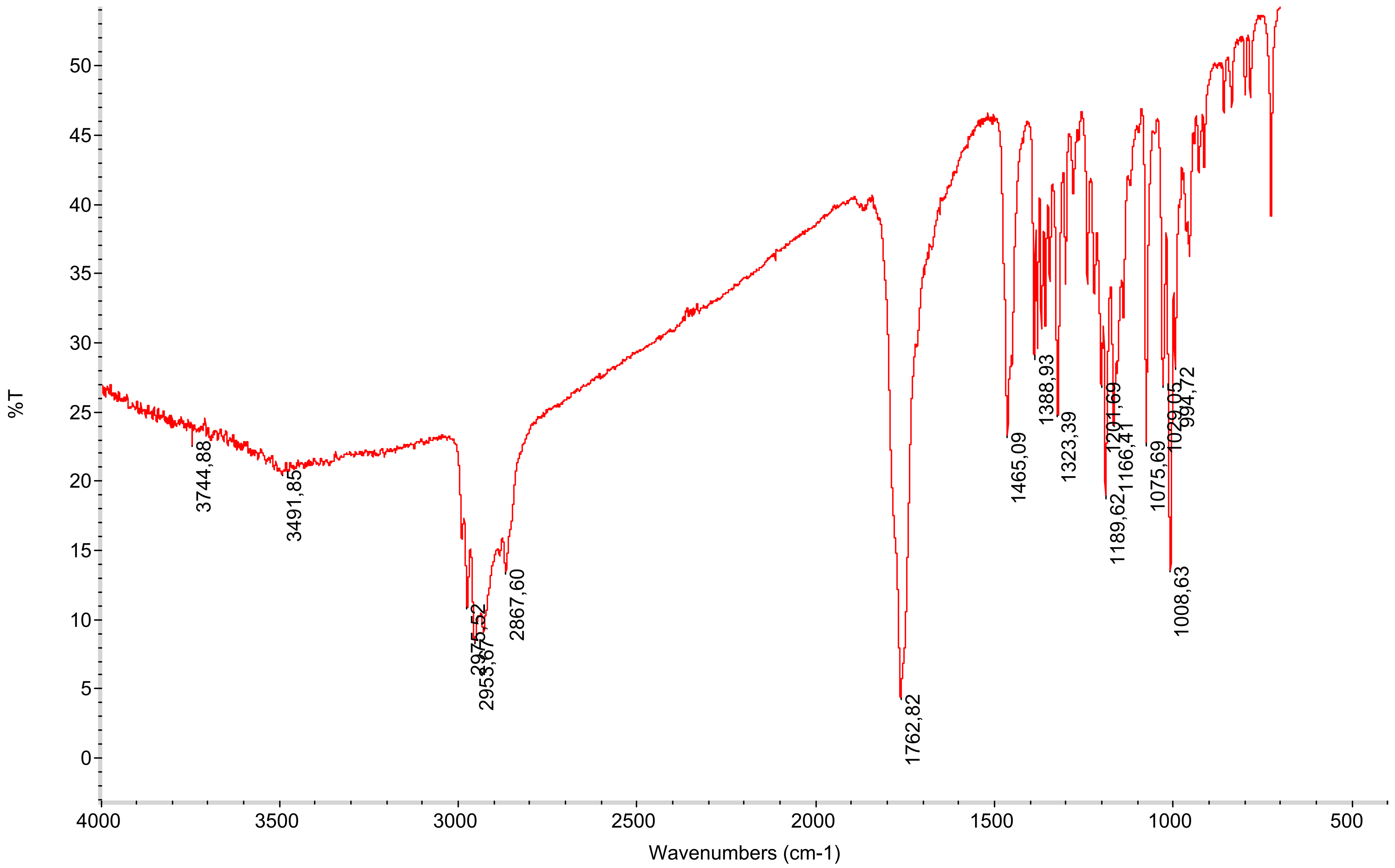

Figure S64. IR spectrum of iodolactone **4b-A**.

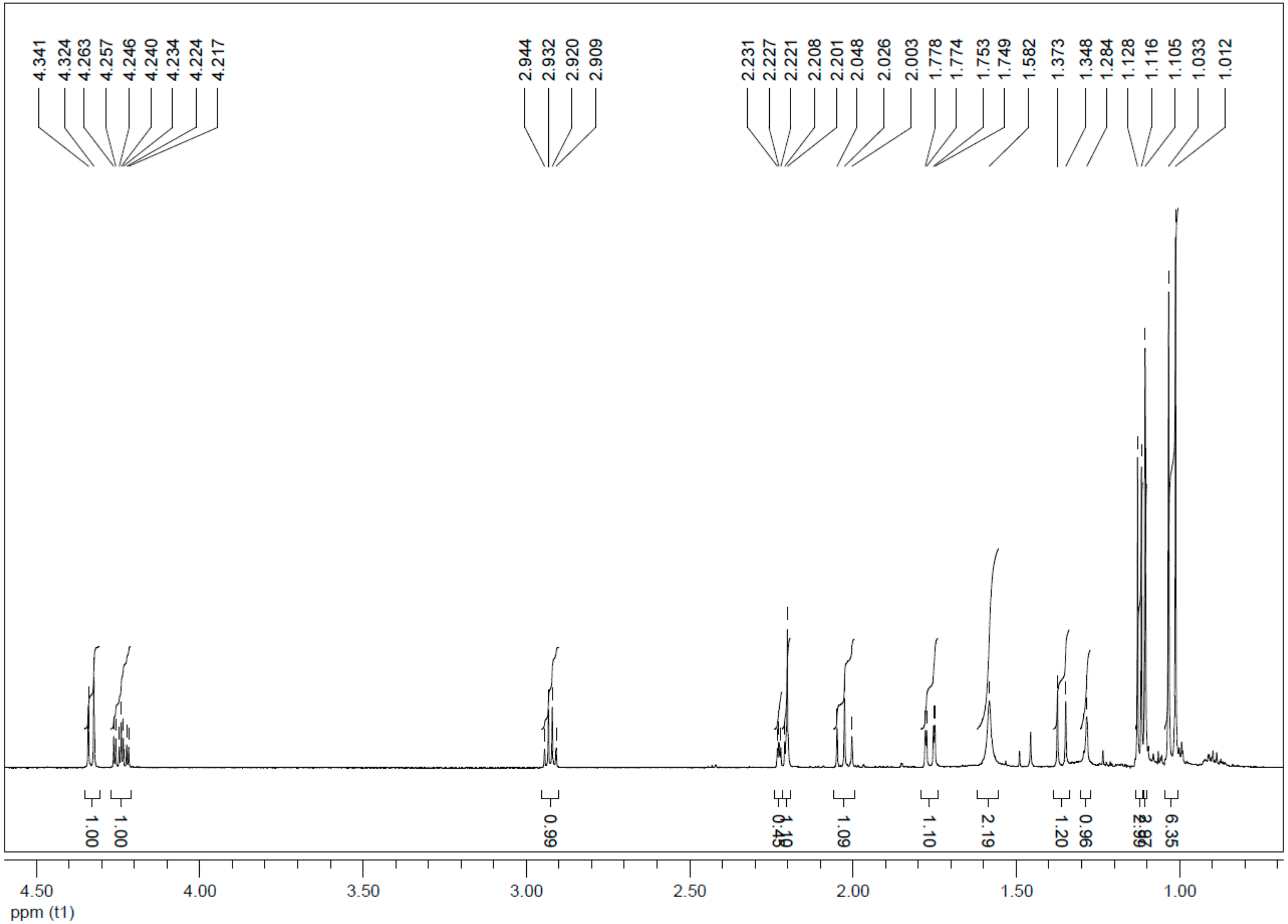

Figure S65. ¹H-NMR (600 MHz, $CDCl_3$) spectrum of iodolactone **4b-B**.

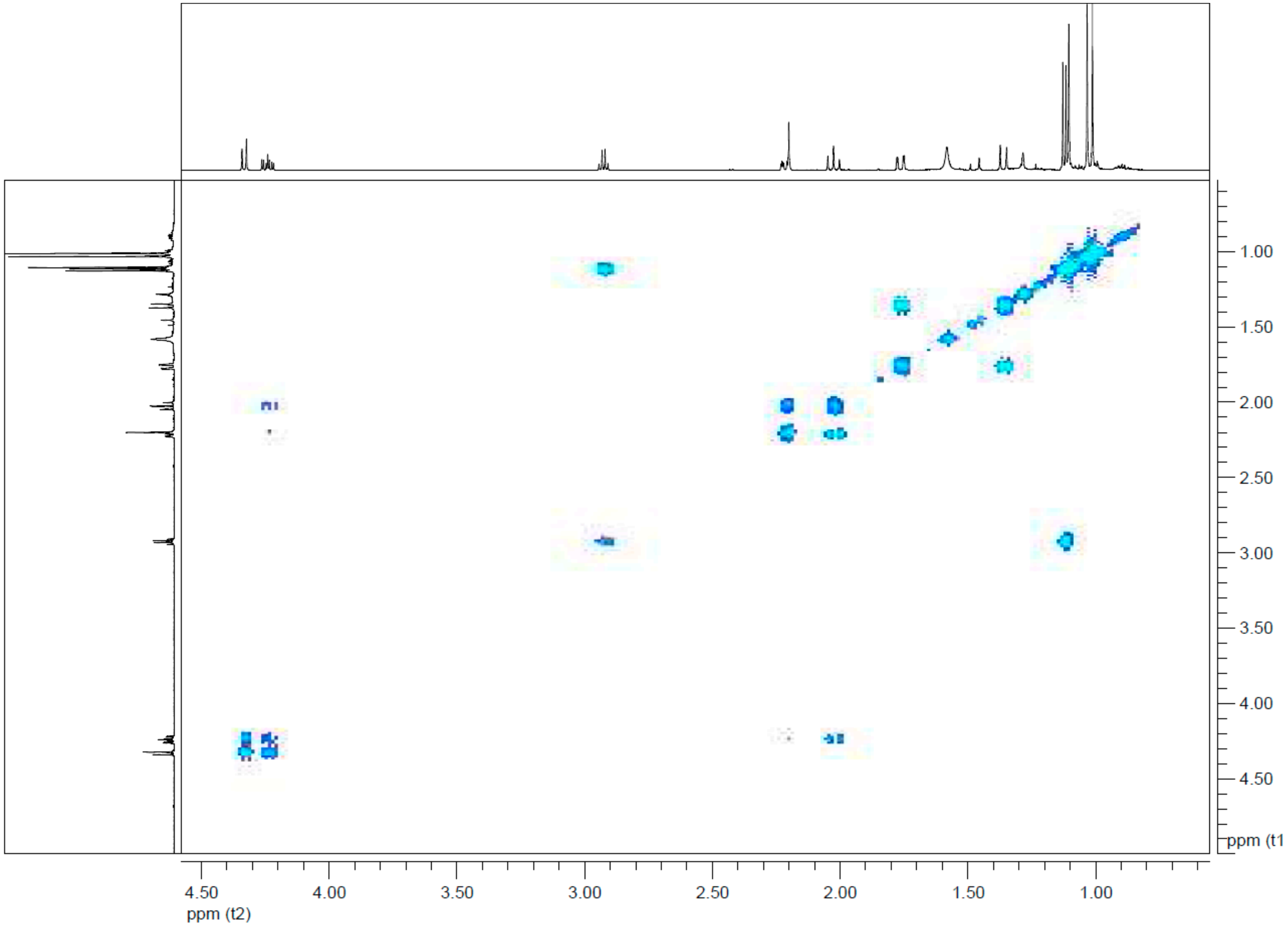

Figure S66. COSY (151 MHz, $CDCl_3$) spectrum of iodolactone **4b-B**.

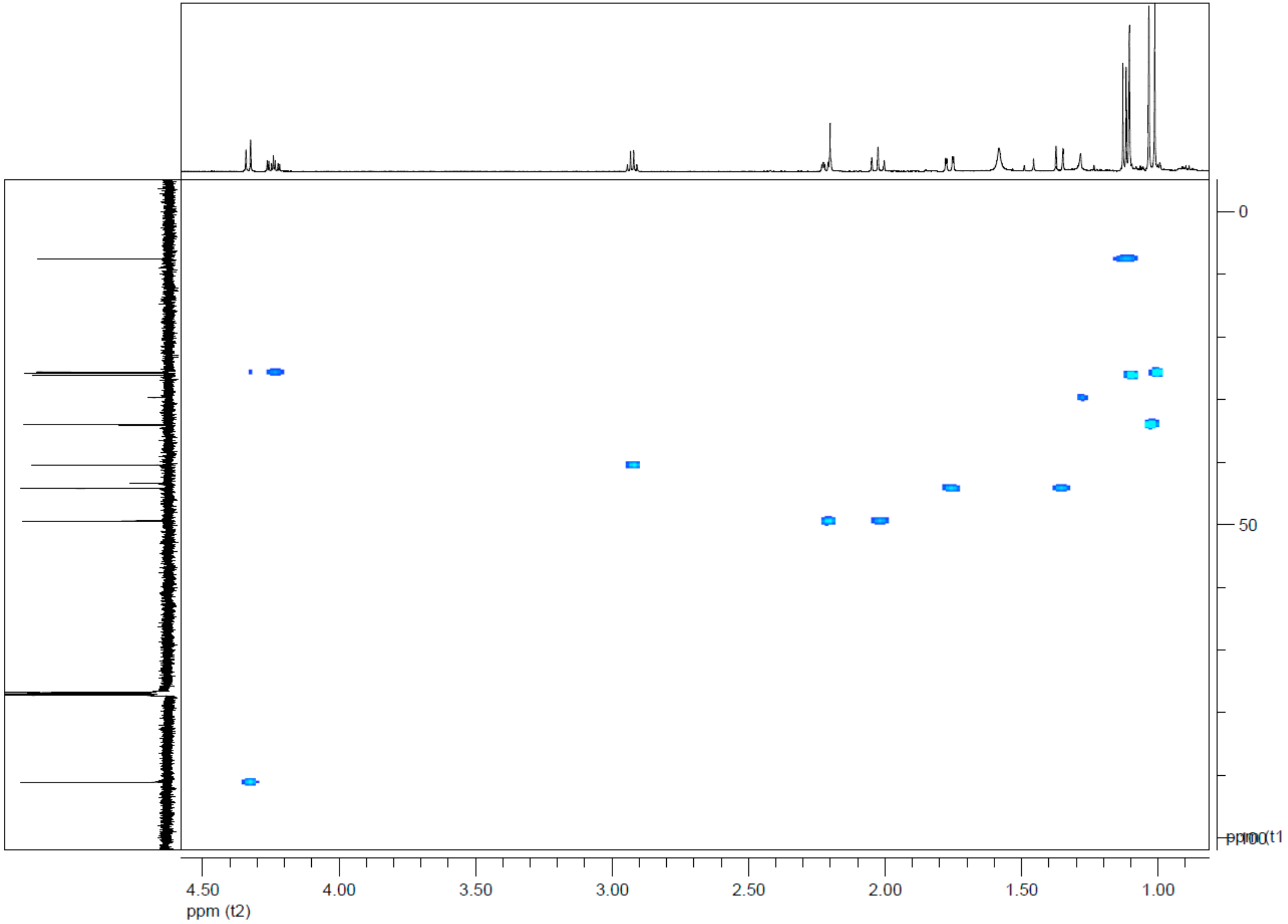

Figure S67. HMQC (151 MHz, $CDCl_3$) spectrum of iodolactone **4b-B**.

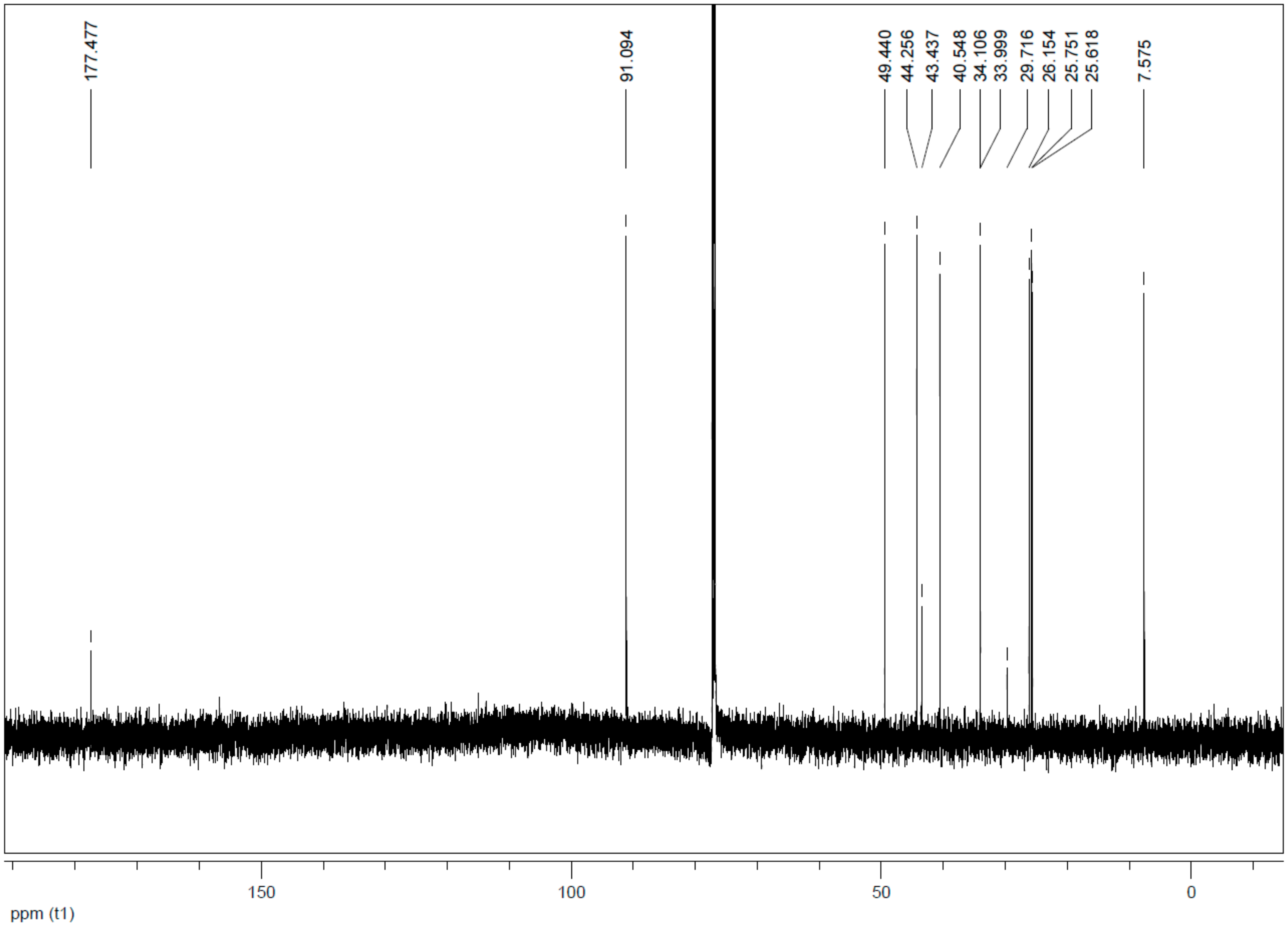

Figure S68. ^{13}C-NMR (151 MHz, $CDCl_3$) spectrum of iodolactone **4b-B**.

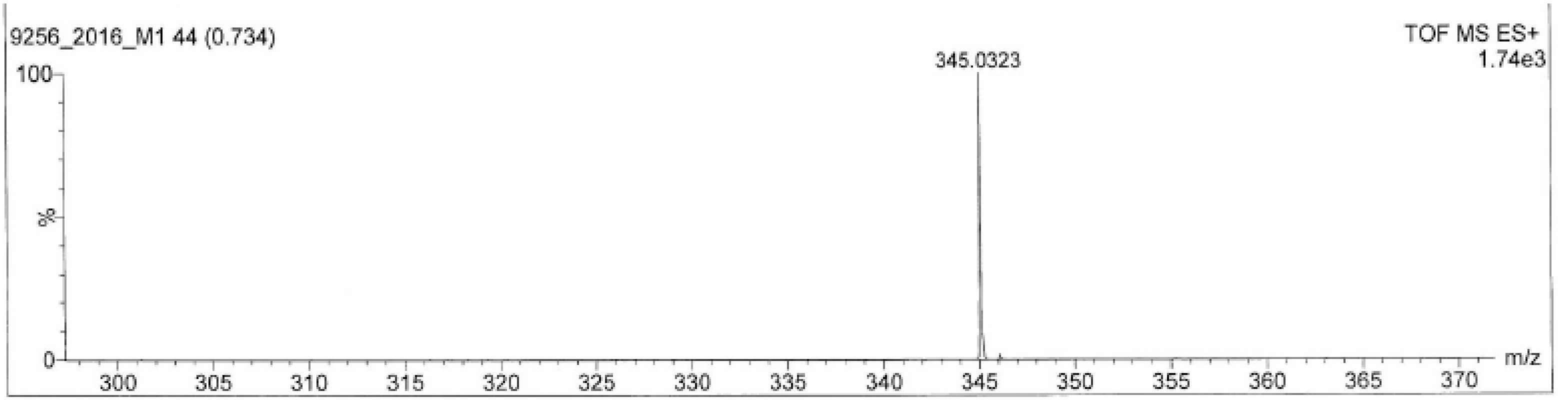

Figure S69. HRMS spectrum of iodolactone **4b-B**.

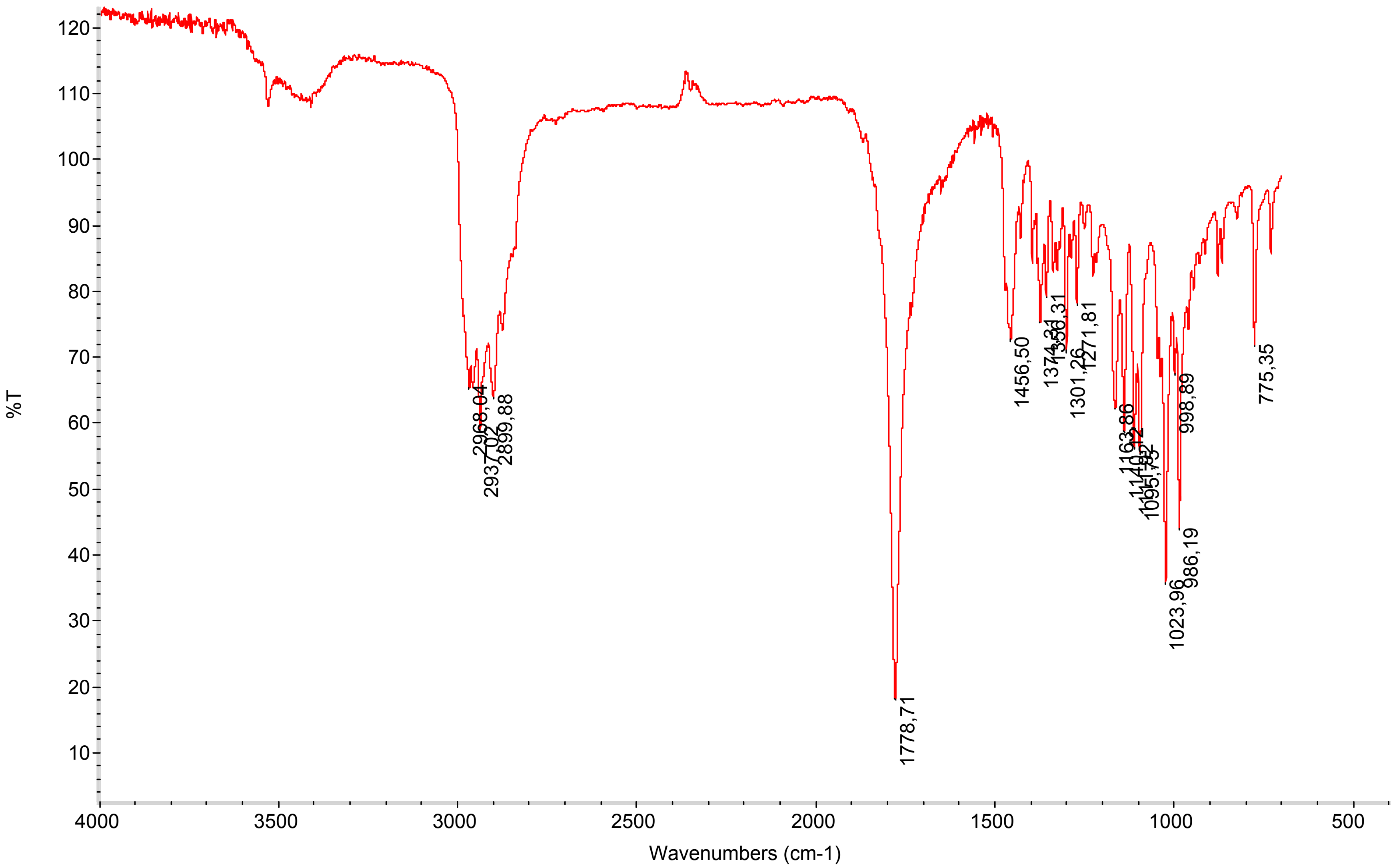

Figure S70. IR spectrum of iodolactone **4b-B**.

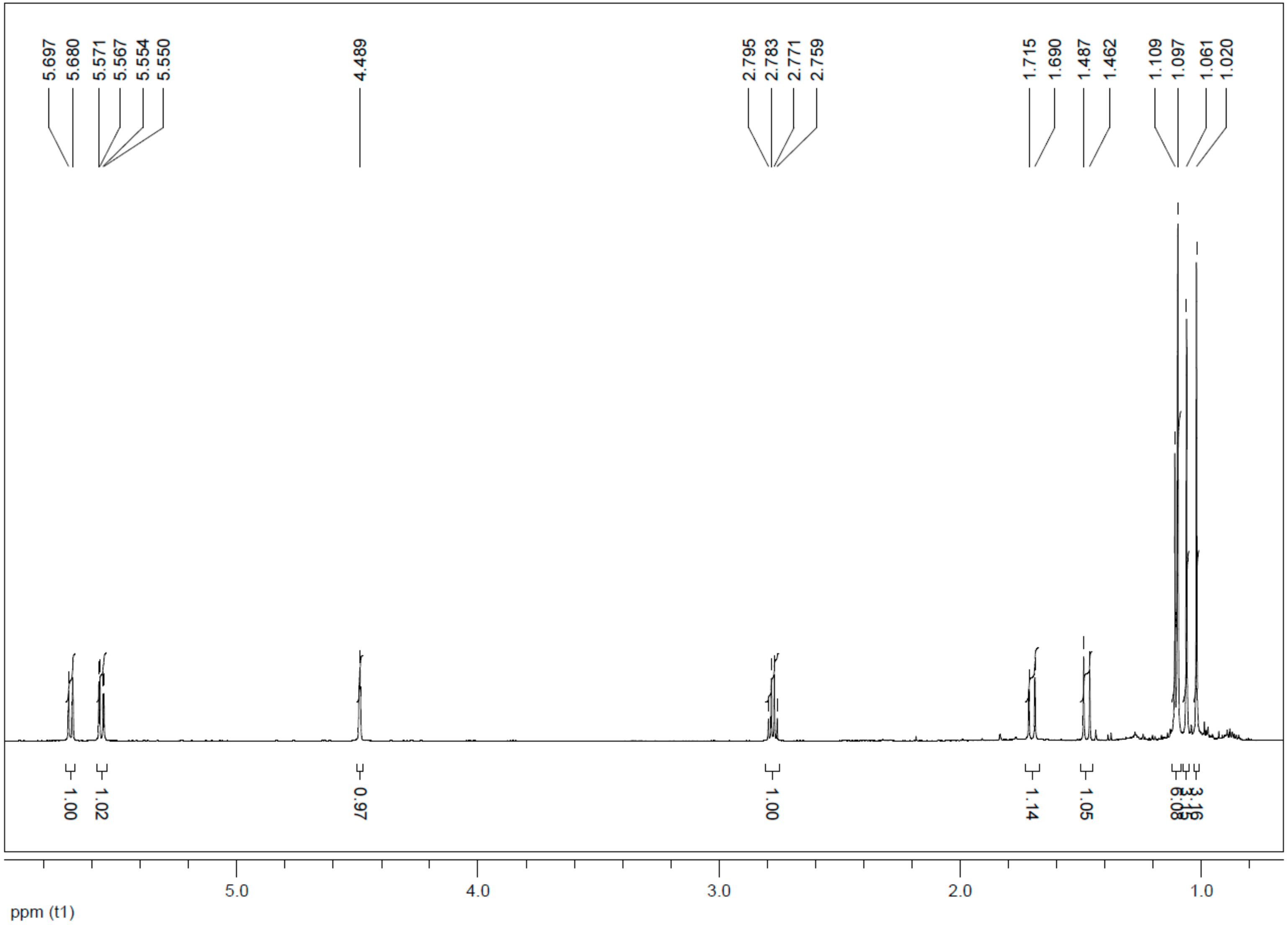

Figure S71. ^{1}H-NMR (600 MHz, $CDCl_3$) spectrum of unsaturated lactone **5b-A**.

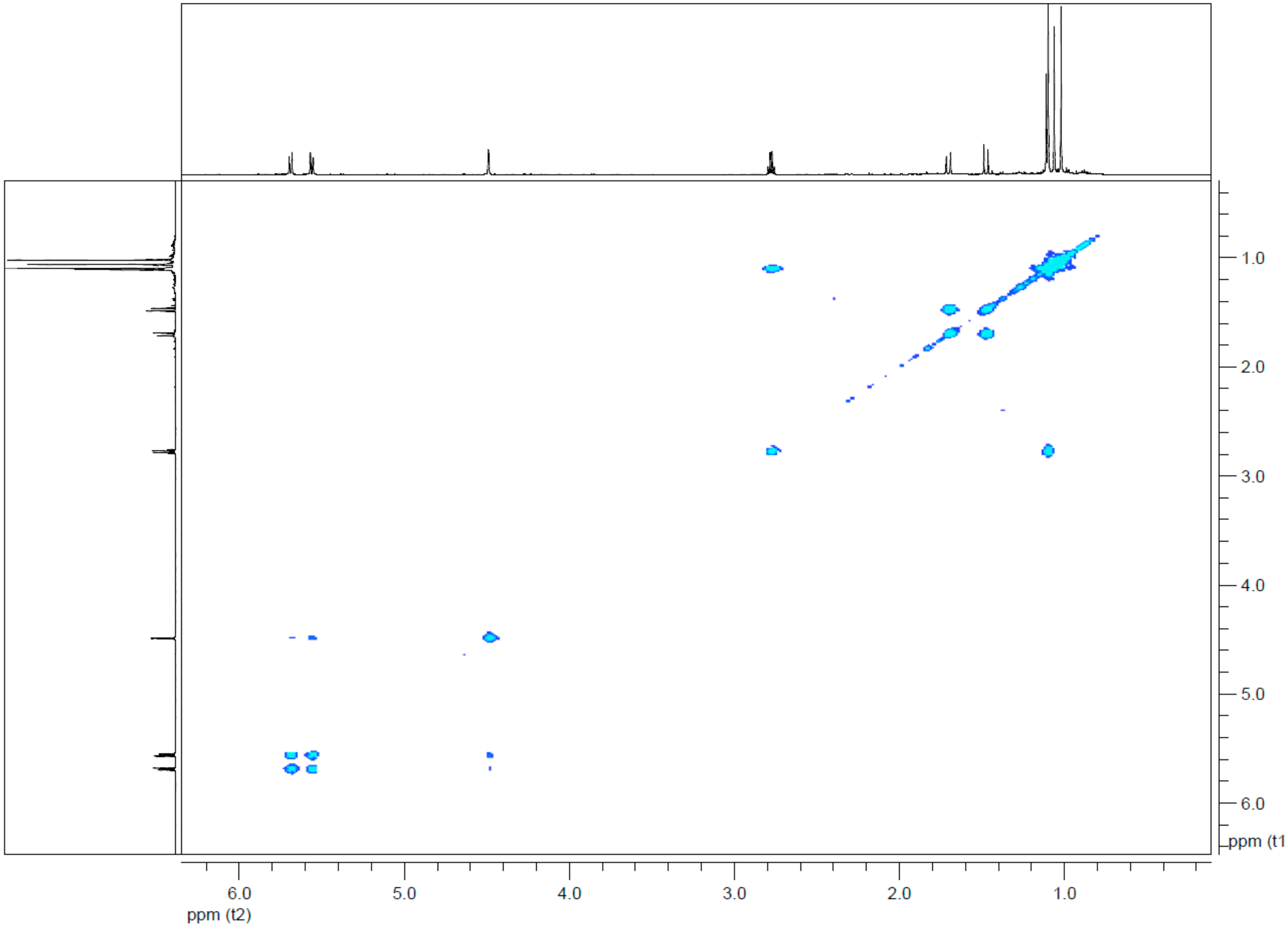

Figure S72. COSY (151 MHz, $CDCl_3$) spectrum of unsaturated lactone **5b-A**.

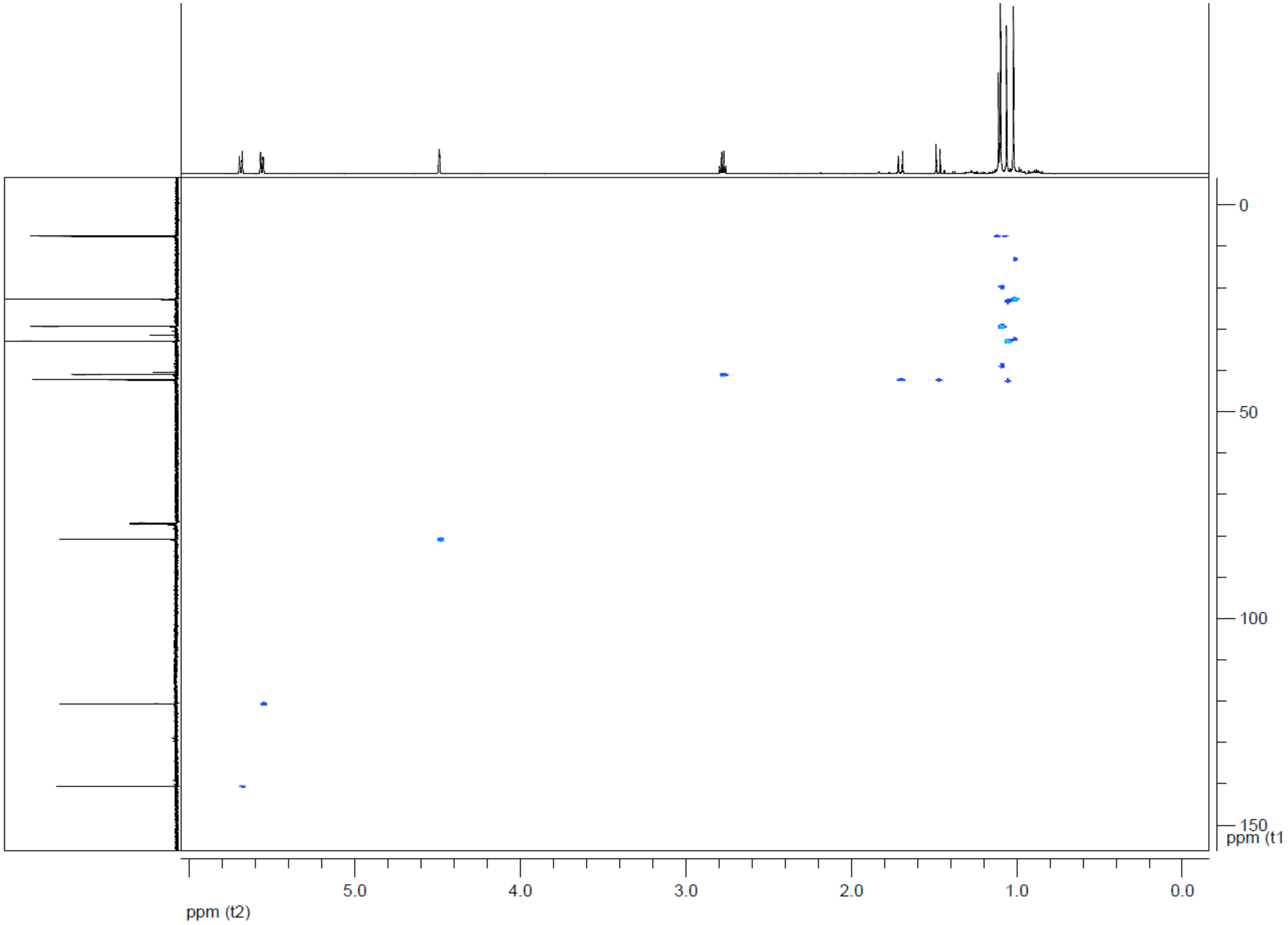

Figure S73. HMQC (151 MHz, $CDCl_3$) spectrum of unsaturated lactone **5b-A**.

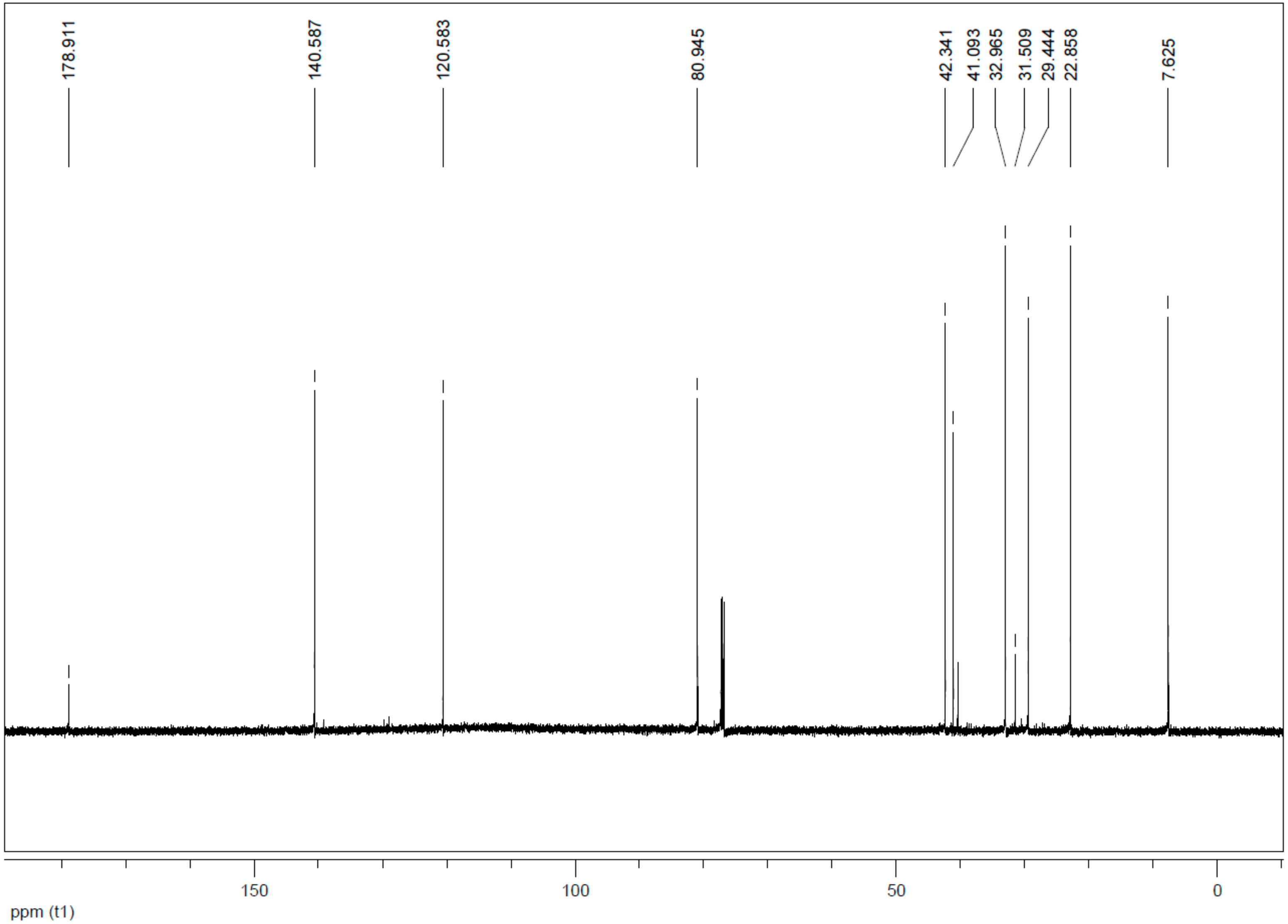

Figure S74. ^{13}C-NMR (151 MHz, $CDCl_3$) spectrum of unsaturated lactone **5b-A**.

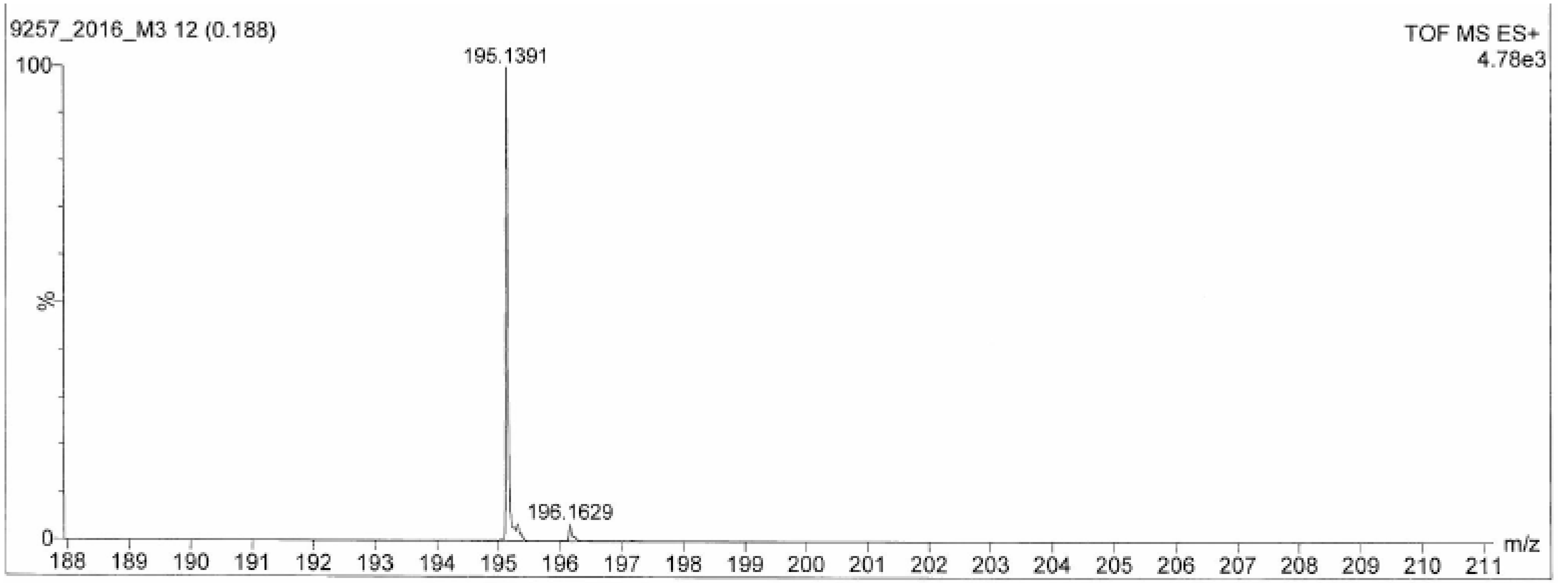

Figure S75. HRMS spectrum of unsaturated lactone **5b-A**.

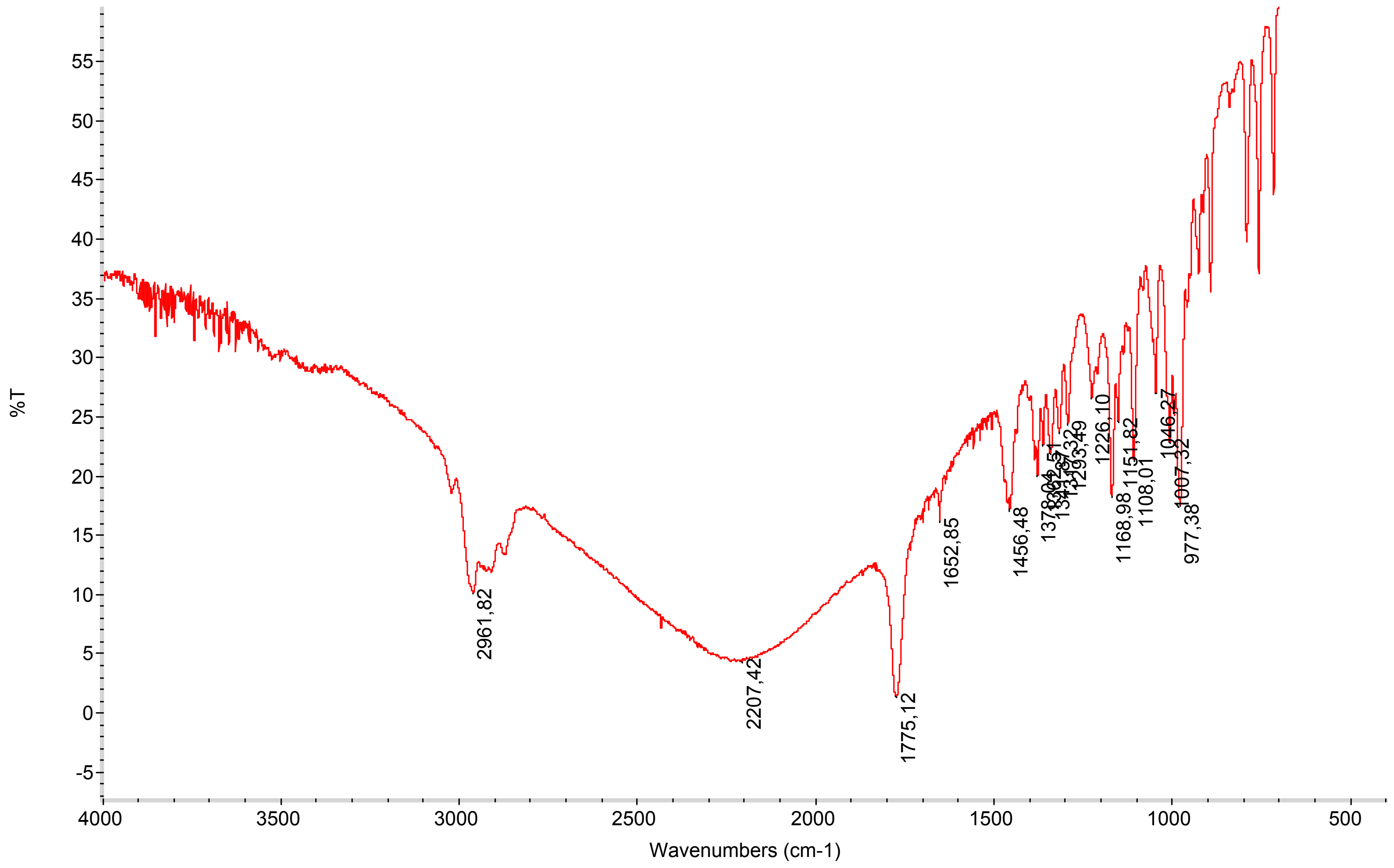

Figure S76. IR spectrum of unsaturated lactone **5b-A**.

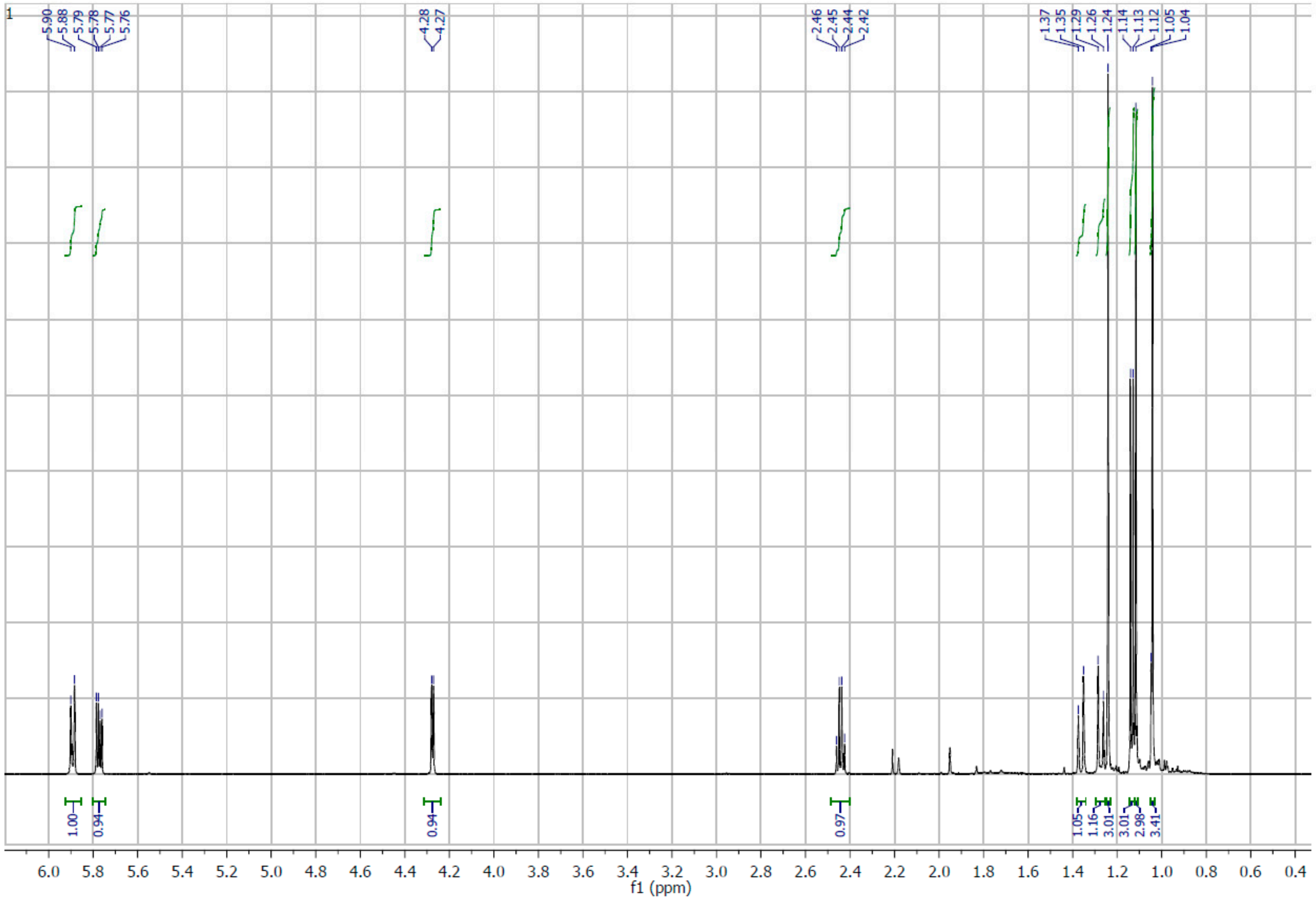

Figure S77. ^{1}H-NMR (600 MHz, $CDCl_3$) spectrum of unsaturated lactone **5b-B**.

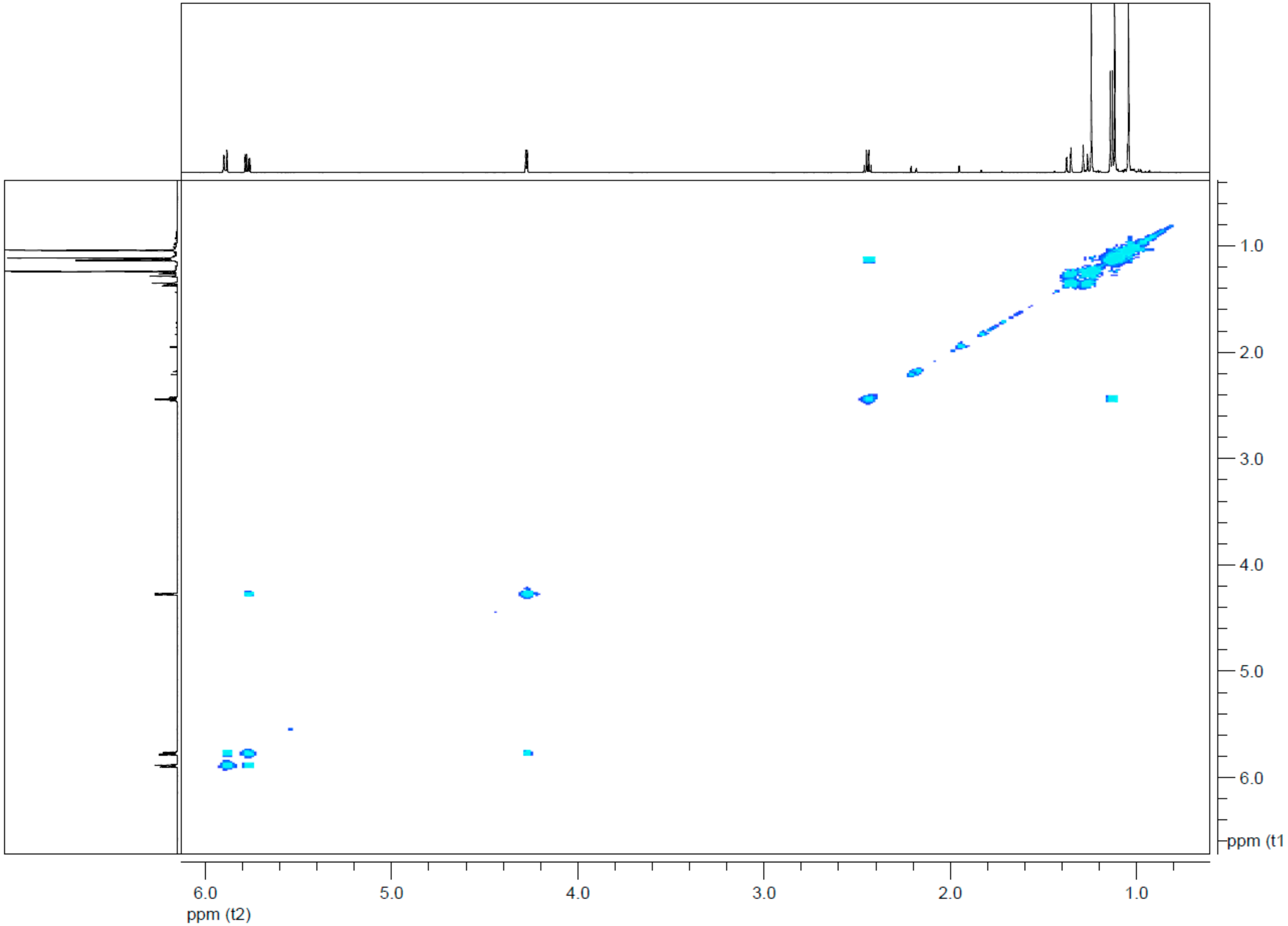

Figure S78. COSY (151 MHz, $CDCl_3$) spectrum of unsaturated lactone **5b-B**.

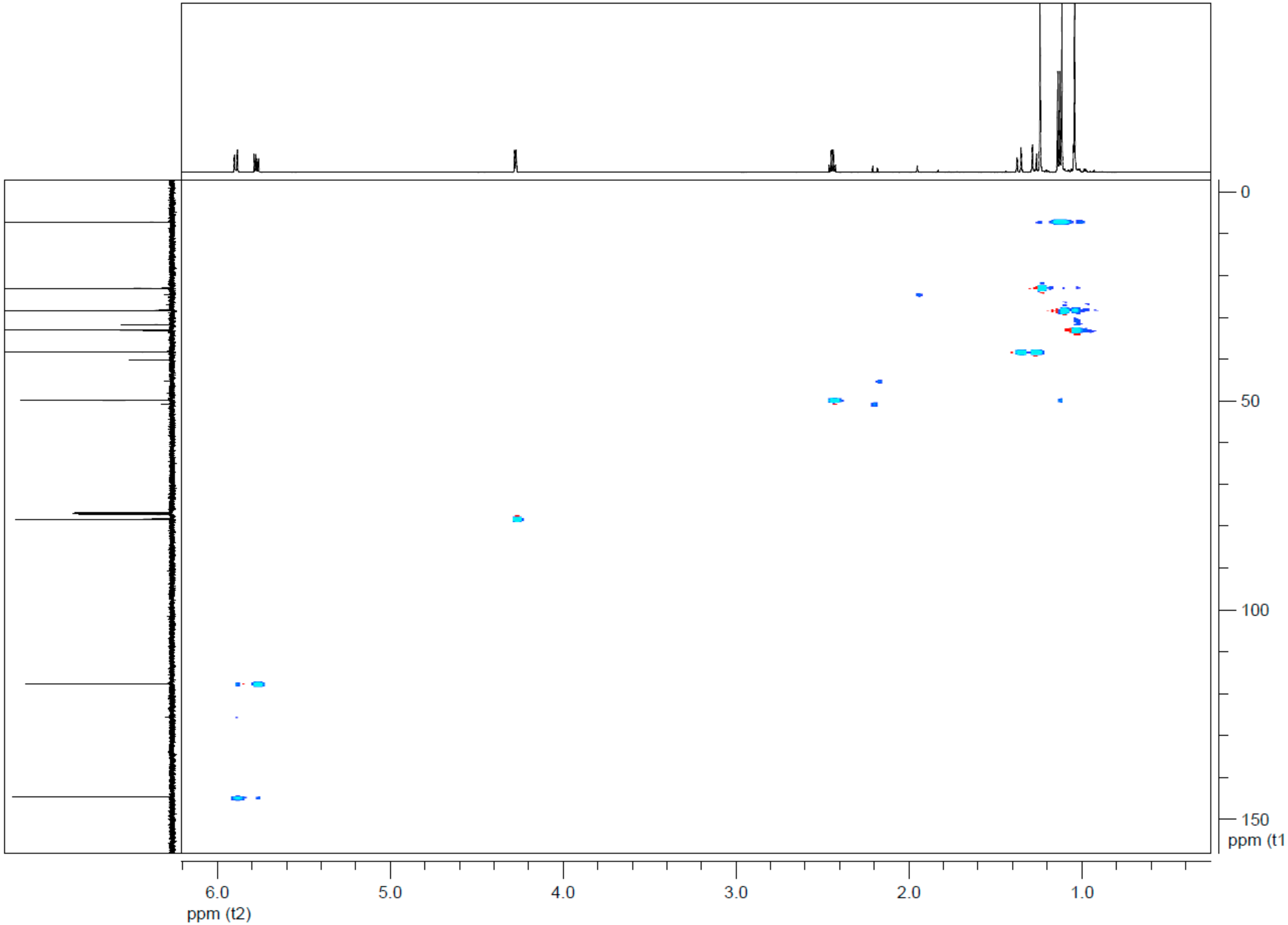

Figure S79. HMQC (151 MHz, $CDCl_3$) spectrum of unsaturated lactone **5b-B**.

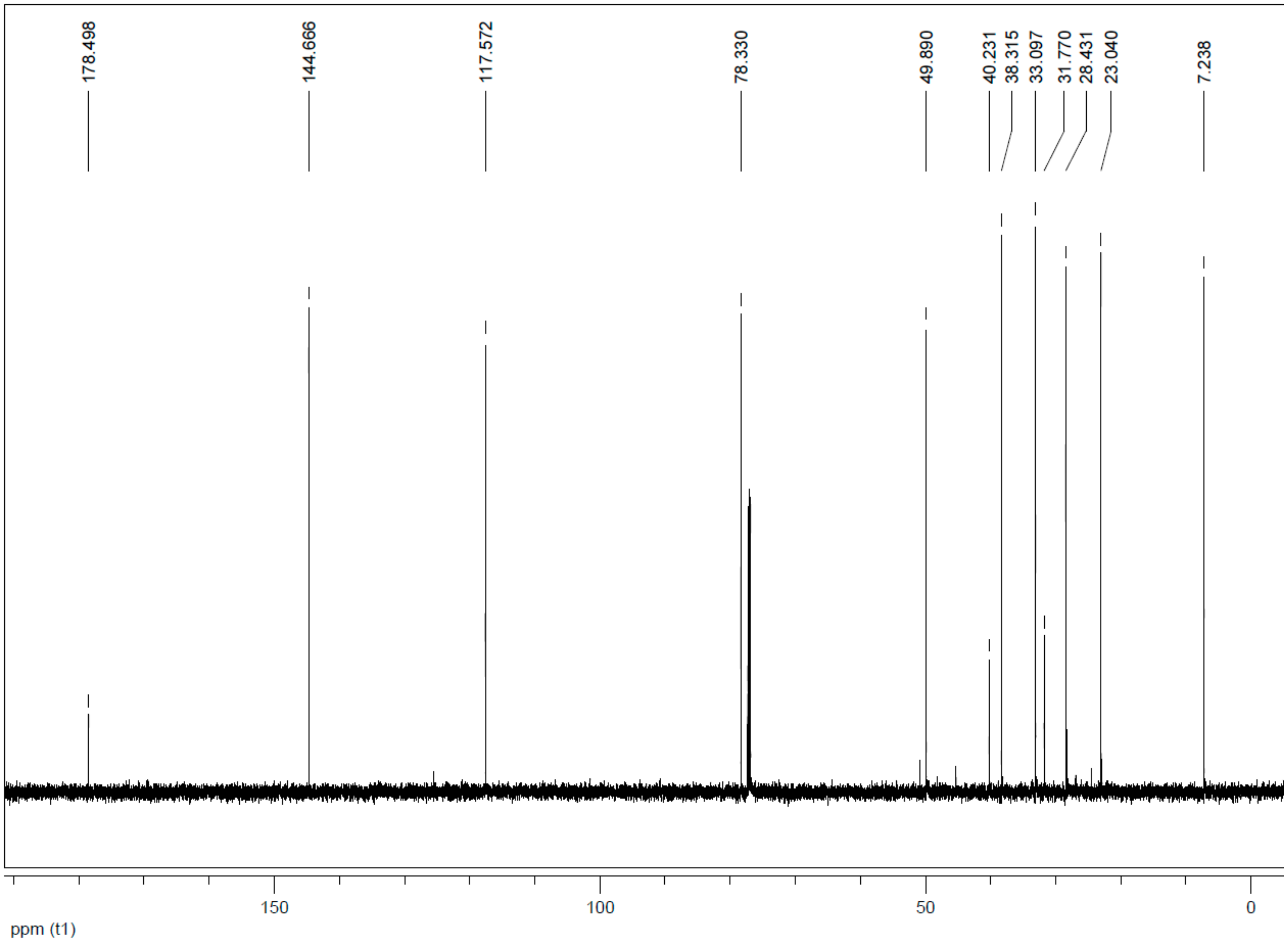

Figure S80. ^{13}C-NMR (151 MHz, $CDCl_3$) spectrum of unsaturated lactone **5b-B**.

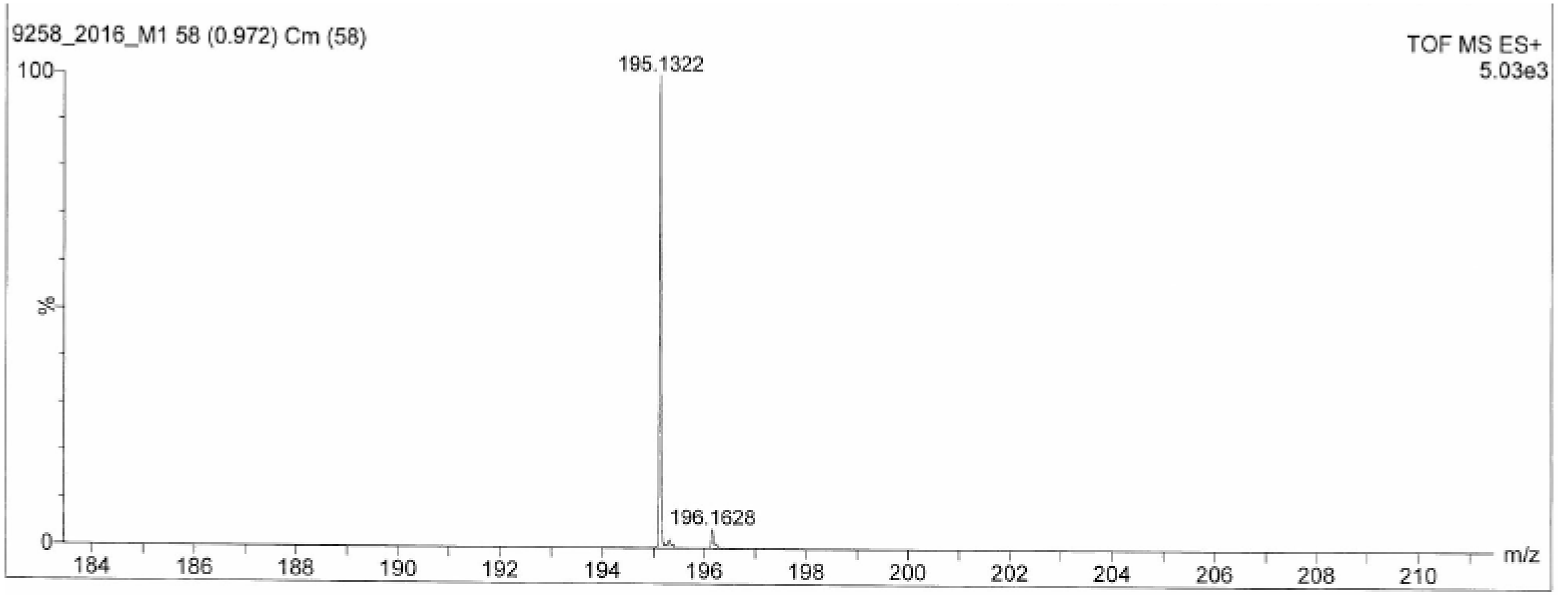

Figure S81. HRMS spectrum of unsaturated lactone **5b-B**.

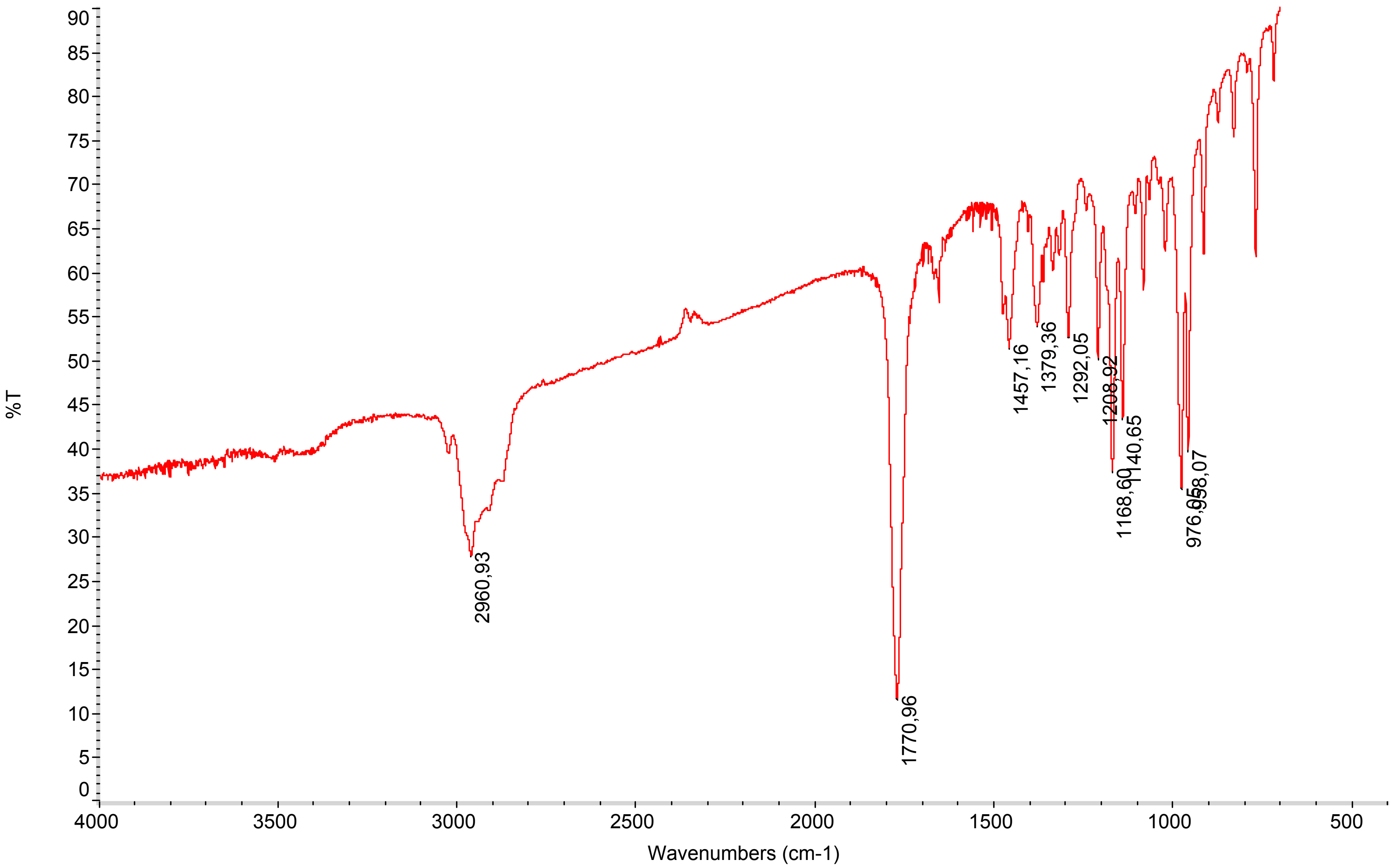

Figure S82. IR spectrum of unsaturated lactone **5b-B**.

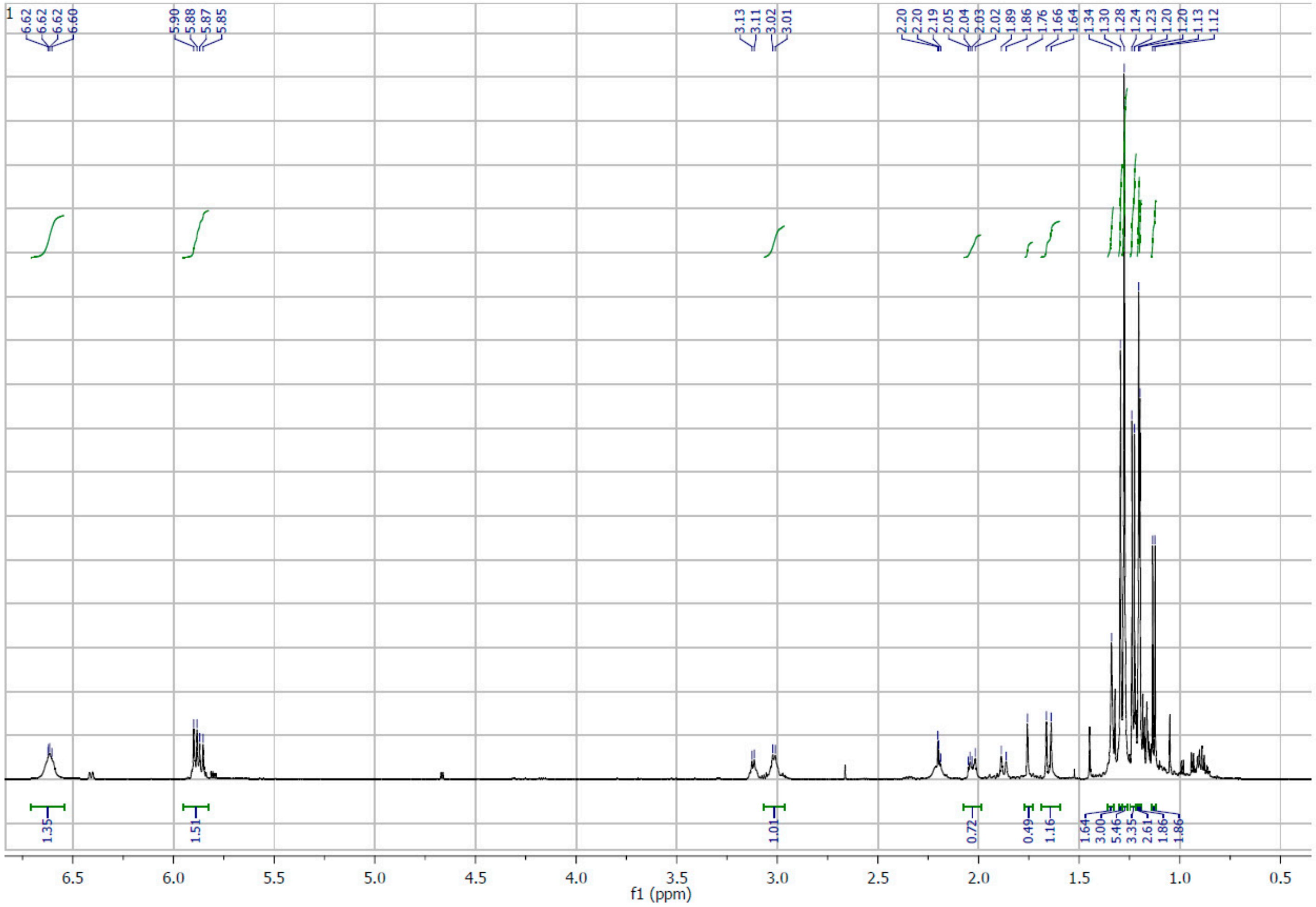

Figure S83. ^{1}H-NMR (600 MHz, $CDCl_3$) spectrum of hydroxylactone **6b**.

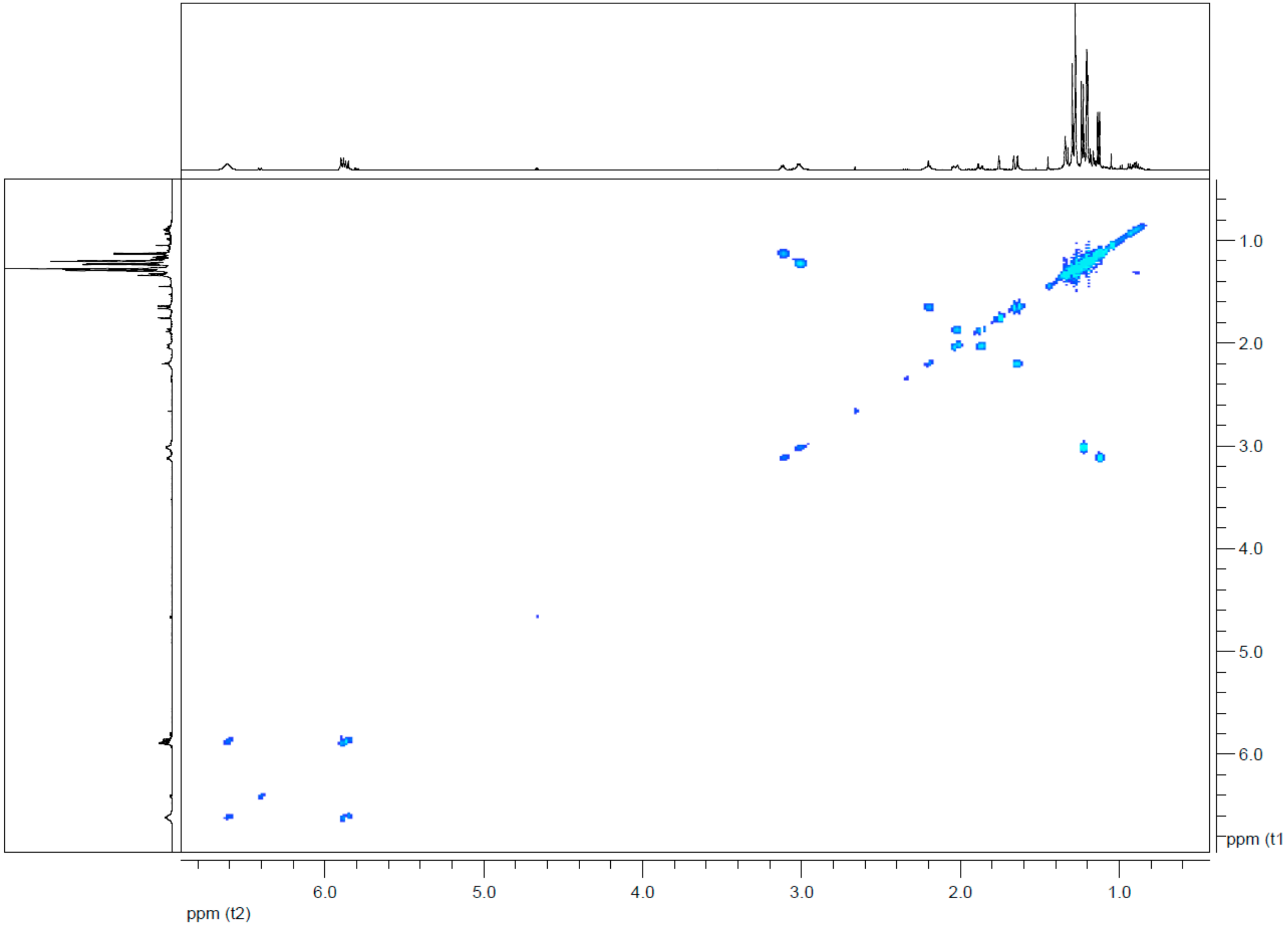

Figure S84. COSY (151 MHz, $CDCl_3$) spectrum of hydroxylactone **6b**.

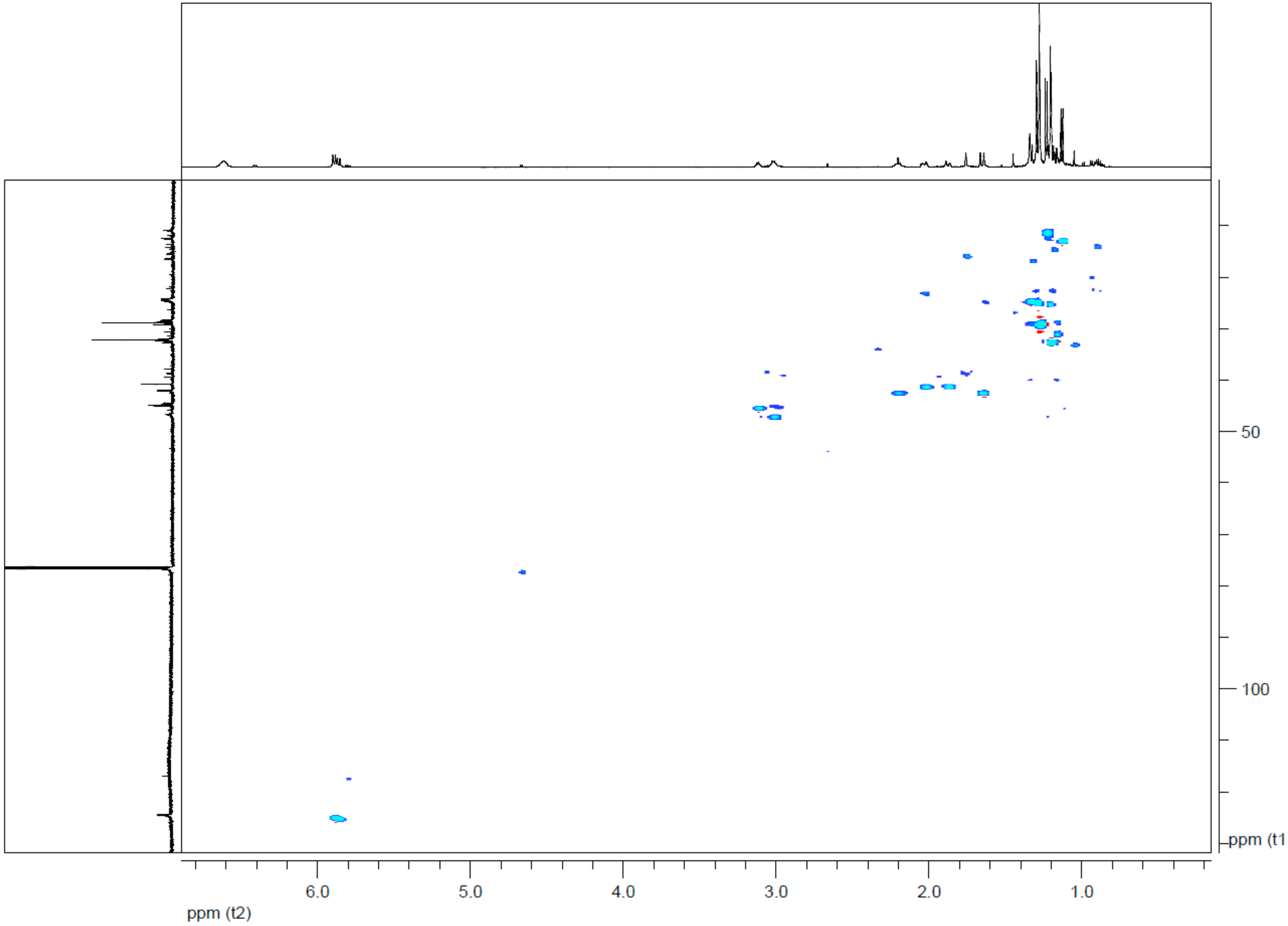

Figure S85. HMQC (151 MHz, $CDCl_3$) spectrum of hydroxylactone **6b**.

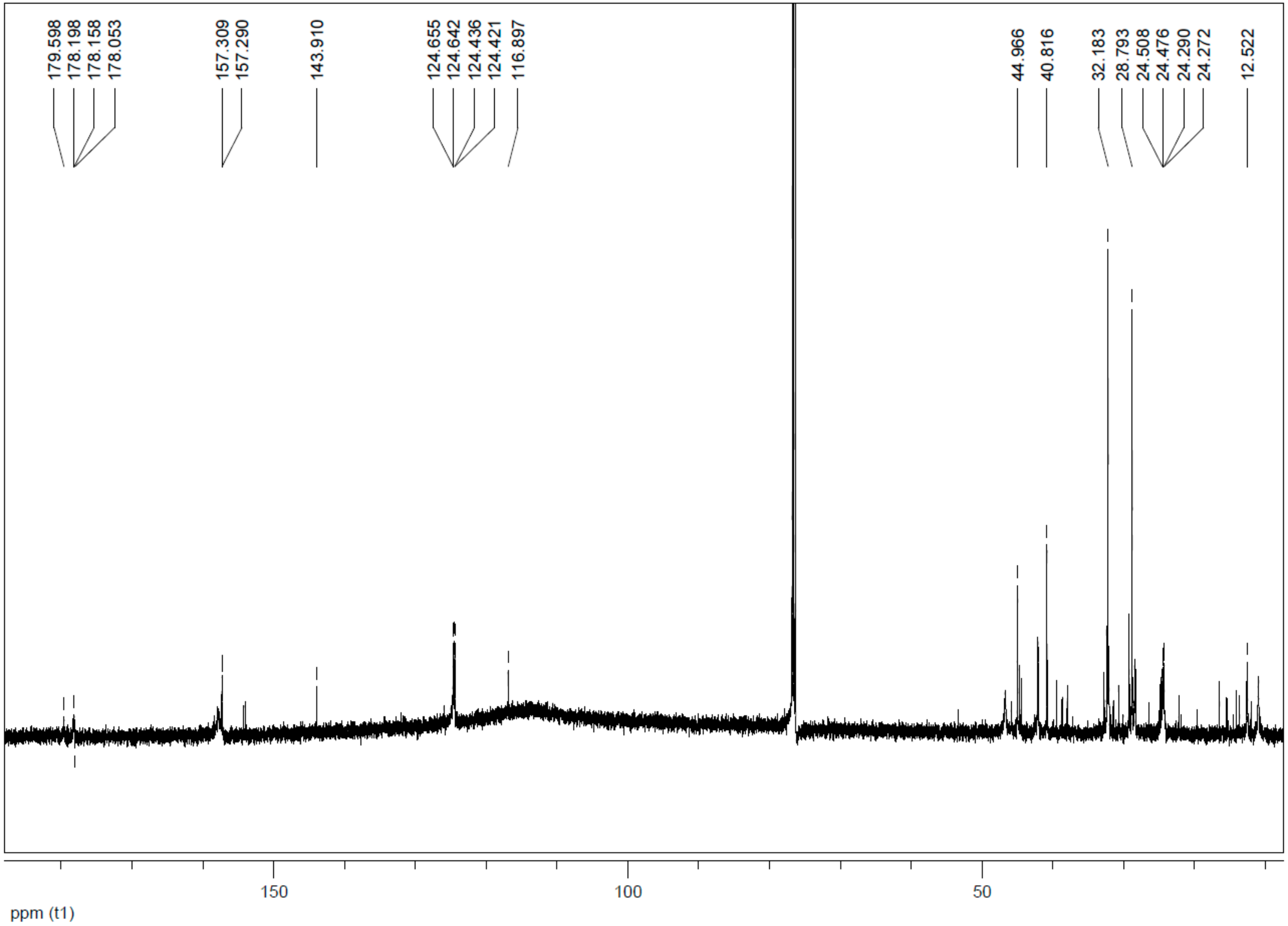

Figure S86. ^{13}C-NMR (151 MHz, $CDCl_3$) spectrum of hydroxylactone **6b**.

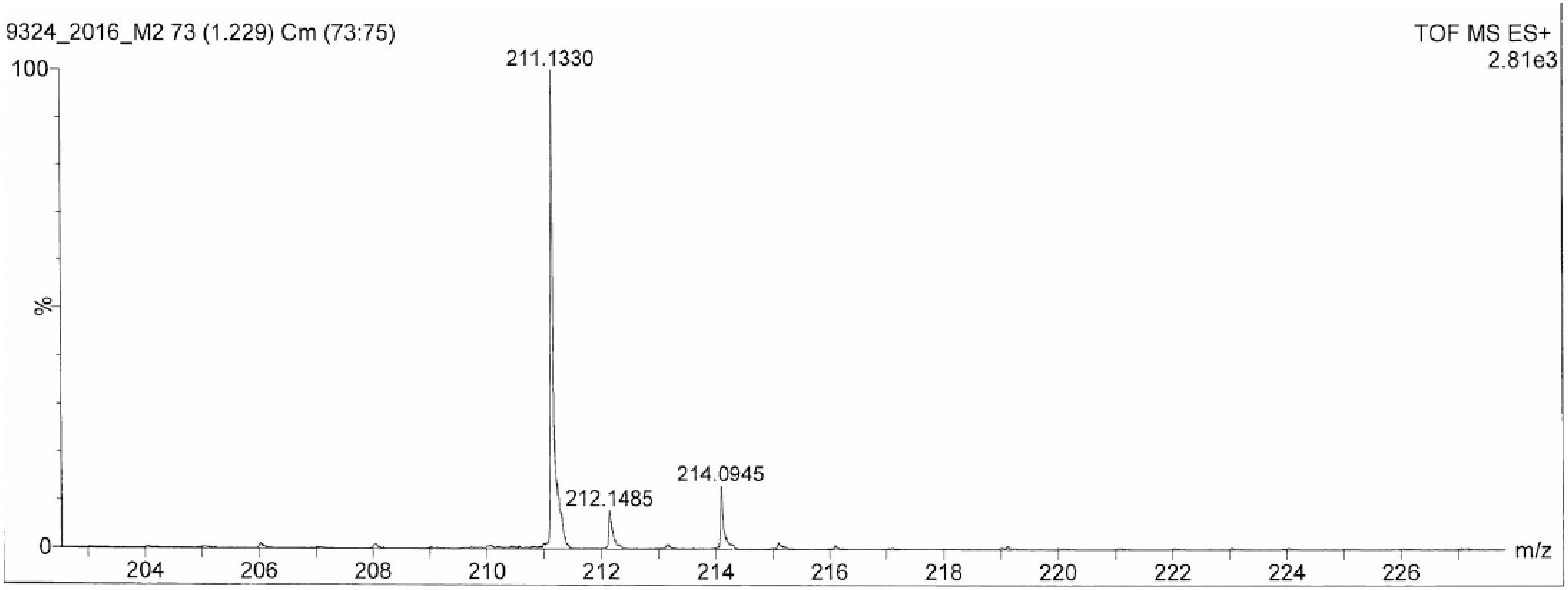

Figure S87. HRMS spectrum of hydroxylactone **6b**.

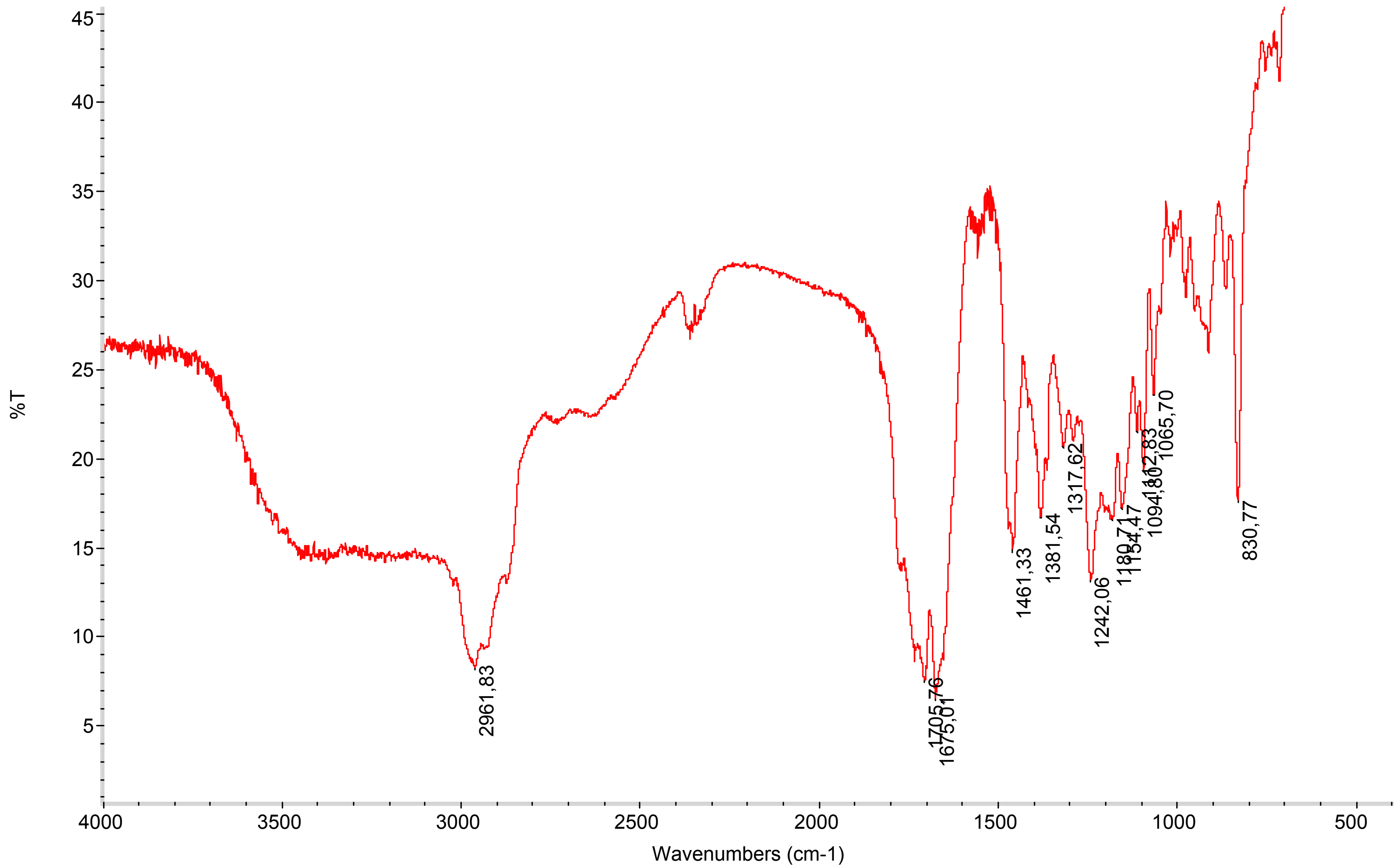

Figure S88. IR spectrum of hydroxylactone **6b**.

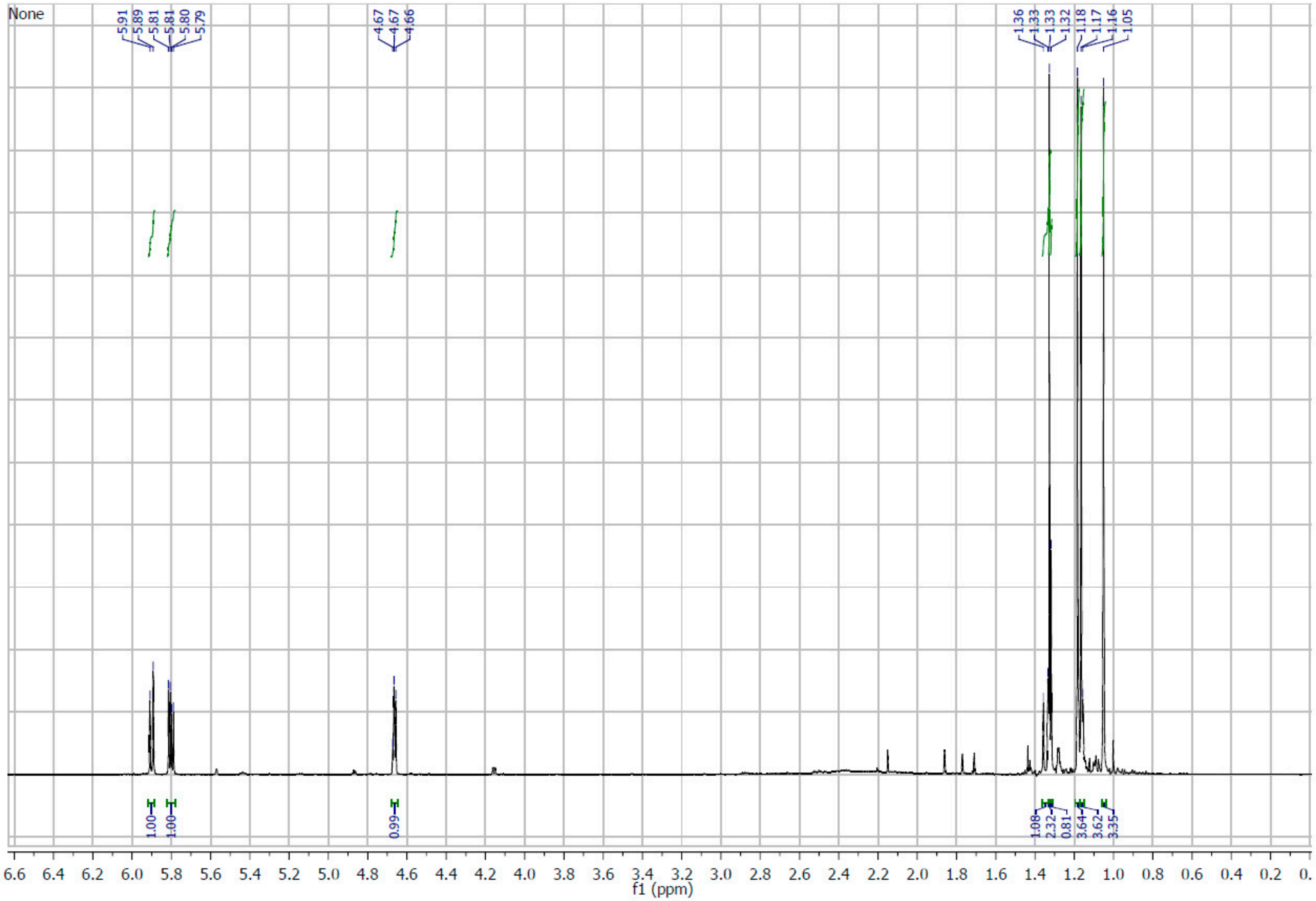

Figure S89. ^{1}H-NMR (600 MHz, $CDCl_3$) spectrum of hydroxylactone **7b**.

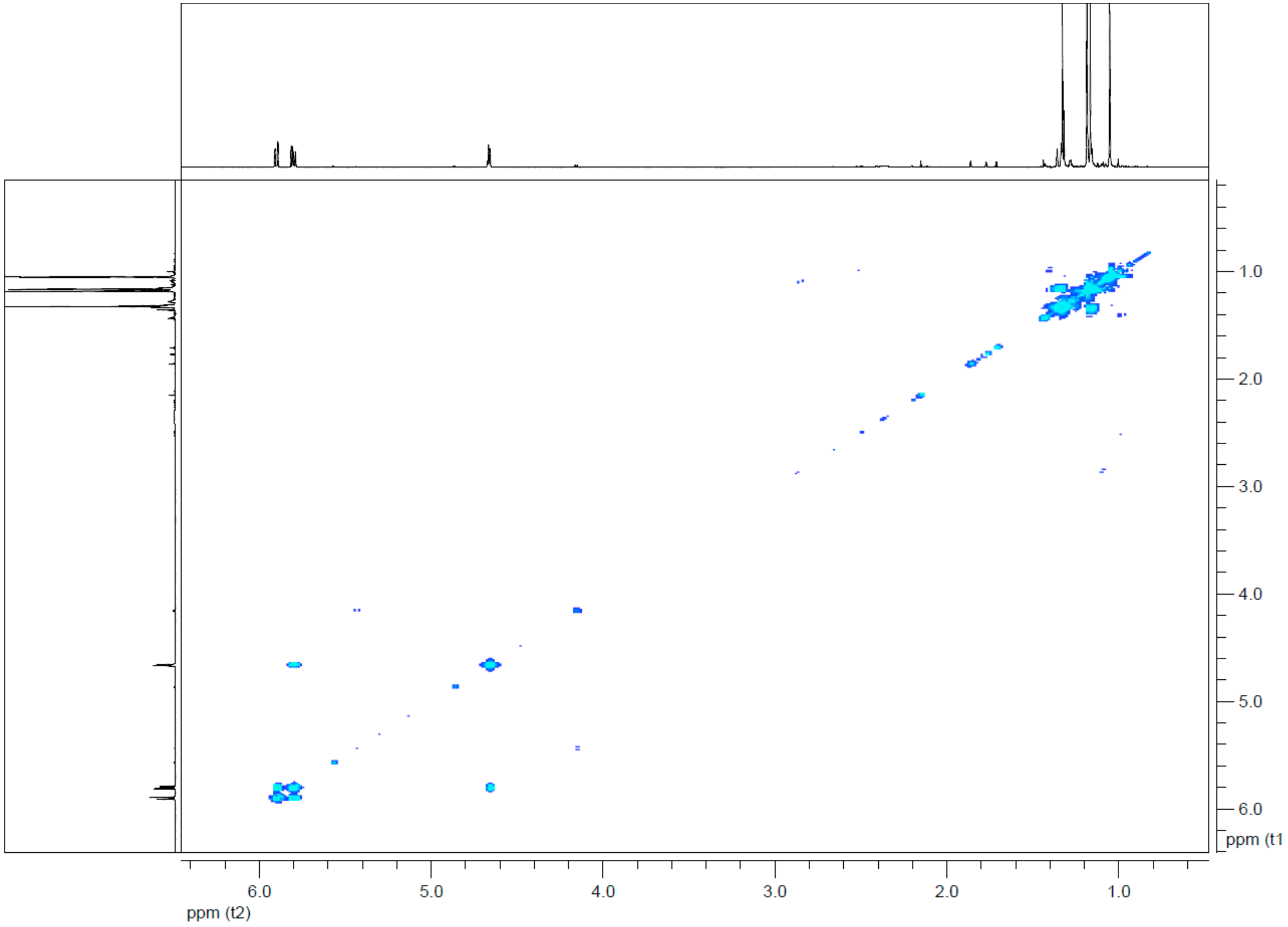

Figure S90. COSY (151 MHz, $CDCl_3$) spectrum of hydroxylactone **7b**.

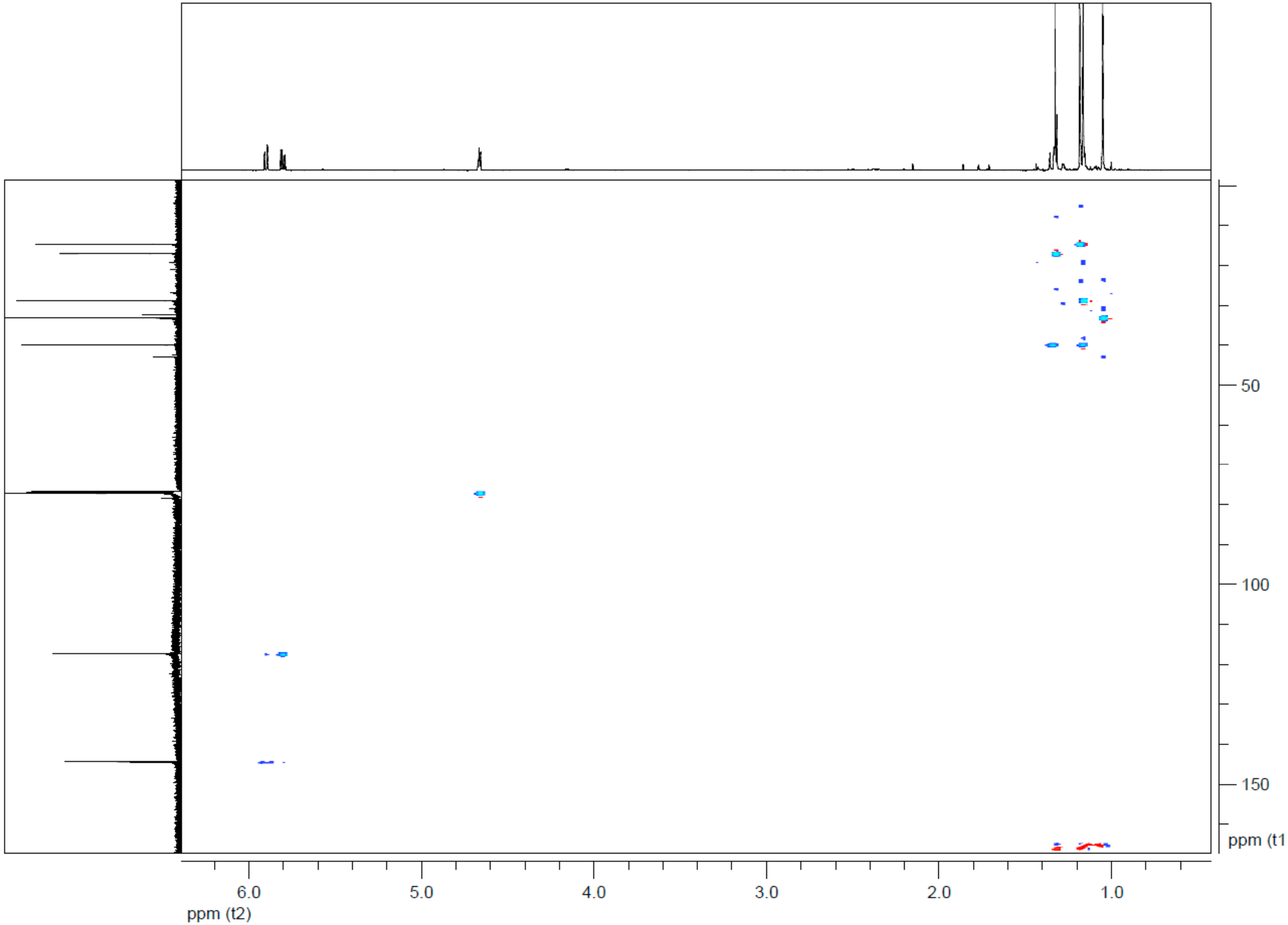

Figure S91. HMQC (151 MHz, $CDCl_3$) spectrum of hydroxylactone **7b**.

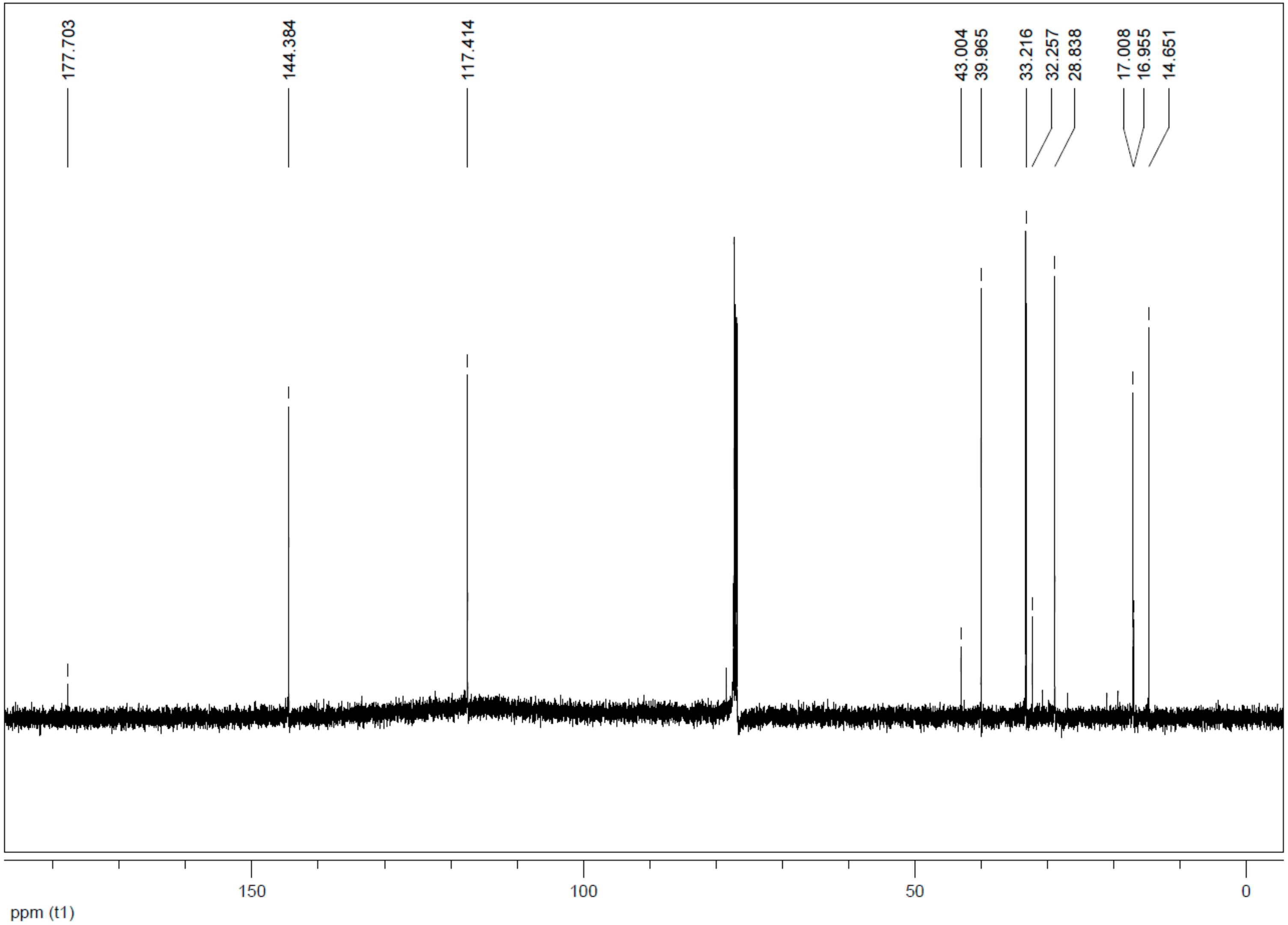

Figure S92. ^{13}C-NMR (151 MHz, $CDCl_3$) spectrum of hydroxylactone **7b**.

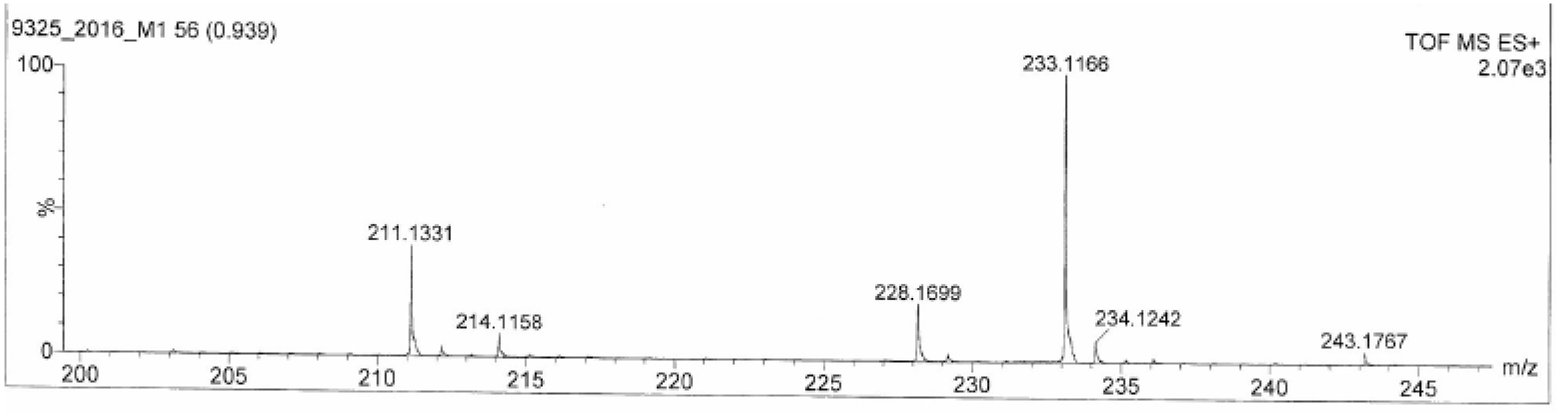

Figure S93. HRMS spectrum of hydroxylactone **7b**.

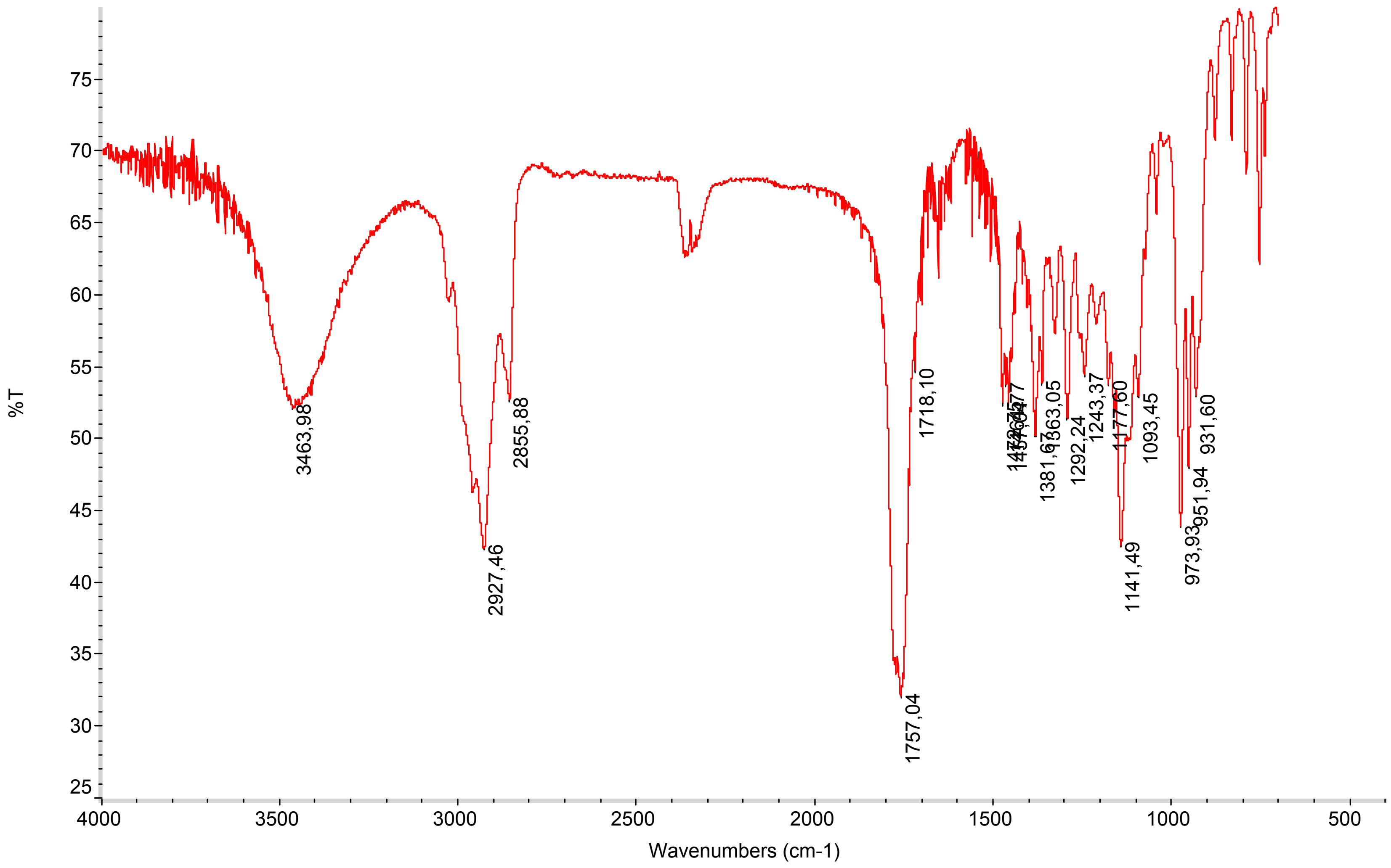

Figure S94. IR spectrum of hydroxylactone **7b**.

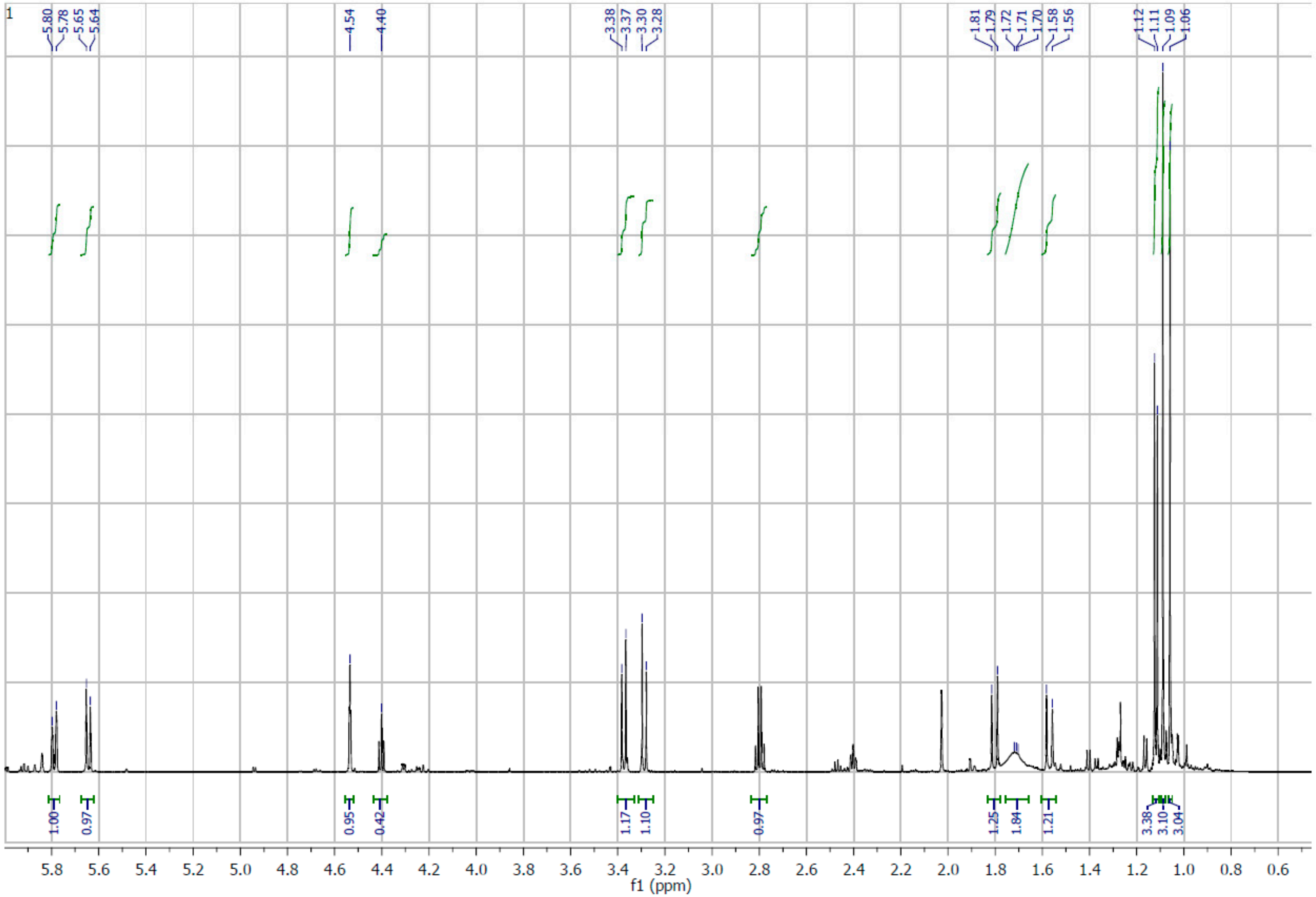

Figure S95. ^{1}H-NMR (600 MHz, $CDCl_3$) spectrum of hydroxylactone **8b**.

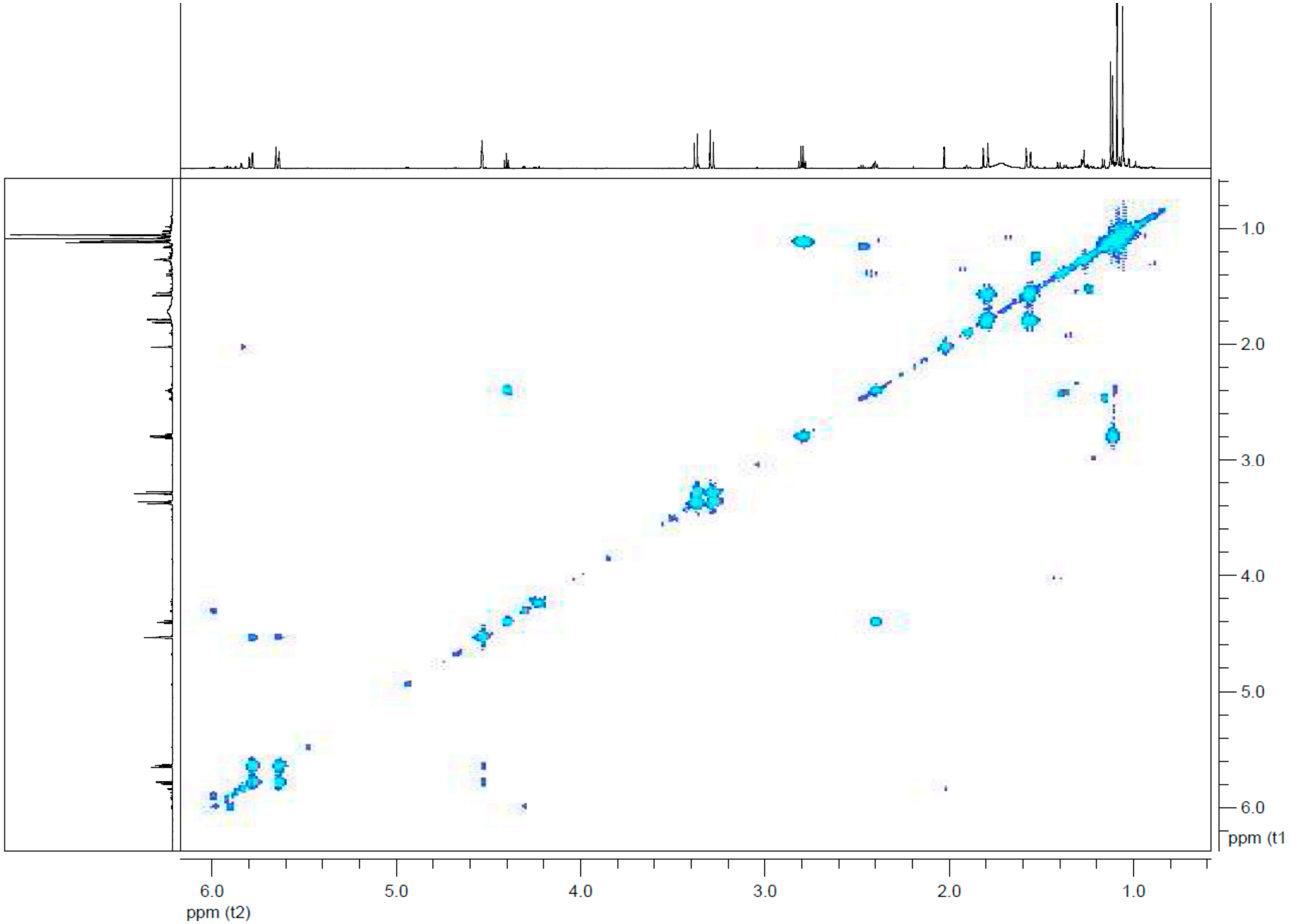

Figure S96. COSY (151 MHz, $CDCl_3$) spectrum of hydroxylactone **8b**.

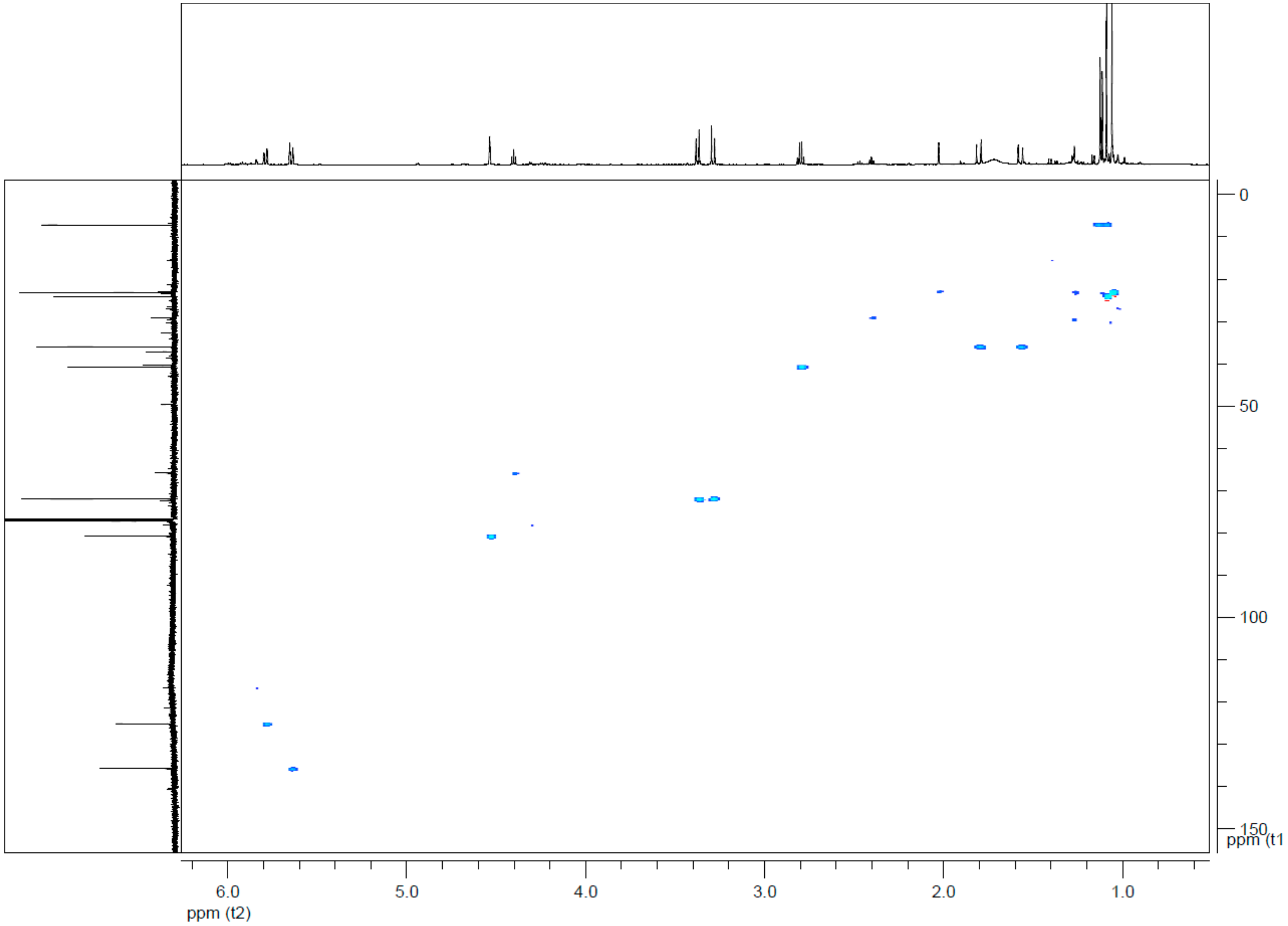

Figure S97. HMQC (151 MHz, $CDCl_3$) spectrum of hydroxylactone **8b**.

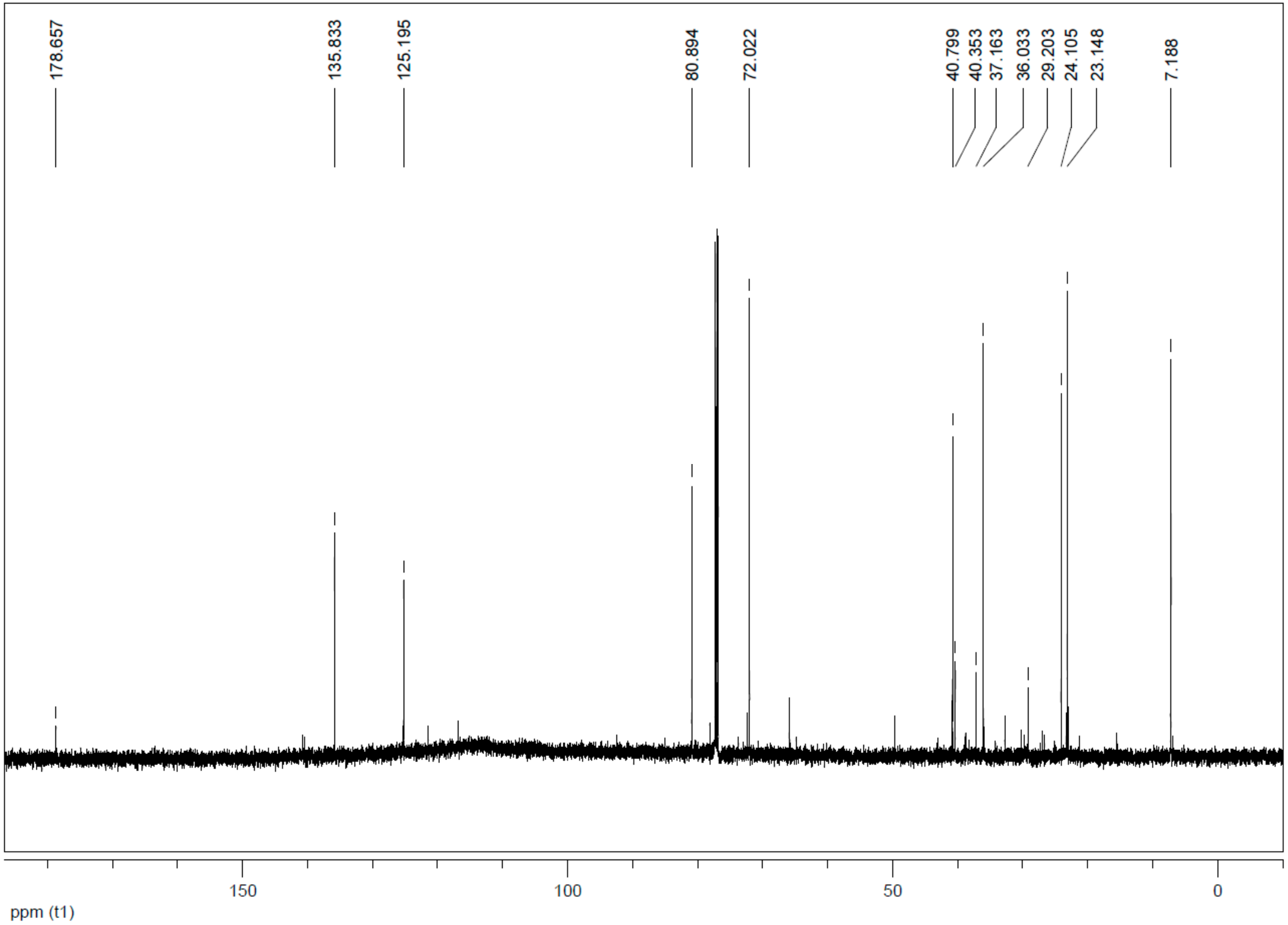

Figure S98. ^{13}C-NMR (151 MHz, $CDCl_3$) spectrum of hydroxylactone **8b**.

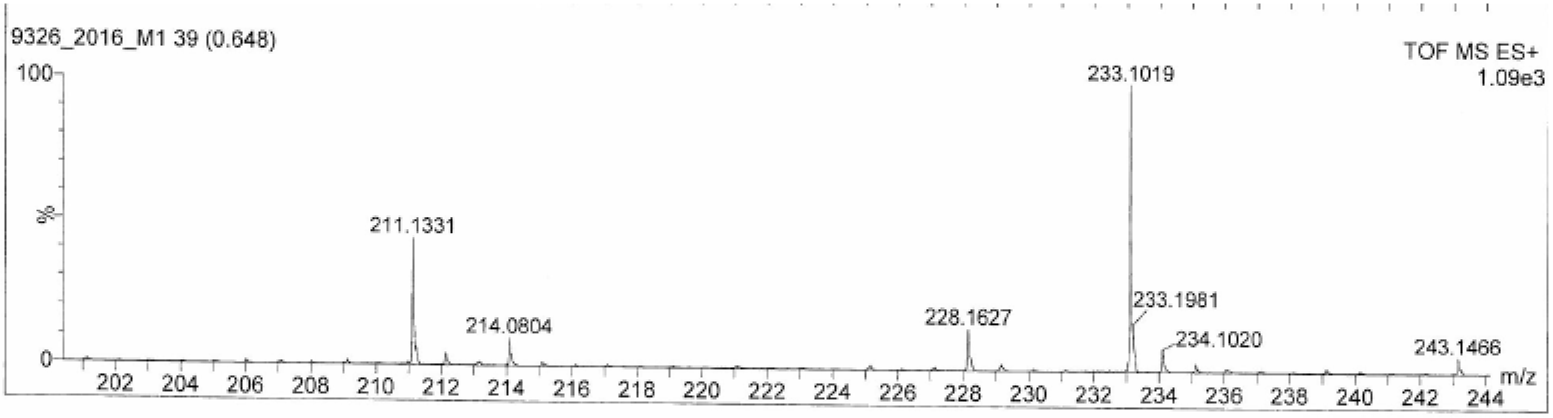

Figure S99. HRMS spectrum of hydroxylactone **8b**.

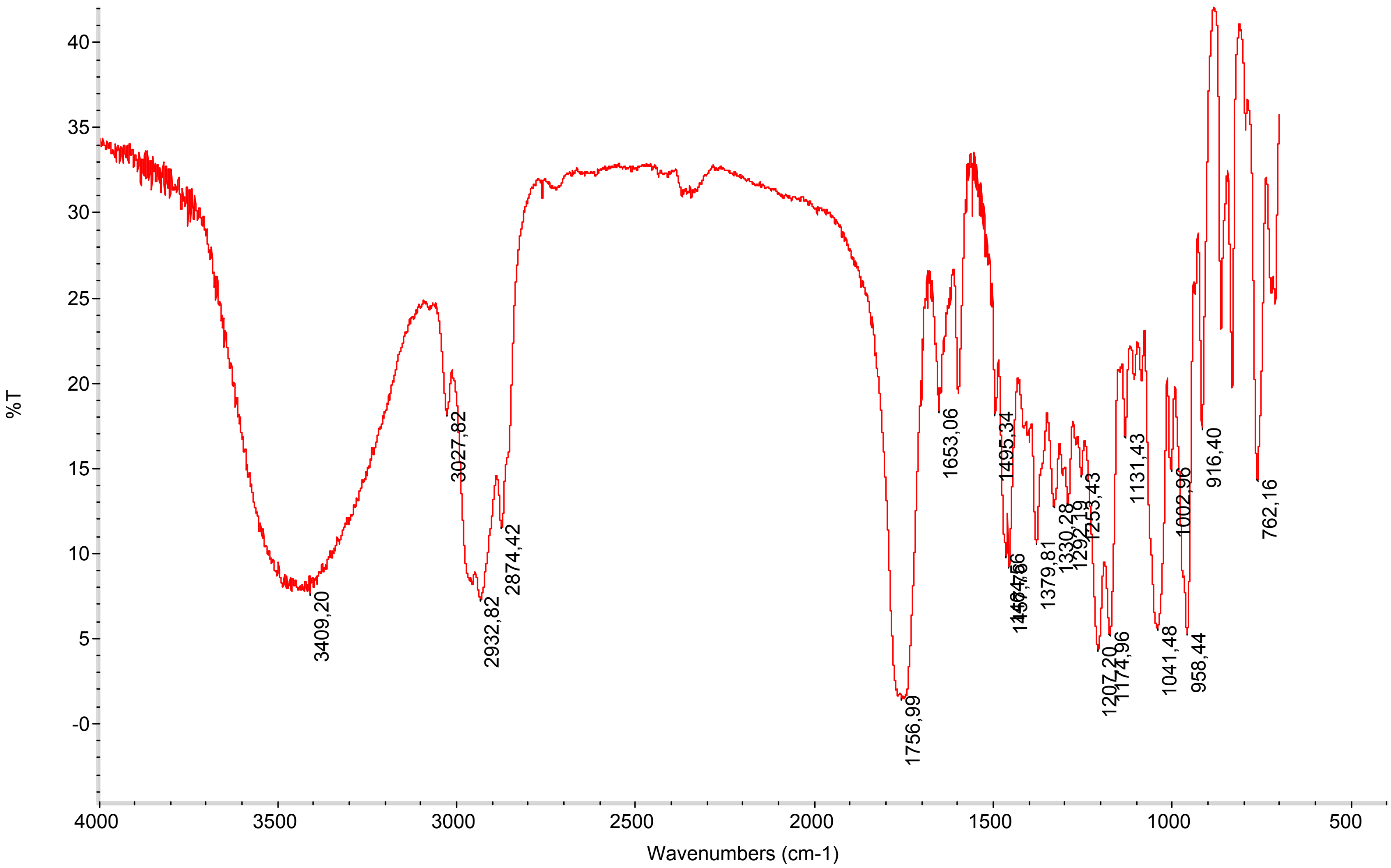

Figure S100. IR spectrum of hydroxylactone **8b**.

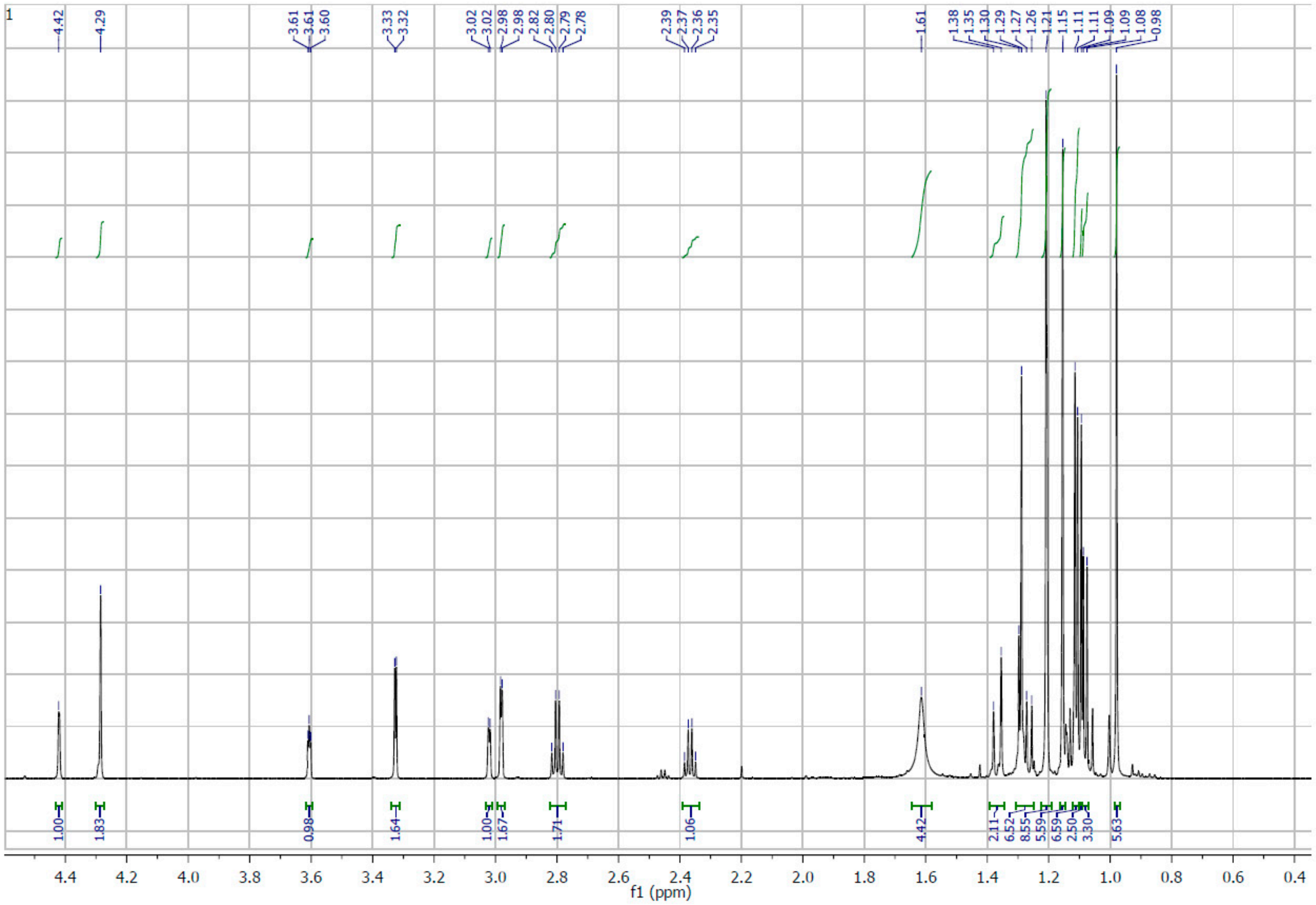

Figure S101. ^{1}H-NMR (600 MHz, $CDCl_3$) spectrum of epoxylactone **9b**.

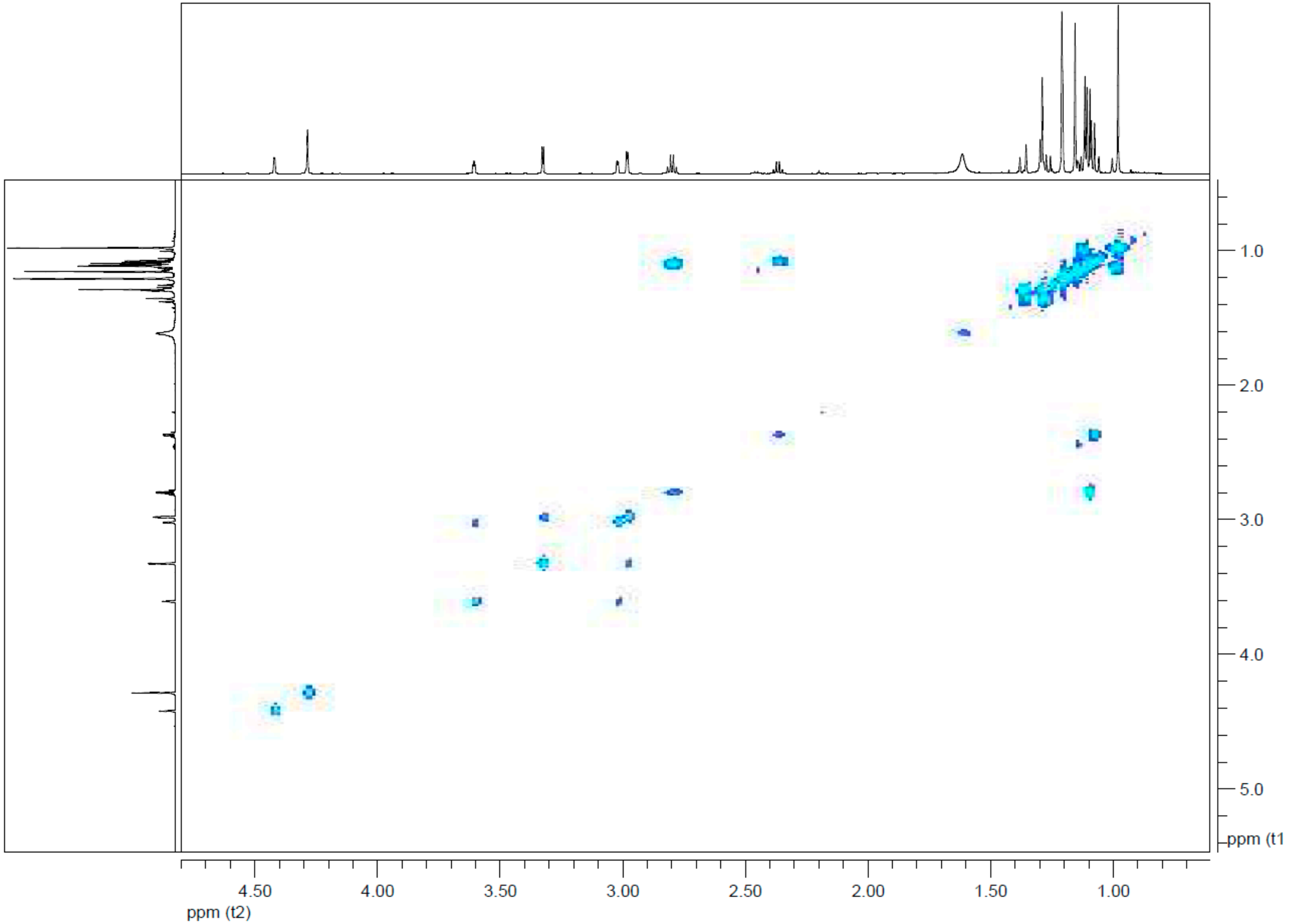

Figure S102. COSY (151 MHz, $CDCl_3$) spectrum of epoxylactone **9b**.

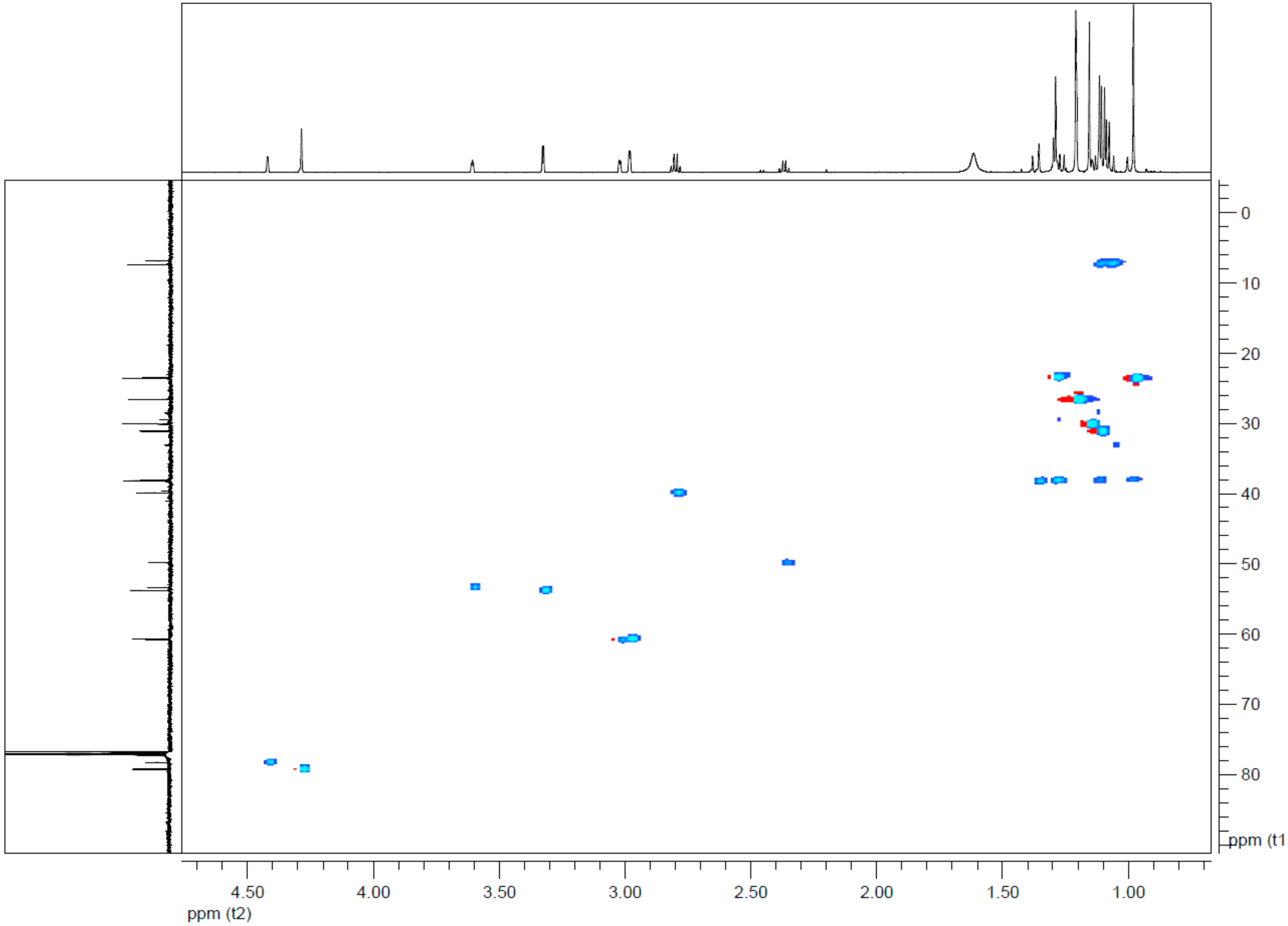

Figure S103. HMQC (151 MHz, CDCl₃) spectrum of epoxylactone **9b**.

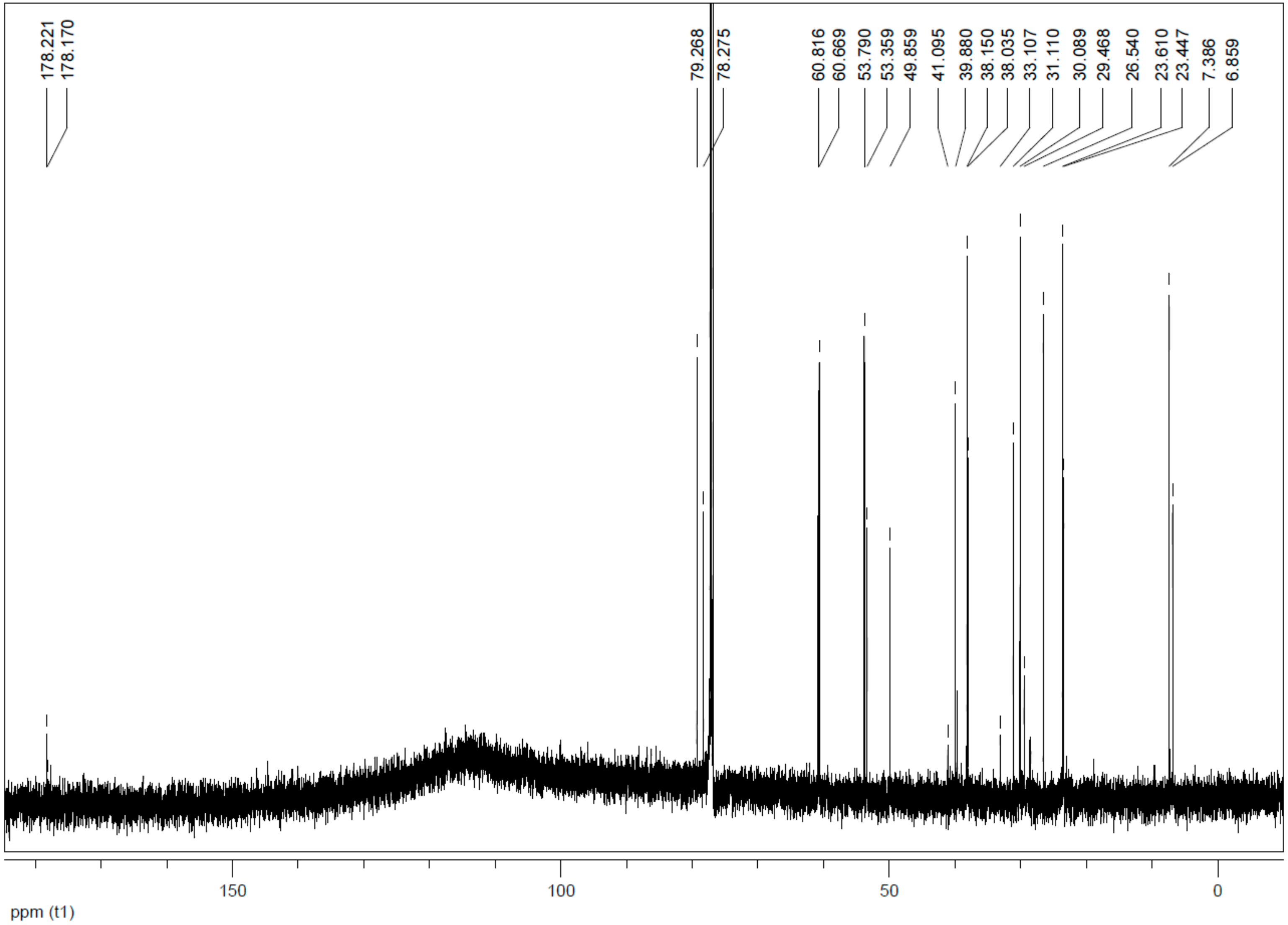

Figure S104. ^{13}C-NMR (151 MHz, $CDCl_3$) spectrum of epoxylactone **9b**.

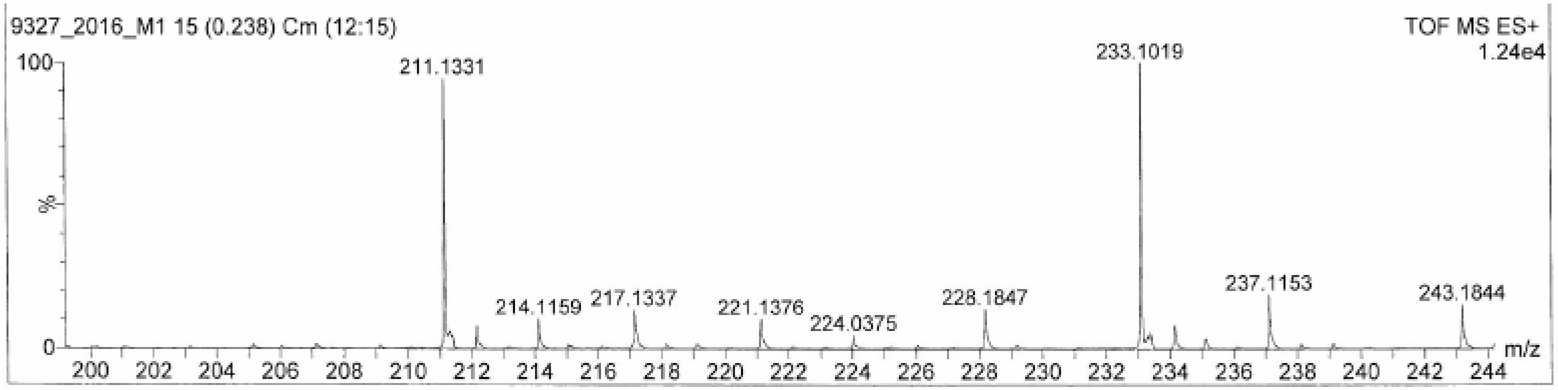

Figure S105. HRMS spectrum of epoxylactone **9b**.

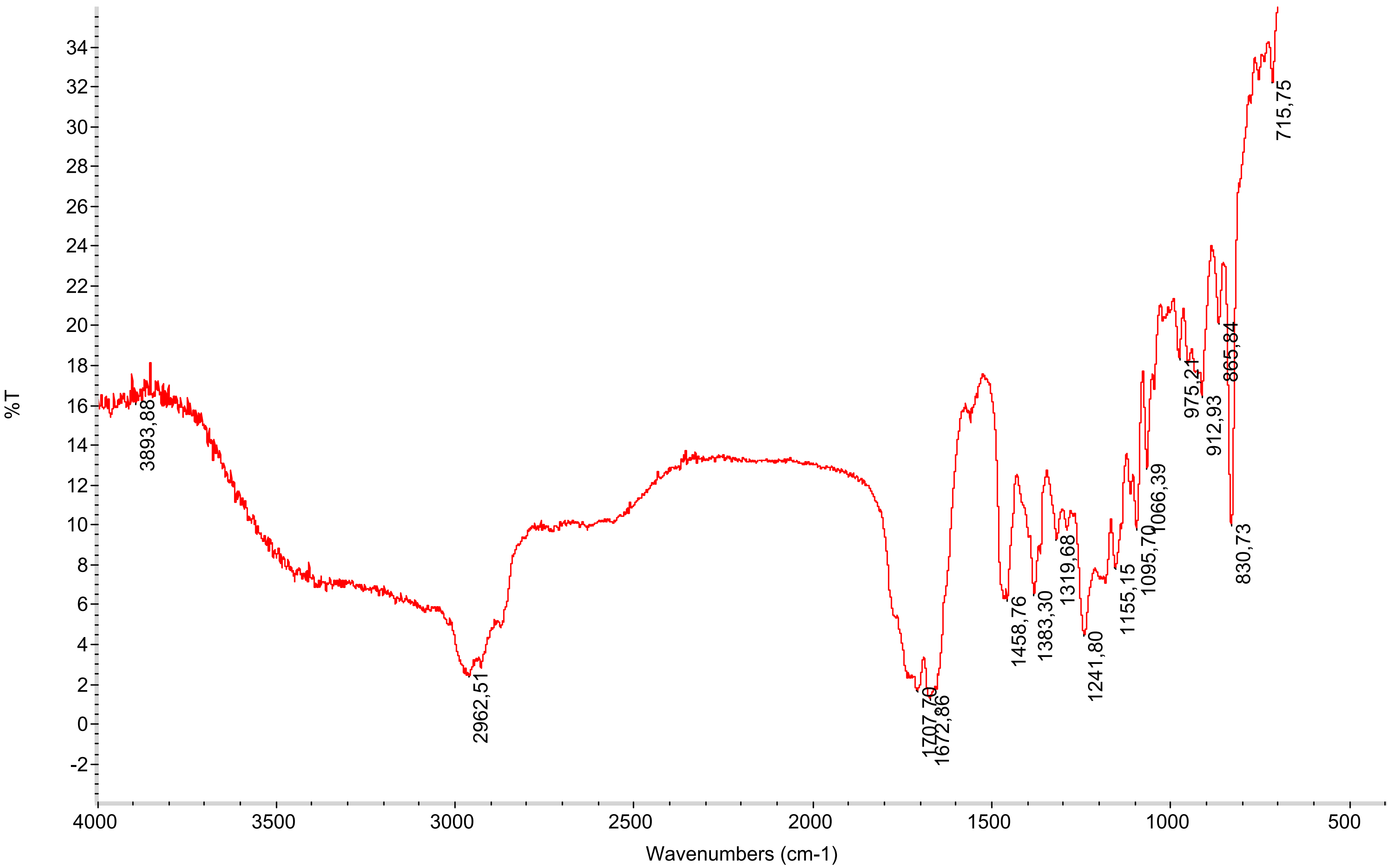

Figure S106. IR spectrum of epoxylactone **9b**.

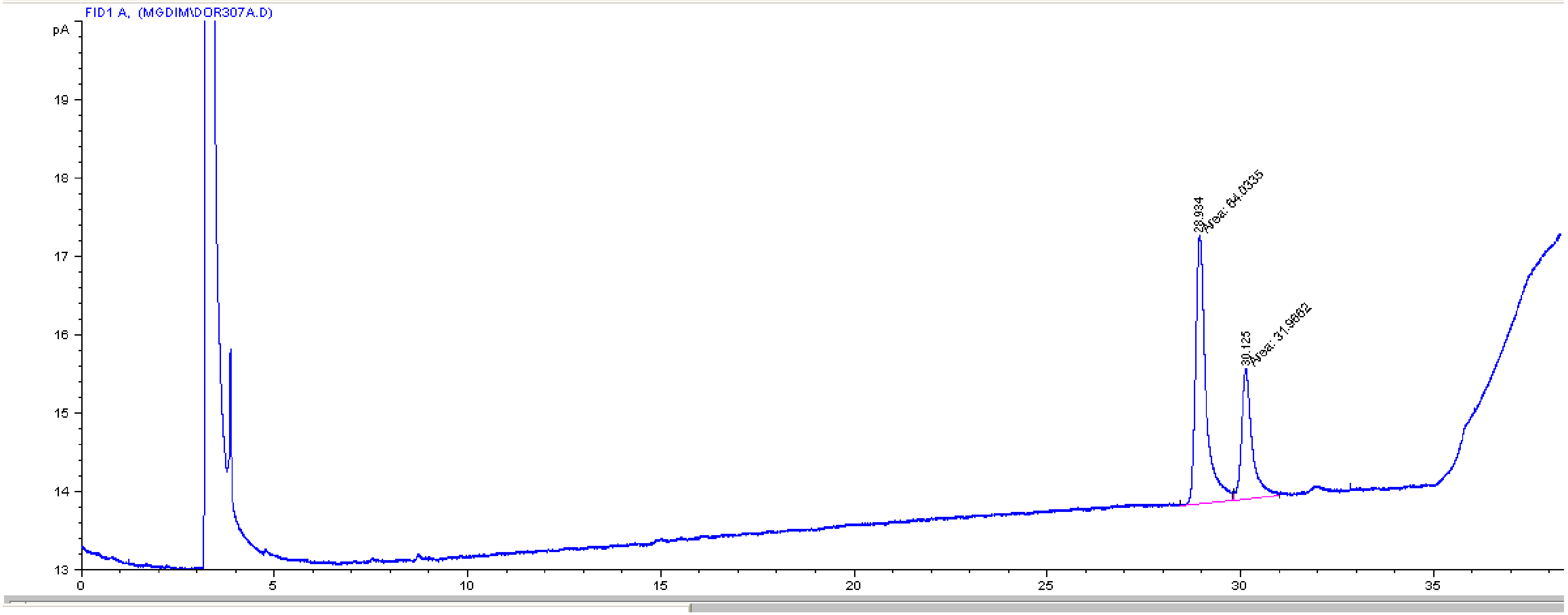

#	Time	Area	Height	Width	Area%	Symmetry
1	28.934	64	3.4	0.311	66.702	0.622
2	30.125	32	1.7	0.3188	33.298	0.608

Figure S107. Chiral chromatogram of lactone **7a** (*P. vermiculatum* AM30).

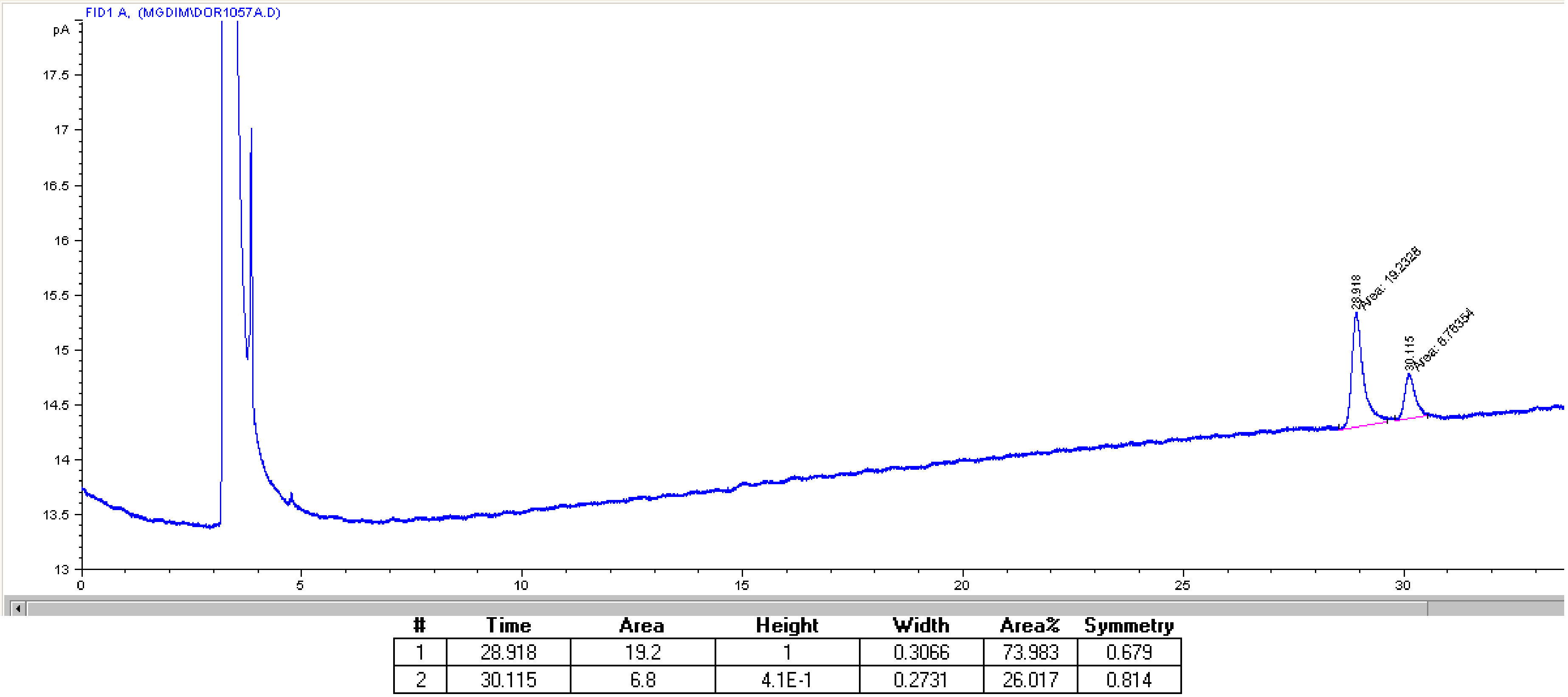

#	Time	Area	Height	Width	Area%	Symmetry
1	28.918	19.2	1	0.3066	73.983	0.679
2	30.115	6.8	4.1E-1	0.2731	26.017	0.814

Figure S108. Chiral chromatogram of lactone **7a** (*S. racemosum* AM105).

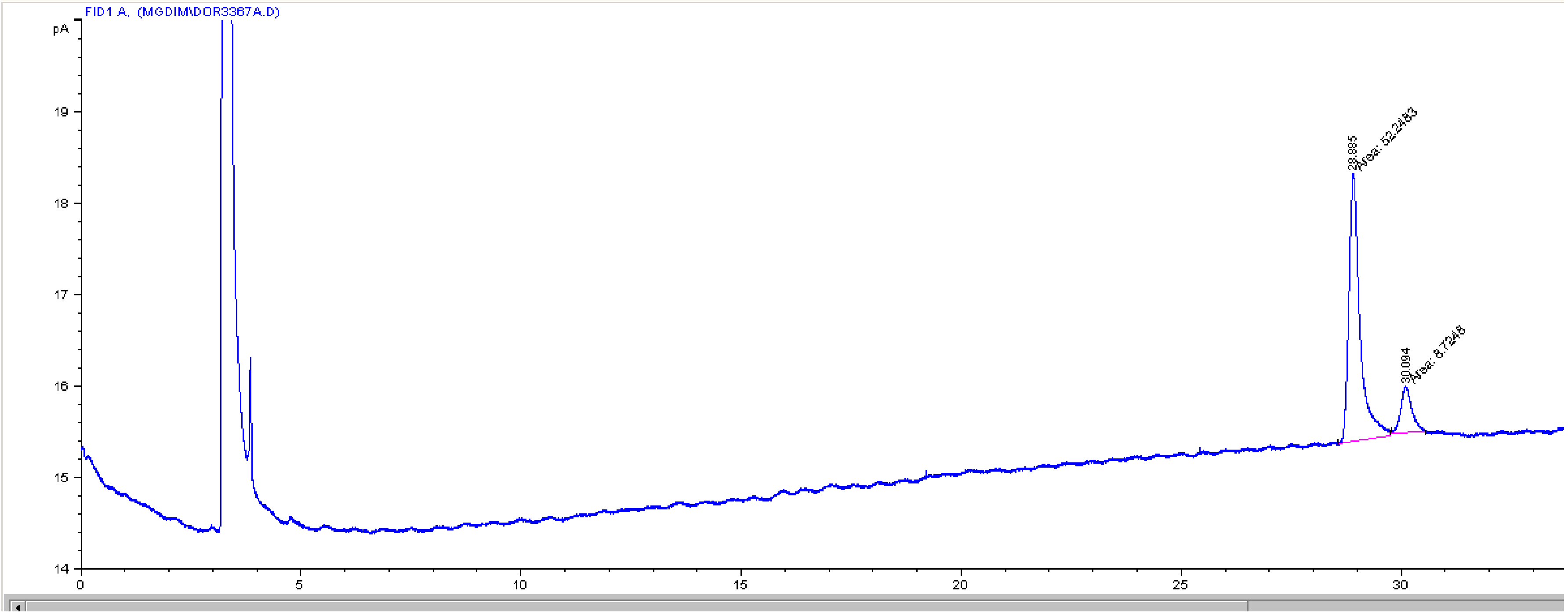

#	Time	Area	Height	Width	Area%	Symmetry
1	28.885	52.2	2.9	0.2971	85.691	0.541
2	30.094	8.7	5.1E-1	0.284	14.309	0.838

Figure S109. Chiral chromatogram of lactone **7a** (*A. cylindrospora* AM336).

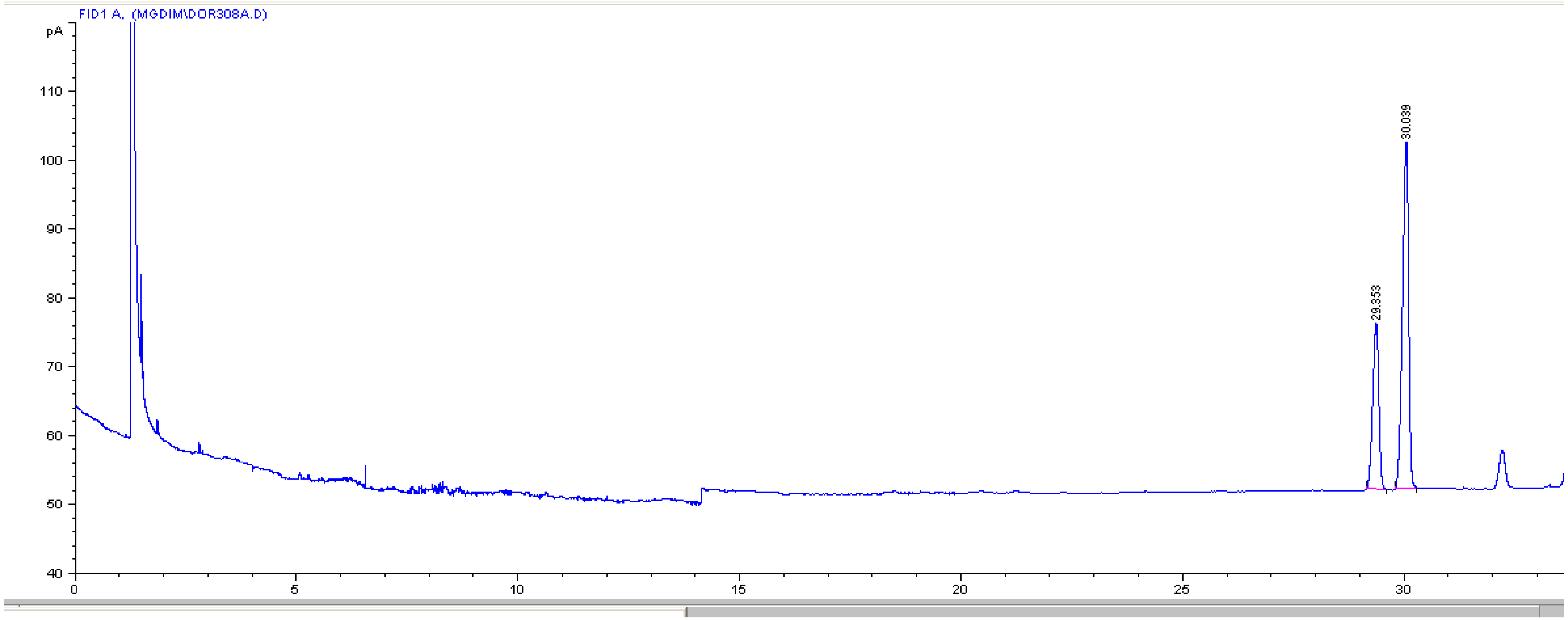

#	Time	Area	Height	Width	Area%	Symmetry
1	29.353	228	24.1	0.1293	32.450	1.242
2	30.039	474.5	50.3	0.1276	67.550	1.613

Figure S110. Chiral chromatogram of lactone **8a** (*P. vermiculatum* AM30).

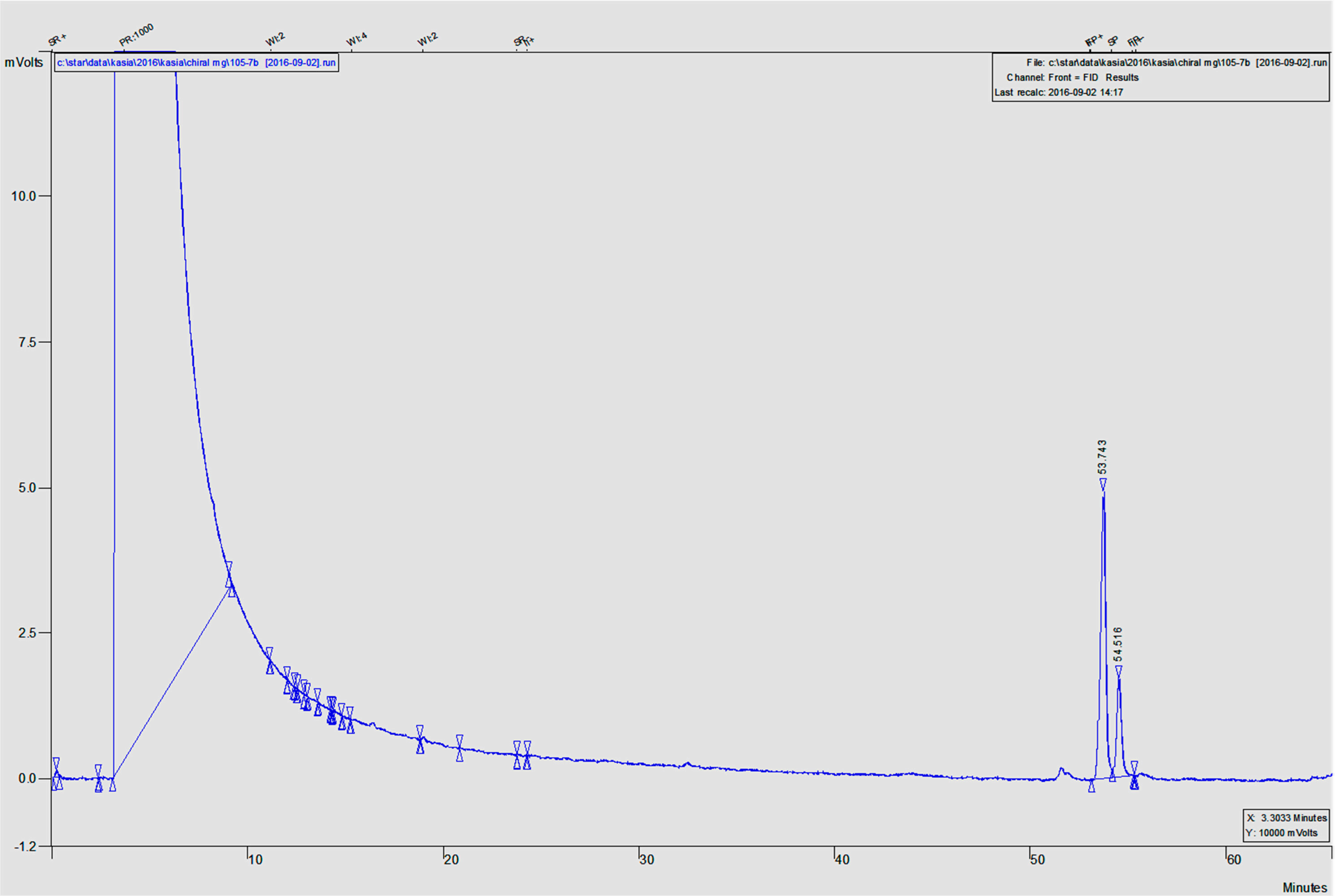
SR+
PR:1000
WI:2
WI:4
WI:2
mVolts
c:\star\data\kasia\2016\kasia\chiral mg\105-7b [2016-09-02].run
File: c:\star\data\kasia\2016\kasia\chiral mg\105-7b [2016-09-02].run
Channel: Front = FID Results
Last recalc: 2016-09-02 14:17
10.0
7.5
5.0
2.5
0.0
-1.2
53.743
54.516
X: 3.3033 Minutes
Y: 10000 mVolts
10
20
30
40
50
60
Minutes

Peak No.	Peak Name	Result ()	Ret. Time Time (min)	Time Offset (min)	Area (counts)	Sep. Code	Width 1/2 (sec)	Status Codes
1		75.4178	53.743	0.000	93428	BV	0.0	
2		24.5822	54.516	0.000	30452	VB	14.7	
Totals:		100.0000		0.000	123880			

Total Unidentified Counts : 123880 counts

Figure S111. Chiral chromatogram of lactone **7b** (*S. racemosum* AM105).

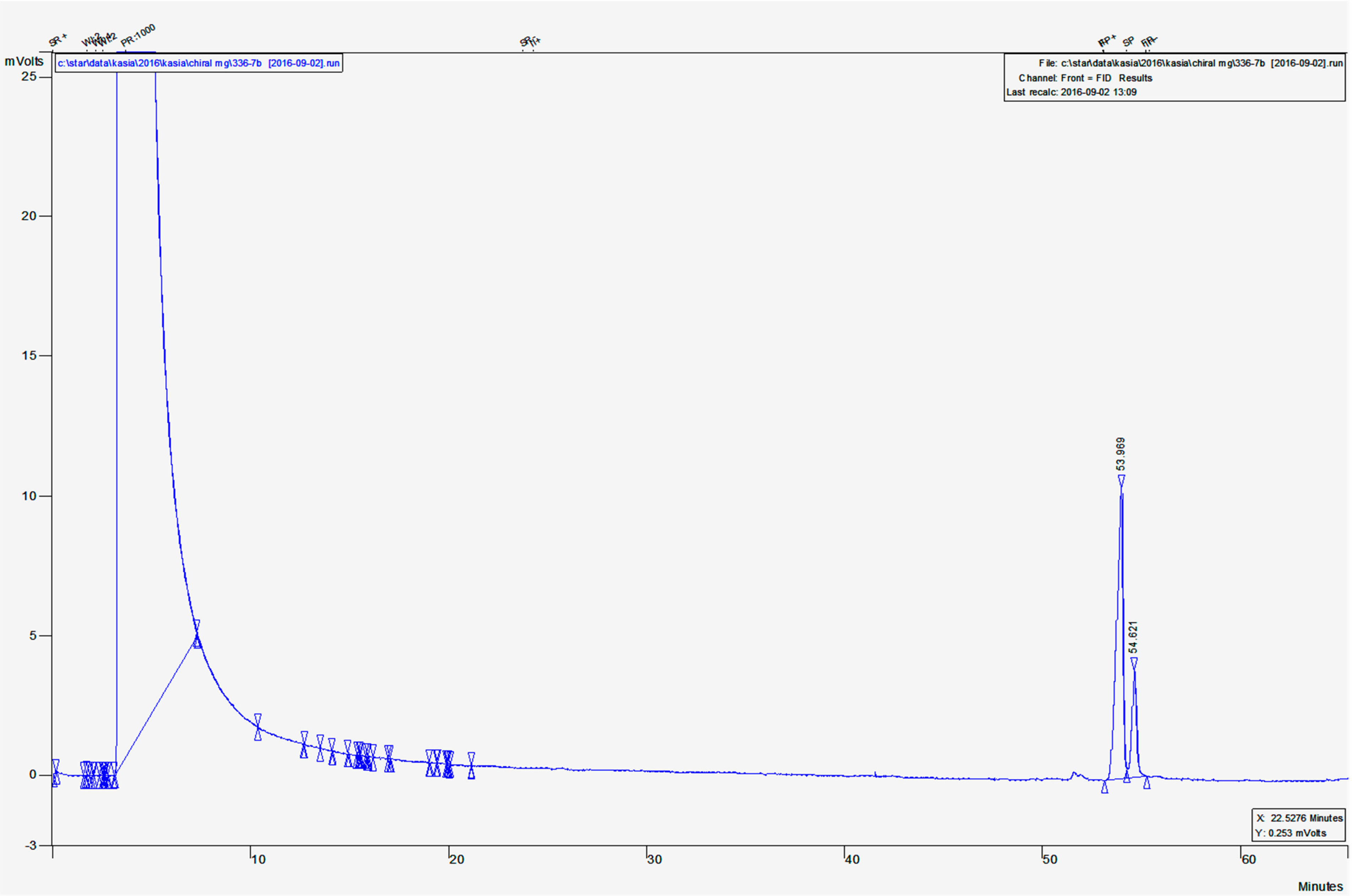
c:\star\data\kasia\2016\kasia\chiral mg\336-7b [2016-09-02].run
File: c:\star\data\kasia\2016\kasia\chiral mg\336-7b [2016-09-02].run
Channel: Front = FID Results
Last recalc: 2016-09-02 13:09
mVolts
Minutes
53.969
54.621
X: 22.5276 Minutes
Y: 0.253 mVolts

Peak No.	Peak Name	Result ()	Ret. Time (min)	Time Offset (min)	Area (counts)	Sep. Code	Width 1/2 (sec)	Status Codes
1		78.6297	53.969	0.000	238261	BV	0.0	
2		21.3703	54.621	0.000	64755	VB	14.1	
Totals:		100.0000		0.000	303016			

Total Unidentified Counts: 303016 counts

Figure S112. Chiral chromatogram of lactone **7b** (*A. cylindrospora* AM336).

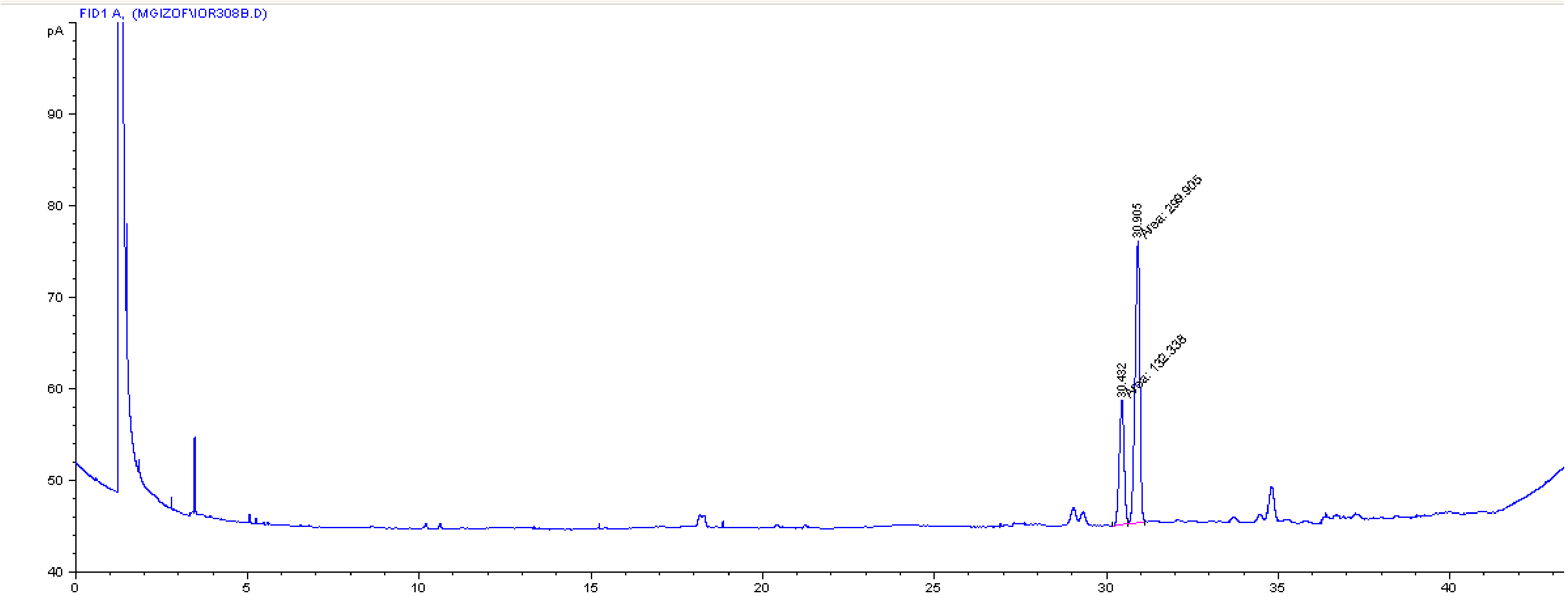

Figure S113. Chiral chromatogram of lactone **8b** (*P. vermiculatum* AM30).
